# Vietnamese pharmacogenomic variation in global context: a systematic review and meta-analysis for clinical implementation

**DOI:** 10.3389/fphar.2026.1921144

**Published:** 2026-09-03

**Authors:** Van Thi Bich Hoang, Anh Van Tran, Tham Thi Bui, Quynh Thi Vu, Mai Thi Quynh Ngo, Khai Van Nguyen, Phuong Thi Thu Nguyen

**Affiliations:** 1 Faculty of Pharmacy, Hai Phong University of Medicine and Pharmacy, Hai Phong, Vietnam; 2 Center for Applied Biomedical Research, Hai Phong University of Medicine and Pharmacy, Hai Phong, Vietnam; 3 Faculty of Public Health, Hai Phong University of Medicine and Pharmacy, Hai Phong, Vietnam; 4 Department of Pharmacy, Hai Phong International Hospital, Hai Phong, Vietnam

**Keywords:** allele frequency, clinical implementation, meta-analysis, pharmacogenetics, pharmacogenomics, population genetics, precision medicine, systematic review

## Abstract

**Background:**

Population-frequency evidence is indispensable for planning pharmacogenomic services, but broad ancestry categories do not reliably capture the distribution of clinically important alleles, haplotypes, and structural variants. We undertook a systematic synthesis of pharmacogenetic variation reported in Vietnamese populations and evaluated its relevance to clinical implementation.

**Methods:**

Following a prospectively registered protocol and PRISMA 2020, we searched international and Vietnamese sources through 30 December 2025. Reports were eligible when they described Vietnamese participants and provided, or materially informed, pharmacogenetic genotype or allele-frequency evidence. Variant representations were reconciled across rsIDs, star alleles, haplotypes, copy-number changes, hybrid alleles, repeats, and HLA alleles. For variants with at least two independent rows, frequencies were synthesized with random-effects logit models; single-row estimates used Wilson confidence intervals. Orientation-validated single-rsID variants were compared with the 1,000 Genomes AFR, AMR, EAS, EUR, and SAS superpopulations with false-discovery-rate control.

**Results:**

Of 207 records, 54 full-text reports were assessed and 52 were retained in the systematic review; 46 independent reports or datasets contributed to the primary meta-analysis. The evidence comprised 108 analysis-ready variant representations across 25 pharmacogenes, including 66 single-rsID and 42 complex or non-rsID representations. Ninety-five representations were supported by one study row. Among included reports, 11 were at low, 38 at moderate, and 3 at high risk of bias; none of the 46 quantitative reports was classified as high risk. Prominent estimates were *VKORC1* -1639G>A, 89.91% (95% CI 81.13-94.86); *VKORC1* 1173C>T, 85.43% (81.61-88.56); *SLCO1B1* c.388A>G, 75.95% (69.75-81.22); CYP3A5*3, 60.81% (44.96-74.67); *ABCB1* 3435C>T, 40.28% (32.62-48.44); *ABCG2* c.421C>A, 36.00% (29.67-42.86); CYP2C19*2, 27.90% (26.57-29.27); NAT2*6, 27.00% (18.69-37.31); and CYP2C19*3, 5.74% (4.82-6.82). Eighteen of 20 variants in the principal global comparison were within 5 percentage points of EAS; larger differences occurred for *ABCB1* 3435C>T (−19.9 points) and CYP3A5*3 (−7.2 points).

**Conclusion:**

Vietnamese pharmacogenomic frequencies are regionally East Asian-adjacent but not interchangeable with a generic East Asian reference. Implementation should combine local frequency evidence with guideline strength, preventable clinical harm, medication use, and assay complexity, while preserving haplotype and structural-variant information.

**Systematic Review Registration:**

https://doi.org/10.17605/OSF.IO/7VTNW, identifier 7VTNW.

## 1 Introduction

Pharmacogenomics has moved from a catalogue of gene-drug associations toward an implementation science that links genotype, phenotype, prescribing guidance, laboratory quality, clinical decision support, and patient outcomes. Guideline consortia now provide actionable recommendations across cardiovascular medicine, transplantation, psychiatry, infectious diseases, oncology, pain management, and immunologically mediated adverse drug reactions. Regulatory labels increasingly include pharmacogenomic biomarkers, although the implied action and evidentiary threshold vary between agencies and products (Kim et al., 2021; Ehmann et al., 2015). The PREemptive Pharmacogenomic testing for prevention of Adverse drug REactions (PREPARE) trial further showed that a multigene strategy can reduce clinically relevant adverse drug reactions when results are embedded in routine prescribing rather than reported as isolated laboratory findings (Evans and McLeod, 2003; Roden et al., 2019; Relling and Evans, 2015; Caudle et al., 2014; Relling et al., 2020; Clinical Pharmacogenetics Implementation Consortium, 2019; Swen et al., 2011; ClinPGx, 2024; Whirl-Carrillo et al., 2021; Pirmohamed, 2023; Swen et al., 2023).

Population evidence matters at a different level from individual prescribing. Allele and diplotype frequencies influence the expected yield of pre-emptive testing, panel composition, residual risk after a negative result, reagent design, quality-control materials, and the economic case for implementation. They do not, however, justify assigning a pharmacogenomic phenotype from self-identified ethnicity or presumed ancestry. The clinically relevant unit remains the patient’s measured genotype interpreted with co-medications, organ function, age, disease state, and other non-genetic determinants. This distinction is particularly important when a star allele is defined by a haplotype, when copy number or hybrid structure changes phenotype, or when an HLA allele cannot be reduced to a nearby tag variant (Gaedigk et al., 2018; Landrum et al., 2018; Auton et al., 2015; Karczewski et al., 2020; Popejoy and Fullerton, 2016; Sirugo et al., 2019; Gaedi and gk, 2013; Gaedigk et al., 2017; Ingelm et al., 2018; Lauschke and Ingelman-Sundberg, 2020; Huddart et al., 2019).

The evidence base used to build pharmacogenomic resources remains geographically uneven. The closest precedents to a country-focused synthesis are large population-frequency surveys and regional sequencing initiatives, including SEAPharm and GenomeAsia, rather than a directly comparable national meta-analysis with report-level source auditing. Global projects such as the 1000 Genomes Project and gnomAD have transformed population-genetic reference data, while Southeast Asian initiatives have documented substantial within-region diversity across pharmacogenes. These resources are essential, but they are not substitutes for a country-specific, study-level synthesis: reference datasets may contain limited numbers from any one population, use different ascertainment and sequencing strategies, and rarely capture all local clinical or Vietnamese-language reports (Auton et al., 2015; Karczewski et al., 2020; Popejoy and Fullerton, 2016; Sirugo et al., 2019; Runcharoen et al., 2021; GenomeAsia100K Consortium, 2019).

Vietnam provides a compelling setting for such a synthesis. The Kinh constitute the majority population, while diverse ethnic-minority communities are distributed unevenly across regions; yet most available pharmacogenetic evidence concerns participants described broadly as Vietnamese or explicitly as Kinh, and minority-population evidence remains sparse. Reports span healthy volunteers, hospital cohorts, transplant recipients, cardiovascular disease, epilepsy, tuberculosis, oncology, and adverse-reaction studies. They also use heterogeneous technologies and nomenclatures, ranging from PCR-based single-variant assays to targeted sequencing, pyrosequencing, HLA typing, copy-number analysis, and star-allele inference. Consequently, apparently simple questions - such as the national frequency of a clinically relevant allele - can be distorted by duplicated cohorts, clinically selected samples, allele-orientation errors, or the merging of biologically non-equivalent representations.

Previous Vietnamese investigations have generated important gene-specific evidence. Studies of *CYP2D6* have identified reduced-function alleles, whole-gene deletion, tandem arrangements, and hybrid structures; studies of *CYP2C19* have documented common loss-of-function alleles and population variation; and multigene surveys have placed Vietnamese frequencies in a broader Asian context (Nguyen et al., 2019; Veiga et al., 2009; Vu et al., 2019). More recent Southeast Asian and fixed-panel datasets extend the breadth of variant discovery (Runcharoen et al., 2021). None of these designs, however, provides a bilingual, report-level audit of the full Vietnamese literature with duplicate control, denominator reconstruction, harmonized effect-allele orientation, random-effects synthesis, and explicit separation of single-rsID comparisons from complex-allele interpretation.

That separation has direct clinical consequences. Warfarin algorithms require a multivariable interpretation of *VKORC1*, *CYP2C9*, and *CYP4F2*; *CYP2C19* diplotypes affect clopidogrel, proton-pump inhibitors, and selected antidepressants; *CYP3A5* influences tacrolimus starting dose but not the need for therapeutic drug monitoring; *SLCO1B1* and *ABCG2* inform statin therapy; *UGT1A1*, *NUDT15*, *TPMT*, and *HLA-B* can identify preventable toxicity; and *CYP2D6* demands copy-number- and structure-aware testing (Johnson et al., 2017; Klein et al., 2009; Gage et al., 2008; Lee et al., 2022; Lima et al., 2021; Bousman et al., 2023; Crews et al., 2021; Cooper-DeHoff et al., 2022; Ramsey et al., 2014; Hulshof et al., 2023; Innocenti et al., 2004; Hershfield et al., 2013; Birdwell et al., 2015; Relling et al., 2019; Saito et al., 2016). A frequency synthesis that collapses these loci to a uniform list of SNPs would therefore be statistically convenient but clinically incomplete.

We aimed to define the Vietnamese pharmacogenomic evidence landscape at the level needed for responsible implementation. Specifically, we (i) synthesized allele and genotype-frequency evidence from Vietnamese participants; (ii) benchmarked orientation-validated single-rsID variants against major global superpopulations; (iii) retained star alleles, haplotypes, HLA alleles, repeats, and structural variants in their clinically meaningful form; and (iv) organized the evidence by implementation relevance rather than frequency alone. The intended contribution is not a national prescribing algorithm, but a transparent foundation for selecting assays, prioritizing prospective studies, and avoiding uncritical extrapolation from broad regional references.

## 2 Methods

### 2.1 Protocol registration, reporting, and review question

The protocol was prospectively registered on the Open Science Framework on 20 November 2025 (doi:10.17605/OSF.IO/7VTNW) and specified eligibility criteria, search sources, outcomes, risk-of-bias domains, and the planned synthesis (Phuong, 2025). The final database search was completed on 30 December 2025. Reporting followed PRISMA 2020 and was informed by PRISMA-P (Page et al., 2021; Shamseer et al., 2015). In this manuscript, a record denotes a database or supplementary-search entry, a report denotes a retrievable publication or document, and an independent dataset denotes a non-overlapping Vietnamese sample contributing to a quantitative estimate.

The PICOS framework was adapted for a genetic-frequency review. The population was participants of Vietnamese origin; the exposure was carriage of pharmacogenetically relevant variation; comparators were global reference populations when effect-allele orientation was unambiguous; outcomes were allele and genotype frequencies, between-population differences, and implementation relevance; and eligible designs included population surveys, cross-sectional and cohort studies, case-control studies with an eligible unselected comparator group, and clinical or pharmacokinetic studies reporting baseline genotype data.

### 2.2 Eligibility criteria

Reports were eligible for the primary quantitative synthesis when they provided reconstructable genotype counts, allele counts, or allele frequencies for at least 30 Vietnamese participants. Mixed-population reports were eligible only when the Vietnamese denominator and allele orientation could be isolated. Participants were not restricted by age, sex, health status, treatment indication, or clinical setting, but clinically selected adverse-reaction case series without an unselected denominator were excluded from population-frequency estimation.

The review covered genes involved in absorption, distribution, metabolism, excretion, immune-mediated drug reactions, pharmacodynamic targets, and regulatory pharmacogenomics. Prespecified genes included *CYP2C19*, *CYP2D6*, *CYP2C9*, *CYP3A5*, *CYP2B6*, *CYP2A6*, *UGT1A1*, *NAT2*, *DPYD*, *TPMT*, *VKORC1*, *CYP4F2*, *SLCO1B1*, *ABCB1*, *ABCG2*, and *HLA-B*. Additional genes were retained when a report provided a clear pharmacogenetic rationale and extractable Vietnamese data.

Reports were excluded from quantitative synthesis when they lacked a valid Vietnamese denominator, reported only a phenotype without corresponding genotype or allele data, used non-human or in vitro-only designs, were narrative articles without original population data, or could not establish Vietnamese origin. Six reports without an independent analysis-ready row were retained only in the qualitative evidence map because they contributed contextual information on assay development, variant representation, or linked reporting. They did not affect any pooled estimate.

### 2.3 Information sources and search strategy

PubMed/MEDLINE, Embase, Scopus, and Web of Science were searched, supplemented by backward and forward citation tracking. Vietnamese and regional retrieval included Vietnam Journals Online and national biomedical journals. Google Scholar was used only to locate local reports and follow citation trails, not as a stand-alone source of quantitative evidence.

English terms combined Vietnam, Vietnamese, Kinh, and ethnic-group terms with pharmacogenetic, pharmacogenomic, allele frequency, genotype frequency, polymorphism, SNP, variant, and gene-specific terms. Vietnamese terms included 'đa hình di truyền,' 'tần suất allele,' 'tần suất alen,' 'kiểu gen,' 'người Việt,' 'dân tộc Kinh,' and 'dân tộc thiểu số.' The complete database strategies and search dates are provided in the registered protocol and Supplementary Material. Comparator frequencies were drawn primarily from 1,000 Genomes Phase 3; gnomAD, PharmGKB, PharmVar, and ClinVar were used for nomenclature or functional annotation rather than mixed with 1,000 Genomes in the principal pairwise tests (Whirl-Carrillo et al., 2021; Gaedigk et al., 2018; Landrum et al., 2018; Auton et al., 2015; Karczewski et al., 2020).

### 2.4 Study selection and report linkage

Two reviewers independently screened titles and abstracts and assessed potentially eligible full texts. Disagreements were resolved by discussion and, when needed, adjudication by a senior reviewer. Full-text review recorded eligibility, population description, sample size, genotyping method, clinical context, and availability of reconstructable variant-level data. Reports suspected of describing the same cohort were linked by institution, recruitment period, sample size, population characteristics, and genotype distributions; only one independent instance contributed to a given primary estimate.

### 2.5 Data extraction and variant harmonization

A piloted extraction form captured study identifier, year, region, ethnicity, clinical context, sample size, genotyping method, gene, reported variant name, rsID, reference and alternate alleles, genotype counts, allele counts, frequency, Hardy-Weinberg equilibrium status, and source page. One reviewer extracted data and a second independently checked denominators, allele orientation, nomenclature, and source traceability. The complete row-level audit is presented in Supplementary Table S2.

For a biallelic variant, the designated variant-allele frequency was calculated as AF = (2nVV + nRV)/(2N), where nVV and nRV are the variant-homozygote and heterozygote counts and N is the number genotyped. Orientation was reversed when the clinically relevant or comparison allele was the reference allele. Rows with irreconcilable strand, allele, or denominator information were not entered into quantitative synthesis.

Single-nucleotide variants were harmonized by gene, rsID, reference allele, alternate allele, and effect-allele orientation. Synonymous study labels that mapped to the same rsID and allele direction were collapsed for the main synthesis; this affected *CYP2B6* rs3745274, *SLCO1B1* rs2306283, and *VKORC1* rs9934438. Star alleles, HLA alleles, haplotypes, copy-number variants, hybrid alleles, deletions, and repeat polymorphisms were retained as separate clinically meaningful representations. A single marker was not relabeled as a star allele unless the source supported the defining haplotype or structural call. This rule is especially important for *CYP2D6*, for which rs1065852 alone does not establish CYP2D6*10 and rs3892097 alone does not resolve all structural contexts of CYP2D6*4.

### 2.6 Risk-of-bias assessment

Risk of bias was evaluated with a six-domain framework adapted for genetic epidemiology from the Newcastle-Ottawa approach and the registered protocol. The protocol listed five items but specified a six-point scoring range; before outcome synthesis, we operationalized the framework as six domains by separating clarity of Vietnamese origin from sample representativeness and by incorporating report linkage or duplicate-sampling concerns into the Hardy-Weinberg/reporting domain. The domains were sample representativeness, sample-size adequacy, clarity of Vietnamese origin, genotyping quality, completeness of genotype or allele reporting, and Hardy-Weinberg/reporting or duplicate-sampling concerns. Each domain received 1 point when adequately addressed and 0 when unclear or inadequate. Total scores of 5–6, 3-4, and 0-2 were interpreted as low, moderate, and high risk, respectively. Domain-level judgments were retained in the audit file; Supplementary Table S1 reports the total score and analytic status.

### 2.7 Meta-analysis of Vietnamese frequencies

Variants represented by at least two independent Vietnamese rows were synthesized on the logit scale with a random-effects model and DerSimonian-Laird estimation of between-study variance; pooled estimates and 95% confidence intervals were back-transformed to the proportion scale (Barendregt et al., 2013; DerSimonian and Laird, 1986). Single-row estimates were reported with Wilson 95% confidence intervals. Cochran’s Q and I^2^ characterized heterogeneity. I^2^ was interpreted together with the absolute spread and number of studies because a proportion meta-analysis can yield high relative heterogeneity even when absolute differences are modest (Higgins and Thompson, 2002).

Sensitivity analyses restricted the evidence by risk of bias, sample size, rsID availability, Hardy-Weinberg status, Kinh ethnicity, healthy or control sampling, and genotyping method. Freeman-Tukey double-arcsine models specified in the protocol were retained as a sparse-data sensitivity analysis and were not mixed with the primary logit estimates.

### 2.8 Cross-population benchmarking and population similarity

Formal global benchmarking was restricted to biallelic single-rsID variants with validated effect-allele orientation. Non-overlapping Vietnamese allele counts were compared with 1,000 Genomes AFR, AMR, EAS, EUR, and SAS superpopulation counts. Two-by-two allele tables yielded odds ratios, 95% confidence intervals, and two-sided p values; a Haldane-Anscombe correction was applied when a cell was zero. Benjamini–Hochberg adjustment controlled the false discovery rate across the retained variant-population comparisons (Benjamini and Hochberg, 1995). Absolute differences were calculated as Vietnamese minus comparator frequency in percentage points.

Principal component analysis used the standardized complete-case matrix shared by Vietnam and all five superpopulations. It was descriptive and was not used to infer individual ancestry, population admixture, or within-Vietnam structure. Because the 1,000 Genomes EAS superpopulation includes Kinh in Ho Chi Minh City (KHV), EAS was treated as a regional benchmark containing a Vietnamese component rather than a fully independent external comparator.

### 2.9 Clinical actionability and implementation framework

Clinical interpretation drew on CPIC and DPWG guidelines, PharmGKB and PharmVar, ClinVar, drug labels, and primary literature (Caudle et al., 2014; Relling et al., 2020; Clinical Pharmacogenetics Implementation Consortium, 2019; Swen et al., 2011; ClinPGx, 2024; Whirl-Carrillo et al., 2021; Gaedigk et al., 2018; Landrum et al., 2018; Johnson et al., 2017; Klein et al., 2009; Gage et al., 2008; Lee et al., 2022; Lima et al., 2021; Bousman et al., 2023; Crews et al., 2021; Cooper-DeHoff et al., 2022; Ramsey et al., 2014; Hulshof et al., 2023; Innocenti et al., 2004; Hershfield et al., 2013; Saksit et al., 2017; Gaedi and gk, 2013; Gaedigk et al., 2017; Ingelm et al., 2018; Lauschke and Ingelman-Sundberg, 2020; Birdwell et al., 2015; Relling et al., 2019; Saito et al., 2016; Desta et al., 2019; Caudle et al., 2020; Karnes et al., 2021; Theken et al., 2020). Classification considered four dimensions: strength of prescribing guidance; severity and preventability of the clinical outcome; expected test yield informed by Vietnamese frequency; and assay complexity. High priority indicated a mature or high-consequence gene-drug relationship with clear implementation relevance. Moderate priority indicated lower expected yield, narrower evidence, or substantial assay and interpretation complexity. The categories were intended for health-system planning and were not a validated clinical score.

Data processing, statistical analysis, orientation checks, duplicate control, and visualization were implemented in scripted Python and R workflows. Figure-source tables and audit outputs were retained. Frequencies were not converted directly into prescribing recommendations; clinical recommendations remained genotype- and drug-specific.

### 2.10 Protocol deviations

Three analytic deviations from the registered protocol were made before final interpretation. First, the primary proportion model was changed from Freeman-Tukey double-arcsine to a logit random-effects model because inverse double-arcsine transformation can produce misleading results, particularly with heterogeneous sample sizes; the prespecified model was retained as sensitivity analysis (Schwarzer et al., 2019). Second, funnel plots and Egger tests were not used because 95 of 108 representations had one study row and only two variants had at least 10 rows, making conventional small-study-effect diagnostics uninformative for most outcomes. Third, a formal GRADE rating was not assigned because the proposed adaptation had not been validated for heterogeneous population allele-frequency evidence and would have required unvalidated judgments for each representation; certainty was instead characterized through risk of bias, precision, heterogeneity, representation, and sensitivity analyses. The six-domain risk-of-bias operationalization described above and the retention of six non-quantitative reports in the qualitative map, while excluding them from all estimates, were documented as post-registration clarifications.

## 3 Results

### 3.1 Study selection and characteristics

**FIGURE 1:**
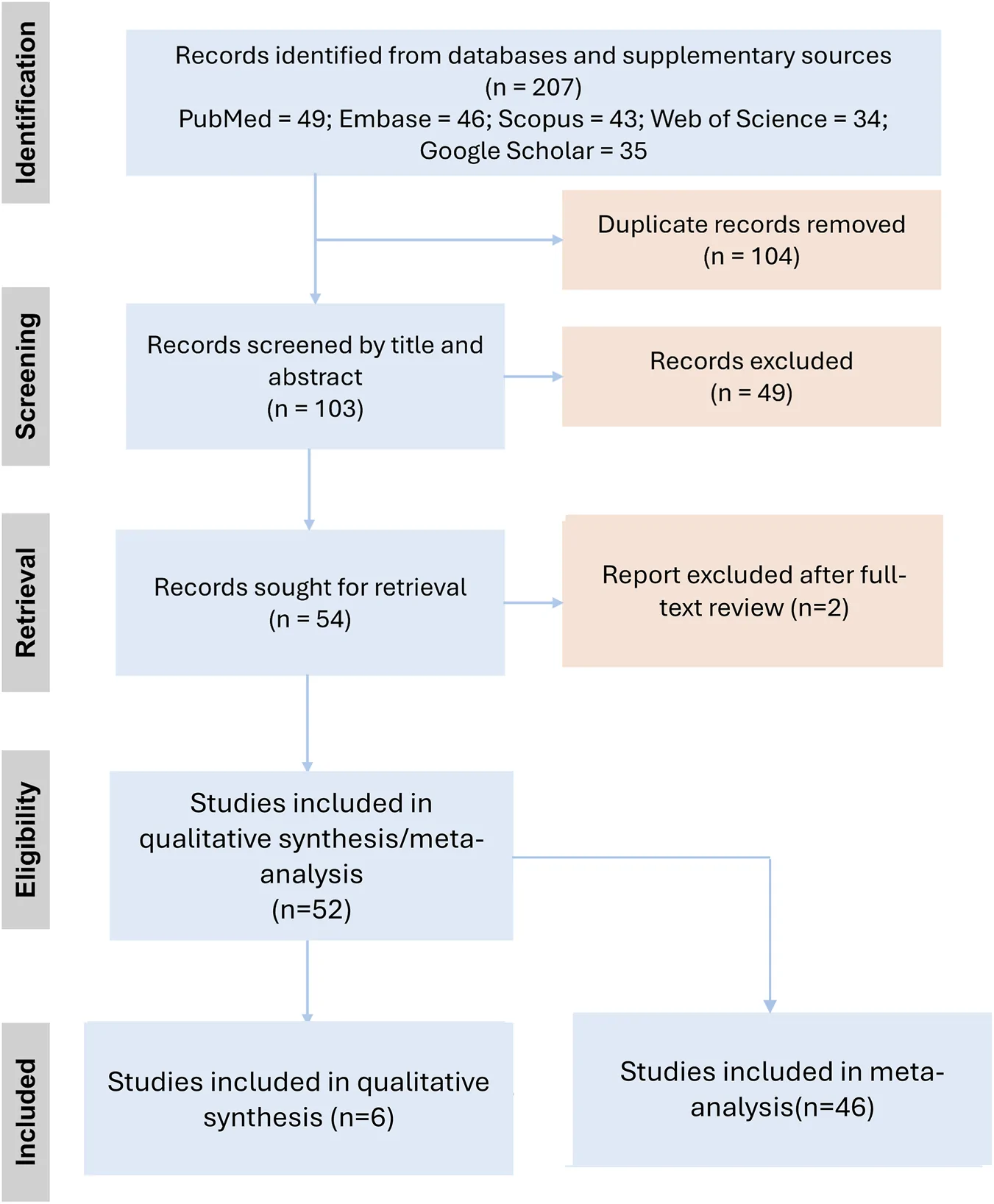
PRISMA 2020 flow diagram. Of 207 identified records, 54 full-text reports were assessed, 52 were retained in the systematic review, 6 remained in qualitative synthesis only because they lacked an independent analysis-ready frequency row, and 46 independent reports or datasets contributed to the primary meta-analysis.

The search identified 207 records: 49 from PubMed, 46 from Embase, 43 from Scopus, 34 from Web of Science, and 35 from Google Scholar or supplementary retrieval. After 104 duplicates were removed, 103 records were screened and 49 were excluded. All 54 reports sought for retrieval were obtained and assessed in full. Two clinically selected adverse-reaction reports lacked an eligible population-frequency denominator and were excluded. Fifty-two reports were retained in the systematic review; six did not provide an independent analysis-ready row and remained in the qualitative synthesis only. Forty-six independent reports or datasets contributed to the primary meta-analysis (Figure 1; Table 1; Supplementary Table S1).

**TABLE 1:** Evidence yield and analysis-ready variant sets in the Vietnamese pharmacogenetic meta-analysis.

Domain	Metric	Value	Interpretation
Study yield	Full-text reports assessed	54	All reports retrieved and evaluated for review eligibility
Study yield	Reports included in the systematic review	52	Two clinically selected adverse-reaction reports lacked an eligible population-frequency denominator
Study yield	Reports retained for qualitative synthesis only	6	No independent, analysis-ready frequency row; none contributed to a pooled estimate
Study yield	Independent reports/datasets in the primary meta-analysis	46	Non-overlapping quantitative evidence used for primary estimates
Variant coverage	Analysis-ready variant representations	108	Evidence set across 25 pharmacogenes; this count preserves clinically distinct representations
Variant coverage	Single-rsID variants eligible for 1,000 Genomes benchmarking	66	Restricted to interpretable, orientation-validated biallelic variants
Variant coverage	Complex or non-rsID representations	42	Star alleles, haplotypes, CNVs, structural variants, HLA alleles, and repeats
Quantitative evidence	Independent study-variant rows	173	Rows remaining after report linkage and overlap control
Quantitative evidence	Aggregate sample-size sum across rows	19,780	Not a count of unique individuals; participants tested for multiple variants recur across rows
Risk of bias	Low/moderate/high among 52 included reports	11/38/3	The three high-risk reports did not contribute an independent quantitative row
Risk of bias	Low/moderate/high among 46 quantitative reports	11/35/0	Most quantitative reports were at moderate risk
Clinical annotation	High/moderate clinical-priority representations	21/28	Counts across the full annotation set; Table 4 shows 20 nonredundant representatives

CNV, copy-number variant; rsID, Reference SNP cluster identifier.

Included reports were published from 2001 to 2025. Fifteen explicitly identified Kinh participants; most others used broader Vietnamese labels, and evidence for ethnic-minority groups was sparse. Twelve reports were based on population or healthy-volunteer samples, whereas the remainder drew mainly from clinical or mixed clinical-population settings, including cardiovascular disease, kidney transplantation, epilepsy, tuberculosis, gastroduodenal disease, gout, oncology, and newborn cohorts. Genotyping approaches ranged from PCR-RFLP and real-time PCR to pyrosequencing, Sanger sequencing, targeted next-generation sequencing, HLA typing, and copy-number assays.

Among the 52 included reports, 11 were classified at low, 38 at moderate, and 3 at high risk of bias. The three high-risk reports did not contribute an independent quantitative row. Among the 46 reports in the primary meta-analysis, 11 were at low and 35 at moderate risk. The most frequent limitations were clinically selected sampling, incomplete reporting of genotype counts or Hardy-Weinberg equilibrium, limited documentation of representativeness, and small denominators for individual variants (Supplementary Table S1).

### 3.2 Evidence coverage and representation

The analysis-ready evidence contained 173 study-variant rows with an aggregate row-level sample-size sum of 19,780; this sum is not a count of unique people because participants tested for multiple variants contribute to more than one row. The evidence was organized into 108 variant representations across 25 pharmacogenes. Sixty-six were orientation-validated single-rsID variants eligible in principle for direct 1,000 Genomes benchmarking, and 42 were star alleles, haplotypes, HLA alleles, repeats, copy-number changes, or other structural representations retained for clinical interpretation (Table 1; Supplementary Figures S1–S4; Supplementary Tables S1, S3).

The depth of evidence was highly uneven. Ninety-five of 108 representations had one contributing row and therefore received a Wilson interval rather than a pooled estimate. Only 13 representations had at least two independent rows; CYP2C19*2 and CYP2C19*3 were the most densely studied, with 19 and 20 rows, respectively. This distribution supports broad evidence mapping but limits the precision of national-frequency claims for many loci.

### 3.3 Vietnamese pharmacogenetic frequencies

**FIGURE 2:**
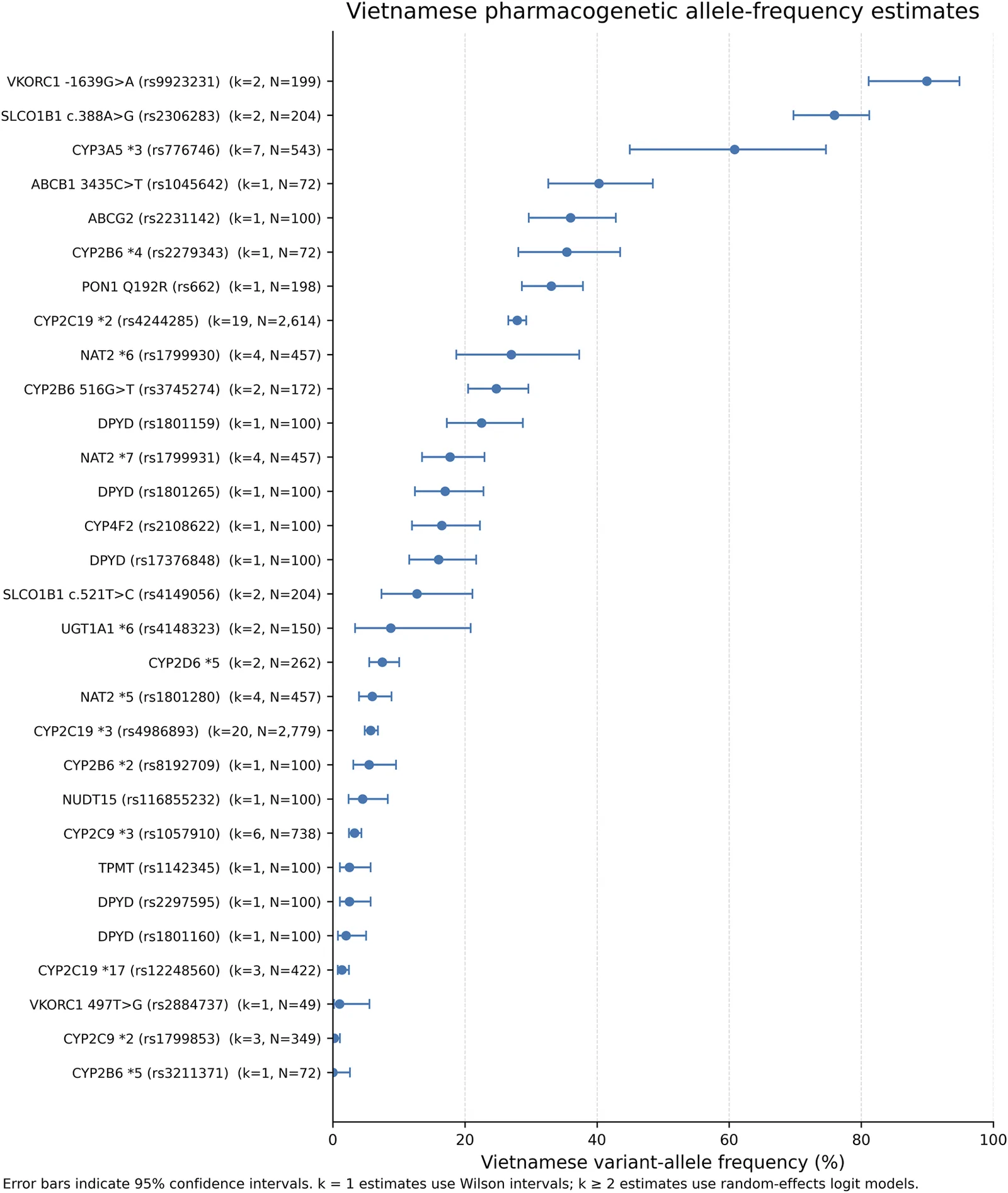
Vietnamese pharmacogenetic allele-frequency estimates. Points show pooled or single-study frequencies with 95% confidence intervals. Estimates with k = 1 use Wilson intervals; estimates with k ≥ 2 use random-effects logit models. Synonymous labels sharing the same rsID and effect-allele orientation were harmonized for *CYP2B6* rs3745274 and *SLCO1B1* rs2306283. k denotes contributing independent rows and N denotes total participants.

High-frequency findings included *VKORC1* -1639G>A at 89.91% (95% CI 81.13-94.86), *VKORC1* 1173C>T at 85.43% (81.61-88.56), *SLCO1B1* c.388A>G at 75.95% (69.75-81.22), CYP3A5*3 at 60.81% (44.96-74.67), *ABCB1* 3435C>T at 40.28% (32.62-48.44), and *ABCG2* c.421C>A at 36.00% (29.67-42.86) (Figure 2; Table 2; Supplementary Table S3). The estimates for *VKORC1* 1173C>T, *SLCO1B1* c.388A>G, and *CYP2B6* 516G>T incorporate study labels that mapped to the same rsID and allele orientation.

**TABLE 2:** Key Vietnamese pharmacogenetic allele-frequency estimates selected for main-text interpretation.

Gene	Allele/variant	rsID	Vietnamese frequency, % (95% CI)	k	Total N	I^2^, %	Main clinical relevance	Source Study ID(s)
*VKORC1*	−1639G>A	rs9923231	89.91 (81.13–94.86)	2	199	76.14	Warfarin dose sensitivity	S0043, S0066
*VKORC1*	1173C>T	rs9934438	85.43 (81.61–88.56)	2	199	0.00	Warfarin dose sensitivity	S0043, S0066
*SLCO1B1*	c.388A>G	rs2306283	75.95 (69.75–81.22)	2	204	47.42	Statin transport/haplotype marker	S0025, S0073
*CYP3A5*	*3	rs776746	60.81 (44.96–74.67)	7	543	95.54	Tacrolimus metabolism	S0008, S0014, S0033, S0036, S0044, S0060, S0089
*ABCB1*	3435C>T	rs1045642	40.28 (32.62–48.44)	1	72	—	Drug transport and exposure	S0014
*ABCG2*	c.421C>A/Q141K	rs2231142	36.00 (29.67–42.86)	1	100	—	Rosuvastatin exposure	S0025
*CYP2B6*	*4	rs2279343	35.42 (28.08–43.51)	1	72	—	CYP2B6 functional variation	S0014
*PON1*	Q192R	rs662	33.08 (28.63–37.86)	1	198	—	Clopidogrel response; evidence variable	S0098
*CYP2C19*	*2	rs4244285	27.90 (26.57–29.27)	19	2,614	11.80	Clopidogrel and proton-pump inhibitors	S0007, S0014, S0018, S0021, S0022, S0025, S0026, S0028, S0030, S0031, S0032, S0045, S0048, S0054, S0060, S0080, S0088, S0098, S0104
*NAT2*	*6	rs1799930	27.00 (18.69–37.31)	4	457	90.11	Isoniazid acetylation phenotype	S0009, S0025, S0067, S0092
*CYP2B6*	516G>T	rs3745274	24.76 (20.48–29.61)	2	172	0.00	Efavirenz metabolism	S0014, S0025
*DPYD*	c.1627A>G	rs1801159	22.50 (17.26–28.77)	1	100	—	Fluoropyrimidine relevance uncertain	S0025
*NAT2*	*7	rs1799931	17.75 (13.51–22.95)	4	457	71.71	Isoniazid acetylation phenotype	S0009, S0025, S0067, S0092
*SLCO1B1*	c.521T>C	rs4149056	12.73 (7.36–21.13)	2	204	75.83	Statin-associated musculoskeletal symptoms	S0025, S0073
*UGT1A1*	*6	rs4148323	8.76 (3.38–20.88)	2	150	76.61	Irinotecan toxicity	S0025, S0049
*UGT1A1*	*28	rs8175347	8.42 (5.25–13.24)	1	95	—	Irinotecan toxicity and bilirubin metabolism	S0087
*CYP2C19*	*3	rs4986893	5.74 (4.82–6.82)	20	2,779	54.69	Clopidogrel and proton-pump inhibitors	S0007, S0014, S0018, S0021, S0022, S0025, S0026, S0028, S0030, S0031, S0032, S0045, S0048, S0054, S0060, S0061, S0080, S0088, S0098, S0104
*CYP2C9*	*3	rs1057910	3.27 (2.46–4.32)	6	738	0.00	Warfarin, phenytoin, and NSAIDs	S0004, S0011, S0012, S0025, S0033, S0043
*TPMT*	c.719A>G	rs1142345	2.50 (1.07–5.72)	1	100	—	Thiopurine toxicity	S0025

CI, confidence interval; NSAID, nonsteroidal anti-inflammatory drug. I^2^ is not estimable for k = 1. Main-text estimates for CYP2B6 rs3745274, SLCO1B1 rs2306283, and VKORC1 rs9934438 collapse synonymous study labels with the same effect-allele orientation. Full row-level source information is provided in Supplementary Table S2.

*CYP2C19* loss-of-function alleles were common: CYP2C19*2 was 27.90% (26.57-29.27) and CYP2C19*3 was 5.74% (4.82-6.82). CYP2C9*3 was less frequent at 3.27% (2.46-4.32), and CYP2C9*2 was 0.22% (0.04-1.08). *CYP2B6* 516G>T was 24.76% (20.48-29.61). NAT2*6 and NAT2*7 were 27.00% (18.69-37.31) and 17.75% (13.51-22.95), respectively. UGT1A1*6 and UGT1A1*28 were 8.76% (3.38-20.88) and 8.42% (5.25-13.24), while *SLCO1B1* c.521T>C was 12.73% (7.36-21.13).

Clinically meaningful complex representations were not interchangeable with single-marker proxies. The CYP2D6*10 star-allele estimate was 43.75% (37.98-49.69), whereas rs1065852 alone yielded 45.07% in a different denominator. CYP2D6*5, defined by whole-gene deletion, was 7.47% (5.51-10.06), and CYP2D6*36 was 0.37% (0.06-2.05). These estimates illustrate why an rsID-only evidence model can misstate both assay requirements and phenotype assignment.

### 3.4 Heterogeneity and sensitivity analyses

Heterogeneity varied substantially by locus. CYP3A5*3 showed I^2^ = 95.54%, NAT2*6 I^2^ = 90.11%, UGT1A1*6 I^2^ = 76.61%, *SLCO1B1* c.521T>C I^2^ = 75.83%, and *VKORC1* -1639G>A I^2^ = 76.14%. In contrast, CYP2C9*3 showed no detectable heterogeneity across six rows and CYP2C19*2 showed low heterogeneity despite 19 contributing rows. The high-I^2^ estimates often reflected differences in clinical setting, ethnicity labels, genotyping method, or small denominators rather than a single uniform national prevalence.

Predefined sensitivity analyses were directionally consistent for most variants, but CYP3A5*3 and NAT2*6 shifted by more than 5 percentage points under selected restrictions. The healthy or control estimate for CYP2C19*2 was 22.94%, 4.96 points below the primary estimate of 27.90%. These changes do not alter the existence of established gene-drug guidance, but they materially affect projected test yield, budget impact, and the precision of health-economic models (Supplementary Tables S4–S6).

### 3.5 Global comparison and population similarity

**FIGURE 3:**
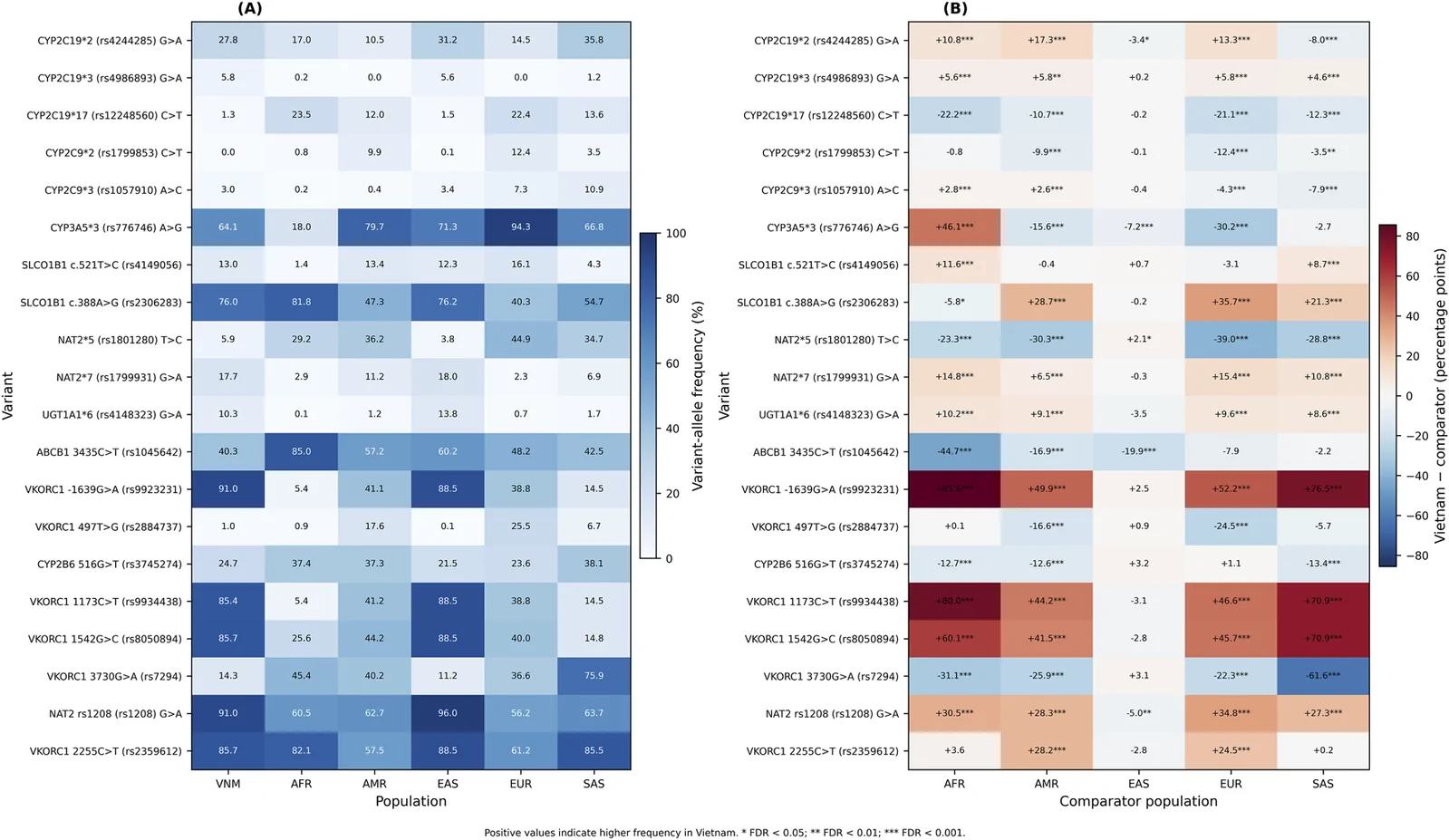
Shared pharmacogenetic allele frequencies and cross-population differences. Panel **(A)** shows allele-count frequencies for 20 variants with harmonized effect-allele orientation in Vietnam and the 1,000 Genomes African (AFR), admixed American (AMR), East Asian (EAS), European (EUR), and South Asian (SAS) superpopulations. Panel **(B)** shows Vietnamese-minus-comparator differences from the same matrix. Positive values indicate a higher frequency in Vietnam. *, **, and *** denote FDR-adjusted p < 0.05, p < 0.01, and p < 0.001, respectively. Vietnamese allele-count frequencies may differ slightly from random-effects pooled estimates in Table 2.

The principal global comparison included 20 single-rsID variants with validated effect-allele orientation (Figure 3; Table 3). Eighteen differed from EAS by no more than 5 percentage points. The largest EAS differences were *ABCB1* 3435C>T, 40.3% in Vietnam versus 60.2% in EAS (−19.9 points), and CYP3A5*3, 64.1% versus 71.3% (−7.2 points). Smaller EAS differences reached false-discovery-rate significance for CYP2C19*2 (−3.4 points), NAT2*5 (+2.1 points), and *NAT2* rs1208 (−5.0 points), demonstrating that statistical significance driven by large reference allele counts need not indicate a large implementation difference.

**TABLE 3:** Orientation-harmonized allele-count frequencies in Vietnam and 1,000 Genomes superpopulations.

Gene/variant	rsID	VNM, %	AFR, %	AMR, %	EAS, %	EUR, %	SAS, %	FDR-significant comparator(s)
CYP2C19*2	rs4244285	27.8	17.0	10.5	31.2	14.5	35.8	AFR, AMR, EAS, EUR, SAS
CYP2C19*3	rs4986893	5.8	0.2	0.0	5.6	0.0	1.2	AFR, AMR, EUR, SAS
CYP2C19*17	rs12248560	1.3	23.5	12.0	1.5	22.4	13.6	AFR, AMR, EUR, SAS
CYP2C9*2	rs1799853	0.0	0.8	9.9	0.1	12.4	3.5	AMR, EUR, SAS
CYP2C9*3	rs1057910	3.0	0.2	0.4	3.4	7.3	10.9	AFR, AMR, EUR, SAS
CYP3A5*3	rs776746	64.1	18.0	79.7	71.3	94.3	66.8	AFR, AMR, EAS, EUR
*SLCO1B1 c.521T>C*	rs4149056	13.0	1.4	13.4	12.3	16.1	4.3	AFR, SAS
*SLCO1B1 c.388A>G*	rs2306283	76.0	81.8	47.3	76.2	40.3	54.7	AFR, AMR, EUR, SAS
NAT2*5	rs1801280	5.9	29.2	36.2	3.8	44.9	34.7	AFR, AMR, EAS, EUR, SAS
NAT2*7	rs1799931	17.7	2.9	11.2	18.0	2.3	6.9	AFR, AMR, EUR, SAS
UGT1A1*6	rs4148323	10.3	0.1	1.2	13.8	0.7	1.7	AFR, AMR, EUR, SAS
*ABCB1 3435C>T*	rs1045642	40.3	85.0	57.2	60.2	48.2	42.5	AFR, AMR, EAS
*VKORC1 -1639G>A*	rs9923231	91.0	5.4	41.1	88.5	38.8	14.5	AFR, AMR, EUR, SAS
*VKORC1 497T>G*	rs2884737	1.0	0.9	17.6	0.1	25.5	6.7	AMR, EUR
*CYP2B6 516G>T*	rs3745274	24.7	37.4	37.3	21.5	23.6	38.1	AFR, AMR, SAS
*VKORC1 1173C>T*	rs9934438	85.4	5.4	41.2	88.5	38.8	14.5	AFR, AMR, EUR, SAS
*VKORC1 1542G>C*	rs8050894	85.7	25.6	44.2	88.5	40.0	14.8	AFR, AMR, EUR, SAS
*VKORC1 3730G>A*	rs7294	14.3	45.4	40.2	11.2	36.6	75.9	AFR, AMR, EUR, SAS
*NAT2 rs1208*	rs1208	91.0	60.5	62.7	96.0	56.2	63.7	AFR, AMR, EAS, EUR, SAS
*VKORC1 2255C>T*	rs2359612	85.7	82.1	57.5	88.5	61.2	85.5	AMR, EUR

AFR, African; AMR, admixed American; EAS, East Asian; EUR, European; FDR, false discovery rate; SAS, South Asian; VNM, Vietnamese. Frequencies are allele-count estimates used for pairwise testing and may differ slightly from pooled estimates in Table 2. Full odds ratios, confidence intervals, and adjusted p values are provided in Supplementary Table S9. The final column identifies the comparator populations for which the Benjamini-Hochberg-adjusted p value is below 0.05; it is not a count.

The *VKORC1* rs2359612 effect-allele orientation was independently checked before final analysis. The T-allele frequency was 85.7% in Vietnam and 88.5% in EAS, an absolute difference of −2.8 points that was not significant after adjustment. Full odds ratios, confidence intervals, adjusted p values, and the orientation audit are provided in Supplementary Table S9. Vietnamese allele-count frequencies in the global comparison can differ modestly from random-effects pooled estimates because the former use non-overlapping aggregate allele counts. The complete all-available frequency heatmaps, Vietnamese-minus-comparator difference heatmaps, and ranked complete-case differences are provided in Supplementary Figures S2–S4.

**FIGURE 4:**
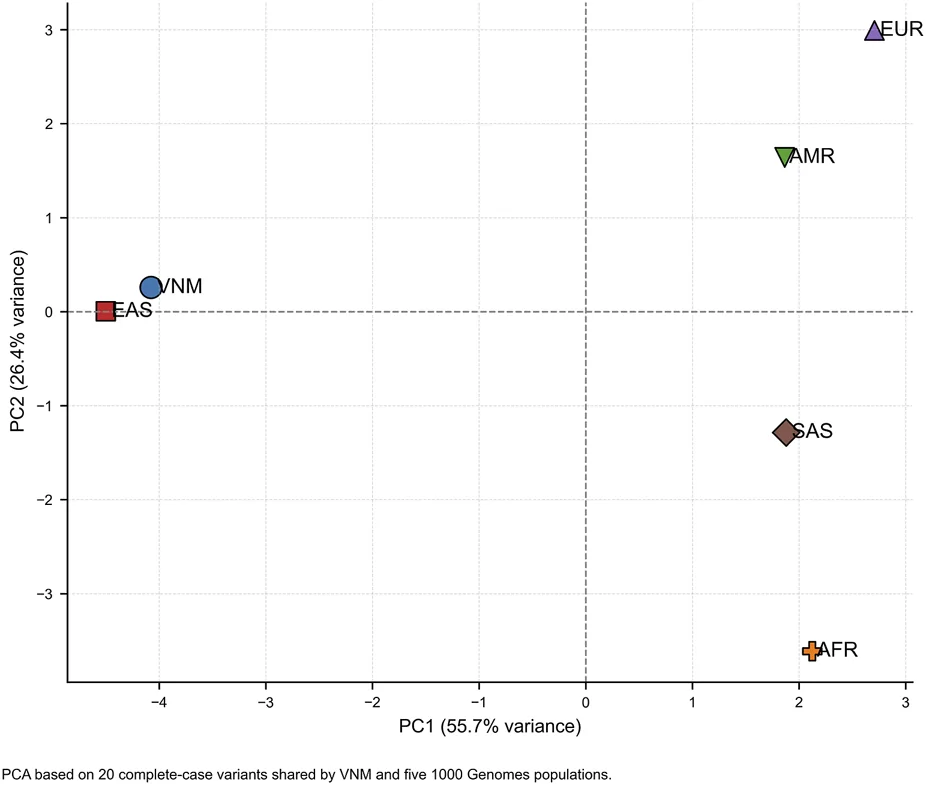
Population similarity based on shared pharmacogenetic frequencies. Principal component analysis used 20 standardized complete-case variants. PC1 and PC2 explain 55.7% and 26.4% of variance. The plot compares aggregate population-frequency profiles and is not an ancestry classifier; the EAS superpopulation includes KHV.

Principal component analysis of the 20 complete-case variants placed Vietnam nearest to EAS; PC1 and PC2 explained 55.7% and 26.4% of the variance, respectively (Figure 4). This result describes similarity in a selected pharmacogenetic frequency matrix. It should not be interpreted as an ancestry classifier, and the inclusion of KHV within the 1,000 Genomes EAS group makes the EAS comparison partly non-independent.

### 3.6 Clinical actionability

**FIGURE 5:**
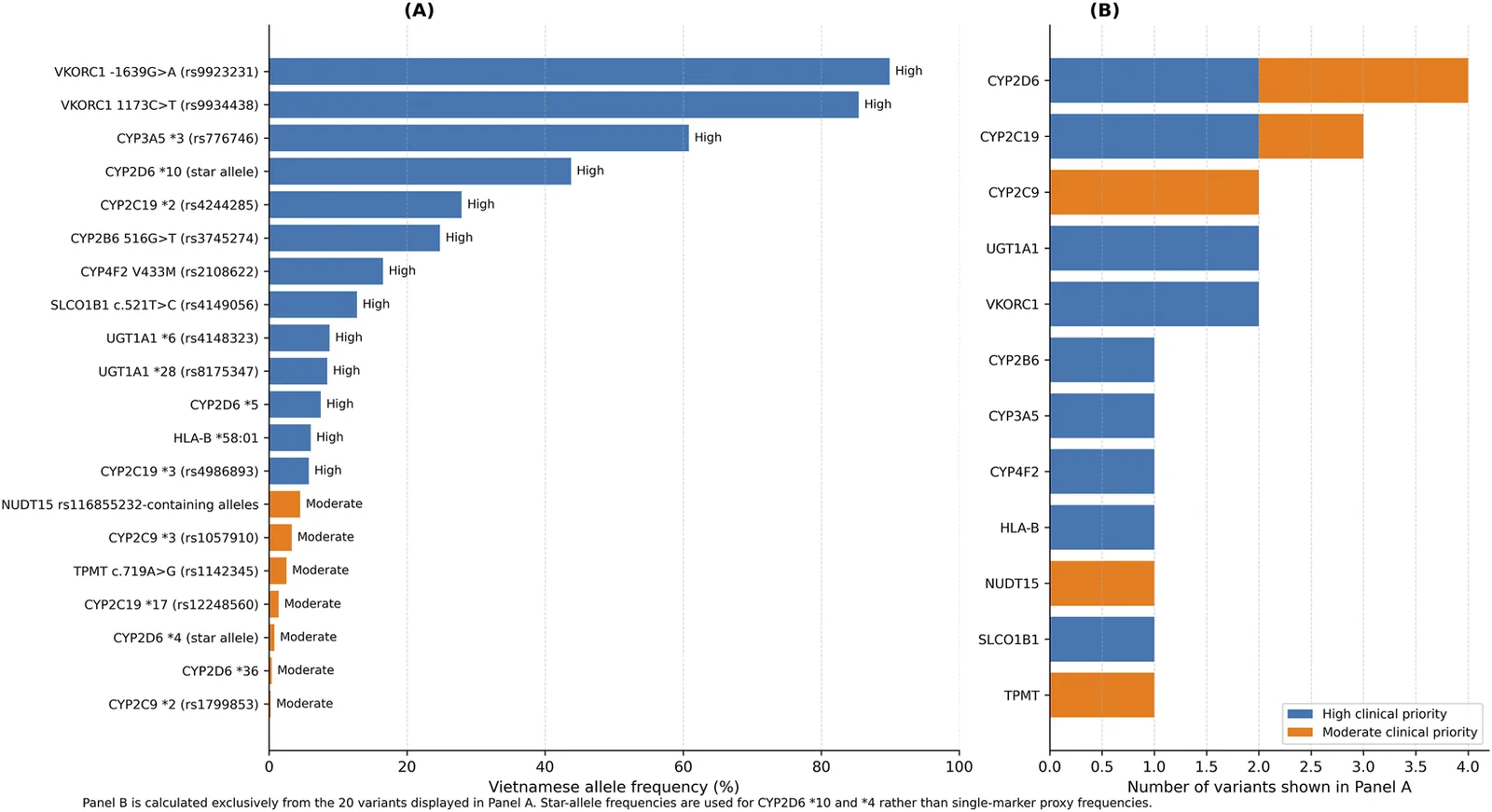
Clinically prioritized pharmacogenetic variation in Vietnam. Panel **(A)** ranks 20 nonredundant primary representatives by Vietnamese frequency and implementation priority. Panel **(B)** is calculated exclusively from Panel A and summarizes the genes and priority categories represented there. CYP2D6*10 and *4 use star-allele estimates rather than single-marker proxy frequencies. High and moderate priorities correspond to Table 4.

Across the full annotation set, 21 representations were assigned high and 28 moderate implementation priority. Table 4 and Figure 5 show 20 nonredundant primary representatives: 13 high-priority and 7 moderate-priority entries. The high-priority group includes common markers with substantial expected yield - such as *VKORC1*, *CYP2C19*, *CYP3A5*, *CYP2B6*, and *SLCO1B1* - as well as lower-frequency markers linked to severe preventable toxicity, including HLA-B*58:01 and *UGT1A1* variants. *NUDT15* and *TPMT* illustrate why clinical consequence can outweigh population frequency.

**TABLE 4:** Clinically actionable or potentially actionable pharmacogenetic variants prioritized for Vietnamese implementation.

Gene	Allele/variant	rsID	Functional effect	Key drug(s)	Guideline/evidence source	Vietnamese frequency, % (95% CI)	Implementation priority
*VKORC1*	−1639G>A	rs9923231	Reduced VKORC1 expression	Warfarin	CPIC warfarin guideline (Johnson et al., 2017)	89.91 (81.13–94.86)	High: common warfarin-sensitivity marker; use within a validated algorithm
*VKORC1*	1173C>T	rs9934438	Warfarin-sensitivity haplotype marker	Warfarin	CPIC warfarin guideline (Johnson et al., 2017)	85.43 (81.61–88.56)	High: common sensitivity marker; not an independent dose rule
*CYP3A5*	*3	rs776746	No function; non-expresser allele	Tacrolimus	CPIC tacrolimus guideline (Birdwell et al., 2015)	60.81 (44.96–74.67)	High: major determinant of starting-dose requirement; TDM remains required
*CYP2D6*	*10	- (haplotype; rs1065852 alone is insufficient)	Decreased function	Codeine; tramadol; selected antidepressants; antipsychotics; tamoxifen	CPIC/DPWG; refs 30, 31, 38–41, 55	43.75 (37.98–49.69)	High: common reduced-function star allele; copy-number and structural assessment are required for phenotype assignment
*CYP2C19*	*2	rs4244285	No function	Clopidogrel; PPIs; selected antidepressants	CPIC/DPWG; refs 28–30	27.90 (26.57–29.27)	High: common loss-of-function allele
*CYP2B6*	516G>T	rs3745274	Decreased function	Efavirenz; other substrates require drug-specific evidence	CPIC efavirenz guideline (Desta et al., 2019)	24.76 (20.48–29.61)	High: common functional variant; guideline action is drug-specific
*CYP4F2*	V433M	rs2108622	Warfarin dose modifier	Warfarin	CPIC warfarin guideline (Johnson et al., 2017)	16.50 (12.00–22.27)	High within a multigene warfarin panel; incremental effect is modest
*SLCO1B1*	c.521T>C	rs4149056	Decreased transporter function	Simvastatin; atorvastatin; rosuvastatin	CPIC statin guideline (Cooper-DeHoff et al., 2022)	12.73 (7.36–21.13)	High: statin-associated musculoskeletal-symptom risk
*UGT1A1*	*6	rs4148323	Decreased function	Irinotecan; atazanavir	DPWG and primary evidence (Hulshof et al., 2023; Innocenti et al., 2004)	8.76 (3.38–20.88)	High: Asian-relevant irinotecan-toxicity allele
*UGT1A1*	*28	rs8175347	Decreased expression	Irinotecan; atazanavir	DPWG and primary evidence (Hulshof et al., 2023; Innocenti et al., 2004)	8.42 (5.25–13.24)	High: irinotecan toxicity and hyperbilirubinemia risk
*CYP2D6*	*5	—	No function; whole-gene deletion	CYP2D6 substrates	CPIC/DPWG; refs 30, 31, 38–41	7.47 (5.51–10.06)	High: copy-number-defined no-function allele
*HLA-B*	*58:01	—	Risk allele	Allopurinol	CPIC allopurinol guideline (Hershfield et al., 2013; Saito et al., 2016)	6.02 (3.74–9.55)	High: severe cutaneous adverse-reaction risk
*CYP2C19*	*3	rs4986893	No function	Clopidogrel; PPIs; selected antidepressants	CPIC/DPWG; refs 28–30	5.74 (4.82–6.82)	High: Asian-relevant loss-of-function allele
*NUDT15*	rs116855232-containing alleles	rs116855232	No or decreased function	Azathioprine; mercaptopurine; thioguanine	CPIC thiopurine guideline (Relling et al., 2019)	4.50 (2.39–8.33)	Moderate: lower frequency but severe preventable myelotoxicity
*CYP2C9*	*3	rs1057910	Decreased function	Warfarin; phenytoin; NSAIDs; siponimod	CPIC/DPWG; refs 25, 32, 56, 57	3.27 (2.46–4.32)	Moderate: clinically actionable, but uncommon
*TPMT*	c.719A>G	rs1142345	No or decreased function	Azathioprine; mercaptopurine; thioguanine	CPIC thiopurine guideline (Relling et al., 2019)	2.50 (1.07–5.72)	Moderate: lower frequency but severe preventable myelotoxicity
*CYP2D6*	*4	- (star allele; rs3892097 is a defining marker)	No function	CYP2D6 substrates	CPIC/DPWG; refs 30, 31, 38–41, 55	0.74 (0.20–2.64)	Moderate: uncommon but phenotype-defining; star-allele and structural context are required
*CYP2C19*	*17	rs12248560	Increased function	PPIs; selected antidepressants; phenotype assignment	CPIC/DPWG; refs 28–30	1.37 (0.76–2.46)	Moderate: uncommon but relevant to diplotype-derived phenotype
*CYP2D6*	*36	—	Hybrid/structurally complex allele	CYP2D6 substrates	PharmVar/CPIC context; refs 10, 38–41	0.37 (0.06–2.05)	Moderate: requires a structural assay and cautious diplotype interpretation
*CYP2C9*	*2	rs1799853	Decreased function	Warfarin; phenytoin; NSAIDs; siponimod	CPIC/DPWG; refs 25, 32, 56, 57	0.22 (0.04–1.08)	Moderate: rare but phenotype-defining

CI, confidence interval; CPIC, clinical pharmacogenetics implementation consortium; DPWG, dutch pharmacogenetics working group; NSAID, nonsteroidal anti-inflammatory drug; PPI, proton-pump inhibitor; TDM, therapeutic drug monitoring. A listed single variant is not necessarily sufficient for phenotype assignment. CYP2D6 implementation requires star-allele, copy-number, and structural-variant assessment; rs1065852 and rs3892097 are not used as stand-alone proxies for CYP2D6*10 and *4.

The ranking also exposed assay-dependent priorities. CYP2D6*10, *5, *4, and *36 cannot be interpreted responsibly through an undifferentiated small-SNP panel; star-allele calling, copy-number assessment, structural-variant detection, and standardized phenotype translation are required. Conversely, the large Vietnam-EAS difference for *ABCB1* 3435C>T does not itself create a guideline-supported prescribing action. Frequency, statistical difference, functional evidence, and therapeutic guidance therefore remained separate dimensions of interpretation.

## 4 Discussion

### 4.1 Principal findings and contribution

This review provides a report-level synthesis of Vietnamese pharmacogenomic evidence that is broader than a single-gene meta-analysis and more clinically structured than a catalogue of rsIDs. The dominant population signal is regional similarity to East Asia, but the clinically important message is conditionality: similarity is strong for many loci, incomplete for others, and least informative when the relevant unit is a haplotype, HLA allele, copy-number state, or hybrid structure. The synthesis also reveals a marked imbalance between breadth and replication. Although 25 pharmacogenes and 108 representations were captured, 88% of representations were supported by one row. The current evidence can therefore guide prioritization, but it does not yet constitute a uniformly precise national reference panel.

The work is complementary to, rather than a replacement for, Southeast Asian sequencing consortia and large population-genetic resources. SEAPharm and GenomeAsia provide standardized, high-throughput snapshots across many genes and populations; the present review adds bilingual local retrieval, study-level denominators, clinical-context metadata, risk-of-bias assessment, duplicate control, sensitivity analyses, and preservation of complex clinical nomenclature (Runcharoen et al., 2021; GenomeAsia100K Consortium, 2019). These layers answer different questions. Population sequencing is strongest for discovery and internally consistent comparison, whereas systematic review is strongest for exposing how published evidence was generated, where it disagrees, and which estimates are robust enough to support implementation planning.

### 4.2 East Asian proximity does not imply interchangeability

The global analysis supports EAS as a useful regional prior, not as a substitute for Vietnamese data. Eighteen of 20 principal variants were within 5 percentage points of EAS, and the PCA placed Vietnam close to EAS. Yet *ABCB1* 3435C>T and CYP3A5*3 showed materially larger differences, and smaller differences were statistically significant at several loci. Health-system decisions should therefore consider absolute differences and confidence intervals, not adjusted p values alone. A 2- or 3-point difference may be statistically convincing but operationally modest; a 20-point difference can substantially alter expected test yield even when it does not change the prescribing guideline itself.

The comparator also requires a methodological caveat. KHV is one of the component populations of 1,000 Genomes EAS, so the EAS benchmark is not fully independent of Vietnamese ancestry. This overlap will tend to attenuate, rather than exaggerate, some Vietnam-EAS differences. Future analyses should report KHV separately and construct EAS-without-KHV sensitivity comparisons when allele counts are available. More broadly, biogeographic labels are pragmatic tools for describing group-level frequency patterns; they are not biological prescriptions for treating an individual (Huddart et al., 2019).

### 4.3 Gene-drug priorities for Vietnam

Warfarin illustrates the need to combine high-frequency local evidence with multivariable clinical prediction. *VKORC1* -1639G>A and linked sensitivity markers were highly prevalent, whereas CYP2C9*2 was rare and CYP2C9*3 remained uncommon but actionable. This pattern is consistent with a population in which *VKORC1* contributes strongly to dose sensitivity while common European *CYP2C9* assumptions may be less informative. Nonetheless, no single frequency should be translated into a dose. Validated algorithms integrate *VKORC1*, *CYP2C9*, *CYP4F2* where appropriate, age, body size, interacting medicines, indication, and other clinical covariates (Johnson et al., 2017; Klein et al., 2009; Gage et al., 2008). Vietnamese implementation research should test calibration and clinical utility of an algorithm in local patients rather than merely reproduce genotype-frequency differences.

*CYP2C19* is the most immediate high-yield multitherapeutic target. Approximately 28% of alleles were CYP2C19*2% and 6% were CYP2C19*3, while increased-function CYP2C19*17 was uncommon. These frequencies imply a substantial burden of intermediate and poor metabolizer diplotypes, but phenotype prevalence cannot be inferred by summing allele frequencies without phased diplotype assumptions. For clopidogrel, loss-of-function phenotypes can support an alternative P2Y12 inhibitor when clinically appropriate; for proton-pump inhibitors, reduced metabolism generally increases exposure; and for selected antidepressants, *CYP2C19* must be interpreted alongside drug-specific guidance and *CYP2D6* (Lee et al., 2022; Lima et al., 2021; Bousman et al., 2023). Reporting should therefore translate a complete tested diplotype into a phenotype and medication-specific recommendation, rather than return isolated variant calls.

For tacrolimus, CYP3A5*3 was common but highly heterogeneous across studies. *CYP3A5* expressers generally require a higher genotype-informed starting dose to approach target exposure, yet therapeutic drug monitoring remains indispensable because time after transplantation, corticosteroids, interacting drugs, adherence, hematocrit, organ function, and assay factors contribute to variability (Birdwell et al., 2015). The pooled frequency is useful for estimating how often a genotype-guided starting dose may be relevant; it should not be treated as a fixed national prevalence or a replacement for center-specific validation.

Transporter findings require especially disciplined interpretation. *SLCO1B1* c.521T>C has guideline-supported relevance to statin-associated musculoskeletal symptoms, and *ABCG2* c.421C>A contributes to rosuvastatin exposure and prescribing guidance in combination with other factors (Cooper-DeHoff et al., 2022). By contrast, the more frequent *SLCO1B1* c.388A>G is best interpreted within haplotype context, and *ABCB1* 3435C>T - despite the largest Vietnam-EAS difference - does not have a comparably mature, generalizable prescribing action. A population difference is a hypothesis for pharmacokinetic or outcomes research, not sufficient evidence for a clinical rule.

The oncology and severe-toxicity loci argue for a second implementation logic: low or moderate frequency can still justify testing when harm is serious and preventable. UGT1A1*6 is particularly relevant in Asian populations and should not be omitted from an irinotecan assay focused only on UGT1A1*28. *NUDT15* should be evaluated with *TPMT* before thiopurines because myelotoxicity can be profound, and HLA-B*58:01 provides a clear opportunity to prevent allopurinol-associated severe cutaneous adverse reactions (Hulshof et al., 2023; Innocenti et al., 2004; Hershfield et al., 2013; Relling et al., 2019; Saito et al., 2016). These relationships are better prioritized by the combination of severity, alternative therapy, turnaround time, and treatment exposure than by allele frequency alone.

*CYP2B6* 516G>T was common after harmonization of two study labels. Its clearest guideline-supported implementation concerns efavirenz, for which reduced-function genotypes can increase exposure and central nervous system toxicity (Desta et al., 2019). Extrapolation to nevirapine, bupropion, or methadone should remain drug-specific because the evidence and recommendations are not interchangeable. This example reinforces the need for tables and electronic decision support to name the precise gene-drug pair, not simply label a variant 'actionable.'

### 4.4 Assay design, phenotype translation, and clinical workflow

*CYP2D6* is the most prominent warning against designing a national panel from a list of high-frequency SNPs. The Vietnamese evidence includes CYP2D6*10, whole-gene deletion, tandem arrangements, and hybrid alleles. A small panel that detects rs1065852 but cannot determine copy number, phase, or hybrid structure can misclassify diplotypes and activity scores. A fit-for-purpose assay should declare which star alleles and structural configurations are detectable, validate copy-number performance, apply standardized genotype-to-phenotype translation, and report residual uncertainty (Gaedigk et al., 2018; Nguyen et al., 2019; Gaedi and gk, 2013; Gaedigk et al., 2017; Ingelm et al., 2018; Lauschke and Ingelman-Sundberg, 2020; Caudle et al., 2020). Clinical systems must also account for phenoconversion when strong *CYP2D6* inhibitors make the observed metabolic phenotype differ from the genotype-predicted phenotype.

A staged Vietnamese implementation model is therefore preferable to a uniform panel launched without supporting infrastructure. A first tier could prioritize mature, high-use pathways such as *CYP2C19*-clopidogrel and proton-pump inhibitors, *VKORC1*/*CYP2C9*/*CYP4F2*-warfarin, *CYP3A5*-tacrolimus, and *SLCO1B1*/*ABCG2*-statins. A parallel safety tier could target HLA-B*58:01-allopurinol, *NUDT15*/*TPMT*-thiopurines, and *UGT1A1*-irinotecan in settings where treatment exposure and turnaround time justify testing. *CYP2D6* and other structurally complex loci should be deployed only with validated assays, informatics, and proficiency testing. The distinction is not one of clinical importance; it is one of laboratory readiness.

Implementation also requires infrastructure that a frequency meta-analysis cannot supply. Laboratories need reference materials, allele-definition version control, external quality assurance, and explicit test limitations. Electronic health records must store genotype, diplotype, phenotype, and the versioned interpretation so that a result can be reused across a patient’s lifetime. Decision support should trigger only for the relevant medicine and clinical context, display an actionable recommendation rather than a generic risk label, and remain responsive to guideline updates. Pharmacists, prescribers, laboratory specialists, genetic professionals, and patients all require role-specific education.

The PREPARE trial establishes that panel-guided prescribing can reduce adverse reactions in a European implementation network, but transferability to Vietnam is not automatic (Swen et al., 2023). Local medication-use patterns determine how often a result is encountered; reimbursement and laboratory capacity determine access; and clinical pathways determine whether a result changes treatment. Prospective Vietnamese evaluations should therefore measure prescribing changes, time to target concentration, adverse reactions, therapeutic failure, turnaround time, clinician acceptance, equity of access, and cost-effectiveness. These endpoints are more informative for policy than allele frequency alone.

### 4.5 Strengths and limitations

The principal strengths are prospective registration, bilingual retrieval, source-level traceability, explicit report linkage, effect-allele orientation auditing, sensitivity analysis, and separation of single-rsID benchmarking from complex-allele interpretation. Harmonizing synonymous labels at the rsID and allele-direction level corrected three main-text estimates without erasing their source rows. The review also distinguishes descriptive population similarity from clinical actionability and avoids treating a statistically different allele as automatically actionable.

Several limitations constrain inference. First, most reports involved Kinh participants, broad 'Vietnamese' labels, or clinically selected samples; ethnic-minority evidence was insufficient for representative subgroup estimates. Second, 95 of 108 representations had one row, and several pooled estimates had high heterogeneity or wide confidence intervals. Third, genotype counts, Hardy-Weinberg testing, recruitment geography, and assay quality were inconsistently reported. Fourth, aggregate data prevented individual-level adjustment for age, sex, disease, region, or relatedness, and the row-level sample-size sum overcounts people tested for multiple variants. Fifth, DerSimonian-Laird models can underestimate uncertainty when few studies contribute, so small-k estimates should be interpreted alongside sensitivity analyses and raw study ranges. Sixth, the global comparison was limited to single-rsID variants and used an EAS reference containing KHV. Seventh, the implementation-priority categories were expert-informed and not prospectively validated. Finally, international guidelines were used to interpret clinical relevance because Vietnamese outcome and economic studies remain limited.

The protocol deviations were reported transparently, but they remain a consideration. Retaining sub-threshold or non-independent reports in the qualitative map broadened contextual coverage without changing pooled estimates; omitting funnel tests and GRADE avoided methods that would have been poorly matched to predominantly single-study frequency outcomes. Independent replication, public release of analysis code, and a fully versioned Vietnamese pharmacogenomic knowledgebase would further strengthen reproducibility.

### 4.6 Research agenda

The next phase should move from opportunistic publication to a coordinated national evidence program. Population sampling should deliberately include major geographic regions and ethnic groups, with transparent recruitment frames and sufficient denominators for rare but consequential alleles. Sequencing and long-read or orthogonal structural assays should be used where short-variant methods are inadequate. Studies should report raw genotype counts, Hardy-Weinberg results, allele-definition versions, quality-control metrics, and phenotype translation rules. A national repository can then connect population frequencies to medication exposure, outcomes, and implementation cost while preserving governance, privacy, and community engagement.

Such a program would allow Vietnam to replace two problematic extremes: uncritical extrapolation from international averages and premature claims of a single national pharmacogenomic profile. The current evidence supports a more precise position. Vietnamese populations share many pharmacogenetic features with East Asian references, selected loci differ enough to affect implementation planning, and clinically meaningful variation extends beyond SNPs. The most defensible path is genotype-based prescribing supported by local evidence, transparent assay capability, and prospective outcome evaluation.

## 5 Conclusion

Vietnamese pharmacogenomic evidence is broadly East Asian-adjacent but variant-specific, method-dependent, and unevenly replicated. The strongest implementation case combines frequent, guideline-supported pathways with tests that prevent severe toxicity, while reserving structurally complex genes for laboratories capable of complete and transparent interpretation. Local frequency data should guide panel design and health-system planning, not substitute for individual genotyping or clinical judgment. Representative multiethnic sampling, standardized reporting, validated assays, and prospective Vietnamese outcome and economic studies are now the critical steps toward equitable pharmacogenomic medicine.

## Data Availability

The original contributions presented in the study are included in the article/Supplementary Material, further inquiries can be directed to the corresponding author.
