# Supplementary material for: Vietnamese pharmacogenomic variation in global context: a systematic review and meta-analysis for clinical implementation
Source: Front Pharmacol. 2026 Sep 3;17:1921144. doi: 10.3389/fphar.2026.1921144 (PMC13582518; doi:10.3389/fphar.2026.1921144)
Supplement: Supplementary file 1 [file Table1.docx]

**Supplementary Table S1. Characteristics, population context, methods, complete bibliographic citations, analytic status, and total risk-of-bias score for the 54 full-text reports assessed**

Study flow: 54 full-text reports were assessed; 52 were retained in the systematic review (46 contributed to the primary quantitative synthesis and 6 to qualitative synthesis only), and 2 clinically selected adverse-reaction reports were excluded because they lacked an eligible unselected population-frequency denominator.

Risk of bias was assessed across six genetic-epidemiology domains. Scores of 5–6, 3–4, and 0–2 denote low, moderate, and high risk, respectively. Among the 52 included reports, 11 were low risk, 38 moderate risk, and 3 high risk; among the 46 quantitative reports, 11 were low risk, 35 moderate risk, and none high risk. The two excluded reports are shown only for transparent full-text accounting and are not included in these risk-of-bias summaries.

| **Study ID** | **First author, year** | **Article/report title** | **Source type** | **Population/ethnicity** | **Study context** | **Vietnamese participants, n** | **Genotyping method** | **Gene(s)** | **Contribution to synthesis** | **Analytic status/reason** | **Reference no.** | **Risk-of-bias score** |
| --- | --- | --- | --- | --- | --- | --- | --- | --- | --- | --- | --- | --- |
| S0004 | Sang Seop Lee et al., 2005 | Genetic Polymorphism of CYP2C9 in a Vietnamese Kinh Population | Journal article | Kinh; Vietnamese | Population/healthy-volunteer sample | 157 | PCR; pyrosequencing | CYP2C9 | Primary meta-analysis | Included in the systematic review and primary quantitative synthesis. | 1 | 5/6 (Low) |
| S0006 | Chang-Woo Yeo et al., 2011 | Discovery of a Novel Allelic Variant of CYP2C8, CYP2C8*11, in Asian Populations and Its Clinical Effect on Rosiglitazone Disposition In Vivo | Journal article | African American; Han Chinese; Korean; Vietnamese; Viet Kinh; White | Population/healthy-volunteer sample | 447 | pyrosequencing | CYP2C8 | Primary meta-analysis | Included in the systematic review and primary quantitative synthesis. | 2 | 5/6 (Low) |
| S0007 | Sirikan Yamada et al., 2001 | Genetic Differences in CYP2C19 Single Nucleotide Polymorphisms among Four Asian Populations | Journal article | Chinese; Japanese; Thai; Vietnamese | Population/healthy-volunteer sample | 121 | PCR-RFLP | CYP2C19 | Primary meta-analysis | Included in the systematic review and primary quantitative synthesis. | 3 | 5/6 (Low) |
| S0008 | Nguyen Thanh Xuan et al., 2022 | Frequencies and Association of CYP3A5 Polymorphism with Tacrolimus Concentration among Renal Transplant Recipients in Vietnam | Journal article | Vietnamese, renal transplant recipients | Kidney-transplant recipients | 68 | PCR-RFLP; direct sequencing | CYP3A5 | Primary meta-analysis | Included in the systematic review and primary quantitative synthesis. | 4 | 4/6 (Moderate) |
| S0009 | Vu Thi Thom et al., 2019 | Drug Resistance and Distribution of NAT2 Variants in Newly Diagnosed and Recurrent Vietnamese Pulmonary Tuberculosis Patients | Journal article | Vietnamese pulmonary tuberculosis patients | Pulmonary tuberculosis | 125 | PCR-RFLP; Sanger sequencing | NAT2 | Primary meta-analysis | Included in the systematic review and primary quantitative synthesis. | 5 | 4/6 (Moderate) |
| S0010 | Nhung Phuong Vu et al., 2023 | Copy Number Variations of Cytochrome P450 Genes in Kinh Vietnamese | Journal article | Kinh Vietnamese | Clinical or population sample | 154 | MLPA; PCR confirmation; MLPA; qPCR confirmation | CYP1B1, CYP2A6, CYP2C9, CYP2E1 | Primary meta-analysis | Included in the systematic review and primary quantitative synthesis. | 6 | 4/6 (Moderate) |
| S0011 | Nhung Phuong Vu et al., 2018 | Polymorphic Analysis of CYP2C9 Gene in Vietnamese Population | Journal article | Kinh Vietnamese | Population/healthy-volunteer sample | 100 | Sequencing; RFLP-PCR | CYP2C9 | Primary meta-analysis | Included in the systematic review and primary quantitative synthesis. | 7 | 5/6 (Low) |
| S0012 | Lan Thi Vu et al., 2025 | CYP2C9 rs1057910 Genotype and Its Association with Paraclinical Characteristics in Gout Patients in the Northeast Region of Vietnam | Journal article | Vietnamese gout patients; Northeast Vietnam | Gout patients | 139 | PCR; Sanger sequencing | CYP2C9 | Primary meta-analysis | Included in the systematic review and primary quantitative synthesis. | 8 | 4/6 (Moderate) |
| S0014 | M. I. Veiga et al., 2008 | Pharmacogenomics of CYP2A6, CYP2B6, CYP2C19, CYP2D6, CYP3A4, CYP3A5 and MDR1 in Vietnam | Journal article | Kinh Vietnamese | Clinical or population sample | 72 | PCR; PCR-RFLP; PCR; pyrosequencing; TaqMan; TaqMan; PCR-RFLP; pyrosequencing | ABCB1, CYP2A6, CYP2B6, CYP2C19, CYP2D6, CYP3A4, CYP3A5 | Primary meta-analysis | Included in the systematic review and primary quantitative synthesis. | 9 | 4/6 (Moderate) |
| S0015 | Ha Van Quang et al., 2024 | Association of UGT1A1 Gene Variants, Expression Levels, and Enzyme Concentrations with 2,3,7,8-TCDD Exposure in Individuals Exposed to Agent Orange/Dioxin | Journal article | Kinh Vietnamese; healthy controls | Drug-exposed or clinically selected participants | 100 | PCR; Sanger sequencing | UGT1A1 | Primary meta-analysis | Included in the systematic review and primary quantitative synthesis. | 10 | 4/6 (Moderate) |
| S0016 | Lê Hồng Công et al., 2015 | Tính đa hình G4268C của gen CYP2D6 trên bệnh nhân ung thư phổi | Journal article | Vietnamese controls | Lung-cancer case-control sample | 200 | PCR-RFLP; sequencing confirmation | CYP2D6 | Primary meta-analysis | Included in the systematic review and primary quantitative synthesis. | 11 | 5/6 (Low) |
| S0018 | Vũ Ngọc Trung et al., 2021 | Nghiên cứu tần suất đa hình gen CYP2C19 và mối liên quan với kết quả điều trị chống ngưng tập tiểu cầu ở người bệnh hội chứng mạch vành cấp | Doctoral thesis | Vietnamese acute coronary syndrome patients; Hanoi | Coronary/cardiovascular patients | 195 | PCR-RFLP; Sanger sequencing; Sequencing | CYP2C19 | Primary meta-analysis | Included in the systematic review and primary quantitative synthesis. | 12 | 4/6 (Moderate) |
| S0019 | Hien Thu Nguyen et al., 2025 | Genotype Frequency of HLA-B*58:01 and Its Association with Paraclinical Characteristics and PSORS1C1 rs9263726 in Gout Patients | Journal article | Vietnamese gout patients; Northeast Vietnam | Gout patients | 133 | PCR; Sanger sequencing | HLA-B, PSORS1C1 | Primary meta-analysis | Included in the systematic review and primary quantitative synthesis. | 13 | 4/6 (Moderate) |
| S0020 | Miriam Saiz-Rodríguez et al., 2023 | The Pharmacogenomics of Carbamazepine-Induced Cutaneous Adverse Drug Reactions in Southern Vietnam | Cross-sectional study | Southern Vietnamese; carbamazepine-tolerant epileptic controls | Epilepsy/carbamazepine-treated patients | 18 | Luminex flow cytometry; real-time PCR TaqMan assay | HLA-B | Qualitative synthesis only | Included in the systematic review; qualitative synthesis only—No independent population-frequency row could be reconstructed from the report. | 14 | 3/6 (Moderate) |
| S0021 | Nguyễn Trân Trân et al., 2023 | Khảo sát đặc điểm hẹp tắc mạch máu não trên MRI 3 Tesla và đa hình gen CYP2C19 ở bệnh nhân đột quỵ thiếu máu não | Cross-sectional study | Vietnamese ischemic stroke patients; Can Tho | Clinical or population sample | 83 | Real-time PCR | CYP2C19 | Primary meta-analysis | Included in the systematic review and primary quantitative synthesis. | 15 | 4/6 (Moderate) |
| S0022 | Nguyễn Trân Trân et al., 2021 | Khảo sát đặc điểm lâm sàng, túi phình và đa hình gen CYP2C19*2, CYP2C19*3 ở bệnh nhân phình mạch máu não điều trị bằng stent chuyển dòng tại Bệnh viện Đa khoa Quốc tế S.I.S Cần Thơ | Cross-sectional study | Vietnamese brain aneurysm patients treated with flow-diverter stent; Can Tho | Clinical or population sample | 30 | Real-time PCR | CYP2C19 | Primary meta-analysis | Included in the systematic review and primary quantitative synthesis. | 16 | 3/6 (Moderate) |
| S0025 | Hang Tong et al., 2025 | Genetic Landscape of VIP Pharmacogenomic Variants in the Kinh Vietnamese Population | Journal article | Kinh Vietnamese healthy population | Population/healthy-volunteer sample | 100 | Targeted panel sequencing; MiSeq; GATK variant calling | ABCG2, CYP2A6, CYP2B6, CYP2C19, CYP2C9, CYP4F2, DPYD, NAT2, NUDT15, SLCO1B1, TPMT, UGT1A1 | Primary meta-analysis | Included in the systematic review and primary quantitative synthesis. | 17 | 5/6 (Low) |
| S0026 | Tạ Anh Hoàng 1 et al., 2024 | Đặc điểm và mối liên quan của đa hình gen CYP2C19 với đề kháng clopidogrel ở bệnh nhân đặt stent động mạch vành | Cross-sectional study | Vietnamese coronary artery stent patients | Coronary/cardiovascular patients | 171 | PCR; Sanger sequencing | CYP2C19 | Primary meta-analysis | Included in the systematic review and primary quantitative synthesis. | 18 | 4/6 (Moderate) |
| S0027 | Do AD et al., 2019 | The prevalence of allele HLA-B*58:01 in Vietnamese gout patients with allopurinol-induced skin reactions | Journal article | Vietnamese gout patients with allopurinol-induced skin reactions | Clinically selected adverse-drug-reaction population | Not reported | HLA allele testing | HLA-B | Excluded after full-text assessment | Excluded after full-text assessment—clinically selected allopurinol adverse-reaction cases without an eligible unselected population-frequency denominator. | 19 | 2/6 (High) |
| S0028 | Vũ Thị Thơm et al., 2018 | Mối liên quan giữa độ ngưng tập tiểu cầu với kiểu gen CYP2C19*2, CYP2C19*3 và một số yếu tố khác trên 54 bệnh nhân đau thắt ngực không ổn định tại Viện Tim mạch Việt Nam | Cross-sectional study | Vietnamese unstable angina patients; Vietnam National Heart Institute | Clinical or population sample | 54 | PCR-RFLP; direct sequencing | CYP2C19 | Primary meta-analysis | Included in the systematic review and primary quantitative synthesis. | 20 | 4/6 (Moderate) |
| S0030 | Hà Thị Minh Thi et al., 2023 | Xác định đa hình CYP2C19*2 và CYP2C19*3 ở bệnh nhân bệnh lý dạ dày-tá tràng có nhiễm Helicobacter pylori bằng kỹ thuật PCR-RFLP | Cross-sectional study | Vietnamese H. pylori-positive gastroduodenal disease patients; Hue | Gastroduodenal disease/H. pylori | 112 | PCR-RFLP; Sanger sequencing validation | CYP2C19 | Primary meta-analysis | Included in the systematic review and primary quantitative synthesis. | 21 | 4/6 (Moderate) |
| S0031 | Thanh NH et al., 2009 | Đặc điểm các yếu tố độc lực cagA, vacA của vi khuẩn Helicobacter pylori và tính đa hình của enzym CYP2C19 trên bệnh nhân viêm loét dạ dày-tá tràng do nhiễm H. pylori chưa từng điều trị | Cross-sectional study | Vietnamese untreated H. pylori-positive peptic ulcer/gastroduodenal disease patients; American International Hospital | Gastroduodenal disease/H. pylori | 216 | PCR | CYP2C19 | Primary meta-analysis | Included in the systematic review and primary quantitative synthesis. | 22 | 4/6 (Moderate) |
| S0032 | Huỳnh Võ Hoài Thanh 1* et al., 2022 | Khảo sát đặc điểm đa hình gen CYP2C19 ở bệnh nhân nhồi máu cơ tim cấp tại Bệnh viện Đa khoa Hoàn Mỹ Cửu Long năm 2021-2022 | Cross-sectional study | Vietnamese acute myocardial infarction patients; Hoan My Cuu Long General Hospital | Coronary/cardiovascular patients | 86 | Real-time PCR | CYP2C19 | Primary meta-analysis | Included in the systematic review and primary quantitative synthesis. | 23 | 4/6 (Moderate) |
| S0033 | PHAM HONG THAM et al., 2022 | Khảo sát tính đa hình và ảnh hưởng của CYP3A5, CYP2C9 trên bệnh nhân bệnh động kinh Việt Nam | Doctoral thesis | Vietnamese epilepsy patients using carbamazepine; Vietnamese epilepsy patients using valproate and/or phenytoin | Epilepsy/carbamazepine-treated patients | 92 | Real-time PCR; TaqMan/SNP-Screen | CYP2C9, CYP3A5 | Primary meta-analysis | Included in the systematic review and primary quantitative synthesis. | 24 | 4/6 (Moderate) |
| S0036 | Hoàng Xuân Sử et al., 2023 | Khảo sát mối liên quan liều, nồng độ tacrolimus, mức lọc cầu thận với đa hình kiểu gen CYP3A5 ở người bệnh ghép thận | Journal article | Vietnamese renal transplant recipients; Military Hospital 103 | Kidney-transplant recipients | 88 | PCR-RFLP; Sanger sequencing confirmation | CYP3A5 | Primary meta-analysis | Included in the systematic review and primary quantitative synthesis. | 25 | 4/6 (Moderate) |
| S0037 | Triệu Tiến Sang 1* et al., 2025 | Tối ưu quy trình Tetra-primer ARMS-PCR xác định đa hình đơn nucleotide rs1799929 của gen NAT2 ở người tại một số tỉnh, thành phố của Việt Nam | Journal article | Vietnamese community samples; Lang Son, Nghe An, Hoa Binh | Clinical or population sample | 20 | Tetra-primer ARMS-PCR; Sanger sequencing validation | NAT2 | Qualitative synthesis only | Included in the systematic review; qualitative synthesis only—Method-development report; no independent analysis-ready Vietnamese frequency row. | 26 | 3/6 (Moderate) |
| S0041 | Rita Piedade et al., 2012 | Distribution and Impact on Interindividual Variability of CYP3A5 and NR1I2/PXR Genetic Variants | Journal article | Vietnamese subjects exposed to artemisinin | Clinical or population sample | 14 | Resequencing; MALDI-TOF MS genotyping | NR1I2/PXR | Qualitative synthesis only | Included in the systematic review; qualitative synthesis only—No independent analysis-ready Vietnamese frequency row for the prespecified outcomes. | 27 | 3/6 (Moderate) |
| S0043 | Thuy Thi PHAM 1 et al., 2018 | VKORC1 and CYP2C9*3 Polymorphisms and Their Impacts on Acenocoumarol Dosage in Vietnamese Heart Valve Replacement Patients | Cross-sectional study | Vietnamese heart valve replacement patients; Hanoi Heart Hospital | Clinical or population sample | 150 | PCR; Sanger sequencing | CYP2C9, VKORC1 | Primary meta-analysis | Included in the systematic review and primary quantitative synthesis. | 28 | 4/6 (Moderate) |
| S0044 | Pham TH et al., 2019 | Effect of CYP3A5 Genotypes on Steady-State Serum Carbamazepine Concentrations in Vietnamese Patients with Epilepsy | Journal article | Vietnamese epilepsy patients treated with carbamazepine monotherapy; Nhan Dan Gia Dinh Hospital | Epilepsy/carbamazepine-treated patients | 38 | PCR-RFLP; sequencing confirmation | CYP3A5 | Primary meta-analysis | Included in the systematic review and primary quantitative synthesis. | 29 | 3/6 (Moderate) |
| S0045 | Nguyễn Trần Khương Nhã * et al., 2023 | Nghiên cứu đặc điểm đa hình gen CYP2C19 và giá trị của tỉ số tế bào viêm trong khả năng tiên lượng biến cố tim mạch ở bệnh nhân hội chứng vành cấp | Cross-sectional study | Vietnamese acute coronary syndrome patients; Can Tho University of Medicine and Pharmacy Hospital | Clinical or population sample | 57 | Real-time PCR | CYP2C19 | Primary meta-analysis | Included in the systematic review and primary quantitative synthesis. | 30 | 4/6 (Moderate) |
| S0048 | Nguyễn Thị Thúy Mậu et al., 2017 | Bước đầu đánh giá mối liên quan giữa đa hình gen CYP2C19 và độ ngưng tập tiểu cầu trên bệnh nhân nhồi máu cơ tim cấp tại một số bệnh viện ở Hà Nội | Cross-sectional study | Vietnamese acute myocardial infarction patients; hospitals in Hanoi | Coronary/cardiovascular patients | 64 | PCR-RFLP; direct sequencing | CYP2C19 | Primary meta-analysis | Included in the systematic review and primary quantitative synthesis. | 31 | 4/6 (Moderate) |
| S0049 | Tien-Thanh Nguyen et al., 2019 | The Relationship between Hyperbilirubinemia and Promoter-Region and First-Exon UGT1A1 Polymorphisms in Vietnamese Newborns | Case-control study | Vietnamese full-term newborn controls; Binh Thuan General Hospital | Newborns | 50 | PCR; direct sequencing | UGT1A1 | Primary meta-analysis | Included in the systematic review and primary quantitative synthesis. | 32 | 4/6 (Moderate) |
| S0053 | Nhung Phuong Vu et al., 2019 | Single Nucleotide and Structural Variants of CYP2D6 Gene in the Kinh Vietnamese Population | Journal article | Kinh; Vietnamese | Population/healthy-volunteer sample | 136 | Sanger sequencing; MLPA; LR-PCR | CYP2D6 | Primary meta-analysis | Included in the systematic review and primary quantitative synthesis. | 33 | 5/6 (Low) |
| S0054 | Toan Nguyen Duy 1 et al., 2025 | CYP2C19 Genetic Variants Associated with Clopidogrel Resistance and Major Adverse Cardiovascular Events in Vietnamese Patients Undergoing Percutaneous Coronary Intervention | Cross-sectional study | Vietnamese ACS patients undergoing PCI; Military Hospital 103, Hanoi | Coronary/cardiovascular patients | 113 | ARMS-PCR; Sanger sequencing | CYP2C19 | Primary meta-analysis | Included in the systematic review and primary quantitative synthesis. | 34 | 4/6 (Moderate) |
| S0055 | Nguyen HH et al., 2021 | Dị ứng carbamazepine và mối liên quan với sự có mặt của allele HLA-B*15:02 và HLA-A*31:01 tại Bệnh viện Tâm Anh | Case-control study | Vietnamese carbamazepine-tolerant controls; Tam Anh Hospital | Epilepsy/carbamazepine-treated patients | 28 | Genotyping method not specified in extract; HLA allele testing | HLA-A, HLA-B | Qualitative synthesis only | Included in the systematic review; qualitative synthesis only—Clinically selected carbamazepine-reaction report; no eligible independent population-frequency row. | 35 | 2/6 (High) |
| S0057 | Van Son C et al., 2021 | Predominant HLA Alleles and Haplotypes in Mild Adverse Drug Reactions Caused by Allopurinol in Vietnamese Patients with Gout | Journal article | Vietnamese gout patients with mild allopurinol-induced adverse drug reactions | Clinically selected adverse-drug-reaction population | Not reported | HLA genotyping | HLA | Excluded after full-text assessment | Excluded after full-text assessment—clinically selected allopurinol adverse-reaction cases without an eligible unselected population-frequency denominator. | 36 | 2/6 (High) |
| S0060 | Ye-Ji Lim et al., 2014 | Genetic Polymorphisms of CYP2C9, CYP2C19, CYP2D6, CYP3A4, and CYP3A5 in Vietnamese-Koreans | Journal article | Vietnamese-Korean; offspring of Vietnamese mother and Korean father | Population/healthy-volunteer sample | 127 | SNaPshot; multiplex single-base extension | CYP2C19, CYP2C9, CYP2D6, CYP3A4, CYP3A5 | Primary meta-analysis | Included in the systematic review and primary quantitative synthesis. | 37 | 4/6 (Moderate) |
| S0061 | Sang Seop Lee et al., 2007 | Comparisons of CYP2C19 Genetic Polymorphisms between Korean and Vietnamese Populations | Journal article | Kinh; Vietnamese; healthy subjects from Hanoi | Population/healthy-volunteer sample | 165 | pyrosequencing | CYP2C19 | Primary meta-analysis | Included in the systematic review and primary quantitative synthesis. | 38 | 5/6 (Low) |
| S0062 | Ton DN et al., 2021 | Study of the UGT1A1*6 polymorphism in a Vietnamese population | Journal article | Vietnamese/Kinh participants | Genetic polymorphism study | Not reported | pyrosequencing | UGT1A1 | Qualitative synthesis only | Included in the systematic review; qualitative synthesis only—Sample size and genotype/allele denominator were insufficiently reported to reconstruct an independent quantitative row. | 39 | 2/6 (High) |
| S0064 | Sang Seop Lee a et al., 2004 | Identification and Functional Characterization of Novel CYP2J2 Variants: G312R Variant Causes Loss of Enzyme Catalytic Activity | Journal article | Viet Kinh; Vietnamese | Clinical or population sample | 159 | PCR-RFLP | CYP2J2 | Primary meta-analysis | Included in the systematic review and primary quantitative synthesis. | 40 | 4/6 (Moderate) |
| S0065 | Sang Seop Lee et al., 2008 | Genetic Polymorphism of Hepatocyte Nuclear Factor-4α Influences Human Cytochrome P450 2D6 Activity | Journal article | Viet Kinh; Vietnamese | Clinical or population sample | 139 | duplex pyrosequencing | HNF4A | Primary meta-analysis | Included in the systematic review and primary quantitative synthesis. | 41 | 4/6 (Moderate) |
| S0066 | Ming Ta Michael Lee et al., 2009 | VKORC1 Haplotypes in Five East-Asian Populations and Indians | Journal article | Vietnamese currently residing in Taiwan | Population/healthy-volunteer sample | 49 | Sequenom MassARRAY; Warfarin Dosage Prediction Kit; Sequenom MassARRAY | VKORC1 | Primary meta-analysis | Included in the systematic review and primary quantitative synthesis. | 42 | 4/6 (Moderate) |
| S0067 | Le Anh Tuan 1 et al., 2022 | Characteristics of N-Acetyltransferase 2 Phenotype Status and Pharmacokinetic Parameters of Vietnamese Pulmonary Tuberculosis Patients | Journal article | Vietnamese pulmonary tuberculosis patients | Pulmonary tuberculosis | 132 | PCR-RFLP; Sanger sequencing | NAT2 | Primary meta-analysis | Included in the systematic review and primary quantitative synthesis. | 43 | 4/6 (Moderate) |
| S0073 | Eun-Young Kim a et al., 2007 | Duplex Pyrosequencing Assay of the 388A>G and 521T>C SLCO1B1 Polymorphisms in Three Asian Populations | Journal article | Viet Kinh; healthy Vietnamese subjects; native residents of Hanoi | Population/healthy-volunteer sample | 104 | duplex pyrosequencing | SLCO1B1 | Primary meta-analysis | Included in the systematic review and primary quantitative synthesis. | 44 | 5/6 (Low) |
| S0078 | Hương BTT et al., 2022 | Nghiên cứu tần số kiểu gen, tần số alen đa hình CYP2E1*5B và CYP2E1*7B ở nhóm người Việt Nam sinh sống tại một số tỉnh phía Bắc | Journal article | Vietnamese living in northern provinces; patients/individuals attending Thai Nguyen National Hospital | Clinical or population sample | 96 | PCR; Sanger sequencing; nucleotide sequence analysis | CYP2E1 | Primary meta-analysis | Included in the systematic review and primary quantitative synthesis. | 45 | 4/6 (Moderate) |
| S0080 | TRẦN HÒA et al., 2020 | Nghiên cứu mối liên quan giữa kiểu gen giảm chức năng CYP2C19*2, *3 và tiên lượng ở bệnh nhân được can thiệp đặt stent động mạch vành có điều trị clopidogrel | Doctoral thesis | Vietnamese patients undergoing coronary stent implantation and treated with clopidogrel | Coronary/cardiovascular patients | 650 | real-time PCR; melting curve analysis; LightCycler Roche; Tibmobiol kit | CYP2C19 | Primary meta-analysis | Included in the systematic review and primary quantitative synthesis. | 46 | 4/6 (Moderate) |
| S0087 | Nguyễn Hải Hà et al., 2020 | Nghiên cứu đa hình gen UGT1A1*28 liên quan đến đáp ứng thuốc irinotecan ở người Kinh Việt Nam | Journal article | Kinh; healthy Vietnamese | Drug-exposed or clinically selected participants | 95 | PCR; direct sequencing of UGT1A1 promoter | UGT1A1 | Primary meta-analysis | Included in the systematic review and primary quantitative synthesis. | 47 | 4/6 (Moderate) |
| S0088 | Nguyễn Hải Hà et al., 2020 | Nghiên cứu đa hình kiểu gen CYP2C19*2, *3 và *17 trên người Việt Nam mắc bệnh động mạch vành | Journal article | Vietnamese patients with coronary artery disease; Bach Mai Hospital | Coronary/cardiovascular patients | 96 | PCR-RFLP; BamHI restriction enzyme; PCR-RFLP; MnlI restriction enzyme; PCR-RFLP; SmaI restriction enzyme | CYP2C19 | Primary meta-analysis | Included in the systematic review and primary quantitative synthesis. | 48 | 4/6 (Moderate) |
| S0089 | Pham-Thai Dung et al., 2025 | Predictive Value of Tacrolimus Concentration/Dose Ratio in the First Post-Transplant Week for CYP3A5 Polymorphism in Kidney-Transplant Recipients | Retrospective cohort study | Vietnamese kidney transplant recipients; Military Hospital 103, Hanoi | Kidney-transplant recipients | 86 | PCR-RFLP; subset confirmed by DNA sequencing | CYP3A5 | Primary meta-analysis | Included in the systematic review and primary quantitative synthesis. | 49 | 4/6 (Moderate) |
| S0092 | Dinh Doan Long et al., 2011 | Variability in the Frequency of Single-Nucleotide Polymorphisms of the N-Acetyltransferase 2 (NAT2) Gene in Vietnamese | Journal article | Vietnamese subjects; Hanoi | Clinical or population sample | 100 | PCR-RFLP; BamHI restriction enzyme; PCR-RFLP; KpnI restriction enzyme; PCR-RFLP; TaqI restriction enzyme | NAT2 | Primary meta-analysis | Included in the systematic review and primary quantitative synthesis. | 50 | 4/6 (Moderate) |
| S0097 | Isa Cavaco et al., 2007 | The Vietnamese Kinh Population Harbors Particular N-Acetyltransferase 2 Allele Frequencies | Letter to the editor | Mostly Kinh Vietnamese adults; National Institute of Malariology, Parasitology and Entomology, Hanoi | Population/healthy-volunteer sample | 72 | full NAT2 gene sequencing | NAT2 | Primary meta-analysis | Included in the systematic review and primary quantitative synthesis. | 51 | 5/6 (Low) |
| S0098 | Trương Đình Cẩm et al., 2024 | Nghiên cứu một số đặc điểm di truyền liên quan đến đề kháng thuốc clopidogrel | Cross-sectional study | Vietnamese patients with coronary artery disease using clopidogrel; Military Hospital 175 | Coronary/cardiovascular patients | 198 | Multiplex real-time PCR; TaqMan probe; real-time PCR; TaqMan probe | CYP2C19, PON1 | Primary meta-analysis | Included in the systematic review and primary quantitative synthesis. | 52 | 4/6 (Moderate) |
| S0100 | Nhung VP et al, 2020 | Study of CYP3A5 genetic polymorphism in a Vietnamese population | Journal article | Vietnamese/Kinh participants | Genetic polymorphism study | Not reported | Multiplex real-time PCR | CYP3A5 | Qualitative synthesis only | Included in the systematic review; qualitative synthesis only—Sample size and genotype/allele denominator were insufficiently reported to reconstruct an independent quantitative row. | 53 | 2/6 (High) |
| S0104 | Nhung Phuong Vu et al., 2019 | CYP2C19 Genetic Polymorphism in the Vietnamese Population | Journal article | Kinh; healthy unrelated Vietnamese subjects | Population/healthy-volunteer sample | 100 | Sanger sequencing of promoter and all exons; PCR-RFLP confirmation | CYP2C19 | Primary meta-analysis | Included in the systematic review and primary quantitative synthesis. | 54 | 5/6 (Low) |

Abbreviations: ADR, adverse drug reaction; HWE, Hardy–Weinberg equilibrium; NOS, Newcastle–Ottawa Scale; PCR, polymerase chain reaction; RoB, risk of bias.

**Complete bibliographic citations corresponding to the Reference no. column**

1. Lee SS, Kim K-M, Thi-Le H, Yea S-S, Cha I-J, Shin J-G. Genetic Polymorphism of CYP2C9 in a Vietnamese Kinh Population. Ther Drug Monit. 2005;27(2):208-10.

2. Yeo C-W, Lee S-J, Lee SS, Bae SK, Kim E-Y, Shon J-H, et al. Discovery of a novel allelic variant of CYP2C8, CYP2C8* 11, in Asian populations and its clinical effect on the rosiglitazone disposition in vivo. Drug Metabolism and Disposition. 2011;39(4):711-6.

3. Yamada S, Onda M, Kato S, Matsuda N, Matsuhisa T, Yamada N, et al. Genetic differences in CYP2C19 single nucleotide polymorphisms among four Asian populations. Journal of gastroenterology. 2001;36(10):669-72.

4. Xuan NT, Hop VQ, Kien TQ, Toan PQ, Thang LV, Binh HT, et al., editors. Frequencies and association of CYP3A5 polymorphism with tacrolimus concentration among renal transplant recipients in Vietnam. Transplantation Proceedings; 2022: Elsevier.

5. Vu TT, Le Thi L, Le Anh T, Pham THN, Nguyen TTH. Drug Resistance and Distribution of NAT2 Variants in Newly Diagnosed and Recurrent Vietnamese Pulmonary Tuberculosis Patients. VNU Journal of Science: Medical and Pharmaceutical Sciences. 2019;35(1).

6. Vu NP, Nguyen TD, Nguyen BH, Nguyen DT, Van Nong H, Nguyen HH. Copy number variations of cytochrome P450 genes in Kinh Vietnamese. Asian Biomedicine: Research, Reviews and News. 2023;17(2):84.

7. Vu NP, Ma TTH, Tran NTB, Huynh HTT, Nguyen TD, Nguyen DT, et al. Polymorphic analysis of CYP2C9 gene in Vietnamese population. Molecular biology reports. 2018;45(5):893-900.

8. Vu LT, Hoang YTT, Nguyen YT, Van Nguyen V, Nguyen HT, Do TH, et al. CYP2C9 rs1057910 Genotype and Its Association with Paraclinical Characteristics in Gout Patients in the Northeast Region of Vietnam. The Indonesian Biomedical Journal. 2025;17(4):343-52.

9. Veiga MI, Asimus S, Ferreira P, Martins J, Cavaco I, Ribeiro V, et al. Pharmacogenomics of Cyp2A6, Cyp2B6, Cyp2C19, Cyp2D6, Cyp3A4, Cyp3A5 and MDR1 in Vietnam. European journal of clinical pharmacology. 2009;65(4):355-63.

10. Van Quang H, Vuong NB, Trang BNL, Toan NL, Van Tong H. Association of UGT1A1 gene variants, expression levels, and enzyme concentrations with 2, 3, 7, 8-TCDD exposure in individuals exposed to Agent Orange/Dioxin. Scientific Reports. 2024;14(1):3315.

11. Vân Khánh T, Vân TT, Thịnh TH. Tính đa hình G4268C của gen CYP2D6 trên bệnh nhân ung thư phổi. Tạp chí Y học lâm sàng Bệnh viện Trung Ương Huế. 2015(30):89-93.

12. Trung VN. Nghiên cứu tần suất đa hình gen CYP2C19 và mối liên quan với kết quả điều trị chống ngưng tập tiểu cầu ở bệnh nhân hội chứng mạch vành cấp. 2021.

13. Nguyen HT, Bui HT, Hoang YTT, Hoang MH, Ngo MD, Nguyen MH, et al. Genotype Frequency of HLA-B* 58: 01 and Its Association with Paraclinical Characteristics and PSORS1C1 rs9263726 in Gout Patients. Diagnostics. 2025;15(16):2114.

14. Nguyen A-H, Sukasem C, Nguyen QN, Pham HT. The pharmacogenomics of carbamazepine-induced cutaneous adverse drug reaction in the South of Vietnam. Frontiers in Pharmacology. 2023;14:1217516.

15. Trân NT, Cường TC, Nga PTN. KHẢO SÁT ĐẶC ĐIỂM HẸP TẮC MẠCH MÁU NÃO TRÊN MRI 3 TESTLA VÀ ĐA HÌNH GEN CYP2C19 Ở BỆNH NHÂN ĐỘT QUỴ THIẾU MÁU NÃO. Tạp chí Y học Việt Nam. 2024;537(2).

16. Trân NT, Cường TC, Giang NL, Cường NM, Thắng LM, Phương LHL, et al. KHẢO SÁT ĐẶC ĐIỂM LÂM SÀNG, TÚI PHÌNH VÀ ĐA HÌNH GEN CYP2C19* 2, CYP2C19* 3 Ở BỆNH NHÂN PHÌNH MẠCH MÁU NÃO ĐIỀU TRỊ BẰNG STENT CHUYỂN DÒNG TẠI BỆNH VIỆN ĐA KHOA QUỐC TẾ SIS CẦN THƠ. Tạp chí Y Dược học Cần Thơ. 2021(41):203-9.

17. Tong H, Phan VTN, Nguyen T, Le L. Genetic Landscape of VIP Pharmacogenomic Variants in the Kinh Vietnamese Population. Pharmacogenomics and Personalized Medicine. 2025:251-61.

18. Toàn ND, Hoàng TA, Tổng HV, Cẩm TĐ. ĐẶC ĐIỂM VÀ MỐI LIÊN QUAN CỦA ĐA HÌNH GEN CYP2C19 VỚI ĐỀ KHÁNG CLOPIDOGREL Ở BỆNH NHÂN ĐẶT STENT ĐỘNG MẠCH VÀNH. Tạp chí Y Dược học Quân sự. 2024;49(8):113-23.

19. Do AD, Le Gia HL, Do MD, Mai TP. The prevalence of allele HLA-B*58:01 in Vietnamese gout patients with allopurinol-induced skin reactions. Ho Chi Minh City Journal of Medicine. 2019;23:80-4.

20. Thơm VT, Thảo VP, Trung VN, Mậu NTT. Mối liên quan giữa độ ngưng tập tiểu cầu với kiểu gen CYP2C19* 2, CYP2C19* 3 và một số yếu tố khác trên 54 bệnh nhân đau thắt ngực không ổn định tại Viện Tim mạch Việt Nam. Tạp chí Khoa học ĐHQGHN: Khoa học Y Dược. 2018;34(1):74-81.

21. Thi HTM, Ngân NTM, Hương NTD. Xác định đa hình CYP2C19* 2 và CYP2C19* 3 ở bệnh nhân bệnh lý dạ dày-tá tràng có nhiễm Helicobacter pylori bằng kỹ thuật PCR-RFLP. Tạp chí Y Dược Huế. 2023;13(5):178-85.

22. Thanh NH, Huy ÂN, Thảo TTM, Tường TTK. Đặc điểm các yếu tố độc lực cagA, vacA của vi khuẩn Helicobacter pylori và tính đa hình của Enzym CYP2C19 trên bệnh nhân viêm loét dạ dày tá tràng do nhiễm H. pylori chưa từng điều trị.

23. Thanh HVH, Diễm NT, Nga PTN. Khảo sát đặc điểm đa hình gen cyp2c19 ở bệnh nhân nhồi máu cơ tim cấp tại Bệnh viện Đa khoa Hoàn Mỹ Cửu Long năm 2021-2022. Tạp chí Y Dược học Cần Thơ. 2022(48):70-7.

24. THẮM PH. KHẢO SÁT TÍNH ĐA HÌNH VÀ ẢNH HƯỞNG CỦA CYP3A5, CYP2C9 TRÊN BỆNH NHÂN BỆNH ĐỘNG KINH VIỆT NAM.

25. Sử HX, Bình HT, Khôi NV, Toản PQ. Khảo sát mối liên quan liều, nồng độ tacrolimus, mức lọc cầu thận với đa hình kiểu gen CYP3A5 ở người bệnh ghép thận. Journal of 108-Clinical Medicine and Phamarcy. 2023.

26. Triệu TS, Trần VK, Nguyễn VP, Tạ TB, Phạm XTA. Tối ưu quy trình Tetra primer ARMS PCR xác định đa hình đơn nucleotide rs1799929 của gen NAT2 ở người tại một số tỉnh, thành phố của Việt Nam. Tạp chí Khoa học và Công nghệ Việt Nam-B. 2025;67(6B):59-.

27. Piedade R, Schaeffeler E, Winter S, Asimus S, Schwab M, Ashton M, et al. PXR variants and artemisinin use in Vietnamese subjects: frequency distribution and impact on the interindividual variability of CYP3A induction by artemisinin. Antimicrobial agents and chemotherapy. 2012;56(4):2153-7.

28. PHAM TT, NHUNG PTH, Van Thanh T, DINH LD, PHAM KT, VU TT. VKORC1 and CYP2C9* 3 polymorphisms and their impacts to acenocoumarol dosage in Vietnamese heart valve replacement patients. Walailak Journal of Science and Technology (WJST). 2019;16(3):207-15.

29. Pham TH, Huynh HTM, Vo HT, Tran HM. Effect of CYP3A5 genotypes on serum carbamazepine concentrations at steady-state in Vietnamese epileptic patients. Research Journal of Pharmacy and Technology. 2020;13(6):2802-6.

30. Nhã NTK, An TV, An HT, Nga PTN. NGHIÊN CỨU ĐẶC ĐIỂM ĐA HÌNH GEN CYP2C19 VÀ GIÁ TRỊ CỦA TỈ SỐ TẾ BÀO VIÊM TRONG KHẢ NĂNG TIÊN LƯỢNG BIẾN CỐ TIM MẠCH Ở BỆNH NHÂN HỘI CHỨNG VÀNH CẤP. Tạp chí Y Dược học Cần Thơ. 2023(61):343-50.

31. Mậu NTT, Trung VN, Hiếu NH. Bước đầu đánh giá mối liên quan giữa đa hình gen CYP2C19 và độ ngưng tập tiểu cầu trên bệnh nhân nhồi máu cơ tim cấp tại một số bệnh viện ở Hà Nội. Tạp chí Khoa học ĐHQGHN, Khoa học Y Dược. 2017;33(2):68-74.

32. Nguyen T-T, Zhao W, Yang X, Zhong D-N. The relationship between hyperbilirubinemia and the promoter region and first exon of UGT1A1 gene polymorphisms in Vietnamese newborns. Pediatric research. 2020;88(6):940-4.

33. Nguyen HH, Ma TTH, Vu NP, Bach QTN, Vu TH, Nguyen TD, et al. Single nucleotide and structural variants of CYP2D6 gene in Kinh Vietnamese population. Medicine. 2019;98(22):e15891.

34. Duy TN, Oanh ON, Trung KN, Le Ha K, Kim HHT, Lien HNT, et al. CYP2C19 Genetic Variants Associated with Clopidogrel Resistance and Major Adverse Cardiovascular Events in Vietnamese Patients Undergoing Percutaneous Coronary Intervention. Cardiovascular & Hematological Disorders-Drug Targets. 2025.

35. Nguyễn ĐT, Nguyễn VL, Nguyễn TVA, Chu VS. Dị ứng Carbamazepine và mối liên quan với sự có mặt của allele hla-b* 15: 02 và hla-a* 31: 01 tại bệnh viện Tâm Anh. Tạp chí Y học Việt Nam. 2022;518(2).

36. Van Son C, Loan NTH, Trang TH, Thinh LX, Khanh NB, Nhung LTH, et al. Predominant HLA Alleles and Haplotypes in Mild Adverse Drug Reactions Caused by Allopurinol in Vietnamese Patients with Gout. Diagnostics. 2021;11(9):1611.

37. Lim Y-J, Cha E-Y, Jung H-E, Ghim J-L, Lee S-J, Kim E-Y, et al. Genetic polymorphisms of CYP2C9, CYP2C19, CYP2D6, CYP3A4, and CYP3A5 in Vietnamese-Koreans. Translational and Clinical Pharmacology. 2014;22(2):70-7.

38. Lee SS, Lee S-J, Gwak J, Jung H-J, Thi-Le H, Song I-S, et al. Comparisons of CYP2C19 genetic polymorphisms between Korean and Vietnamese populations. Therapeutic drug monitoring. 2007;29(4):455-9.

39. Ton ND, Hoa NTT, Nam NH, Nhung VP, Yen HTT, Ha NH. Tần số alen UGT1A1*6 ở người dân tộc Kinh Việt Nam. UGT1A1*6 Allele Frequency in Vietnamese Kinh Ethnic Group. 2021;226(05):110-7.

40. Lee SS, Jeong H-E, Liu K-H, Ryu J-Y, Moon T, Yoon CN, et al. Identification and functional characterization of novel CYP2J2 variants: G312R variant causes loss of enzyme catalytic activity. Pharmacogenetics and genomics. 2005;15(2):105-13.

41. Lee SS, Cha EY, Jung HJ, Shon JH, Kim EY, Yeo CW, et al. Genetic polymorphism of hepatocyte nuclear factor‐4α influences human cytochrome P450 2D6 activity. Hepatology. 2008;48(2):635-45.

42. Lee MTM, Chen C-H, Chuang H-P, Lu L-S, Chou C-H, Chen Y-T, et al. VKORC1 haplotypes in five East-Asian populations and Indians. Pharmacogenomics. 2009;10(10):1609-16.

43. Le Anh T, Bui SN, Le Thi L, Vu DAM, Le Ba H, Tran TTT, et al. Characteristics of N-acetyltransferase 2 Phenotype Status and Pharmacokinetic Parameters of Vietnam Pulmonary Tuberculosis Patients. VNU Journal of Science: Medical and Pharmaceutical Sciences. 2022;38(3).

44. Kim E-Y, Cho D-Y, Shin H-J, Lee S-S, Shon J-H, Shin J-G, et al. Duplex pyrosequencing assay of the 388A> G and 521T> C SLCO1B1 polymorphisms in three Asian populations. Clinica Chimica Acta. 2008;388(1-2):68-72.

45. Hương BTT, Thảo NP, Yến NT, Yến HTT. NGHIÊN CỨU TẦN SỐ KIỂU GEN, TẦN SỐ ALEN ĐA HÌNH CYP2E1* 5B VÀ CYP2E1* 7B Ở NHÓM NGƯỜI VIỆT NAM SINH SỐNG TẠI MỘT SỐ TỈNH PHÍA BẮC. TNU Journal of Science and Technology. 2023;228(01):211-8.

46. Hòa T. Nghiên cứu mối liên quan giữa kiểu gen giảm chức năng CYP2C19* 2,* 3 và tiên lượng ở bệnh nhân được can thiệp đặt stent động mạch vành có điều trị Clopidogrel. Đại học Y Dược TP Hồ Chí Minh. 2020;118.

47. Nguyen HH, Nguyen TTH, Vu BG, Vu PN, Hoang TTY, Nguyen DT, et al. Nghiên cứu đa hình gen UGT1A1*28 liên quan đến đáp ứng thuốc irinotecan ở người Kinh Việt Nam. Study of UGT1A1*28 polymorphism related to irinotecan response in Kinh Vietnamese. 2020;18(3):425-35.

48. Ha NH, Hoa NTT. Studying of cyp2c19* 2,* 3 and* 17 polymorphism in Vietnamese patients with coronary artery disease. Vietnam Journal of Biotechnology. 2020;18(1):41-8.

49. Dung P-T, Su H-X, Tue N-C, Ben N-H, Phuong N-M, Tran T-N, et al. Predictive value of tacrolimus concentration/dose ratio in first post-transplant week for CYP3A5-polymorphism in kidney-transplant recipients. World Journal of Transplantation. 2025;15(2):103247.

50. Dinh DL, Pham THN, Tran TTA, Hoang HY. Variability in the frequency of Single nucleotide polymorphisms of N-acetyl transferase (NAT2) gene in Vietnamese. VNU Journal of Science: Natural Sciences and Technology. 2011;27(4).

51. Cavaco I, Asimus S, Peyrard-Janvid M, Ferreira PE, Veiga MI, Hai TN, et al. The Vietnamese Khin population harbors particular N-acetyltransferase 2 allele frequencies. Clinical chemistry. 2007;53(11):1977-9.

52. Cẩm TĐ, Hoàng TA, Thắng TVC, An LB, Linh LGH, Minh ĐĐ. NGHIÊN CỨU MỘT SỐ ĐẶC ĐIỂM DI TRUYỀN LIÊN QUAN ĐẾN ĐỀ KHÁNG THUỐC CLOPIDOGREL. Tạp chí Y học Việt Nam. 2024;537(2).

53. Nhung VP, Ton ND, Ha NH. Study of CYP3A5 Genetic Polymorphism in Vietnamese Kinh Ethnic Group. Academia Journal of Biology. 2020;42(1):111-23.

54. Vu NP, Nguyen HTT, Tran NTB, Nguyen TD, Huynh HTT, Nguyen XT, et al. CYP2C19 genetic polymorphism in the Vietnamese population. Annals of Human Biology. 2019;46(6):491-7.
