# Supplementary material for: Vietnamese pharmacogenomic variation in global context: a systematic review and meta-analysis for clinical implementation
Source: Front Pharmacol. 2026 Sep 3;17:1921144. doi: 10.3389/fphar.2026.1921144 (PMC13582518; doi:10.3389/fphar.2026.1921144)
Supplement: Supplementary file 2 [file Table2.docx]

**Supplementary Table S2. Study–variant source traceability for records contributing to the primary Vietnamese meta-analysis**

The table contains 173 analysis-ready study–variant rows from 46 independent reports or datasets after report linkage and overlap control. The aggregate row-level sample-size sum is 19,780 and must not be interpreted as the number of unique participants in the review.

Reference/effect states may denote nucleotides, star alleles, haplotypes, copy-number states, structural alleles, or HLA alleles. Main-text harmonization combined only three verified synonym groups with matching rsID and effect-allele orientation: CYP2B6 rs3745274 G>T, SLCO1B1 rs2306283 A>G, and VKORC1 rs9934438 C>T. CYP2D6 rs1065852 and rs3892097 are retained as single-marker source rows and are not treated as CYP2D6*10 or CYP2D6*4 star-allele estimates.

| **Extraction row ID** | **Source study ID** | **Source page** | **Gene** | **Allele/variant** | **rsID** | **Reference allele/state** | **Effect allele/state** | **Participants, n** | **n(ref/ref)** | **n(ref/effect)** | **n(effect/effect)** | **Effect-allele/state count** | **Allele/state number** | **Effect-allele/state frequency, %** | **HWE assessment** | **Genotyping method** | **Population/ethnicity** |
| --- | --- | --- | --- | --- | --- | --- | --- | --- | --- | --- | --- | --- | --- | --- | --- | --- | --- |
| Study_S0014_VNM_16 | S0014 | 5.0 | *ABCB1* | 3435C>T | rs1045642 | C | T | 72 | 23 | 40 | 9 | 58 | 144 | 40.28 | Pass (HWE p>=0.05) | TaqMan | Kinh Vietnamese |
| Study_S0025_VNM_07 | S0025 | 6.0 | *ABCG2* | rs2231142 | rs2231142 | G | T | 100 | 41 | 46 | 13 | 72 | 200 | 36.00 | Pass (HWE p>=0.05) | Targeted panel sequencing; MiSeq; GATK variant calling | Kinh Vietnamese healthy population |
| Study_S0010_VNM_01 | S0010 | 4.0 | *CYP1B1* | Dup | — | WT | DUP | 154 | 151 | 3 | 0 | 3 | 308 | 0.97 | Pass (HWE p>=0.05) | MLPA; qPCR confirmation | Kinh Vietnamese |
| Study_S0025_VNM_25 | S0025 | 7.0 | *CYP2A6* | *11 | rs28399447 | A | G | 100 | 97 | 3 | 0 | 3 | 200 | 1.50 | Pass (HWE p>=0.05) | Targeted panel sequencing; MiSeq; GATK variant calling | Kinh Vietnamese healthy population |
| Study_S0025_VNM_26 | S0025 | 7.0 | *CYP2A6* | *15 | rs199916117 | T | C | 100 | 99 | 1 | 0 | 1 | 200 | 0.50 | Pass (HWE p>=0.05) | Targeted panel sequencing; MiSeq; GATK variant calling | Kinh Vietnamese healthy population |
| Study_S0014_VNM_01 | S0014 | 5.0 | *CYP2A6* | *4 | — | WT | DEL | 72 | 55 | 17 | 0 | 17 | 144 | 11.81 | Pass (HWE p>=0.05) | PCR; pyrosequencing | Kinh Vietnamese |
| Study_S0014_VNM_02 | S0014 | 5.0 | *CYP2A6* | *5 | rs5031017 | G | T | 72 | 51 | 21 | 0 | 21 | 144 | 14.58 | Pass (HWE p>=0.05) | pyrosequencing | Kinh Vietnamese |
| Study_S0010_VNM_02 | S0010 | 4.0 | *CYP2A6* | Del | — | WT | DEL | 154 | 124 | 26 | 4 | 34 | 308 | 11.04 | Pass (HWE p>=0.05) | MLPA; PCR confirmation | Kinh Vietnamese |
| Study_S0025_VNM_24 | S0025 | 7.0 | *CYP2A6* | rs1809810 | rs1809810 | T | A | 100 | 96 | 4 | 0 | 4 | 200 | 2.00 | Pass (HWE p>=0.05) | Targeted panel sequencing; MiSeq; GATK variant calling | Kinh Vietnamese healthy population |
| Study_S0025_VNM_27 | S0025 | 7.0 | *CYP2B6* | *2 | rs8192709 | C | T | 100 | 89 | 11 | 0 | 11 | 200 | 5.50 | Pass (HWE p>=0.05) | Targeted panel sequencing; MiSeq; GATK variant calling | Kinh Vietnamese healthy population |
| Study_S0014_VNM_04 | S0014 | 5.0 | *CYP2B6* | *4 | rs2279343 | A | G | 72 | 32 | 29 | 11 | 51 | 144 | 35.42 | Pass (HWE p>=0.05) | PCR-RFLP | Kinh Vietnamese |
| Study_S0014_VNM_05 | S0014 | 5.0 | *CYP2B6* | *5 | rs3211371 | C | T | 72 | 72 | 0 | 0 | 0 | 144 | 0.00 | Not informative | PCR-RFLP | Kinh Vietnamese |
| Study_S0014_VNM_03 | S0014 | 5.0 | *CYP2B6* | 516G>T | rs3745274 | G | T | 72 | 38 | 29 | 5 | 39 | 144 | 27.08 | Pass (HWE p>=0.05) | PCR-RFLP | Kinh Vietnamese |
| Study_S0025_VNM_28 | S0025 | 7.0 | *CYP2B6* | rs3745274 | rs3745274 | G | T | 100 | 59 | 36 | 5 | 46 | 200 | 23.00 | Pass (HWE p>=0.05) | Targeted panel sequencing; MiSeq; GATK variant calling | Kinh Vietnamese healthy population |
| Study_S0088_03 | S0088 | 4.0 | *CYP2C19* | *17 | rs11188072 | C | T | 96 | 94 | 2 | 0 | 2 | 192 | 1.04 | Pass (HWE p>=0.05) | PCR-RFLP; MnlI restriction enzyme | Vietnamese patients with coronary artery disease; Bach Mai Hospital |
| Study_S0018_VNM_03 | S0018 | 70.0 | *CYP2C19* | *17 | rs12248560 | C | T | 195 | 191 | 4 | 0 | 4 | 390 | 1.03 | Pass (HWE p>=0.05) | Sequencing | Vietnamese acute coronary syndrome patients; Hanoi |
| Study_S0060_05 | S0060 | 5.0 | *CYP2C19* | *17 | rs12248560 | C | T | 127 | 122 | 5 | 0 | 5 | 254 | 1.97 | Pass (HWE p>=0.05) | SNaPshot; multiplex single-base extension | Vietnamese-Korean; offspring of Vietnamese mother and Korean father |
| Study_S0104_KINH_03 | S0104 | 4.0 | *CYP2C19* | *17 | rs12248560 | C | T | 100 | 98 | 2 | 0 | 2 | 200 | 1.00 | Pass (HWE p>=0.05) | Sanger sequencing of promoter and all exons; PCR-RFLP confirmation | Kinh; healthy unrelated Vietnamese subjects |
| Study_S0007_VNM_01 | S0007 | 3.0 | *CYP2C19* | *2 | rs4244285 | G | A | 90 | 48 | 36 | 6 | 48 | 180 | 26.67 | Pass (HWE p>=0.05) | PCR-RFLP | Vietnamese |
| Study_S0014_VNM_06 | S0014 | 5.0 | *CYP2C19* | *2 | rs4244285 | G | A | 72 | 33 | 34 | 5 | 44 | 144 | 30.56 | Pass (HWE p>=0.05) | TaqMan; PCR-RFLP | Kinh Vietnamese |
| Study_S0018_VNM_01 | S0018 | 70.0 | *CYP2C19* | *2 | rs4244285 | G | A | 195 | 95 | 83 | 17 | 117 | 390 | 30.00 | Pass (HWE p>=0.05) | PCR-RFLP; Sanger sequencing | Vietnamese acute coronary syndrome patients; Hanoi |
| Study_S0021_VNM_01 | S0021 | 3.0 | *CYP2C19* | *2 | rs4244285 | G | A | 83 | 36 | 39 | 8 | 55 | 166 | 33.13 | Pass (HWE p>=0.05) | Real-time PCR | Vietnamese ischemic stroke patients; Can Tho |
| Study_S0022_VNM_01 | S0022 | 5.0 | *CYP2C19* | *2 | rs4244285 | G | A | 30 | 18 | 8 | 4 | 16 | 60 | 26.67 | Pass (HWE p>=0.05) | Real-time PCR | Vietnamese brain aneurysm patients treated with flow-diverter stent; Can Tho |
| Study_S0025_VNM_14 | S0025 | 6.0 | *CYP2C19* | *2 | rs4244285 | G | A | 100 | 56 | 38 | 6 | 50 | 200 | 25.00 | Pass (HWE p>=0.05) | Targeted panel sequencing; MiSeq; GATK variant calling | Kinh Vietnamese healthy population |
| Study_S0026_VNM_01 | S0026 | 6.0 | *CYP2C19* | *2 | rs4244285 | G | A | 171 | 94 | 65 | 12 | 89 | 342 | 26.02 | Pass (HWE p>=0.05) | PCR; Sanger sequencing | Vietnamese coronary artery stent patients |
| Study_S0028_VNM_01 | S0028 | 3.0 | *CYP2C19* | *2 | rs4244285 | G | A | 54 | 28 | 20 | 6 | 32 | 108 | 29.63 | Pass (HWE p>=0.05) | PCR-RFLP; direct sequencing | Vietnamese unstable angina patients; Vietnam National Heart Institute |
| Study_S0030_VNM_01 | S0030 | 5.0 | *CYP2C19* | *2 | rs4244285 | G | A | 112 | 55 | 49 | 8 | 65 | 224 | 29.02 | Pass (HWE p>=0.05) | PCR-RFLP; Sanger sequencing validation | Vietnamese H. pylori-positive gastroduodenal disease patients; Hue |
| Study_S0031_VNM_01 | S0031 | 7.0 | *CYP2C19* | *2 | rs4244285 | G | A | 216 | 102 | 94 | 20 | 134 | 432 | 31.02 | Pass (HWE p>=0.05) | PCR | Vietnamese untreated H. pylori-positive peptic ulcer/gastroduodenal disease patients; American International Hospital |
| Study_S0032_VNM_01 | S0032 | 4.0 | *CYP2C19* | *2 | rs4244285 | G | A | 86 | 52 | 32 | 2 | 36 | 172 | 20.93 | Pass (HWE p>=0.05) | Real-time PCR | Vietnamese acute myocardial infarction patients; Hoan My Cuu Long General Hospital |
| Study_S0045_VNM_01 | S0045 | 4.0 | *CYP2C19* | *2 | rs4244285 | G | A | 57 | 32 | 23 | 2 | 27 | 114 | 23.68 | Pass (HWE p>=0.05) | Real-time PCR | Vietnamese acute coronary syndrome patients; Can Tho University of Medicine and Pharmacy Hospital |
| Study_S0048_VNM_01 | S0048 | 3.0 | *CYP2C19* | *2 | rs4244285 | G | A | 64 | 29 | 33 | 2 | 37 | 128 | 28.91 | Warning (HWE p<0.05) | PCR-RFLP; direct sequencing | Vietnamese acute myocardial infarction patients; hospitals in Hanoi |
| Study_S0054_01 | S0054 | 5.0 | *CYP2C19* | *2 | rs4244285 | G | A | 113 | 56 | 45 | 12 | 69 | 226 | 30.53 | Pass (HWE p>=0.05) | ARMS-PCR; Sanger sequencing | Vietnamese ACS patients undergoing PCI; Military Hospital 103, Hanoi |
| Study_S0060_03 | S0060 | 5.0 | *CYP2C19* | *2 | rs4244285 | G | A | 127 | 69 | 51 | 7 | 65 | 254 | 25.59 | Pass (HWE p>=0.05) | SNaPshot; multiplex single-base extension | Vietnamese-Korean; offspring of Vietnamese mother and Korean father |
| Study_S0080_01 | S0080 | 67.0 | *CYP2C19* | *2 | rs4244285 | G | A | 650 | 298 | 331 | 21 | 373 | 1,300 | 28.69 | Fail (HWE p<0.001) | real-time PCR; melting curve analysis; LightCycler Roche; Tibmobiol kit | Vietnamese patients undergoing coronary stent implantation and treated with clopidogrel |
| Study_S0088_01 | S0088 | 4.0 | *CYP2C19* | *2 | rs4244285 | G | A | 96 | 50 | 38 | 8 | 54 | 192 | 28.12 | Pass (HWE p>=0.05) | PCR-RFLP; SmaI restriction enzyme | Vietnamese patients with coronary artery disease; Bach Mai Hospital |
| Study_S0098_01 | S0098 | 3.0 | *CYP2C19* | *2 | rs4244285 | G | A | 198 | 109 | 75 | 14 | 103 | 396 | 26.01 | Pass (HWE p>=0.05) | Multiplex real-time PCR; TaqMan probe | Vietnamese patients with coronary artery disease using clopidogrel; Military Hospital 175 |
| Study_S0104_KINH_01 | S0104 | 4.0 | *CYP2C19* | *2 | rs4244285 | G | A | 100 | 62 | 35 | 3 | 41 | 200 | 20.50 | Pass (HWE p>=0.05) | Sanger sequencing of promoter and all exons; PCR-RFLP confirmation | Kinh; healthy unrelated Vietnamese subjects |
| Study_S0025_VNM_15 | S0025 | 6.0 | *CYP2C19* | *26 | rs375781227 | G | A | 100 | 99 | 1 | 0 | 1 | 200 | 0.50 | Pass (HWE p>=0.05) | Targeted panel sequencing; MiSeq; GATK variant calling | Kinh Vietnamese healthy population |
| Study_S0007_VNM_02 | S0007 | 3.0 | *CYP2C19* | *3 | rs4986893 | G | A | 90 | 70 | 16 | 4 | 24 | 180 | 13.33 | Warning (HWE p<0.05) | PCR-RFLP | Vietnamese |
| Study_S0014_VNM_07 | S0014 | 5.0 | *CYP2C19* | *3 | rs4986893 | G | A | 72 | 63 | 9 | 0 | 9 | 144 | 6.25 | Pass (HWE p>=0.05) | PCR-RFLP | Kinh Vietnamese |
| Study_S0018_VNM_02 | S0018 | 70.0 | *CYP2C19* | *3 | rs4986893 | G | A | 195 | 171 | 23 | 1 | 25 | 390 | 6.41 | Pass (HWE p>=0.05) | PCR-RFLP; Sanger sequencing | Vietnamese acute coronary syndrome patients; Hanoi |
| Study_S0021_VNM_02 | S0021 | 3.0 | *CYP2C19* | *3 | rs4986893 | G | A | 83 | 80 | 3 | 0 | 3 | 166 | 1.81 | Pass (HWE p>=0.05) | Real-time PCR | Vietnamese ischemic stroke patients; Can Tho |
| Study_S0022_VNM_02 | S0022 | 5.0 | *CYP2C19* | *3 | rs4986893 | G | A | 30 | 28 | 2 | 0 | 2 | 60 | 3.33 | Pass (HWE p>=0.05) | Real-time PCR | Vietnamese brain aneurysm patients treated with flow-diverter stent; Can Tho |
| Study_S0025_VNM_13 | S0025 | 6.0 | *CYP2C19* | *3 | rs4986893 | G | A | 100 | 92 | 8 | 0 | 8 | 200 | 4.00 | Pass (HWE p>=0.05) | Targeted panel sequencing; MiSeq; GATK variant calling | Kinh Vietnamese healthy population |
| Study_S0026_VNM_02 | S0026 | 6.0 | *CYP2C19* | *3 | rs4986893 | G | A | 171 | 149 | 20 | 2 | 24 | 342 | 7.02 | Pass (HWE p>=0.05) | PCR; Sanger sequencing | Vietnamese coronary artery stent patients |
| Study_S0028_VNM_02 | S0028 | 3.0 | *CYP2C19* | *3 | rs4986893 | G | A | 54 | 48 | 6 | 0 | 6 | 108 | 5.56 | Pass (HWE p>=0.05) | PCR-RFLP; direct sequencing | Vietnamese unstable angina patients; Vietnam National Heart Institute |
| Study_S0030_VNM_02 | S0030 | 5.0 | *CYP2C19* | *3 | rs4986893 | G | A | 112 | 105 | 7 | 0 | 7 | 224 | 3.12 | Pass (HWE p>=0.05) | PCR-RFLP; Sanger sequencing validation | Vietnamese H. pylori-positive gastroduodenal disease patients; Hue |
| Study_S0031_VNM_02 | S0031 | 7.0 | *CYP2C19* | *3 | rs4986893 | G | A | 216 | 198 | 18 | 0 | 18 | 432 | 4.17 | Pass (HWE p>=0.05) | PCR | Vietnamese untreated H. pylori-positive peptic ulcer/gastroduodenal disease patients; American International Hospital |
| Study_S0032_VNM_02 | S0032 | 4.0 | *CYP2C19* | *3 | rs4986893 | G | A | 86 | 77 | 8 | 1 | 10 | 172 | 5.81 | Pass (HWE p>=0.05) | Real-time PCR | Vietnamese acute myocardial infarction patients; Hoan My Cuu Long General Hospital |
| Study_S0045_VNM_02 | S0045 | 4.0 | *CYP2C19* | *3 | rs4986893 | G | A | 57 | 47 | 10 | 0 | 10 | 114 | 8.77 | Pass (HWE p>=0.05) | Real-time PCR | Vietnamese acute coronary syndrome patients; Can Tho University of Medicine and Pharmacy Hospital |
| Study_S0048_VNM_02 | S0048 | 3.0 | *CYP2C19* | *3 | rs4986893 | G | A | 64 | 56 | 8 | 0 | 8 | 128 | 6.25 | Pass (HWE p>=0.05) | PCR-RFLP; direct sequencing | Vietnamese acute myocardial infarction patients; hospitals in Hanoi |
| Study_S0054_02 | S0054 | 5.0 | *CYP2C19* | *3 | rs4986893 | G | A | 113 | 101 | 11 | 1 | 13 | 226 | 5.75 | Pass (HWE p>=0.05) | ARMS-PCR; Sanger sequencing | Vietnamese ACS patients undergoing PCI; Military Hospital 103, Hanoi |
| Study_S0060_04 | S0060 | 5.0 | *CYP2C19* | *3 | rs4986893 | G | A | 127 | 113 | 14 | 0 | 14 | 254 | 5.51 | Pass (HWE p>=0.05) | SNaPshot; multiplex single-base extension | Vietnamese-Korean; offspring of Vietnamese mother and Korean father |
| Study_S0061_02 | S0061 | 3.0 | *CYP2C19* | *3 | rs4986893 | G | A | 165 | 150 | 14 | 1 | 16 | 330 | 4.85 | Pass (HWE p>=0.05) | pyrosequencing | Kinh; Vietnamese; healthy subjects from Hanoi |
| Study_S0080_02 | S0080 | 67.0 | *CYP2C19* | *3 | rs4986893 | G | A | 650 | 555 | 95 | 0 | 95 | 1,300 | 7.31 | Warning (HWE p<0.05) | real-time PCR; melting curve analysis; LightCycler Roche; Tibmobiol kit | Vietnamese patients undergoing coronary stent implantation and treated with clopidogrel |
| Study_S0088_02 | S0088 | 4.0 | *CYP2C19* | *3 | rs4986893 | G | A | 96 | 90 | 6 | 0 | 6 | 192 | 3.12 | Pass (HWE p>=0.05) | PCR-RFLP; BamHI restriction enzyme | Vietnamese patients with coronary artery disease; Bach Mai Hospital |
| Study_S0098_02 | S0098 | 3.0 | *CYP2C19* | *3 | rs4986893 | G | A | 198 | 180 | 18 | 0 | 18 | 396 | 4.55 | Pass (HWE p>=0.05) | Multiplex real-time PCR; TaqMan probe | Vietnamese patients with coronary artery disease using clopidogrel; Military Hospital 175 |
| Study_S0104_KINH_02 | S0104 | 4.0 | *CYP2C19* | *3 | rs4986893 | G | A | 100 | 95 | 5 | 0 | 5 | 200 | 2.50 | Pass (HWE p>=0.05) | Sanger sequencing of promoter and all exons; PCR-RFLP confirmation | Kinh; healthy unrelated Vietnamese subjects |
| Study_S0025_VNM_17 | S0025 | 6.0 | *CYP2C19* | *5 | rs56337013 | C | T | 100 | 99 | 1 | 0 | 1 | 200 | 0.50 | Pass (HWE p>=0.05) | Targeted panel sequencing; MiSeq; GATK variant calling | Kinh Vietnamese healthy population |
| Study_S0025_VNM_16 | S0025 | 6.0 | *CYP2C19* | rs3758581 | rs3758581 | A | G | 100 | 0 | 5 | 95 | 195 | 200 | 97.50 | Pass (HWE p>=0.05) | Targeted panel sequencing; MiSeq; GATK variant calling | Kinh Vietnamese healthy population |
| Study_S0006_VNM_01 | S0006 | 3.0 | *CYP2C8* | *11 | rs78637571 | G | T | 100 | 98 | 2 | 0 | 2 | 200 | 1.00 | Pass (HWE p>=0.05) | pyrosequencing | Vietnamese; Viet Kinh |
| Study_S0025_VNM_18 | S0025 | 6.0 | *CYP2C9* | *13 | rs72558187 | T | C | 100 | 99 | 1 | 0 | 1 | 200 | 0.50 | Pass (HWE p>=0.05) | Targeted panel sequencing; MiSeq; GATK variant calling | Kinh Vietnamese healthy population |
| Study_S0060_02 | S0060 | 5.0 | *CYP2C9* | *13 | rs72558187 | T | C | 127 | 125 | 2 | 0 | 2 | 254 | 0.79 | Pass (HWE p>=0.05) | SNaPshot; multiplex single-base extension | Vietnamese-Korean; offspring of Vietnamese mother and Korean father |
| Study_S0004_01 | S0004 | 2.0 | *CYP2C9* | *2 | rs1799853 | C | T | 157 | 157 | 0 | 0 | 0 | 314 | 0.00 | Not informative | PCR; pyrosequencing | Kinh; Vietnamese |
| Study_S0011_VNM_KINH_01 | S0011 | 4.0 | *CYP2C9* | *2 | rs1799853 | C | T | 100 | 100 | 0 | 0 | 0 | 200 | 0.00 | Not informative | Sequencing; RFLP-PCR | Kinh Vietnamese |
| Study_S0033_VNM_02 | S0033 | 77.0 | *CYP2C9* | *2 | rs1799853 | C | T | 92 | 92 | 0 | 0 | 0 | 184 | 0.00 | Not informative | Real-time PCR; TaqMan/SNP-Screen | Vietnamese epilepsy patients using valproate and/or phenytoin |
| Study_S0004_02 | S0004 | 2.0 | *CYP2C9* | *3 | rs1057910 | A | C | 157 | 150 | 7 | 0 | 7 | 314 | 2.23 | Pass (HWE p>=0.05) | PCR; pyrosequencing | Kinh; Vietnamese |
| Study_S0011_VNM_KINH_02 | S0011 | 4.0 | *CYP2C9* | *3 | rs1057910 | A | C | 100 | 93 | 7 | 0 | 7 | 200 | 3.50 | Pass (HWE p>=0.05) | Sequencing; RFLP-PCR | Kinh Vietnamese |
| Study_S0012_VNM_01 | S0012 | 5.0 | *CYP2C9* | *3 | rs1057910 | A | C | 139 | 128 | 10 | 1 | 12 | 278 | 4.32 | Pass (HWE p>=0.05) | PCR; Sanger sequencing | Vietnamese gout patients; Northeast Vietnam |
| Study_S0025_VNM_19 | S0025 | 6.0 | *CYP2C9* | *3 | rs1057910 | A | C | 100 | 95 | 5 | 0 | 5 | 200 | 2.50 | Pass (HWE p>=0.05) | Targeted panel sequencing; MiSeq; GATK variant calling | Kinh Vietnamese healthy population |
| Study_S0033_VNM_03 | S0033 | 77.0 | *CYP2C9* | *3 | rs1057910 | A | C | 92 | 86 | 6 | 0 | 6 | 184 | 3.26 | Pass (HWE p>=0.05) | Real-time PCR; TaqMan/SNP-Screen | Vietnamese epilepsy patients using valproate and/or phenytoin |
| Study_S0043_VNM_01 | S0043 | 4.0 | *CYP2C9* | *3 | rs1057910 | A | C | 150 | 143 | 7 | 0 | 7 | 300 | 2.33 | Pass (HWE p>=0.05) | PCR; Sanger sequencing | Vietnamese heart valve replacement patients; Hanoi Heart Hospital |
| Study_S0010_VNM_03 | S0010 | 4.0 | *CYP2C9* | Del | — | WT | DEL | 154 | 151 | 3 | 0 | 3 | 308 | 0.97 | Pass (HWE p>=0.05) | MLPA; qPCR confirmation | Kinh Vietnamese |
| Study_S0053_04 | S0053 | 5.0 | *CYP2D6* | *10 | — | NON-*10 | *10 | 136 | 48 | 57 | 31 | 119 | 272 | 43.75 | Pass (HWE p>=0.05) | Sanger sequencing; MLPA; LR-PCR | Kinh; Vietnamese |
| Study_S0014_VNM_09 | S0014 | 5.0 | *CYP2D6* | rs1065852 C>T (source labelled CYP2D6*10) | rs1065852 | C | T | 71 | 17 | 44 | 10 | 64 | 142 | 45.07 | Warning (HWE p<0.05) | pyrosequencing | Kinh Vietnamese |
| Study_S0060_08 | S0060 | 5.0 | *CYP2D6* | *10B | — | NON-*10B | *10B | 126 | 27 | 57 | 42 | 141 | 252 | 55.95 | Pass (HWE p>=0.05) | SNaPshot; multiplex single-base extension | Vietnamese-Korean; offspring of Vietnamese mother and Korean father |
| Study_S0060_14 | S0060 | 5.0 | *CYP2D6* | *10BX2 | — | NON-*10BX2 | *10BX2 | 126 | 125 | 1 | 0 | 1 | 252 | 0.40 | Pass (HWE p>=0.05) | SNaPshot; multiplex single-base extension | Vietnamese-Korean; offspring of Vietnamese mother and Korean father |
| Study_S0053_05 | S0053 | 5.0 | *CYP2D6* | *13 | — | NON-*13 | *13 | 136 | 134 | 2 | 0 | 2 | 272 | 0.74 | Pass (HWE p>=0.05) | Sanger sequencing; MLPA; LR-PCR | Kinh; Vietnamese |
| Study_S0053_06 | S0053 | 5.0 | *CYP2D6* | *14 | — | NON-*14 | *14 | 136 | 135 | 1 | 0 | 1 | 272 | 0.37 | Pass (HWE p>=0.05) | Sanger sequencing; MLPA; LR-PCR | Kinh; Vietnamese |
| Study_S0060_09 | S0060 | 6.0 | *CYP2D6* | *14B | — | NON-*14B | *14B | 126 | 124 | 2 | 0 | 2 | 252 | 0.79 | Pass (HWE p>=0.05) | SNaPshot; multiplex single-base extension | Vietnamese-Korean; offspring of Vietnamese mother and Korean father |
| Study_S0053_07 | S0053 | 5.0 | *CYP2D6* | *15 | — | NON-*15 | *15 | 136 | 135 | 1 | 0 | 1 | 272 | 0.37 | Pass (HWE p>=0.05) | Sanger sequencing; MLPA; LR-PCR | Kinh; Vietnamese |
| Study_S0060_13 | S0060 | 5.0 | *CYP2D6* | *1XN | — | NON-*1XN | *1XN | 126 | 125 | 1 | 0 | 1 | 252 | 0.40 | Pass (HWE p>=0.05) | SNaPshot; multiplex single-base extension | Vietnamese-Korean; offspring of Vietnamese mother and Korean father |
| Study_S0060_06 | S0060 | 5.0 | *CYP2D6* | *2 | — | NON-*2 | *2 | 126 | 106 | 20 | 0 | 20 | 252 | 7.94 | Pass (HWE p>=0.05) | SNaPshot; multiplex single-base extension | Vietnamese-Korean; offspring of Vietnamese mother and Korean father |
| Study_S0053_01 | S0053 | 5.0 | *CYP2D6* | *2 | — | NON-*2 | *2 | 136 | 115 | 21 | 0 | 21 | 272 | 7.72 | Pass (HWE p>=0.05) | Sanger sequencing; MLPA; LR-PCR | Kinh; Vietnamese |
| Study_S0060_10 | S0060 | 5.0 | *CYP2D6* | *21B | — | NON-*21B | *21B | 126 | 125 | 1 | 0 | 1 | 252 | 0.40 | Pass (HWE p>=0.05) | SNaPshot; multiplex single-base extension | Vietnamese-Korean; offspring of Vietnamese mother and Korean father |
| Study_S0053_08 | S0053 | 5.0 | *CYP2D6* | *36 | — | NON-*36 | *36 | 136 | 135 | 1 | 0 | 1 | 272 | 0.37 | Pass (HWE p>=0.05) | Sanger sequencing; MLPA; LR-PCR | Kinh; Vietnamese |
| Study_S0053_09 | S0053 | 5.0 | *CYP2D6* | *36-*10 | — | NON-*36-*10 | *36-*10 | 136 | 110 | 21 | 5 | 31 | 272 | 11.40 | Warning (HWE p<0.05) | Sanger sequencing; MLPA; LR-PCR | Kinh; Vietnamese |
| Study_S0053_10 | S0053 | 5.0 | *CYP2D6* | *36-*36-*10 | — | NON-*36-*36-*10 | *36-*36-*10 | 136 | 135 | 1 | 0 | 1 | 272 | 0.37 | Pass (HWE p>=0.05) | Sanger sequencing; MLPA; LR-PCR | Kinh; Vietnamese |
| Study_S0053_02 | S0053 | 5.0 | *CYP2D6* | *4 | — | NON-*4 | *4 | 136 | 134 | 2 | 0 | 2 | 272 | 0.74 | Pass (HWE p>=0.05) | Sanger sequencing; MLPA; LR-PCR | Kinh; Vietnamese |
| Study_S0014_VNM_10 | S0014 | 5.0 | *CYP2D6* | rs3892097 G>A (source labelled CYP2D6*4) | rs3892097 | G | A | 69 | 67 | 2 | 0 | 2 | 138 | 1.45 | Pass (HWE p>=0.05) | PCR-RFLP | Kinh Vietnamese |
| Study_S0060_11 | S0060 | 5.0 | *CYP2D6* | *41 | — | NON-*41 | *41 | 126 | 115 | 11 | 0 | 11 | 252 | 4.37 | Pass (HWE p>=0.05) | SNaPshot; multiplex single-base extension | Vietnamese-Korean; offspring of Vietnamese mother and Korean father |
| Study_S0060_12 | S0060 | 5.0 | *CYP2D6* | *49 | — | NON-*49 | *49 | 126 | 123 | 3 | 0 | 3 | 252 | 1.19 | Pass (HWE p>=0.05) | SNaPshot; multiplex single-base extension | Vietnamese-Korean; offspring of Vietnamese mother and Korean father |
| Study_S0014_VNM_08 | S0014 | 5.0 | *CYP2D6* | *5 | — | NON-*5 | *5 | 71 | 61 | 9 | 1 | 11 | 142 | 7.75 | Pass (HWE p>=0.05) | PCR | Kinh Vietnamese |
| Study_S0053_03 | S0053 | 5.0 | *CYP2D6* | *5 | — | NON-*5 | *5 | 136 | 114 | 22 | 0 | 22 | 272 | 8.09 | Pass (HWE p>=0.05) | Sanger sequencing; MLPA; LR-PCR | Kinh; Vietnamese |
| Study_S0060_07 | S0060 | 5.0 | *CYP2D6* | *5 | — | NON-*5 | *5 | 126 | 109 | 17 | 0 | 17 | 252 | 6.75 | Pass (HWE p>=0.05) | SNaPshot; multiplex single-base extension | Vietnamese-Korean; offspring of Vietnamese mother and Korean father |
| Study_S0053_11 | S0053 | 5.0 | *CYP2D6* | *60 | — | NON-*60 | *60 | 136 | 135 | 0 | 1 | 2 | 272 | 0.74 | Fail (HWE p<0.001) | Sanger sequencing; MLPA; LR-PCR | Kinh; Vietnamese |
| Study_S0053_12 | S0053 | 5.0 | *CYP2D6* | *65 | — | NON-*65 | *65 | 136 | 128 | 8 | 0 | 8 | 272 | 2.94 | Pass (HWE p>=0.05) | Sanger sequencing; MLPA; LR-PCR | Kinh; Vietnamese |
| Study_S0053_13 | S0053 | 5.0 | *CYP2D6* | *67 | — | NON-*67 | *67 | 136 | 135 | 1 | 0 | 1 | 272 | 0.37 | Pass (HWE p>=0.05) | Sanger sequencing; MLPA; LR-PCR | Kinh; Vietnamese |
| Study_S0053_14 | S0053 | 5.0 | *CYP2D6* | *68-*4 | — | NON-*68-*4 | *68-*4 | 136 | 135 | 1 | 0 | 1 | 272 | 0.37 | Pass (HWE p>=0.05) | Sanger sequencing; MLPA; LR-PCR | Kinh; Vietnamese |
| Study_S0053_15 | S0053 | 5.0 | *CYP2D6* | *86 | — | NON-*86 | *86 | 136 | 135 | 1 | 0 | 1 | 272 | 0.37 | Pass (HWE p>=0.05) | Sanger sequencing; MLPA; LR-PCR | Kinh; Vietnamese |
| Study_S0016_VNM_CTRL_01 | S0016 | 3.0 | *CYP2D6* | G4268C | rs1135840 | G | C | 200 | 28 | 51 | 121 | 293 | 400 | 73.25 | Fail (HWE p<0.001) | PCR-RFLP; sequencing confirmation | Vietnamese controls |
| Study_S0078_01 | S0078 | 5.0 | *CYP2E1* | *5B | rs2031920 | C | T | 96 | 65 | 29 | 2 | 33 | 192 | 17.19 | Pass (HWE p>=0.05) | PCR; Sanger sequencing; nucleotide sequence analysis | Vietnamese living in northern provinces; patients/individuals attending Thai Nguyen National Hospital |
| Study_S0078_02 | S0078 | 5.0 | *CYP2E1* | *7B | rs6413420 | G | T | 96 | 96 | 0 | 0 | 0 | 192 | 0.00 | Not informative | PCR; Sanger sequencing; nucleotide sequence analysis | Vietnamese living in northern provinces; patients/individuals attending Thai Nguyen National Hospital |
| Study_S0010_VNM_04 | S0010 | 4.0 | *CYP2E1* | Del | — | WT | DEL | 154 | 153 | 1 | 0 | 1 | 308 | 0.32 | Pass (HWE p>=0.05) | MLPA; qPCR confirmation | Kinh Vietnamese |
| Study_S0010_VNM_05 | S0010 | 4.0 | *CYP2E1* | Dup | — | WT | DUP | 154 | 151 | 3 | 0 | 3 | 308 | 0.97 | Pass (HWE p>=0.05) | MLPA; qPCR confirmation | Kinh Vietnamese |
| Study_S0064_01 | S0064 | 8.0 | *CYP2J2* | *8 | — | G312 | R312 | 159 | 159 | 0 | 0 | 0 | 318 | 0.00 | Not informative | PCR-RFLP | Viet Kinh; Vietnamese |
| Study_S0064_02 | S0064 | 8.0 | *CYP2J2* | *9 | — | P351 | L351 | 159 | 159 | 0 | 0 | 0 | 318 | 0.00 | Not informative | PCR-RFLP | Viet Kinh; Vietnamese |
| Study_S0060_15 | S0060 | 6.0 | *CYP3A4* | *18 | — | NON-*18 | *18 | 127 | 125 | 2 | 0 | 2 | 254 | 0.79 | Pass (HWE p>=0.05) | SNaPshot; multiplex single-base extension | Vietnamese-Korean; offspring of Vietnamese mother and Korean father |
| Study_S0014_VNM_11 | S0014 | 5.0 | *CYP3A4* | *1B | rs2740574 | A | G | 72 | 69 | 3 | 0 | 3 | 144 | 2.08 | Pass (HWE p>=0.05) | PCR-RFLP | Kinh Vietnamese |
| Study_S0008_01 | S0008 | 4.0 | *CYP3A5* | *3 | rs776746 | A | G | 68 | 4 | 16 | 48 | 112 | 136 | 82.35 | Pass (HWE p>=0.05) | PCR-RFLP; direct sequencing | Vietnamese, renal transplant recipients |
| Study_S0014_VNM_12 | S0014 | 5.0 | *CYP3A5* | *3 | rs776746 | A | G | 72 | 7 | 34 | 31 | 96 | 144 | 66.67 | Pass (HWE p>=0.05) | TaqMan; PCR-RFLP | Kinh Vietnamese |
| Study_S0033_VNM_01 | S0033 | 74.0 | *CYP3A5* | *3 | rs776746 | A | G | 64 | 36 | 19 | 9 | 37 | 128 | 28.91 | Warning (HWE p<0.05) | Real-time PCR; TaqMan/SNP-Screen | Vietnamese epilepsy patients using carbamazepine |
| Study_S0036_VNM_01 | S0036 | 4.0 | *CYP3A5* | *3 | rs776746 | A | G | 88 | 9 | 41 | 38 | 117 | 176 | 66.48 | Pass (HWE p>=0.05) | PCR-RFLP; Sanger sequencing confirmation | Vietnamese renal transplant recipients; Military Hospital 103 |
| Study_S0044_VNM_01 | S0044 | 3.0 | *CYP3A5* | *3 | rs776746 | A | G | 38 | 23 | 9 | 6 | 21 | 76 | 27.63 | Warning (HWE p<0.05) | PCR-RFLP; sequencing confirmation | Vietnamese epilepsy patients treated with carbamazepine monotherapy; Nhan Dan Gia Dinh Hospital |
| Study_S0060_16 | S0060 | 6.0 | *CYP3A5* | *3 | rs776746 | A | G | 127 | 5 | 45 | 77 | 199 | 254 | 78.35 | Pass (HWE p>=0.05) | SNaPshot; multiplex single-base extension | Vietnamese-Korean; offspring of Vietnamese mother and Korean father |
| Study_S0089_01 | S0089 | 4.0 | *CYP3A5* | *3 | rs776746 | A | G | 86 | 9 | 40 | 37 | 114 | 172 | 66.28 | Pass (HWE p>=0.05) | PCR-RFLP; subset confirmed by DNA sequencing | Vietnamese kidney transplant recipients; Military Hospital 103, Hanoi |
| Study_S0014_VNM_13 | S0014 | 5.0 | *CYP3A5* | *6 | rs10264272 | G | A | 72 | 72 | 0 | 0 | 0 | 144 | 0.00 | Not informative | PCR-RFLP | Kinh Vietnamese |
| Study_S0014_VNM_14 | S0014 | 5.0 | *CYP3A5* | *7 | rs41303343 | T | TT | 72 | 72 | 0 | 0 | 0 | 144 | 0.00 | Not informative | PCR-RFLP | Kinh Vietnamese |
| Study_S0014_VNM_15 | S0014 | 5.0 | *CYP3A5* | *8 | rs55817950 | C | T | 72 | 72 | 0 | 0 | 0 | 144 | 0.00 | Not informative | PCR-RFLP | Kinh Vietnamese |
| Study_S0025_VNM_23 | S0025 | 7.0 | *CYP4F2* | rs2108622 | rs2108622 | C | T | 100 | 70 | 27 | 3 | 33 | 200 | 16.50 | Pass (HWE p>=0.05) | Targeted panel sequencing; MiSeq; GATK variant calling | Kinh Vietnamese healthy population |
| Study_S0025_VNM_02 | S0025 | 6.0 | *DPYD* | rs17376848 | rs17376848 | A | G | 100 | 71 | 26 | 3 | 32 | 200 | 16.00 | Pass (HWE p>=0.05) | Targeted panel sequencing; MiSeq; GATK variant calling | Kinh Vietnamese healthy population |
| Study_S0025_VNM_03 | S0025 | 6.0 | *DPYD* | rs1801159 | rs1801159 | T | C | 100 | 60 | 35 | 5 | 45 | 200 | 22.50 | Pass (HWE p>=0.05) | Targeted panel sequencing; MiSeq; GATK variant calling | Kinh Vietnamese healthy population |
| Study_S0025_VNM_01 | S0025 | 6.0 | *DPYD* | rs1801160 | rs1801160 | C | T | 100 | 96 | 4 | 0 | 4 | 200 | 2.00 | Pass (HWE p>=0.05) | Targeted panel sequencing; MiSeq; GATK variant calling | Kinh Vietnamese healthy population |
| Study_S0025_VNM_05 | S0025 | 6.0 | *DPYD* | rs1801265 | rs1801265 | A | G | 100 | 69 | 28 | 3 | 34 | 200 | 17.00 | Pass (HWE p>=0.05) | Targeted panel sequencing; MiSeq; GATK variant calling | Kinh Vietnamese healthy population |
| Study_S0025_VNM_04 | S0025 | 6.0 | *DPYD* | rs2297595 | rs2297595 | T | C | 100 | 95 | 5 | 0 | 5 | 200 | 2.50 | Pass (HWE p>=0.05) | Targeted panel sequencing; MiSeq; GATK variant calling | Kinh Vietnamese healthy population |
| Study_S0019_VNM_01 | S0019 | 7.0 | *HLA-B* | *58:01 | — | *X | *58:01 | 133 | 117 | 16 | 0 | 16 | 266 | 6.02 | Pass (HWE p>=0.05) | PCR; Sanger sequencing | Vietnamese gout patients; Northeast Vietnam |
| Study_S0065_01 | S0065 | 6.0 | *HNF4A* | G36S | — | G36 | S36 | 139 | 129 | 10 | 0 | 10 | 278 | 3.60 | Pass (HWE p>=0.05) | duplex pyrosequencing | Viet Kinh; Vietnamese |
| Study_S0065_02 | S0065 | 6.0 | *HNF4A* | G60D | — | G60 | D60 | 139 | 137 | 2 | 0 | 2 | 278 | 0.72 | Pass (HWE p>=0.05) | duplex pyrosequencing | Viet Kinh; Vietnamese |
| Study_S0097_05 | S0097 | 2.0 | *NAT2* | *12 | — | NON-*12 | *12 | 72 | 67 | 5 | 0 | 5 | 144 | 3.47 | Pass (HWE p>=0.05) | full NAT2 gene sequencing | Mostly Kinh Vietnamese adults; National Institute of Malariology, Parasitology and Entomology, Hanoi |
| Study_S0097_04 | S0097 | 2.0 | *NAT2* | *13 | — | NON-*13 | *13 | 72 | 33 | 34 | 5 | 44 | 144 | 30.56 | Pass (HWE p>=0.05) | full NAT2 gene sequencing | Mostly Kinh Vietnamese adults; National Institute of Malariology, Parasitology and Entomology, Hanoi |
| Study_S0097_06 | S0097 | 2.0 | *NAT2* | *19 | — | NON-*19 | *19 | 72 | 70 | 2 | 0 | 2 | 144 | 1.39 | Pass (HWE p>=0.05) | full NAT2 gene sequencing | Mostly Kinh Vietnamese adults; National Institute of Malariology, Parasitology and Entomology, Hanoi |
| Study_S0097_01 | S0097 | 2.0 | *NAT2* | *5 | — | NON-*5 | *5 | 72 | 68 | 4 | 0 | 4 | 144 | 2.78 | Pass (HWE p>=0.05) | full NAT2 gene sequencing | Mostly Kinh Vietnamese adults; National Institute of Malariology, Parasitology and Entomology, Hanoi |
| Study_S0009_01 | S0009 | 4.0 | *NAT2* | *5 | rs1801280 | T | C | 125 | 110 | 14 | 1 | 16 | 250 | 6.40 | Pass (HWE p>=0.05) | PCR-RFLP; Sanger sequencing | Vietnamese pulmonary tuberculosis patients |
| Study_S0025_VNM_09 | S0025 | 6.0 | *NAT2* | *5 | rs1801280 | T | C | 100 | 85 | 14 | 1 | 16 | 200 | 8.00 | Pass (HWE p>=0.05) | Targeted panel sequencing; MiSeq; GATK variant calling | Kinh Vietnamese healthy population |
| Study_S0067_01 | S0067 | 5.0 | *NAT2* | *5 | rs1801280 | T | C | 132 | 115 | 16 | 1 | 18 | 264 | 6.82 | Pass (HWE p>=0.05) | PCR-RFLP; Sanger sequencing | Vietnamese pulmonary tuberculosis patients |
| Study_S0092_01 | S0092 | 3.0 | *NAT2* | *5 | rs1801280 | T | C | 100 | 96 | 4 | 0 | 4 | 200 | 2.00 | Pass (HWE p>=0.05) | PCR-RFLP; KpnI restriction enzyme | Vietnamese subjects; Hanoi |
| Study_S0009_02 | S0009 | 4.0 | *NAT2* | *6 | rs1799930 | G | A | 125 | 62 | 48 | 15 | 78 | 250 | 31.20 | Pass (HWE p>=0.05) | PCR-RFLP; Sanger sequencing | Vietnamese pulmonary tuberculosis patients |
| Study_S0025_VNM_10 | S0025 | 6.0 | *NAT2* | *6 | rs1799930 | G | A | 100 | 41 | 46 | 13 | 72 | 200 | 36.00 | Pass (HWE p>=0.05) | Targeted panel sequencing; MiSeq; GATK variant calling | Kinh Vietnamese healthy population |
| Study_S0067_02 | S0067 | 5.0 | *NAT2* | *6 | rs1799930 | G | A | 132 | 65 | 50 | 17 | 84 | 264 | 31.82 | Pass (HWE p>=0.05) | PCR-RFLP; Sanger sequencing | Vietnamese pulmonary tuberculosis patients |
| Study_S0092_02 | S0092 | 4.0 | *NAT2* | *6 | rs1799930 | G | A | 100 | 79 | 17 | 4 | 25 | 200 | 12.50 | Warning (HWE p<0.05) | PCR-RFLP; TaqI restriction enzyme | Vietnamese subjects; Hanoi |
| Study_S0097_02 | S0097 | 2.0 | *NAT2* | *6A | — | NON-*6A | *6A | 72 | 54 | 11 | 7 | 25 | 144 | 17.36 | Fail (HWE p<0.001) | full NAT2 gene sequencing | Mostly Kinh Vietnamese adults; National Institute of Malariology, Parasitology and Entomology, Hanoi |
| Study_S0097_03 | S0097 | 2.0 | *NAT2* | *7 | — | NON-*7 | *7 | 72 | 72 | 0 | 0 | 0 | 144 | 0.00 | Not informative | full NAT2 gene sequencing | Mostly Kinh Vietnamese adults; National Institute of Malariology, Parasitology and Entomology, Hanoi |
| Study_S0009_03 | S0009 | 4.0 | *NAT2* | *7 | rs1799931 | G | A | 125 | 90 | 30 | 5 | 40 | 250 | 16.00 | Pass (HWE p>=0.05) | PCR-RFLP; Sanger sequencing | Vietnamese pulmonary tuberculosis patients |
| Study_S0025_VNM_12 | S0025 | 6.0 | *NAT2* | *7 | rs1799931 | G | A | 100 | 73 | 25 | 2 | 29 | 200 | 14.50 | Pass (HWE p>=0.05) | Targeted panel sequencing; MiSeq; GATK variant calling | Kinh Vietnamese healthy population |
| Study_S0067_03 | S0067 | 5.0 | *NAT2* | *7 | rs1799931 | G | A | 132 | 96 | 30 | 6 | 42 | 264 | 15.91 | Pass (HWE p>=0.05) | PCR-RFLP; Sanger sequencing | Vietnamese pulmonary tuberculosis patients |
| Study_S0092_03 | S0092 | 5.0 | *NAT2* | *7 | rs1799931 | G | A | 100 | 54 | 41 | 5 | 51 | 200 | 25.50 | Pass (HWE p>=0.05) | PCR-RFLP; BamHI restriction enzyme | Vietnamese subjects; Hanoi |
| Study_S0025_VNM_11 | S0025 | 6.0 | *NAT2* | rs1208 | rs1208 | G | A | 100 | 1 | 16 | 83 | 182 | 200 | 91.00 | Pass (HWE p>=0.05) | Targeted panel sequencing; MiSeq; GATK variant calling | Kinh Vietnamese healthy population |
| Study_S0025_VNM_22 | S0025 | 7.0 | *NUDT15* | *2;*3 | rs116855232 | C | T | 100 | 91 | 9 | 0 | 9 | 200 | 4.50 | Pass (HWE p>=0.05) | Targeted panel sequencing; MiSeq; GATK variant calling | Kinh Vietnamese healthy population |
| Study_S0098_03 | S0098 | 3.0 | *PON1* | Q192R | rs662 | C | T | 198 | 90 | 85 | 23 | 131 | 396 | 33.08 | Pass (HWE p>=0.05) | real-time PCR; TaqMan probe | Vietnamese patients with coronary artery disease using clopidogrel; Military Hospital 175 |
| Study_S0019_VNM_02 | S0019 | 7.0 | *PSORS1C1* | rs9263726 | rs9263726 | G | A | 133 | 112 | 21 | 0 | 21 | 266 | 7.89 | Pass (HWE p>=0.05) | PCR; Sanger sequencing | Vietnamese gout patients; Northeast Vietnam |
| Study_S0073_03 | S0073 | 4.0 | *SLCO1B1* | *15 | rs4149056 | T | C | 104 | 74 | 26 | 4 | 34 | 208 | 16.35 | Pass (HWE p>=0.05) | duplex pyrosequencing | Viet Kinh; healthy Vietnamese subjects; native residents of Hanoi |
| Study_S0073_02 | S0073 | 4.0 | *SLCO1B1* | *5 | rs4149056 | T | C | 104 | 104 | 0 | 0 | 0 | 208 | 0.00 | Not informative | duplex pyrosequencing | Viet Kinh; healthy Vietnamese subjects; native residents of Hanoi |
| Study_S0073_01 | S0073 | 4.0 | *SLCO1B1* | c.388A>G | rs2306283 | A | G | 104 | 6 | 32 | 66 | 164 | 208 | 78.85 | Pass (HWE p>=0.05) | duplex pyrosequencing | Viet Kinh; healthy Vietnamese subjects; native residents of Hanoi |
| Study_S0025_VNM_21 | S0025 | 7.0 | *SLCO1B1* | c.521T>C | rs4149056 | T | C | 100 | 82 | 17 | 1 | 19 | 200 | 9.50 | Pass (HWE p>=0.05) | Targeted panel sequencing; MiSeq; GATK variant calling | Kinh Vietnamese healthy population |
| Study_S0073_04 | S0073 | 4.0 | *SLCO1B1* | c.521T>C | rs4149056 | T | C | 104 | 74 | 26 | 4 | 34 | 208 | 16.35 | Pass (HWE p>=0.05) | duplex pyrosequencing | Viet Kinh; healthy Vietnamese subjects; native residents of Hanoi |
| Study_S0025_VNM_20 | S0025 | 7.0 | *SLCO1B1* | rs2306283 | rs2306283 | A | G | 100 | 7 | 40 | 53 | 146 | 200 | 73.00 | Pass (HWE p>=0.05) | Targeted panel sequencing; MiSeq; GATK variant calling | Kinh Vietnamese healthy population |
| Study_S0025_VNM_08 | S0025 | 6.0 | *TPMT* | rs1142345 | rs1142345 | T | C | 100 | 95 | 5 | 0 | 5 | 200 | 2.50 | Pass (HWE p>=0.05) | Targeted panel sequencing; MiSeq; GATK variant calling | Kinh Vietnamese healthy population |
| Study_S0049_VNM_CTRL_03 | S0049 | 3.0 | *UGT1A1* | *28 | rs8175347 | TA6 | TA7 | 50 | 45 | 4 | 1 | 6 | 100 | 6.00 | Warning (HWE p<0.05) | PCR; direct sequencing | Vietnamese full-term newborn controls; Binh Thuan General Hospital |
| Study_S0087_01 | S0087 | 5.0 | *UGT1A1* | *28 | rs8175347 | TA6 | TA7 | 95 | 80 | 14 | 1 | 16 | 190 | 8.42 | Pass (HWE p>=0.05) | PCR; direct sequencing of UGT1A1 promoter | Kinh; healthy Vietnamese |
| Study_S0025_VNM_06 | S0025 | 6.0 | *UGT1A1* | *6 | rs4148323 | G | A | 100 | 76 | 22 | 2 | 26 | 200 | 13.00 | Pass (HWE p>=0.05) | Targeted panel sequencing; MiSeq; GATK variant calling | Kinh Vietnamese healthy population |
| Study_S0049_VNM_CTRL_01 | S0049 | 3.0 | *UGT1A1* | *6 | rs4148323 | G | A | 50 | 45 | 5 | 0 | 5 | 100 | 5.00 | Pass (HWE p>=0.05) | PCR; direct sequencing | Vietnamese full-term newborn controls; Binh Thuan General Hospital |
| Study_S0049_VNM_CTRL_02 | S0049 | 3.0 | *UGT1A1* | c.189C>T | rs191471887 | C | T | 50 | 50 | 0 | 0 | 0 | 100 | 0.00 | Not informative | PCR; direct sequencing | Vietnamese full-term newborn controls; Binh Thuan General Hospital |
| Study_S0015_VNM_CTRL_02 | S0015 | 4.0 | *UGT1A1* | rs1042640C>G | rs1042640 | C | G | 100 | 58 | 34 | 8 | 50 | 200 | 25.00 | Pass (HWE p>=0.05) | PCR; Sanger sequencing | Kinh Vietnamese; healthy controls |
| Study_S0015_VNM_CTRL_01 | S0015 | 4.0 | *UGT1A1* | rs10929303C>T | rs10929303 | C | T | 100 | 58 | 34 | 8 | 50 | 200 | 25.00 | Pass (HWE p>=0.05) | PCR; Sanger sequencing | Kinh Vietnamese; healthy controls |
| Study_S0015_VNM_CTRL_03 | S0015 | 4.0 | *UGT1A1* | rs8330C>G | rs8330 | C | G | 100 | 58 | 34 | 8 | 50 | 200 | 25.00 | Pass (HWE p>=0.05) | PCR; Sanger sequencing | Kinh Vietnamese; healthy controls |
| Study_S0043_VNM_02 | S0043 | 4.0 | *VKORC1* | -1173C>T | rs9934438 | C | T | 150 | 0 | 44 | 106 | 256 | 300 | 85.33 | Warning (HWE p<0.05) | PCR; Sanger sequencing | Vietnamese heart valve replacement patients; Hanoi Heart Hospital |
| Study_S0043_VNM_03 | S0043 | 4.0 | *VKORC1* | -1639G>A | rs9923231 | G | A | 150 | 3 | 16 | 131 | 278 | 300 | 92.67 | Warning (HWE p<0.05) | PCR; Sanger sequencing | Vietnamese heart valve replacement patients; Hanoi Heart Hospital |
| Study_S0066_01 | S0066 | 5.0 | *VKORC1* | -1639G>A | rs9923231 | G | A | 49 | 0 | 14 | 35 | 84 | 98 | 85.71 | Pass (HWE p>=0.05) | Warfarin Dosage Prediction Kit; Sequenom MassARRAY | Vietnamese currently residing in Taiwan |
| Study_S0066_03 | S0066 | 5.0 | *VKORC1* | 1173C>T | rs9934438 | C | T | 49 | 0 | 14 | 35 | 84 | 98 | 85.71 | Pass (HWE p>=0.05) | Sequenom MassARRAY | Vietnamese currently residing in Taiwan |
| Study_S0066_04 | S0066 | 5.0 | *VKORC1* | 1542G>C | rs8050894 | G | C | 49 | 0 | 14 | 35 | 84 | 98 | 85.71 | Pass (HWE p>=0.05) | Sequenom MassARRAY | Vietnamese currently residing in Taiwan |
| Study_S0066_05 | S0066 | 5.0 | *VKORC1* | 2255C>T | rs2359612 | C | T | 49 | 0 | 14 | 35 | 84 | 98 | 85.71 | Pass (HWE p>=0.05) | Sequenom MassARRAY | Vietnamese currently residing in Taiwan |
| Study_S0066_06 | S0066 | 5.0 | *VKORC1* | 3730G>A | rs7294 | G | A | 49 | 35 | 14 | 0 | 14 | 98 | 14.29 | Pass (HWE p>=0.05) | Sequenom MassARRAY | Vietnamese currently residing in Taiwan |
| Study_S0066_02 | S0066 | 5.0 | *VKORC1* | 497T>G | rs2884737 | T | G | 49 | 48 | 1 | 0 | 1 | 98 | 1.02 | Pass (HWE p>=0.05) | Sequenom MassARRAY | Vietnamese currently residing in Taiwan |

Abbreviations: AF, allele frequency; HWE, Hardy–Weinberg equilibrium; N, participants successfully genotyped for the row; n, genotype or allele-state count. A dash indicates that the field was not applicable or not reconstructable from the source.
