# Supplementary material for: Vietnamese pharmacogenomic variation in global context: a systematic review and meta-analysis for clinical implementation
Source: Front Pharmacol. 2026 Sep 3;17:1921144. doi: 10.3389/fphar.2026.1921144 (PMC13582518; doi:10.3389/fphar.2026.1921144)
Supplement: Supplementary file 3 [file Table3.docx]

**Supplementary Table S3. Full Vietnamese pharmacogenetic allele-frequency estimates across 108 source-level variant representations**

For representations with k ≥2 independent rows, the preferred estimate is a DerSimonian–Laird random-effects logit model with a back-transformed 95% confidence interval. For k=1, the observed frequency and Wilson 95% confidence interval are reported. Ninety-five of 108 representations are supported by one study row.

Main-text estimates combine only verified synonymous source labels sharing an rsID and effect-allele direction. The corrected harmonized estimates are CYP2B6 rs3745274 G>T, 24.76% (95% CI 20.48–29.61; k=2; N=172); SLCO1B1 rs2306283 A>G, 75.95% (69.75–81.22; k=2; N=204); and VKORC1 rs9934438 C>T, 85.43% (81.61–88.56; k=2; N=199). Complex alleles are not reduced to single-marker proxies. Abbreviations: CI, confidence interval; DL, DerSimonian–Laird; I², percentage of total variation attributable to between-row heterogeneity; k, contributing independent study rows; N, row-level participant sum; τ², between-row variance on the analysis scale.

| **Gene** | **Allele/variant representation** | **rsID** | **Representation key** | **Reference allele/state** | **Effect allele/state** | **Estimate, %** | **95% CI** | **k** | **Total N** | **I², %** | **τ²** | **Analysis model** | **Harmonization/interpretation note** |
| --- | --- | --- | --- | --- | --- | --- | --- | --- | --- | --- | --- | --- | --- |
| *ABCB1* | 3435C>T | rs1045642 | rs1045642 | C | T | 40.28 | 32.62–48.44 | 1 | 72 | — | — | Single-study Wilson | Source-level single-study estimate. |
| *ABCG2* | rs2231142 | rs2231142 | rs2231142 | G | T | 36.00 | 29.67–42.86 | 1 | 100 | — | — | Single-study Wilson | Source-level single-study estimate. |
| *CYP1B1* | Dup | — | CYP1B1 copy-number variation | WT | DUP | 0.97 | 0.33–2.82 | 1 | 154 | — | — | Single-study Wilson | Source-level single-study estimate. |
| *CYP2A6* | *11 | rs28399447 | rs28399447 | A | G | 1.50 | 0.51–4.32 | 1 | 100 | — | — | Single-study Wilson | Source-level single-study estimate. |
| *CYP2A6* | *15 | rs199916117 | rs199916117 | T | C | 0.50 | 0.09–2.78 | 1 | 100 | — | — | Single-study Wilson | Source-level single-study estimate. |
| *CYP2A6* | *4 | — | CYP2A6 deletion | WT | DEL | 11.81 | 7.50–18.09 | 1 | 72 | — | — | Single-study Wilson | Source-level single-study estimate. |
| *CYP2A6* | *5 | rs5031017 | rs5031017 | G | T | 14.58 | 9.74–21.27 | 1 | 72 | — | — | Single-study Wilson | Source-level single-study estimate. |
| *CYP2A6* | Del | — | CYP2A6 copy-number variation | WT | DEL | 11.04 | 8.01–15.03 | 1 | 154 | — | — | Single-study Wilson | Source-level single-study estimate. |
| *CYP2A6* | rs1809810 | rs1809810 | rs1809810 | T | A | 2.00 | 0.78–5.03 | 1 | 100 | — | — | Single-study Wilson | Source-level single-study estimate. |
| *CYP2B6* | *2 | rs8192709 | rs8192709 | C | T | 5.50 | 3.10–9.58 | 1 | 100 | — | — | Single-study Wilson | Source-level single-study estimate. |
| *CYP2B6* | *4 | rs2279343 | rs2279343 | A | G | 35.42 | 28.08–43.51 | 1 | 72 | — | — | Single-study Wilson | Source-level single-study estimate. |
| *CYP2B6* | *5 | rs3211371 | rs3211371 | C | T | 0.00 | 0.00–2.60 | 1 | 72 | — | — | Single-study Wilson | Source-level single-study estimate. |
| *CYP2B6* | 516G>T | rs3745274 | rs3745274 | G | T | 27.08 | 20.49–34.87 | 1 | 72 | — | — | Single-study Wilson | Source label harmonized with the other rs3745274 G>T representation for the main-text estimate: 24.76% (95% CI 20.48–29.61), k=2, N=172, I²=0.00%. |
| *CYP2B6* | rs3745274 | rs3745274 | rs3745274 | G | T | 23.00 | 17.71–29.31 | 1 | 100 | — | — | Single-study Wilson | Source label harmonized with the other rs3745274 G>T representation for the main-text estimate: 24.76% (95% CI 20.48–29.61), k=2, N=172, I²=0.00%. |
| *CYP2C19* | *17 | rs11188072 | rs11188072 | C | T | 1.04 | 0.29–3.72 | 1 | 96 | — | — | Single-study Wilson | Source-labelled CYP2C19*17-associated marker; not treated as equivalent to the defining rs12248560 increased-function allele. |
| *CYP2C19* | *17 | rs12248560 | rs12248560 | C | T | 1.37 | 0.76–2.46 | 3 | 422 | 0.00 | 0.0000 | Random-effects logit (DL) | Primary CYP2C19*17 representation used for phenotype interpretation and Table 4/Figure 5. |
| *CYP2C19* | *2 | rs4244285 | rs4244285 | G | A | 27.90 | 26.57–29.27 | 19 | 2,614 | 11.80 | 0.0026 | Random-effects logit (DL) | Pooled estimate across 19 independent study rows. |
| *CYP2C19* | *26 | rs375781227 | rs375781227 | G | A | 0.50 | 0.09–2.78 | 1 | 100 | — | — | Single-study Wilson | Source-level single-study estimate. |
| *CYP2C19* | *3 | rs4986893 | rs4986893 | G | A | 5.74 | 4.82–6.82 | 20 | 2,779 | 54.69 | 0.0841 | Random-effects logit (DL) | Pooled estimate across 20 independent study rows. |
| *CYP2C19* | *5 | rs56337013 | rs56337013 | C | T | 0.50 | 0.09–2.78 | 1 | 100 | — | — | Single-study Wilson | Source-level single-study estimate. |
| *CYP2C19* | rs3758581 | rs3758581 | rs3758581 | A | G | 97.50 | 94.28–98.93 | 1 | 100 | — | — | Single-study Wilson | Source-level single-study estimate. |
| *CYP2C8* | *11 | rs78637571 | rs78637571 | G | T | 1.00 | 0.27–3.57 | 1 | 100 | — | — | Single-study Wilson | Source-level single-study estimate. |
| *CYP2C9* | *13 | rs72558187 | rs72558187 | NON-*13 | *13 | 0.79 | 0.22–2.83 | 1 | 127 | — | — | Single-study Wilson | Source-level single-study estimate. |
| *CYP2C9* | *13 | rs72558187 | rs72558187 | T | C | 0.50 | 0.09–2.78 | 1 | 100 | — | — | Single-study Wilson | Source-level single-study estimate. |
| *CYP2C9* | *2 | rs1799853 | rs1799853 | C | T | 0.22 | 0.04–1.08 | 3 | 349 | 0.00 | 0.0000 | Random-effects logit (DL) | Pooled estimate across 3 independent study rows. |
| *CYP2C9* | *3 | rs1057910 | rs1057910 | A | C | 3.27 | 2.46–4.32 | 6 | 738 | 0.00 | 0.0000 | Random-effects logit (DL) | Pooled estimate across 6 independent study rows. |
| *CYP2C9* | Del | — | CYP2C9 copy-number variation | WT | DEL | 0.97 | 0.33–2.82 | 1 | 154 | — | — | Single-study Wilson | Source-level single-study estimate. |
| *CYP2D6* | *10 | — | Complex star allele/structure | NON-*10 | *10 | 43.75 | 37.98–49.69 | 1 | 136 | — | — | Single-study Wilson | Source-supported CYP2D6*10 star-allele estimate used in Table 4 and Figure 5; phenotype assignment still requires full diplotype, copy-number, and structural-variant assessment. |
| *CYP2D6* | rs1065852 C>T marker | rs1065852 | rs1065852 | C | T | 45.07 | 37.12–53.28 | 1 | 71 | — | — | Single-study Wilson | Single-marker frequency; not interpreted as the CYP2D6*10 star-allele frequency. Table 4/Figure 5 use the source-supported CYP2D6*10 estimate of 43.75% (95% CI 37.98–49.69). |
| *CYP2D6* | *10B | — | Star allele/haplotype | NON-*10B | *10B | 55.95 | 49.78–61.95 | 1 | 126 | — | — | Single-study Wilson | Source-level single-study estimate. |
| *CYP2D6* | *10BX2 | — | Copy-number gain | NON-*10BX2 | *10BX2 | 0.40 | 0.07–2.21 | 1 | 126 | — | — | Single-study Wilson | Source-level single-study estimate. |
| *CYP2D6* | *13 | — | Complex star allele/structure | NON-*13 | *13 | 0.74 | 0.20–2.64 | 1 | 136 | — | — | Single-study Wilson | Source-level single-study estimate. |
| *CYP2D6* | *14 | — | Complex star allele/structure | NON-*14 | *14 | 0.37 | 0.06–2.05 | 1 | 136 | — | — | Single-study Wilson | Source-level single-study estimate. |
| *CYP2D6* | *14B | — | Star allele/haplotype | NON-*14B | *14B | 0.79 | 0.22–2.85 | 1 | 126 | — | — | Single-study Wilson | Source-level single-study estimate. |
| *CYP2D6* | *15 | — | Complex star allele/structure | NON-*15 | *15 | 0.37 | 0.06–2.05 | 1 | 136 | — | — | Single-study Wilson | Source-level single-study estimate. |
| *CYP2D6* | *1XN | — | Copy-number gain | NON-*1XN | *1XN | 0.40 | 0.07–2.21 | 1 | 126 | — | — | Single-study Wilson | Source-level single-study estimate. |
| *CYP2D6* | *2 | — | Star allele/haplotype | NON-*2 | *2 | 7.94 | 5.20–11.94 | 1 | 126 | — | — | Single-study Wilson | Source-level single-study estimate. |
| *CYP2D6* | *2 | — | Complex star allele/structure | NON-*2 | *2 | 7.72 | 5.10–11.51 | 1 | 136 | — | — | Single-study Wilson | Source-level single-study estimate. |
| *CYP2D6* | *21B | — | Star allele/haplotype | NON-*21B | *21B | 0.40 | 0.07–2.21 | 1 | 126 | — | — | Single-study Wilson | Source-level single-study estimate. |
| *CYP2D6* | *36 | — | Complex star allele/structure | NON-*36 | *36 | 0.37 | 0.06–2.05 | 1 | 136 | — | — | Single-study Wilson | Source-level single-study estimate. |
| *CYP2D6* | *36-*10 | — | Tandem arrangement | NON-*36-*10 | *36-*10 | 11.40 | 8.15–15.72 | 1 | 136 | — | — | Single-study Wilson | Source-level single-study estimate. |
| *CYP2D6* | *36-*36-*10 | — | Tandem arrangement | NON-*36-*36-*10 | *36-*36-*10 | 0.37 | 0.06–2.05 | 1 | 136 | — | — | Single-study Wilson | Source-level single-study estimate. |
| *CYP2D6* | *4 | — | Complex star allele/structure | NON-*4 | *4 | 0.74 | 0.20–2.64 | 1 | 136 | — | — | Single-study Wilson | Source-supported CYP2D6*4 star-allele estimate used in Table 4 and Figure 5; rs3892097 alone is not used as a stand-alone proxy. |
| *CYP2D6* | rs3892097 G>A marker | rs3892097 | rs3892097 | G | A | 1.45 | 0.40–5.13 | 1 | 69 | — | — | Single-study Wilson | Single-marker frequency; not interpreted as the CYP2D6*4 star-allele frequency. Table 4/Figure 5 use the source-supported CYP2D6*4 estimate of 0.74% (95% CI 0.20–2.64). |
| *CYP2D6* | *41 | — | Star allele/haplotype | NON-*41 | *41 | 4.37 | 2.45–7.65 | 1 | 126 | — | — | Single-study Wilson | Source-level single-study estimate. |
| *CYP2D6* | *49 | — | Star allele/haplotype | NON-*49 | *49 | 1.19 | 0.41–3.44 | 1 | 126 | — | — | Single-study Wilson | Source-level single-study estimate. |
| *CYP2D6* | *5 | — | CYP2D6 deletion (source-specific) | WT | DEL | 7.75 | 4.38–13.34 | 1 | 71 | — | — | Single-study Wilson | Source-level deletion representation retained for traceability; not displayed independently in Table 4. |
| *CYP2D6* | *5 | — | Whole-gene deletion (pooled) | NON-*5 | *5 | 7.47 | 5.51–10.06 | 2 | 262 | 0.00 | 0.0000 | Random-effects logit (DL) | Primary pooled whole-gene-deletion estimate used in Table 4/Figure 5; k=2 independent rows. |
| *CYP2D6* | *60 | — | Complex star allele/structure | NON-*60 | *60 | 0.74 | 0.20–2.64 | 1 | 136 | — | — | Single-study Wilson | Source-level single-study estimate. |
| *CYP2D6* | *65 | — | Complex star allele/structure | NON-*65 | *65 | 2.94 | 1.50–5.70 | 1 | 136 | — | — | Single-study Wilson | Source-level single-study estimate. |
| *CYP2D6* | *67 | — | Complex star allele/structure | NON-*67 | *67 | 0.37 | 0.06–2.05 | 1 | 136 | — | — | Single-study Wilson | Source-level single-study estimate. |
| *CYP2D6* | *68-*4 | — | Hybrid structure | NON-*68-*4 | *68-*4 | 0.37 | 0.06–2.05 | 1 | 136 | — | — | Single-study Wilson | Source-level single-study estimate. |
| *CYP2D6* | *86 | — | Complex star allele/structure | NON-*86 | *86 | 0.37 | 0.06–2.05 | 1 | 136 | — | — | Single-study Wilson | Source-level single-study estimate. |
| *CYP2D6* | G4268C | rs1135840 | rs1135840 | G | C | 73.25 | 68.71–77.35 | 1 | 200 | — | — | Single-study Wilson | Source-level single-study estimate. |
| *CYP2E1* | *5B | rs2031920 | rs2031920 | C | T | 17.19 | 12.51–23.15 | 1 | 96 | — | — | Single-study Wilson | Source-level single-study estimate. |
| *CYP2E1* | *7B | rs6413420 | rs6413420 | G | T | 0.00 | 0.00–1.96 | 1 | 96 | — | — | Single-study Wilson | Source-level single-study estimate. |
| *CYP2E1* | Del | — | CYP2E1 copy-number variation | WT | DEL | 0.32 | 0.06–1.82 | 1 | 154 | — | — | Single-study Wilson | Source-level single-study estimate. |
| *CYP2E1* | Dup | — | CYP2E1 copy-number variation | WT | DUP | 0.97 | 0.33–2.82 | 1 | 154 | — | — | Single-study Wilson | Source-level single-study estimate. |
| *CYP2J2* | *8 | — | G312R | G312 | R312 | 0.00 | 0.00–1.19 | 1 | 159 | — | — | Single-study Wilson | Source-level single-study estimate. |
| *CYP2J2* | *9 | — | P351L | P351 | L351 | 0.00 | 0.00–1.19 | 1 | 159 | — | — | Single-study Wilson | Source-level single-study estimate. |
| *CYP3A4* | *18 | — | Star allele/haplotype | NON-*18 | *18 | 0.79 | 0.22–2.83 | 1 | 127 | — | — | Single-study Wilson | Source-level single-study estimate. |
| *CYP3A4* | *1B | rs2740574 | rs2740574 | A | G | 2.08 | 0.71–5.95 | 1 | 72 | — | — | Single-study Wilson | Source-level single-study estimate. |
| *CYP3A5* | *3 | rs776746 | rs776746 | A | G | 60.81 | 44.96–74.67 | 7 | 543 | 95.54 | 0.7136 | Random-effects logit (DL) | Pooled estimate across 7 independent study rows. |
| *CYP3A5* | *6 | rs10264272 | rs10264272 | G | A | 0.00 | 0.00–2.60 | 1 | 72 | — | — | Single-study Wilson | Source-level single-study estimate. |
| *CYP3A5* | *7 | rs41303343 | rs41303343 | T | TT | 0.00 | 0.00–2.60 | 1 | 72 | — | — | Single-study Wilson | Source-level single-study estimate. |
| *CYP3A5* | *8 | rs55817950 | rs55817950 | C | T | 0.00 | 0.00–2.60 | 1 | 72 | — | — | Single-study Wilson | Source-level single-study estimate. |
| *CYP4F2* | rs2108622 | rs2108622 | rs2108622 | C | T | 16.50 | 12.00–22.27 | 1 | 100 | — | — | Single-study Wilson | Source-level single-study estimate. |
| *DPYD* | rs17376848 | rs17376848 | rs17376848 | A | G | 16.00 | 11.57–21.71 | 1 | 100 | — | — | Single-study Wilson | Source-level single-study estimate. |
| *DPYD* | rs1801159 | rs1801159 | rs1801159 | T | C | 22.50 | 17.26–28.77 | 1 | 100 | — | — | Single-study Wilson | Source-level single-study estimate. |
| *DPYD* | rs1801160 | rs1801160 | rs1801160 | C | T | 2.00 | 0.78–5.03 | 1 | 100 | — | — | Single-study Wilson | Source-level single-study estimate. |
| *DPYD* | rs1801265 | rs1801265 | rs1801265 | A | G | 17.00 | 12.43–22.82 | 1 | 100 | — | — | Single-study Wilson | Source-level single-study estimate. |
| *DPYD* | rs2297595 | rs2297595 | rs2297595 | T | C | 2.50 | 1.07–5.72 | 1 | 100 | — | — | Single-study Wilson | Source-level single-study estimate. |
| *HLA-B* | *58:01 | — | HLA-B*58:01 | *X | *58:01 | 6.02 | 3.74–9.55 | 1 | 133 | — | — | Single-study Wilson | Source-level single-study estimate. |
| *HNF4A* | G36S | — | HNF4A G36S | G36 | S36 | 3.60 | 1.97–6.49 | 1 | 139 | — | — | Single-study Wilson | Source-level single-study estimate. |
| *HNF4A* | G60D | — | HNF4A G60D | G60 | D60 | 0.72 | 0.20–2.58 | 1 | 139 | — | — | Single-study Wilson | Source-level single-study estimate. |
| *NAT2* | *12 | — | Star allele/haplotype | NON-*12 | *12 | 3.47 | 1.49–7.87 | 1 | 72 | — | — | Single-study Wilson | Source-level single-study estimate. |
| *NAT2* | *13 | — | Star allele/haplotype | NON-*13 | *13 | 30.56 | 23.62–38.50 | 1 | 72 | — | — | Single-study Wilson | Source-level single-study estimate. |
| *NAT2* | *19 | — | Star allele/haplotype | NON-*19 | *19 | 1.39 | 0.38–4.92 | 1 | 72 | — | — | Single-study Wilson | Source-level single-study estimate. |
| *NAT2* | *5 | — | Star allele/haplotype | NON-*5 | *5 | 2.78 | 1.09–6.92 | 1 | 72 | — | — | Single-study Wilson | Source-level single-study estimate. |
| *NAT2* | *5 | rs1801280 | rs1801280 | T | C | 5.97 | 3.96–8.89 | 4 | 457 | 54.94 | 0.1023 | Random-effects logit (DL) | Pooled estimate across 4 independent study rows. |
| *NAT2* | *6 | rs1799930 | rs1799930 | G | A | 27.00 | 18.69–37.31 | 4 | 457 | 90.11 | 0.2103 | Random-effects logit (DL) | Pooled estimate across 4 independent study rows. |
| *NAT2* | *6A | — | Star allele/haplotype | NON-*6A | *6A | 17.36 | 12.04–24.37 | 1 | 72 | — | — | Single-study Wilson | Source-level single-study estimate. |
| *NAT2* | *7 | — | Star allele/haplotype | NON-*7 | *7 | 0.00 | 0.00–2.60 | 1 | 72 | — | — | Single-study Wilson | Source-level single-study estimate. |
| *NAT2* | *7 | rs1799931 | rs1799931 | G | A | 17.75 | 13.51–22.95 | 4 | 457 | 71.71 | 0.0776 | Random-effects logit (DL) | Pooled estimate across 4 independent study rows. |
| *NAT2* | rs1208 | rs1208 | rs1208 | G | A | 91.00 | 86.22–94.23 | 1 | 100 | — | — | Single-study Wilson | Source-level single-study estimate. |
| *NUDT15* | *2;*3 | rs116855232 | rs116855232 | C | T | 4.50 | 2.39–8.33 | 1 | 100 | — | — | Single-study Wilson | Source-level single-study estimate. |
| *PON1* | Q192R | rs662 | rs662 | C | T | 33.08 | 28.63–37.86 | 1 | 198 | — | — | Single-study Wilson | Source-level single-study estimate. |
| *PSORS1C1* | rs9263726 | rs9263726 | rs9263726 | G | A | 7.89 | 5.22–11.77 | 1 | 133 | — | — | Single-study Wilson | Source-level single-study estimate. |
| *SLCO1B1* | *15 | rs4149056 | rs4149056 | T | C | 16.35 | 11.94–21.97 | 1 | 104 | — | — | Single-study Wilson | Source-reported *15 haplotype/star-allele label involving rs4149056; retained for traceability and not treated as an independent c.521T>C observation. |
| *SLCO1B1* | *5 | rs4149056 | rs4149056 | T | C | 0.00 | 0.00–1.81 | 1 | 104 | — | — | Single-study Wilson | Source-reported *5 haplotype/star-allele label involving rs4149056; retained for traceability and not treated as an independent c.521T>C observation. |
| *SLCO1B1* | c.388A>G | rs2306283 | rs2306283 | A | G | 78.85 | 72.80–83.85 | 1 | 104 | — | — | Single-study Wilson | Source label harmonized with the other rs2306283 A>G representation for the main-text estimate: 75.95% (95% CI 69.75–81.22), k=2, N=204, I²=47.42%. |
| *SLCO1B1* | c.521T>C | rs4149056 | rs4149056 | T | C | 12.73 | 7.36–21.13 | 2 | 204 | 75.83 | 0.1464 | Random-effects logit (DL) | Primary c.521T>C representation. The pooled estimate from the two explicit c.521T>C rows is 12.73% (95% CI 7.36–21.13), k=2, N=204, I²=75.83%; source-reported *5/*15 labels are not double-counted. |
| *SLCO1B1* | rs2306283 | rs2306283 | rs2306283 | A | G | 73.00 | 66.46–78.68 | 1 | 100 | — | — | Single-study Wilson | Source label harmonized with the other rs2306283 A>G representation for the main-text estimate: 75.95% (95% CI 69.75–81.22), k=2, N=204, I²=47.42%. |
| *TPMT* | rs1142345 | rs1142345 | rs1142345 | T | C | 2.50 | 1.07–5.72 | 1 | 100 | — | — | Single-study Wilson | Source-level single-study estimate. |
| *UGT1A1* | *28 | rs8175347 | rs8175347 | *1 [(TA)6TAA] | *28 [(TA)7TAA] | 8.42 | 5.25–13.24 | 1 | 95 | — | — | Single-study Wilson | Primary source representation used in Table 4/Figure 5. |
| *UGT1A1* | *28 | rs8175347 | rs8175347 | TA6 | TA7 | 6.00 | 2.78–12.48 | 1 | 50 | — | — | Single-study Wilson | Additional source-level TA-repeat representation retained for traceability; not displayed independently in Table 4. |
| *UGT1A1* | *6 | rs4148323 | rs4148323 | G | A | 8.76 | 3.38–20.88 | 2 | 150 | 76.61 | 0.4171 | Random-effects logit (DL) | Pooled estimate across 2 independent study rows. |
| *UGT1A1* | c.189C>T | rs191471887 | rs191471887 | C | T | 0.00 | 0.00–3.70 | 1 | 50 | — | — | Single-study Wilson | Source-level single-study estimate. |
| *UGT1A1* | rs1042640C>G | rs1042640 | rs1042640 | C | G | 25.00 | 19.51–31.43 | 1 | 100 | — | — | Single-study Wilson | Source-level single-study estimate. |
| *UGT1A1* | rs10929303C>T | rs10929303 | rs10929303 | C | T | 25.00 | 19.51–31.43 | 1 | 100 | — | — | Single-study Wilson | Source-level single-study estimate. |
| *UGT1A1* | rs8330C>G | rs8330 | rs8330 | C | G | 25.00 | 19.51–31.43 | 1 | 100 | — | — | Single-study Wilson | Source-level single-study estimate. |
| *VKORC1* | -1173C>T | rs9934438 | rs9934438 | C | T | 85.33 | 80.88–88.89 | 1 | 150 | — | — | Single-study Wilson | Source label harmonized with the other rs9934438 C>T representation for the main-text estimate: 85.43% (95% CI 81.61–88.56), k=2, N=199, I²=0.00%. |
| *VKORC1* | -1639G>A | rs9923231 | rs9923231 | G | A | 89.91 | 81.13–94.86 | 2 | 199 | 76.14 | 0.2112 | Random-effects logit (DL) | Pooled estimate across 2 independent study rows. |
| *VKORC1* | 1173C>T | rs9934438 | rs9934438 | C | T | 85.71 | 77.44–91.30 | 1 | 49 | — | — | Single-study Wilson | Source label harmonized with the other rs9934438 C>T representation for the main-text estimate: 85.43% (95% CI 81.61–88.56), k=2, N=199, I²=0.00%. |
| *VKORC1* | 1542G>C | rs8050894 | rs8050894 | G | C | 85.71 | 77.44–91.30 | 1 | 49 | — | — | Single-study Wilson | Source-level single-study estimate. |
| *VKORC1* | 2255C>T | rs2359612 | rs2359612 | C | T | 85.71 | 77.44–91.30 | 1 | 49 | — | — | Single-study Wilson | Source-level single-study estimate. |
| *VKORC1* | 3730G>A | rs7294 | rs7294 | G | A | 14.29 | 8.70–22.56 | 1 | 49 | — | — | Single-study Wilson | Source-level single-study estimate. |
| *VKORC1* | 497T>G | rs2884737 | rs2884737 | T | G | 1.02 | 0.18–5.56 | 1 | 49 | — | — | Single-study Wilson | Source-level single-study estimate. |
