# Supplementary material for: Vietnamese pharmacogenomic variation in global context: a systematic review and meta-analysis for clinical implementation
Source: Front Pharmacol. 2026 Sep 3;17:1921144. doi: 10.3389/fphar.2026.1921144 (PMC13582518; doi:10.3389/fphar.2026.1921144)
Supplement: Supplementary file 4 [file Table4.docx]

**Supplementary Table S4. Sensitivity comparison of Vietnamese allele-frequency estimates across predefined analytic scenarios**

Sensitivity scenarios restrict the evidence by methodological quality, sample size, availability of a single-rsID representation, Hardy–Weinberg status, Kinh ethnicity, or healthy/control sampling. Scenario estimates are recomputed from the eligible row-level data and compared with the corrected source-level estimate in Supplementary Table S3.

Rows remain at the source-label representation level. Main-text alias consolidation is therefore not duplicated in this table; the corrected harmonized estimates for CYP2B6 rs3745274, SLCO1B1 rs2306283, and VKORC1 rs9934438 are stated in Supplementary Table S3 and the main manuscript. An absolute difference is descriptive and is not, by itself, a test of clinical importance.

| **Scenario** | **Gene** | **Allele/variant representation** | **rsID** | **Reference allele/state** | **Effect allele/state** | **Scenario estimate, %** | **95% CI** | **k** | **Total N** | **I², %** | **Primary source-level estimate, %** | **Primary source-level 95% CI** | **Absolute difference, pp** | **Analysis model** |
| --- | --- | --- | --- | --- | --- | --- | --- | --- | --- | --- | --- | --- | --- | --- |
| HWE passed or not informative | *CYP3A5* | *3 | rs776746 | A | G | 72.21 | 65.12–78.34 | 5 | 441 | 79.32 | 60.81 | 44.96–74.67 | 11.40 | Random-effects logit (DL) |
| Kinh-only samples | *NAT2* | *6 | rs1799930 | G | A | 36.00 | 29.67–42.86 | 1 | 100 | — | 27.00 | 18.69–37.31 | 9.00 | Single-study Wilson |
| Healthy/control samples only | *NAT2* | *6 | rs1799930 | G | A | 36.00 | 29.67–42.86 | 1 | 100 | — | 27.00 | 18.69–37.31 | 9.00 | Single-study Wilson |
| Kinh-only samples | *CYP3A5* | *3 | rs776746 | A | G | 66.67 | 58.62–73.84 | 1 | 72 | — | 60.81 | 44.96–74.67 | 5.86 | Single-study Wilson |
| HWE passed or not informative | *NAT2* | *6 | rs1799930 | G | A | 32.88 | 29.53–36.40 | 3 | 357 | 0.00 | 27.00 | 18.69–37.31 | 5.88 | Random-effects logit (DL) |
| Sample size ≥50 | *CYP3A5* | *3 | rs776746 | A | G | 65.89 | 51.22–78.04 | 6 | 505 | 94.77 | 60.81 | 44.96–74.67 | 5.08 | Random-effects logit (DL) |
| Healthy/control samples only | *CYP2C19* | *2 | rs4244285 | G | A | 24.66 | 21.85–27.70 | 4 | 417 | 0.00 | 27.90 | 26.57–29.27 | 3.24 | Random-effects logit (DL) |
| HWE passed or not informative | *VKORC1* | -1639G>A | rs9923231 | G | A | 85.71 | 77.44–91.30 | 1 | 49 | — | 89.91 | 81.13–94.86 | 4.20 | Single-study Wilson |
| Kinh-only samples | *UGT1A1* | *6 | rs4148323 | G | A | 13.00 | 9.03–18.37 | 1 | 100 | — | 8.76 | 3.38–20.88 | 4.24 | Single-study Wilson |
| Healthy/control samples only | *UGT1A1* | *6 | rs4148323 | G | A | 13.00 | 9.03–18.37 | 1 | 100 | — | 8.76 | 3.38–20.88 | 4.24 | Single-study Wilson |
| Kinh-only samples | *NAT2* | *7 | rs1799931 | G | A | 14.50 | 10.29–20.05 | 1 | 100 | — | 17.75 | 13.51–22.95 | 3.25 | Single-study Wilson |
| Healthy/control samples only | *NAT2* | *7 | rs1799931 | G | A | 14.50 | 10.29–20.05 | 1 | 100 | — | 17.75 | 13.51–22.95 | 3.25 | Single-study Wilson |
| Sample size ≥50 | *VKORC1* | -1639G>A | rs9923231 | G | A | 92.67 | 89.15–95.11 | 1 | 150 | — | 89.91 | 81.13–94.86 | 2.76 | Single-study Wilson |
| Kinh-only samples | *CYP2C19* | *2 | rs4244285 | G | A | 25.22 | 20.11–31.11 | 3 | 272 | 55.58 | 27.90 | 26.57–29.27 | 2.68 | Random-effects logit (DL) |
| Kinh-only samples | *NAT2* | *5 | rs1801280 | T | C | 8.00 | 4.98–12.60 | 1 | 100 | — | 5.97 | 3.96–8.89 | 2.03 | Single-study Wilson |
| Healthy/control samples only | *NAT2* | *5 | rs1801280 | T | C | 8.00 | 4.98–12.60 | 1 | 100 | — | 5.97 | 3.96–8.89 | 2.03 | Single-study Wilson |
| Healthy/control samples only | *CYP2C19* | *3 | rs4986893 | G | A | 5.68 | 3.23–9.78 | 5 | 582 | 81.35 | 5.74 | 4.82–6.82 | 0.06 | Random-effects logit (DL) |
| Kinh-only samples | *CYP2C19* | *3 | rs4986893 | G | A | 4.72 | 3.48–6.38 | 4 | 437 | 0.00 | 5.74 | 4.82–6.82 | 1.02 | Random-effects logit (DL) |
| Healthy/control samples only | *CYP2C9* | *3 | rs1057910 | A | C | 2.91 | 1.90–4.45 | 3 | 357 | 0.00 | 3.27 | 2.46–4.32 | 0.36 | Random-effects logit (DL) |
| Kinh-only samples | *CYP2C19* | *17 | rs12248560 | C | T | 1.00 | 0.28–3.57 | 1 | 100 | — | 1.37 | 0.76–2.46 | 0.37 | Single-study Wilson |
| Healthy/control samples only | *CYP2C19* | *17 | rs12248560 | C | T | 1.00 | 0.28–3.57 | 1 | 100 | — | 1.37 | 0.76–2.46 | 0.37 | Single-study Wilson |
| Kinh-only samples | *CYP2D6* | *5 | — | NON-*5 | *5 | 8.09 | 5.40–11.94 | 1 | 136 | — | 7.47 | 5.51–10.06 | 0.62 | Single-study Wilson |
| HWE passed or not informative | *CYP2C19* | *3 | rs4986893 | G | A | 5.35 | 4.64–6.15 | 18 | 2,039 | 9.80 | 5.74 | 4.82–6.82 | 0.39 | Random-effects logit (DL) |
| Kinh-only samples | *CYP2C9* | *3 | rs1057910 | A | C | 2.91 | 1.90–4.45 | 3 | 357 | 0.00 | 3.27 | 2.46–4.32 | 0.36 | Random-effects logit (DL) |
| HWE passed or not informative | *CYP2C19* | *2 | rs4244285 | G | A | 27.62 | 26.03–29.26 | 17 | 1,900 | 19.48 | 27.90 | 26.57–29.27 | 0.28 | Random-effects logit (DL) |
| Strict sensitivity set | *CYP2C19* | *2 | rs4244285 | G | A | 27.69 | 26.17–29.26 | 18 | 1,964 | 14.92 | 27.90 | 26.57–29.27 | 0.21 | Random-effects logit (DL) |
| Healthy/control samples only | *CYP2C9* | *2 | rs1799853 | C | T | 0.20 | 0.03–1.40 | 2 | 257 | 0.00 | 0.22 | 0.04–1.08 | 0.02 | Random-effects logit (DL) |
| Sample size ≥50 | *CYP2C19* | *3 | rs4986893 | G | A | 5.77 | 4.83–6.88 | 19 | 2,749 | 56.57 | 5.74 | 4.82–6.82 | 0.03 | Random-effects logit (DL) |
| Sample size ≥50 | *CYP2C19* | *2 | rs4244285 | G | A | 27.88 | 26.49–29.31 | 18 | 2,584 | 16.58 | 27.90 | 26.57–29.27 | 0.02 | Random-effects logit (DL) |
| Kinh-only samples | *CYP2C9* | *2 | rs1799853 | C | T | 0.20 | 0.03–1.40 | 2 | 257 | 0.00 | 0.22 | 0.04–1.08 | 0.02 | Random-effects logit (DL) |
| Strict sensitivity set | *ABCB1* | 3435C>T | rs1045642 | C | T | 40.28 | 32.62–48.44 | 1 | 72 | — | 40.28 | 32.62–48.44 | 0.00 | Single-study Wilson |
| Sample size ≥50 | *ABCB1* | 3435C>T | rs1045642 | C | T | 40.28 | 32.62–48.44 | 1 | 72 | — | 40.28 | 32.62–48.44 | 0.00 | Single-study Wilson |
| Single-rsID representations only | *ABCB1* | 3435C>T | rs1045642 | C | T | 40.28 | 32.62–48.44 | 1 | 72 | — | 40.28 | 32.62–48.44 | 0.00 | Single-study Wilson |
| HWE passed or not informative | *ABCB1* | 3435C>T | rs1045642 | C | T | 40.28 | 32.62–48.44 | 1 | 72 | — | 40.28 | 32.62–48.44 | 0.00 | Single-study Wilson |
| Kinh-only samples | *ABCB1* | 3435C>T | rs1045642 | C | T | 40.28 | 32.62–48.44 | 1 | 72 | — | 40.28 | 32.62–48.44 | 0.00 | Single-study Wilson |
| Strict sensitivity set | *ABCG2* | rs2231142 | rs2231142 | G | T | 36.00 | 29.67–42.86 | 1 | 100 | — | 36.00 | 29.67–42.86 | 0.00 | Single-study Wilson |
| Sample size ≥50 | *ABCG2* | rs2231142 | rs2231142 | G | T | 36.00 | 29.67–42.86 | 1 | 100 | — | 36.00 | 29.67–42.86 | 0.00 | Single-study Wilson |
| Single-rsID representations only | *ABCG2* | rs2231142 | rs2231142 | G | T | 36.00 | 29.67–42.86 | 1 | 100 | — | 36.00 | 29.67–42.86 | 0.00 | Single-study Wilson |
| HWE passed or not informative | *ABCG2* | rs2231142 | rs2231142 | G | T | 36.00 | 29.67–42.86 | 1 | 100 | — | 36.00 | 29.67–42.86 | 0.00 | Single-study Wilson |
| Kinh-only samples | *ABCG2* | rs2231142 | rs2231142 | G | T | 36.00 | 29.67–42.86 | 1 | 100 | — | 36.00 | 29.67–42.86 | 0.00 | Single-study Wilson |
| Healthy/control samples only | *ABCG2* | rs2231142 | rs2231142 | G | T | 36.00 | 29.67–42.86 | 1 | 100 | — | 36.00 | 29.67–42.86 | 0.00 | Single-study Wilson |
| Strict sensitivity set | *CYP1B1* | Dup | — | WT | DUP | 0.97 | 0.33–2.82 | 1 | 154 | — | 0.97 | 0.33–2.82 | 0.00 | Single-study Wilson |
| Sample size ≥50 | *CYP1B1* | Dup | — | WT | DUP | 0.97 | 0.33–2.82 | 1 | 154 | — | 0.97 | 0.33–2.82 | 0.00 | Single-study Wilson |
| HWE passed or not informative | *CYP1B1* | Dup | — | WT | DUP | 0.97 | 0.33–2.82 | 1 | 154 | — | 0.97 | 0.33–2.82 | 0.00 | Single-study Wilson |
| Kinh-only samples | *CYP1B1* | Dup | — | WT | DUP | 0.97 | 0.33–2.82 | 1 | 154 | — | 0.97 | 0.33–2.82 | 0.00 | Single-study Wilson |
| Strict sensitivity set | *CYP2A6* | *11 | rs28399447 | A | G | 1.50 | 0.51–4.32 | 1 | 100 | — | 1.50 | 0.51–4.32 | 0.00 | Single-study Wilson |
| Sample size ≥50 | *CYP2A6* | *11 | rs28399447 | A | G | 1.50 | 0.51–4.32 | 1 | 100 | — | 1.50 | 0.51–4.32 | 0.00 | Single-study Wilson |
| Single-rsID representations only | *CYP2A6* | *11 | rs28399447 | A | G | 1.50 | 0.51–4.32 | 1 | 100 | — | 1.50 | 0.51–4.32 | 0.00 | Single-study Wilson |
| HWE passed or not informative | *CYP2A6* | *11 | rs28399447 | A | G | 1.50 | 0.51–4.32 | 1 | 100 | — | 1.50 | 0.51–4.32 | 0.00 | Single-study Wilson |
| Kinh-only samples | *CYP2A6* | *11 | rs28399447 | A | G | 1.50 | 0.51–4.32 | 1 | 100 | — | 1.50 | 0.51–4.32 | 0.00 | Single-study Wilson |
| Healthy/control samples only | *CYP2A6* | *11 | rs28399447 | A | G | 1.50 | 0.51–4.32 | 1 | 100 | — | 1.50 | 0.51–4.32 | 0.00 | Single-study Wilson |
| Strict sensitivity set | *CYP2A6* | *15 | rs199916117 | T | C | 0.50 | 0.09–2.78 | 1 | 100 | — | 0.50 | 0.09–2.78 | 0.00 | Single-study Wilson |
| Sample size ≥50 | *CYP2A6* | *15 | rs199916117 | T | C | 0.50 | 0.09–2.78 | 1 | 100 | — | 0.50 | 0.09–2.78 | 0.00 | Single-study Wilson |
| Single-rsID representations only | *CYP2A6* | *15 | rs199916117 | T | C | 0.50 | 0.09–2.78 | 1 | 100 | — | 0.50 | 0.09–2.78 | 0.00 | Single-study Wilson |
| HWE passed or not informative | *CYP2A6* | *15 | rs199916117 | T | C | 0.50 | 0.09–2.78 | 1 | 100 | — | 0.50 | 0.09–2.78 | 0.00 | Single-study Wilson |
| Kinh-only samples | *CYP2A6* | *15 | rs199916117 | T | C | 0.50 | 0.09–2.78 | 1 | 100 | — | 0.50 | 0.09–2.78 | 0.00 | Single-study Wilson |
| Healthy/control samples only | *CYP2A6* | *15 | rs199916117 | T | C | 0.50 | 0.09–2.78 | 1 | 100 | — | 0.50 | 0.09–2.78 | 0.00 | Single-study Wilson |
| Strict sensitivity set | *CYP2A6* | *4 | — | WT | DEL | 11.81 | 7.50–18.09 | 1 | 72 | — | 11.81 | 7.50–18.09 | 0.00 | Single-study Wilson |
| Sample size ≥50 | *CYP2A6* | *4 | — | WT | DEL | 11.81 | 7.50–18.09 | 1 | 72 | — | 11.81 | 7.50–18.09 | 0.00 | Single-study Wilson |
| HWE passed or not informative | *CYP2A6* | *4 | — | WT | DEL | 11.81 | 7.50–18.09 | 1 | 72 | — | 11.81 | 7.50–18.09 | 0.00 | Single-study Wilson |
| Kinh-only samples | *CYP2A6* | *4 | — | WT | DEL | 11.81 | 7.50–18.09 | 1 | 72 | — | 11.81 | 7.50–18.09 | 0.00 | Single-study Wilson |
| Strict sensitivity set | *CYP2A6* | *5 | rs5031017 | G | T | 14.58 | 9.74–21.27 | 1 | 72 | — | 14.58 | 9.74–21.27 | 0.00 | Single-study Wilson |
| Sample size ≥50 | *CYP2A6* | *5 | rs5031017 | G | T | 14.58 | 9.74–21.27 | 1 | 72 | — | 14.58 | 9.74–21.27 | 0.00 | Single-study Wilson |
| Single-rsID representations only | *CYP2A6* | *5 | rs5031017 | G | T | 14.58 | 9.74–21.27 | 1 | 72 | — | 14.58 | 9.74–21.27 | 0.00 | Single-study Wilson |
| HWE passed or not informative | *CYP2A6* | *5 | rs5031017 | G | T | 14.58 | 9.74–21.27 | 1 | 72 | — | 14.58 | 9.74–21.27 | 0.00 | Single-study Wilson |
| Kinh-only samples | *CYP2A6* | *5 | rs5031017 | G | T | 14.58 | 9.74–21.27 | 1 | 72 | — | 14.58 | 9.74–21.27 | 0.00 | Single-study Wilson |
| Strict sensitivity set | *CYP2A6* | Del | — | WT | DEL | 11.04 | 8.01–15.03 | 1 | 154 | — | 11.04 | 8.01–15.03 | 0.00 | Single-study Wilson |
| Sample size ≥50 | *CYP2A6* | Del | — | WT | DEL | 11.04 | 8.01–15.03 | 1 | 154 | — | 11.04 | 8.01–15.03 | 0.00 | Single-study Wilson |
| HWE passed or not informative | *CYP2A6* | Del | — | WT | DEL | 11.04 | 8.01–15.03 | 1 | 154 | — | 11.04 | 8.01–15.03 | 0.00 | Single-study Wilson |
| Kinh-only samples | *CYP2A6* | Del | — | WT | DEL | 11.04 | 8.01–15.03 | 1 | 154 | — | 11.04 | 8.01–15.03 | 0.00 | Single-study Wilson |
| Strict sensitivity set | *CYP2A6* | rs1809810 | rs1809810 | T | A | 2.00 | 0.78–5.03 | 1 | 100 | — | 2.00 | 0.78–5.03 | 0.00 | Single-study Wilson |
| Sample size ≥50 | *CYP2A6* | rs1809810 | rs1809810 | T | A | 2.00 | 0.78–5.03 | 1 | 100 | — | 2.00 | 0.78–5.03 | 0.00 | Single-study Wilson |
| Single-rsID representations only | *CYP2A6* | rs1809810 | rs1809810 | T | A | 2.00 | 0.78–5.03 | 1 | 100 | — | 2.00 | 0.78–5.03 | 0.00 | Single-study Wilson |
| HWE passed or not informative | *CYP2A6* | rs1809810 | rs1809810 | T | A | 2.00 | 0.78–5.03 | 1 | 100 | — | 2.00 | 0.78–5.03 | 0.00 | Single-study Wilson |
| Kinh-only samples | *CYP2A6* | rs1809810 | rs1809810 | T | A | 2.00 | 0.78–5.03 | 1 | 100 | — | 2.00 | 0.78–5.03 | 0.00 | Single-study Wilson |
| Healthy/control samples only | *CYP2A6* | rs1809810 | rs1809810 | T | A | 2.00 | 0.78–5.03 | 1 | 100 | — | 2.00 | 0.78–5.03 | 0.00 | Single-study Wilson |
| Strict sensitivity set | *CYP2B6* | *2 | rs8192709 | C | T | 5.50 | 3.10–9.58 | 1 | 100 | — | 5.50 | 3.10–9.58 | 0.00 | Single-study Wilson |
| Sample size ≥50 | *CYP2B6* | *2 | rs8192709 | C | T | 5.50 | 3.10–9.58 | 1 | 100 | — | 5.50 | 3.10–9.58 | 0.00 | Single-study Wilson |
| Single-rsID representations only | *CYP2B6* | *2 | rs8192709 | C | T | 5.50 | 3.10–9.58 | 1 | 100 | — | 5.50 | 3.10–9.58 | 0.00 | Single-study Wilson |
| HWE passed or not informative | *CYP2B6* | *2 | rs8192709 | C | T | 5.50 | 3.10–9.58 | 1 | 100 | — | 5.50 | 3.10–9.58 | 0.00 | Single-study Wilson |
| Kinh-only samples | *CYP2B6* | *2 | rs8192709 | C | T | 5.50 | 3.10–9.58 | 1 | 100 | — | 5.50 | 3.10–9.58 | 0.00 | Single-study Wilson |
| Healthy/control samples only | *CYP2B6* | *2 | rs8192709 | C | T | 5.50 | 3.10–9.58 | 1 | 100 | — | 5.50 | 3.10–9.58 | 0.00 | Single-study Wilson |
| Strict sensitivity set | *CYP2B6* | *4 | rs2279343 | A | G | 35.42 | 28.08–43.51 | 1 | 72 | — | 35.42 | 28.08–43.51 | 0.00 | Single-study Wilson |
| Sample size ≥50 | *CYP2B6* | *4 | rs2279343 | A | G | 35.42 | 28.08–43.51 | 1 | 72 | — | 35.42 | 28.08–43.51 | 0.00 | Single-study Wilson |
| Single-rsID representations only | *CYP2B6* | *4 | rs2279343 | A | G | 35.42 | 28.08–43.51 | 1 | 72 | — | 35.42 | 28.08–43.51 | 0.00 | Single-study Wilson |
| HWE passed or not informative | *CYP2B6* | *4 | rs2279343 | A | G | 35.42 | 28.08–43.51 | 1 | 72 | — | 35.42 | 28.08–43.51 | 0.00 | Single-study Wilson |
| Kinh-only samples | *CYP2B6* | *4 | rs2279343 | A | G | 35.42 | 28.08–43.51 | 1 | 72 | — | 35.42 | 28.08–43.51 | 0.00 | Single-study Wilson |
| Strict sensitivity set | *CYP2B6* | *5 | rs3211371 | C | T | 0.00 | 0.00–2.60 | 1 | 72 | — | 0.00 | 0.00–2.60 | 0.00 | Single-study Wilson |
| Sample size ≥50 | *CYP2B6* | *5 | rs3211371 | C | T | 0.00 | 0.00–2.60 | 1 | 72 | — | 0.00 | 0.00–2.60 | 0.00 | Single-study Wilson |
| Single-rsID representations only | *CYP2B6* | *5 | rs3211371 | C | T | 0.00 | 0.00–2.60 | 1 | 72 | — | 0.00 | 0.00–2.60 | 0.00 | Single-study Wilson |
| HWE passed or not informative | *CYP2B6* | *5 | rs3211371 | C | T | 0.00 | 0.00–2.60 | 1 | 72 | — | 0.00 | 0.00–2.60 | 0.00 | Single-study Wilson |
| Kinh-only samples | *CYP2B6* | *5 | rs3211371 | C | T | 0.00 | 0.00–2.60 | 1 | 72 | — | 0.00 | 0.00–2.60 | 0.00 | Single-study Wilson |
| Strict sensitivity set | *CYP2B6* | 516G>T | rs3745274 | G | T | 27.08 | 20.49–34.87 | 1 | 72 | — | 27.08 | 20.49–34.87 | 0.00 | Single-study Wilson |
| Sample size ≥50 | *CYP2B6* | 516G>T | rs3745274 | G | T | 27.08 | 20.49–34.87 | 1 | 72 | — | 27.08 | 20.49–34.87 | 0.00 | Single-study Wilson |
| Single-rsID representations only | *CYP2B6* | 516G>T | rs3745274 | G | T | 27.08 | 20.49–34.87 | 1 | 72 | — | 27.08 | 20.49–34.87 | 0.00 | Single-study Wilson |
| HWE passed or not informative | *CYP2B6* | 516G>T | rs3745274 | G | T | 27.08 | 20.49–34.87 | 1 | 72 | — | 27.08 | 20.49–34.87 | 0.00 | Single-study Wilson |
| Kinh-only samples | *CYP2B6* | 516G>T | rs3745274 | G | T | 27.08 | 20.49–34.87 | 1 | 72 | — | 27.08 | 20.49–34.87 | 0.00 | Single-study Wilson |
| Strict sensitivity set | *CYP2B6* | rs3745274 | rs3745274 | G | T | 23.00 | 17.71–29.31 | 1 | 100 | — | 23.00 | 17.71–29.31 | 0.00 | Single-study Wilson |
| Sample size ≥50 | *CYP2B6* | rs3745274 | rs3745274 | G | T | 23.00 | 17.71–29.31 | 1 | 100 | — | 23.00 | 17.71–29.31 | 0.00 | Single-study Wilson |
| Single-rsID representations only | *CYP2B6* | rs3745274 | rs3745274 | G | T | 23.00 | 17.71–29.31 | 1 | 100 | — | 23.00 | 17.71–29.31 | 0.00 | Single-study Wilson |
| HWE passed or not informative | *CYP2B6* | rs3745274 | rs3745274 | G | T | 23.00 | 17.71–29.31 | 1 | 100 | — | 23.00 | 17.71–29.31 | 0.00 | Single-study Wilson |
| Kinh-only samples | *CYP2B6* | rs3745274 | rs3745274 | G | T | 23.00 | 17.71–29.31 | 1 | 100 | — | 23.00 | 17.71–29.31 | 0.00 | Single-study Wilson |
| Healthy/control samples only | *CYP2B6* | rs3745274 | rs3745274 | G | T | 23.00 | 17.71–29.31 | 1 | 100 | — | 23.00 | 17.71–29.31 | 0.00 | Single-study Wilson |
| Strict sensitivity set | *CYP2C19* | *17 | rs11188072 | C | T | 1.04 | 0.29–3.72 | 1 | 96 | — | 1.04 | 0.29–3.72 | 0.00 | Single-study Wilson |
| Strict sensitivity set | *CYP2C19* | *17 | rs12248560 | C | T | 1.37 | 0.76–2.46 | 3 | 422 | 0.00 | 1.37 | 0.76–2.46 | 0.00 | Random-effects logit (DL) |
| Sample size ≥50 | *CYP2C19* | *17 | rs11188072 | C | T | 1.04 | 0.29–3.72 | 1 | 96 | — | 1.04 | 0.29–3.72 | 0.00 | Single-study Wilson |
| Sample size ≥50 | *CYP2C19* | *17 | rs12248560 | C | T | 1.37 | 0.76–2.46 | 3 | 422 | 0.00 | 1.37 | 0.76–2.46 | 0.00 | Random-effects logit (DL) |
| Single-rsID representations only | *CYP2C19* | *17 | rs11188072 | C | T | 1.04 | 0.29–3.72 | 1 | 96 | — | 1.04 | 0.29–3.72 | 0.00 | Single-study Wilson |
| Single-rsID representations only | *CYP2C19* | *17 | rs12248560 | C | T | 1.37 | 0.76–2.46 | 3 | 422 | 0.00 | 1.37 | 0.76–2.46 | 0.00 | Random-effects logit (DL) |
| HWE passed or not informative | *CYP2C19* | *17 | rs11188072 | C | T | 1.04 | 0.29–3.72 | 1 | 96 | — | 1.04 | 0.29–3.72 | 0.00 | Single-study Wilson |
| HWE passed or not informative | *CYP2C19* | *17 | rs12248560 | C | T | 1.37 | 0.76–2.46 | 3 | 422 | 0.00 | 1.37 | 0.76–2.46 | 0.00 | Random-effects logit (DL) |
| Single-rsID representations only | *CYP2C19* | *2 | rs4244285 | G | A | 27.90 | 26.57–29.27 | 19 | 2,614 | 11.80 | 27.90 | 26.57–29.27 | 0.00 | Random-effects logit (DL) |
| Strict sensitivity set | *CYP2C19* | *26 | rs375781227 | G | A | 0.50 | 0.09–2.78 | 1 | 100 | — | 0.50 | 0.09–2.78 | 0.00 | Single-study Wilson |
| Sample size ≥50 | *CYP2C19* | *26 | rs375781227 | G | A | 0.50 | 0.09–2.78 | 1 | 100 | — | 0.50 | 0.09–2.78 | 0.00 | Single-study Wilson |
| Single-rsID representations only | *CYP2C19* | *26 | rs375781227 | G | A | 0.50 | 0.09–2.78 | 1 | 100 | — | 0.50 | 0.09–2.78 | 0.00 | Single-study Wilson |
| HWE passed or not informative | *CYP2C19* | *26 | rs375781227 | G | A | 0.50 | 0.09–2.78 | 1 | 100 | — | 0.50 | 0.09–2.78 | 0.00 | Single-study Wilson |
| Kinh-only samples | *CYP2C19* | *26 | rs375781227 | G | A | 0.50 | 0.09–2.78 | 1 | 100 | — | 0.50 | 0.09–2.78 | 0.00 | Single-study Wilson |
| Healthy/control samples only | *CYP2C19* | *26 | rs375781227 | G | A | 0.50 | 0.09–2.78 | 1 | 100 | — | 0.50 | 0.09–2.78 | 0.00 | Single-study Wilson |
| Strict sensitivity set | *CYP2C19* | *3 | rs4986893 | G | A | 5.74 | 4.82–6.82 | 20 | 2,779 | 54.69 | 5.74 | 4.82–6.82 | 0.00 | Random-effects logit (DL) |
| Single-rsID representations only | *CYP2C19* | *3 | rs4986893 | G | A | 5.74 | 4.82–6.82 | 20 | 2,779 | 54.69 | 5.74 | 4.82–6.82 | 0.00 | Random-effects logit (DL) |
| Strict sensitivity set | *CYP2C19* | *5 | rs56337013 | C | T | 0.50 | 0.09–2.78 | 1 | 100 | — | 0.50 | 0.09–2.78 | 0.00 | Single-study Wilson |
| Sample size ≥50 | *CYP2C19* | *5 | rs56337013 | C | T | 0.50 | 0.09–2.78 | 1 | 100 | — | 0.50 | 0.09–2.78 | 0.00 | Single-study Wilson |
| Single-rsID representations only | *CYP2C19* | *5 | rs56337013 | C | T | 0.50 | 0.09–2.78 | 1 | 100 | — | 0.50 | 0.09–2.78 | 0.00 | Single-study Wilson |
| HWE passed or not informative | *CYP2C19* | *5 | rs56337013 | C | T | 0.50 | 0.09–2.78 | 1 | 100 | — | 0.50 | 0.09–2.78 | 0.00 | Single-study Wilson |
| Kinh-only samples | *CYP2C19* | *5 | rs56337013 | C | T | 0.50 | 0.09–2.78 | 1 | 100 | — | 0.50 | 0.09–2.78 | 0.00 | Single-study Wilson |
| Healthy/control samples only | *CYP2C19* | *5 | rs56337013 | C | T | 0.50 | 0.09–2.78 | 1 | 100 | — | 0.50 | 0.09–2.78 | 0.00 | Single-study Wilson |
| Strict sensitivity set | *CYP2C19* | rs3758581 | rs3758581 | A | G | 97.50 | 94.28–98.93 | 1 | 100 | — | 97.50 | 94.28–98.93 | 0.00 | Single-study Wilson |
| Sample size ≥50 | *CYP2C19* | rs3758581 | rs3758581 | A | G | 97.50 | 94.28–98.93 | 1 | 100 | — | 97.50 | 94.28–98.93 | 0.00 | Single-study Wilson |
| Single-rsID representations only | *CYP2C19* | rs3758581 | rs3758581 | A | G | 97.50 | 94.28–98.93 | 1 | 100 | — | 97.50 | 94.28–98.93 | 0.00 | Single-study Wilson |
| HWE passed or not informative | *CYP2C19* | rs3758581 | rs3758581 | A | G | 97.50 | 94.28–98.93 | 1 | 100 | — | 97.50 | 94.28–98.93 | 0.00 | Single-study Wilson |
| Kinh-only samples | *CYP2C19* | rs3758581 | rs3758581 | A | G | 97.50 | 94.28–98.93 | 1 | 100 | — | 97.50 | 94.28–98.93 | 0.00 | Single-study Wilson |
| Healthy/control samples only | *CYP2C19* | rs3758581 | rs3758581 | A | G | 97.50 | 94.28–98.93 | 1 | 100 | — | 97.50 | 94.28–98.93 | 0.00 | Single-study Wilson |
| Strict sensitivity set | *CYP2C8* | *11 | rs78637571 | G | T | 1.00 | 0.27–3.57 | 1 | 100 | — | 1.00 | 0.27–3.57 | 0.00 | Single-study Wilson |
| Sample size ≥50 | *CYP2C8* | *11 | rs78637571 | G | T | 1.00 | 0.27–3.57 | 1 | 100 | — | 1.00 | 0.27–3.57 | 0.00 | Single-study Wilson |
| Single-rsID representations only | *CYP2C8* | *11 | rs78637571 | G | T | 1.00 | 0.27–3.57 | 1 | 100 | — | 1.00 | 0.27–3.57 | 0.00 | Single-study Wilson |
| HWE passed or not informative | *CYP2C8* | *11 | rs78637571 | G | T | 1.00 | 0.27–3.57 | 1 | 100 | — | 1.00 | 0.27–3.57 | 0.00 | Single-study Wilson |
| Kinh-only samples | *CYP2C8* | *11 | rs78637571 | G | T | 1.00 | 0.27–3.57 | 1 | 100 | — | 1.00 | 0.27–3.57 | 0.00 | Single-study Wilson |
| Strict sensitivity set | *CYP2C9* | *13 | rs72558187 | NON-*13 | *13 | 0.79 | 0.22–2.83 | 1 | 127 | — | 0.79 | 0.22–2.83 | 0.00 | Single-study Wilson |
| Strict sensitivity set | *CYP2C9* | *13 | rs72558187 | T | C | 0.50 | 0.09–2.78 | 1 | 100 | — | 0.50 | 0.09–2.78 | 0.00 | Single-study Wilson |
| Sample size ≥50 | *CYP2C9* | *13 | rs72558187 | NON-*13 | *13 | 0.79 | 0.22–2.83 | 1 | 127 | — | 0.79 | 0.22–2.83 | 0.00 | Single-study Wilson |
| Sample size ≥50 | *CYP2C9* | *13 | rs72558187 | T | C | 0.50 | 0.09–2.78 | 1 | 100 | — | 0.50 | 0.09–2.78 | 0.00 | Single-study Wilson |
| Single-rsID representations only | *CYP2C9* | *13 | rs72558187 | NON-*13 | *13 | 0.79 | 0.22–2.83 | 1 | 127 | — | 0.79 | 0.22–2.83 | 0.00 | Single-study Wilson |
| Single-rsID representations only | *CYP2C9* | *13 | rs72558187 | T | C | 0.50 | 0.09–2.78 | 1 | 100 | — | 0.50 | 0.09–2.78 | 0.00 | Single-study Wilson |
| HWE passed or not informative | *CYP2C9* | *13 | rs72558187 | NON-*13 | *13 | 0.79 | 0.22–2.83 | 1 | 127 | — | 0.79 | 0.22–2.83 | 0.00 | Single-study Wilson |
| HWE passed or not informative | *CYP2C9* | *13 | rs72558187 | T | C | 0.50 | 0.09–2.78 | 1 | 100 | — | 0.50 | 0.09–2.78 | 0.00 | Single-study Wilson |
| Kinh-only samples | *CYP2C9* | *13 | rs72558187 | T | C | 0.50 | 0.09–2.78 | 1 | 100 | — | 0.50 | 0.09–2.78 | 0.00 | Single-study Wilson |
| Healthy/control samples only | *CYP2C9* | *13 | rs72558187 | T | C | 0.50 | 0.09–2.78 | 1 | 100 | — | 0.50 | 0.09–2.78 | 0.00 | Single-study Wilson |
| Strict sensitivity set | *CYP2C9* | *2 | rs1799853 | C | T | 0.22 | 0.04–1.08 | 3 | 349 | 0.00 | 0.22 | 0.04–1.08 | 0.00 | Random-effects logit (DL) |
| Sample size ≥50 | *CYP2C9* | *2 | rs1799853 | C | T | 0.22 | 0.04–1.08 | 3 | 349 | 0.00 | 0.22 | 0.04–1.08 | 0.00 | Random-effects logit (DL) |
| Single-rsID representations only | *CYP2C9* | *2 | rs1799853 | C | T | 0.22 | 0.04–1.08 | 3 | 349 | 0.00 | 0.22 | 0.04–1.08 | 0.00 | Random-effects logit (DL) |
| HWE passed or not informative | *CYP2C9* | *2 | rs1799853 | C | T | 0.22 | 0.04–1.08 | 3 | 349 | 0.00 | 0.22 | 0.04–1.08 | 0.00 | Random-effects logit (DL) |
| Strict sensitivity set | *CYP2C9* | *3 | rs1057910 | A | C | 3.27 | 2.46–4.32 | 6 | 738 | 0.00 | 3.27 | 2.46–4.32 | 0.00 | Random-effects logit (DL) |
| Sample size ≥50 | *CYP2C9* | *3 | rs1057910 | A | C | 3.27 | 2.46–4.32 | 6 | 738 | 0.00 | 3.27 | 2.46–4.32 | 0.00 | Random-effects logit (DL) |
| Single-rsID representations only | *CYP2C9* | *3 | rs1057910 | A | C | 3.27 | 2.46–4.32 | 6 | 738 | 0.00 | 3.27 | 2.46–4.32 | 0.00 | Random-effects logit (DL) |
| HWE passed or not informative | *CYP2C9* | *3 | rs1057910 | A | C | 3.27 | 2.46–4.32 | 6 | 738 | 0.00 | 3.27 | 2.46–4.32 | 0.00 | Random-effects logit (DL) |
| Strict sensitivity set | *CYP2C9* | Del | — | WT | DEL | 0.97 | 0.33–2.82 | 1 | 154 | — | 0.97 | 0.33–2.82 | 0.00 | Single-study Wilson |
| Sample size ≥50 | *CYP2C9* | Del | — | WT | DEL | 0.97 | 0.33–2.82 | 1 | 154 | — | 0.97 | 0.33–2.82 | 0.00 | Single-study Wilson |
| HWE passed or not informative | *CYP2C9* | Del | — | WT | DEL | 0.97 | 0.33–2.82 | 1 | 154 | — | 0.97 | 0.33–2.82 | 0.00 | Single-study Wilson |
| Kinh-only samples | *CYP2C9* | Del | — | WT | DEL | 0.97 | 0.33–2.82 | 1 | 154 | — | 0.97 | 0.33–2.82 | 0.00 | Single-study Wilson |
| Strict sensitivity set | *CYP2D6* | *10 | — | NON-*10 | *10 | 43.75 | 37.98–49.69 | 1 | 136 | — | 43.75 | 37.98–49.69 | 0.00 | Single-study Wilson |
| Strict sensitivity set | *CYP2D6* | rs1065852 C>T marker | rs1065852 | C | T | 45.07 | 37.12–53.28 | 1 | 71 | — | 45.07 | 37.12–53.28 | 0.00 | Single-study Wilson |
| Sample size ≥50 | *CYP2D6* | *10 | — | NON-*10 | *10 | 43.75 | 37.98–49.69 | 1 | 136 | — | 43.75 | 37.98–49.69 | 0.00 | Single-study Wilson |
| Sample size ≥50 | *CYP2D6* | rs1065852 C>T marker | rs1065852 | C | T | 45.07 | 37.12–53.28 | 1 | 71 | — | 45.07 | 37.12–53.28 | 0.00 | Single-study Wilson |
| Single-rsID representations only | *CYP2D6* | rs1065852 C>T marker | rs1065852 | C | T | 45.07 | 37.12–53.28 | 1 | 71 | — | 45.07 | 37.12–53.28 | 0.00 | Single-study Wilson |
| HWE passed or not informative | *CYP2D6* | *10 | — | NON-*10 | *10 | 43.75 | 37.98–49.69 | 1 | 136 | — | 43.75 | 37.98–49.69 | 0.00 | Single-study Wilson |
| Kinh-only samples | *CYP2D6* | *10 | — | NON-*10 | *10 | 43.75 | 37.98–49.69 | 1 | 136 | — | 43.75 | 37.98–49.69 | 0.00 | Single-study Wilson |
| Kinh-only samples | *CYP2D6* | rs1065852 C>T marker | rs1065852 | C | T | 45.07 | 37.12–53.28 | 1 | 71 | — | 45.07 | 37.12–53.28 | 0.00 | Single-study Wilson |
| Strict sensitivity set | *CYP2D6* | *10B | — | NON-*10B | *10B | 55.95 | 49.78–61.95 | 1 | 126 | — | 55.95 | 49.78–61.95 | 0.00 | Single-study Wilson |
| Sample size ≥50 | *CYP2D6* | *10B | — | NON-*10B | *10B | 55.95 | 49.78–61.95 | 1 | 126 | — | 55.95 | 49.78–61.95 | 0.00 | Single-study Wilson |
| HWE passed or not informative | *CYP2D6* | *10B | — | NON-*10B | *10B | 55.95 | 49.78–61.95 | 1 | 126 | — | 55.95 | 49.78–61.95 | 0.00 | Single-study Wilson |
| Strict sensitivity set | *CYP2D6* | *10BX2 | — | NON-*10BX2 | *10BX2 | 0.40 | 0.07–2.21 | 1 | 126 | — | 0.40 | 0.07–2.21 | 0.00 | Single-study Wilson |
| Sample size ≥50 | *CYP2D6* | *10BX2 | — | NON-*10BX2 | *10BX2 | 0.40 | 0.07–2.21 | 1 | 126 | — | 0.40 | 0.07–2.21 | 0.00 | Single-study Wilson |
| HWE passed or not informative | *CYP2D6* | *10BX2 | — | NON-*10BX2 | *10BX2 | 0.40 | 0.07–2.21 | 1 | 126 | — | 0.40 | 0.07–2.21 | 0.00 | Single-study Wilson |
| Strict sensitivity set | *CYP2D6* | *13 | — | NON-*13 | *13 | 0.73 | 0.20–2.64 | 1 | 136 | — | 0.74 | 0.20–2.64 | 0.01 | Single-study Wilson |
| Sample size ≥50 | *CYP2D6* | *13 | — | NON-*13 | *13 | 0.73 | 0.20–2.64 | 1 | 136 | — | 0.74 | 0.20–2.64 | 0.01 | Single-study Wilson |
| HWE passed or not informative | *CYP2D6* | *13 | — | NON-*13 | *13 | 0.73 | 0.20–2.64 | 1 | 136 | — | 0.74 | 0.20–2.64 | 0.01 | Single-study Wilson |
| Kinh-only samples | *CYP2D6* | *13 | — | NON-*13 | *13 | 0.73 | 0.20–2.64 | 1 | 136 | — | 0.74 | 0.20–2.64 | 0.01 | Single-study Wilson |
| Strict sensitivity set | *CYP2D6* | *14 | — | NON-*14 | *14 | 0.37 | 0.07–2.05 | 1 | 136 | — | 0.37 | 0.06–2.05 | 0.00 | Single-study Wilson |
| Sample size ≥50 | *CYP2D6* | *14 | — | NON-*14 | *14 | 0.37 | 0.07–2.05 | 1 | 136 | — | 0.37 | 0.06–2.05 | 0.00 | Single-study Wilson |
| HWE passed or not informative | *CYP2D6* | *14 | — | NON-*14 | *14 | 0.37 | 0.07–2.05 | 1 | 136 | — | 0.37 | 0.06–2.05 | 0.00 | Single-study Wilson |
| Kinh-only samples | *CYP2D6* | *14 | — | NON-*14 | *14 | 0.37 | 0.07–2.05 | 1 | 136 | — | 0.37 | 0.06–2.05 | 0.00 | Single-study Wilson |
| Strict sensitivity set | *CYP2D6* | *14B | — | NON-*14B | *14B | 0.79 | 0.22–2.85 | 1 | 126 | — | 0.79 | 0.22–2.85 | 0.00 | Single-study Wilson |
| Sample size ≥50 | *CYP2D6* | *14B | — | NON-*14B | *14B | 0.79 | 0.22–2.85 | 1 | 126 | — | 0.79 | 0.22–2.85 | 0.00 | Single-study Wilson |
| HWE passed or not informative | *CYP2D6* | *14B | — | NON-*14B | *14B | 0.79 | 0.22–2.85 | 1 | 126 | — | 0.79 | 0.22–2.85 | 0.00 | Single-study Wilson |
| Strict sensitivity set | *CYP2D6* | *15 | — | NON-*15 | *15 | 0.37 | 0.07–2.05 | 1 | 136 | — | 0.37 | 0.06–2.05 | 0.00 | Single-study Wilson |
| Sample size ≥50 | *CYP2D6* | *15 | — | NON-*15 | *15 | 0.37 | 0.07–2.05 | 1 | 136 | — | 0.37 | 0.06–2.05 | 0.00 | Single-study Wilson |
| HWE passed or not informative | *CYP2D6* | *15 | — | NON-*15 | *15 | 0.37 | 0.07–2.05 | 1 | 136 | — | 0.37 | 0.06–2.05 | 0.00 | Single-study Wilson |
| Kinh-only samples | *CYP2D6* | *15 | — | NON-*15 | *15 | 0.37 | 0.07–2.05 | 1 | 136 | — | 0.37 | 0.06–2.05 | 0.00 | Single-study Wilson |
| Strict sensitivity set | *CYP2D6* | *1XN | — | NON-*1XN | *1XN | 0.40 | 0.07–2.21 | 1 | 126 | — | 0.40 | 0.07–2.21 | 0.00 | Single-study Wilson |
| Sample size ≥50 | *CYP2D6* | *1XN | — | NON-*1XN | *1XN | 0.40 | 0.07–2.21 | 1 | 126 | — | 0.40 | 0.07–2.21 | 0.00 | Single-study Wilson |
| HWE passed or not informative | *CYP2D6* | *1XN | — | NON-*1XN | *1XN | 0.40 | 0.07–2.21 | 1 | 126 | — | 0.40 | 0.07–2.21 | 0.00 | Single-study Wilson |
| Strict sensitivity set | *CYP2D6* | *2 | — | NON-*2 | *2 | 7.94 | 5.20–11.94 | 1 | 126 | — | 7.94 | 5.20–11.94 | 0.00 | Single-study Wilson |
| Strict sensitivity set | *CYP2D6* | *2 | — | NON-*2 | *2 | 7.72 | 5.11–11.51 | 1 | 136 | — | 7.72 | 5.10–11.51 | 0.00 | Single-study Wilson |
| Sample size ≥50 | *CYP2D6* | *2 | — | NON-*2 | *2 | 7.94 | 5.20–11.94 | 1 | 126 | — | 7.94 | 5.20–11.94 | 0.00 | Single-study Wilson |
| Sample size ≥50 | *CYP2D6* | *2 | — | NON-*2 | *2 | 7.72 | 5.11–11.51 | 1 | 136 | — | 7.72 | 5.10–11.51 | 0.00 | Single-study Wilson |
| HWE passed or not informative | *CYP2D6* | *2 | — | NON-*2 | *2 | 7.94 | 5.20–11.94 | 1 | 126 | — | 7.94 | 5.20–11.94 | 0.00 | Single-study Wilson |
| HWE passed or not informative | *CYP2D6* | *2 | — | NON-*2 | *2 | 7.72 | 5.11–11.51 | 1 | 136 | — | 7.72 | 5.10–11.51 | 0.00 | Single-study Wilson |
| Kinh-only samples | *CYP2D6* | *2 | — | NON-*2 | *2 | 7.72 | 5.11–11.51 | 1 | 136 | — | 7.72 | 5.10–11.51 | 0.00 | Single-study Wilson |
| Strict sensitivity set | *CYP2D6* | *21B | — | NON-*21B | *21B | 0.40 | 0.07–2.21 | 1 | 126 | — | 0.40 | 0.07–2.21 | 0.00 | Single-study Wilson |
| Sample size ≥50 | *CYP2D6* | *21B | — | NON-*21B | *21B | 0.40 | 0.07–2.21 | 1 | 126 | — | 0.40 | 0.07–2.21 | 0.00 | Single-study Wilson |
| HWE passed or not informative | *CYP2D6* | *21B | — | NON-*21B | *21B | 0.40 | 0.07–2.21 | 1 | 126 | — | 0.40 | 0.07–2.21 | 0.00 | Single-study Wilson |
| Strict sensitivity set | *CYP2D6* | *36 | — | NON-*36 | *36 | 0.37 | 0.07–2.05 | 1 | 136 | — | 0.37 | 0.06–2.05 | 0.00 | Single-study Wilson |
| Sample size ≥50 | *CYP2D6* | *36 | — | NON-*36 | *36 | 0.37 | 0.07–2.05 | 1 | 136 | — | 0.37 | 0.06–2.05 | 0.00 | Single-study Wilson |
| HWE passed or not informative | *CYP2D6* | *36 | — | NON-*36 | *36 | 0.37 | 0.07–2.05 | 1 | 136 | — | 0.37 | 0.06–2.05 | 0.00 | Single-study Wilson |
| Kinh-only samples | *CYP2D6* | *36 | — | NON-*36 | *36 | 0.37 | 0.07–2.05 | 1 | 136 | — | 0.37 | 0.06–2.05 | 0.00 | Single-study Wilson |
| Strict sensitivity set | *CYP2D6* | *36-*10 | — | NON-*36-*10 | *36-*10 | 11.40 | 8.15–15.72 | 1 | 136 | — | 11.40 | 8.15–15.72 | 0.00 | Single-study Wilson |
| Sample size ≥50 | *CYP2D6* | *36-*10 | — | NON-*36-*10 | *36-*10 | 11.40 | 8.15–15.72 | 1 | 136 | — | 11.40 | 8.15–15.72 | 0.00 | Single-study Wilson |
| Kinh-only samples | *CYP2D6* | *36-*10 | — | NON-*36-*10 | *36-*10 | 11.40 | 8.15–15.72 | 1 | 136 | — | 11.40 | 8.15–15.72 | 0.00 | Single-study Wilson |
| Strict sensitivity set | *CYP2D6* | *36-*36-*10 | — | NON-*36-*36-*10 | *36-*36-*10 | 0.37 | 0.07–2.05 | 1 | 136 | — | 0.37 | 0.06–2.05 | 0.00 | Single-study Wilson |
| Sample size ≥50 | *CYP2D6* | *36-*36-*10 | — | NON-*36-*36-*10 | *36-*36-*10 | 0.37 | 0.07–2.05 | 1 | 136 | — | 0.37 | 0.06–2.05 | 0.00 | Single-study Wilson |
| HWE passed or not informative | *CYP2D6* | *36-*36-*10 | — | NON-*36-*36-*10 | *36-*36-*10 | 0.37 | 0.07–2.05 | 1 | 136 | — | 0.37 | 0.06–2.05 | 0.00 | Single-study Wilson |
| Kinh-only samples | *CYP2D6* | *36-*36-*10 | — | NON-*36-*36-*10 | *36-*36-*10 | 0.37 | 0.07–2.05 | 1 | 136 | — | 0.37 | 0.06–2.05 | 0.00 | Single-study Wilson |
| Strict sensitivity set | *CYP2D6* | *4 | — | NON-*4 | *4 | 0.73 | 0.20–2.64 | 1 | 136 | — | 0.74 | 0.20–2.64 | 0.01 | Single-study Wilson |
| Strict sensitivity set | *CYP2D6* | rs3892097 G>A marker | rs3892097 | G | A | 1.45 | 0.40–5.13 | 1 | 69 | — | 1.45 | 0.40–5.13 | 0.00 | Single-study Wilson |
| Sample size ≥50 | *CYP2D6* | *4 | — | NON-*4 | *4 | 0.73 | 0.20–2.64 | 1 | 136 | — | 0.74 | 0.20–2.64 | 0.01 | Single-study Wilson |
| Sample size ≥50 | *CYP2D6* | rs3892097 G>A marker | rs3892097 | G | A | 1.45 | 0.40–5.13 | 1 | 69 | — | 1.45 | 0.40–5.13 | 0.00 | Single-study Wilson |
| Single-rsID representations only | *CYP2D6* | rs3892097 G>A marker | rs3892097 | G | A | 1.45 | 0.40–5.13 | 1 | 69 | — | 1.45 | 0.40–5.13 | 0.00 | Single-study Wilson |
| HWE passed or not informative | *CYP2D6* | *4 | — | NON-*4 | *4 | 0.73 | 0.20–2.64 | 1 | 136 | — | 0.74 | 0.20–2.64 | 0.01 | Single-study Wilson |
| HWE passed or not informative | *CYP2D6* | rs3892097 G>A marker | rs3892097 | G | A | 1.45 | 0.40–5.13 | 1 | 69 | — | 1.45 | 0.40–5.13 | 0.00 | Single-study Wilson |
| Kinh-only samples | *CYP2D6* | *4 | — | NON-*4 | *4 | 0.73 | 0.20–2.64 | 1 | 136 | — | 0.74 | 0.20–2.64 | 0.01 | Single-study Wilson |
| Kinh-only samples | *CYP2D6* | rs3892097 G>A marker | rs3892097 | G | A | 1.45 | 0.40–5.13 | 1 | 69 | — | 1.45 | 0.40–5.13 | 0.00 | Single-study Wilson |
| Strict sensitivity set | *CYP2D6* | *41 | — | NON-*41 | *41 | 4.37 | 2.46–7.65 | 1 | 126 | — | 4.37 | 2.45–7.65 | 0.00 | Single-study Wilson |
| Sample size ≥50 | *CYP2D6* | *41 | — | NON-*41 | *41 | 4.37 | 2.46–7.65 | 1 | 126 | — | 4.37 | 2.45–7.65 | 0.00 | Single-study Wilson |
| HWE passed or not informative | *CYP2D6* | *41 | — | NON-*41 | *41 | 4.37 | 2.46–7.65 | 1 | 126 | — | 4.37 | 2.45–7.65 | 0.00 | Single-study Wilson |
| Strict sensitivity set | *CYP2D6* | *49 | — | NON-*49 | *49 | 1.19 | 0.41–3.44 | 1 | 126 | — | 1.19 | 0.41–3.44 | 0.00 | Single-study Wilson |
| Sample size ≥50 | *CYP2D6* | *49 | — | NON-*49 | *49 | 1.19 | 0.41–3.44 | 1 | 126 | — | 1.19 | 0.41–3.44 | 0.00 | Single-study Wilson |
| HWE passed or not informative | *CYP2D6* | *49 | — | NON-*49 | *49 | 1.19 | 0.41–3.44 | 1 | 126 | — | 1.19 | 0.41–3.44 | 0.00 | Single-study Wilson |
| Strict sensitivity set | *CYP2D6* | *5 | — | WT | DEL | 7.75 | 4.38–13.34 | 1 | 71 | — | 7.75 | 4.38–13.34 | 0.00 | Single-study Wilson |
| Strict sensitivity set | *CYP2D6* | *5 | — | NON-*5 | *5 | 7.47 | 5.51–10.06 | 2 | 262 | 0.00 | 7.47 | 5.51–10.06 | 0.00 | Random-effects logit (DL) |
| Sample size ≥50 | *CYP2D6* | *5 | — | WT | DEL | 7.75 | 4.38–13.34 | 1 | 71 | — | 7.75 | 4.38–13.34 | 0.00 | Single-study Wilson |
| Sample size ≥50 | *CYP2D6* | *5 | — | NON-*5 | *5 | 7.47 | 5.51–10.06 | 2 | 262 | 0.00 | 7.47 | 5.51–10.06 | 0.00 | Random-effects logit (DL) |
| HWE passed or not informative | *CYP2D6* | *5 | — | WT | DEL | 7.75 | 4.38–13.34 | 1 | 71 | — | 7.75 | 4.38–13.34 | 0.00 | Single-study Wilson |
| HWE passed or not informative | *CYP2D6* | *5 | — | NON-*5 | *5 | 7.47 | 5.51–10.06 | 2 | 262 | 0.00 | 7.47 | 5.51–10.06 | 0.00 | Random-effects logit (DL) |
| Kinh-only samples | *CYP2D6* | *5 | — | WT | DEL | 7.75 | 4.38–13.34 | 1 | 71 | — | 7.75 | 4.38–13.34 | 0.00 | Single-study Wilson |
| Sample size ≥50 | *CYP2D6* | *60 | — | NON-*60 | *60 | 0.73 | 0.20–2.64 | 1 | 136 | — | 0.74 | 0.20–2.64 | 0.01 | Single-study Wilson |
| Kinh-only samples | *CYP2D6* | *60 | — | NON-*60 | *60 | 0.73 | 0.20–2.64 | 1 | 136 | — | 0.74 | 0.20–2.64 | 0.01 | Single-study Wilson |
| Strict sensitivity set | *CYP2D6* | *65 | — | NON-*65 | *65 | 2.94 | 1.50–5.70 | 1 | 136 | — | 2.94 | 1.50–5.70 | 0.00 | Single-study Wilson |
| Sample size ≥50 | *CYP2D6* | *65 | — | NON-*65 | *65 | 2.94 | 1.50–5.70 | 1 | 136 | — | 2.94 | 1.50–5.70 | 0.00 | Single-study Wilson |
| HWE passed or not informative | *CYP2D6* | *65 | — | NON-*65 | *65 | 2.94 | 1.50–5.70 | 1 | 136 | — | 2.94 | 1.50–5.70 | 0.00 | Single-study Wilson |
| Kinh-only samples | *CYP2D6* | *65 | — | NON-*65 | *65 | 2.94 | 1.50–5.70 | 1 | 136 | — | 2.94 | 1.50–5.70 | 0.00 | Single-study Wilson |
| Strict sensitivity set | *CYP2D6* | *67 | — | NON-*67 | *67 | 0.37 | 0.07–2.05 | 1 | 136 | — | 0.37 | 0.06–2.05 | 0.00 | Single-study Wilson |
| Sample size ≥50 | *CYP2D6* | *67 | — | NON-*67 | *67 | 0.37 | 0.07–2.05 | 1 | 136 | — | 0.37 | 0.06–2.05 | 0.00 | Single-study Wilson |
| HWE passed or not informative | *CYP2D6* | *67 | — | NON-*67 | *67 | 0.37 | 0.07–2.05 | 1 | 136 | — | 0.37 | 0.06–2.05 | 0.00 | Single-study Wilson |
| Kinh-only samples | *CYP2D6* | *67 | — | NON-*67 | *67 | 0.37 | 0.07–2.05 | 1 | 136 | — | 0.37 | 0.06–2.05 | 0.00 | Single-study Wilson |
| Strict sensitivity set | *CYP2D6* | *68-*4 | — | NON-*68-*4 | *68-*4 | 0.37 | 0.07–2.05 | 1 | 136 | — | 0.37 | 0.06–2.05 | 0.00 | Single-study Wilson |
| Sample size ≥50 | *CYP2D6* | *68-*4 | — | NON-*68-*4 | *68-*4 | 0.37 | 0.07–2.05 | 1 | 136 | — | 0.37 | 0.06–2.05 | 0.00 | Single-study Wilson |
| HWE passed or not informative | *CYP2D6* | *68-*4 | — | NON-*68-*4 | *68-*4 | 0.37 | 0.07–2.05 | 1 | 136 | — | 0.37 | 0.06–2.05 | 0.00 | Single-study Wilson |
| Kinh-only samples | *CYP2D6* | *68-*4 | — | NON-*68-*4 | *68-*4 | 0.37 | 0.07–2.05 | 1 | 136 | — | 0.37 | 0.06–2.05 | 0.00 | Single-study Wilson |
| Strict sensitivity set | *CYP2D6* | *86 | — | NON-*86 | *86 | 0.37 | 0.07–2.05 | 1 | 136 | — | 0.37 | 0.06–2.05 | 0.00 | Single-study Wilson |
| Sample size ≥50 | *CYP2D6* | *86 | — | NON-*86 | *86 | 0.37 | 0.07–2.05 | 1 | 136 | — | 0.37 | 0.06–2.05 | 0.00 | Single-study Wilson |
| HWE passed or not informative | *CYP2D6* | *86 | — | NON-*86 | *86 | 0.37 | 0.07–2.05 | 1 | 136 | — | 0.37 | 0.06–2.05 | 0.00 | Single-study Wilson |
| Kinh-only samples | *CYP2D6* | *86 | — | NON-*86 | *86 | 0.37 | 0.07–2.05 | 1 | 136 | — | 0.37 | 0.06–2.05 | 0.00 | Single-study Wilson |
| Sample size ≥50 | *CYP2D6* | G4268C | rs1135840 | G | C | 73.25 | 68.71–77.35 | 1 | 200 | — | 73.25 | 68.71–77.35 | 0.00 | Single-study Wilson |
| Single-rsID representations only | *CYP2D6* | G4268C | rs1135840 | G | C | 73.25 | 68.71–77.35 | 1 | 200 | — | 73.25 | 68.71–77.35 | 0.00 | Single-study Wilson |
| Healthy/control samples only | *CYP2D6* | G4268C | rs1135840 | G | C | 73.25 | 68.71–77.35 | 1 | 200 | — | 73.25 | 68.71–77.35 | 0.00 | Single-study Wilson |
| Strict sensitivity set | *CYP2E1* | *5B | rs2031920 | C | T | 17.19 | 12.51–23.15 | 1 | 96 | — | 17.19 | 12.51–23.15 | 0.00 | Single-study Wilson |
| Sample size ≥50 | *CYP2E1* | *5B | rs2031920 | C | T | 17.19 | 12.51–23.15 | 1 | 96 | — | 17.19 | 12.51–23.15 | 0.00 | Single-study Wilson |
| Single-rsID representations only | *CYP2E1* | *5B | rs2031920 | C | T | 17.19 | 12.51–23.15 | 1 | 96 | — | 17.19 | 12.51–23.15 | 0.00 | Single-study Wilson |
| HWE passed or not informative | *CYP2E1* | *5B | rs2031920 | C | T | 17.19 | 12.51–23.15 | 1 | 96 | — | 17.19 | 12.51–23.15 | 0.00 | Single-study Wilson |
| Strict sensitivity set | *CYP2E1* | *7B | rs6413420 | G | T | 0.00 | 0.00–1.96 | 1 | 96 | — | 0.00 | 0.00–1.96 | 0.00 | Single-study Wilson |
| Sample size ≥50 | *CYP2E1* | *7B | rs6413420 | G | T | 0.00 | 0.00–1.96 | 1 | 96 | — | 0.00 | 0.00–1.96 | 0.00 | Single-study Wilson |
| Single-rsID representations only | *CYP2E1* | *7B | rs6413420 | G | T | 0.00 | 0.00–1.96 | 1 | 96 | — | 0.00 | 0.00–1.96 | 0.00 | Single-study Wilson |
| HWE passed or not informative | *CYP2E1* | *7B | rs6413420 | G | T | 0.00 | 0.00–1.96 | 1 | 96 | — | 0.00 | 0.00–1.96 | 0.00 | Single-study Wilson |
| Strict sensitivity set | *CYP2E1* | Del | — | WT | DEL | 0.33 | 0.06–1.82 | 1 | 154 | — | 0.32 | 0.06–1.82 | 0.01 | Single-study Wilson |
| Sample size ≥50 | *CYP2E1* | Del | — | WT | DEL | 0.33 | 0.06–1.82 | 1 | 154 | — | 0.32 | 0.06–1.82 | 0.01 | Single-study Wilson |
| HWE passed or not informative | *CYP2E1* | Del | — | WT | DEL | 0.33 | 0.06–1.82 | 1 | 154 | — | 0.32 | 0.06–1.82 | 0.01 | Single-study Wilson |
| Kinh-only samples | *CYP2E1* | Del | — | WT | DEL | 0.33 | 0.06–1.82 | 1 | 154 | — | 0.32 | 0.06–1.82 | 0.01 | Single-study Wilson |
| Strict sensitivity set | *CYP2E1* | Dup | — | WT | DUP | 0.97 | 0.33–2.82 | 1 | 154 | — | 0.97 | 0.33–2.82 | 0.00 | Single-study Wilson |
| Sample size ≥50 | *CYP2E1* | Dup | — | WT | DUP | 0.97 | 0.33–2.82 | 1 | 154 | — | 0.97 | 0.33–2.82 | 0.00 | Single-study Wilson |
| HWE passed or not informative | *CYP2E1* | Dup | — | WT | DUP | 0.97 | 0.33–2.82 | 1 | 154 | — | 0.97 | 0.33–2.82 | 0.00 | Single-study Wilson |
| Kinh-only samples | *CYP2E1* | Dup | — | WT | DUP | 0.97 | 0.33–2.82 | 1 | 154 | — | 0.97 | 0.33–2.82 | 0.00 | Single-study Wilson |
| Strict sensitivity set | *CYP2J2* | *8 | — | G312 | R312 | 0.00 | 0.00–1.19 | 1 | 159 | — | 0.00 | 0.00–1.19 | 0.00 | Single-study Wilson |
| Sample size ≥50 | *CYP2J2* | *8 | — | G312 | R312 | 0.00 | 0.00–1.19 | 1 | 159 | — | 0.00 | 0.00–1.19 | 0.00 | Single-study Wilson |
| HWE passed or not informative | *CYP2J2* | *8 | — | G312 | R312 | 0.00 | 0.00–1.19 | 1 | 159 | — | 0.00 | 0.00–1.19 | 0.00 | Single-study Wilson |
| Kinh-only samples | *CYP2J2* | *8 | — | G312 | R312 | 0.00 | 0.00–1.19 | 1 | 159 | — | 0.00 | 0.00–1.19 | 0.00 | Single-study Wilson |
| Strict sensitivity set | *CYP2J2* | *9 | — | P351 | L351 | 0.00 | 0.00–1.19 | 1 | 159 | — | 0.00 | 0.00–1.19 | 0.00 | Single-study Wilson |
| Sample size ≥50 | *CYP2J2* | *9 | — | P351 | L351 | 0.00 | 0.00–1.19 | 1 | 159 | — | 0.00 | 0.00–1.19 | 0.00 | Single-study Wilson |
| HWE passed or not informative | *CYP2J2* | *9 | — | P351 | L351 | 0.00 | 0.00–1.19 | 1 | 159 | — | 0.00 | 0.00–1.19 | 0.00 | Single-study Wilson |
| Kinh-only samples | *CYP2J2* | *9 | — | P351 | L351 | 0.00 | 0.00–1.19 | 1 | 159 | — | 0.00 | 0.00–1.19 | 0.00 | Single-study Wilson |
| Strict sensitivity set | *CYP3A4* | *18 | — | NON-*18 | *18 | 0.79 | 0.22–2.83 | 1 | 127 | — | 0.79 | 0.22–2.83 | 0.00 | Single-study Wilson |
| Sample size ≥50 | *CYP3A4* | *18 | — | NON-*18 | *18 | 0.79 | 0.22–2.83 | 1 | 127 | — | 0.79 | 0.22–2.83 | 0.00 | Single-study Wilson |
| HWE passed or not informative | *CYP3A4* | *18 | — | NON-*18 | *18 | 0.79 | 0.22–2.83 | 1 | 127 | — | 0.79 | 0.22–2.83 | 0.00 | Single-study Wilson |
| Strict sensitivity set | *CYP3A4* | *1B | rs2740574 | A | G | 2.08 | 0.71–5.95 | 1 | 72 | — | 2.08 | 0.71–5.95 | 0.00 | Single-study Wilson |
| Sample size ≥50 | *CYP3A4* | *1B | rs2740574 | A | G | 2.08 | 0.71–5.95 | 1 | 72 | — | 2.08 | 0.71–5.95 | 0.00 | Single-study Wilson |
| Single-rsID representations only | *CYP3A4* | *1B | rs2740574 | A | G | 2.08 | 0.71–5.95 | 1 | 72 | — | 2.08 | 0.71–5.95 | 0.00 | Single-study Wilson |
| HWE passed or not informative | *CYP3A4* | *1B | rs2740574 | A | G | 2.08 | 0.71–5.95 | 1 | 72 | — | 2.08 | 0.71–5.95 | 0.00 | Single-study Wilson |
| Kinh-only samples | *CYP3A4* | *1B | rs2740574 | A | G | 2.08 | 0.71–5.95 | 1 | 72 | — | 2.08 | 0.71–5.95 | 0.00 | Single-study Wilson |
| Strict sensitivity set | *CYP3A5* | *3 | rs776746 | A | G | 60.81 | 44.96–74.67 | 7 | 543 | 95.54 | 60.81 | 44.96–74.67 | 0.00 | Random-effects logit (DL) |
| Single-rsID representations only | *CYP3A5* | *3 | rs776746 | A | G | 60.81 | 44.96–74.67 | 7 | 543 | 95.54 | 60.81 | 44.96–74.67 | 0.00 | Random-effects logit (DL) |
| Strict sensitivity set | *CYP3A5* | *6 | rs10264272 | G | A | 0.00 | 0.00–2.60 | 1 | 72 | — | 0.00 | 0.00–2.60 | 0.00 | Single-study Wilson |
| Sample size ≥50 | *CYP3A5* | *6 | rs10264272 | G | A | 0.00 | 0.00–2.60 | 1 | 72 | — | 0.00 | 0.00–2.60 | 0.00 | Single-study Wilson |
| Single-rsID representations only | *CYP3A5* | *6 | rs10264272 | G | A | 0.00 | 0.00–2.60 | 1 | 72 | — | 0.00 | 0.00–2.60 | 0.00 | Single-study Wilson |
| HWE passed or not informative | *CYP3A5* | *6 | rs10264272 | G | A | 0.00 | 0.00–2.60 | 1 | 72 | — | 0.00 | 0.00–2.60 | 0.00 | Single-study Wilson |
| Kinh-only samples | *CYP3A5* | *6 | rs10264272 | G | A | 0.00 | 0.00–2.60 | 1 | 72 | — | 0.00 | 0.00–2.60 | 0.00 | Single-study Wilson |
| Strict sensitivity set | *CYP3A5* | *7 | rs41303343 | T | TT | 0.00 | 0.00–2.60 | 1 | 72 | — | 0.00 | 0.00–2.60 | 0.00 | Single-study Wilson |
| Sample size ≥50 | *CYP3A5* | *7 | rs41303343 | T | TT | 0.00 | 0.00–2.60 | 1 | 72 | — | 0.00 | 0.00–2.60 | 0.00 | Single-study Wilson |
| Single-rsID representations only | *CYP3A5* | *7 | rs41303343 | T | TT | 0.00 | 0.00–2.60 | 1 | 72 | — | 0.00 | 0.00–2.60 | 0.00 | Single-study Wilson |
| HWE passed or not informative | *CYP3A5* | *7 | rs41303343 | T | TT | 0.00 | 0.00–2.60 | 1 | 72 | — | 0.00 | 0.00–2.60 | 0.00 | Single-study Wilson |
| Kinh-only samples | *CYP3A5* | *7 | rs41303343 | T | TT | 0.00 | 0.00–2.60 | 1 | 72 | — | 0.00 | 0.00–2.60 | 0.00 | Single-study Wilson |
| Strict sensitivity set | *CYP3A5* | *8 | rs55817950 | C | T | 0.00 | 0.00–2.60 | 1 | 72 | — | 0.00 | 0.00–2.60 | 0.00 | Single-study Wilson |
| Sample size ≥50 | *CYP3A5* | *8 | rs55817950 | C | T | 0.00 | 0.00–2.60 | 1 | 72 | — | 0.00 | 0.00–2.60 | 0.00 | Single-study Wilson |
| Single-rsID representations only | *CYP3A5* | *8 | rs55817950 | C | T | 0.00 | 0.00–2.60 | 1 | 72 | — | 0.00 | 0.00–2.60 | 0.00 | Single-study Wilson |
| HWE passed or not informative | *CYP3A5* | *8 | rs55817950 | C | T | 0.00 | 0.00–2.60 | 1 | 72 | — | 0.00 | 0.00–2.60 | 0.00 | Single-study Wilson |
| Kinh-only samples | *CYP3A5* | *8 | rs55817950 | C | T | 0.00 | 0.00–2.60 | 1 | 72 | — | 0.00 | 0.00–2.60 | 0.00 | Single-study Wilson |
| Strict sensitivity set | *CYP4F2* | rs2108622 | rs2108622 | C | T | 16.50 | 12.00–22.27 | 1 | 100 | — | 16.50 | 12.00–22.27 | 0.00 | Single-study Wilson |
| Sample size ≥50 | *CYP4F2* | rs2108622 | rs2108622 | C | T | 16.50 | 12.00–22.27 | 1 | 100 | — | 16.50 | 12.00–22.27 | 0.00 | Single-study Wilson |
| Single-rsID representations only | *CYP4F2* | rs2108622 | rs2108622 | C | T | 16.50 | 12.00–22.27 | 1 | 100 | — | 16.50 | 12.00–22.27 | 0.00 | Single-study Wilson |
| HWE passed or not informative | *CYP4F2* | rs2108622 | rs2108622 | C | T | 16.50 | 12.00–22.27 | 1 | 100 | — | 16.50 | 12.00–22.27 | 0.00 | Single-study Wilson |
| Kinh-only samples | *CYP4F2* | rs2108622 | rs2108622 | C | T | 16.50 | 12.00–22.27 | 1 | 100 | — | 16.50 | 12.00–22.27 | 0.00 | Single-study Wilson |
| Healthy/control samples only | *CYP4F2* | rs2108622 | rs2108622 | C | T | 16.50 | 12.00–22.27 | 1 | 100 | — | 16.50 | 12.00–22.27 | 0.00 | Single-study Wilson |
| Strict sensitivity set | *DPYD* | rs17376848 | rs17376848 | A | G | 16.00 | 11.57–21.71 | 1 | 100 | — | 16.00 | 11.57–21.71 | 0.00 | Single-study Wilson |
| Sample size ≥50 | *DPYD* | rs17376848 | rs17376848 | A | G | 16.00 | 11.57–21.71 | 1 | 100 | — | 16.00 | 11.57–21.71 | 0.00 | Single-study Wilson |
| Single-rsID representations only | *DPYD* | rs17376848 | rs17376848 | A | G | 16.00 | 11.57–21.71 | 1 | 100 | — | 16.00 | 11.57–21.71 | 0.00 | Single-study Wilson |
| HWE passed or not informative | *DPYD* | rs17376848 | rs17376848 | A | G | 16.00 | 11.57–21.71 | 1 | 100 | — | 16.00 | 11.57–21.71 | 0.00 | Single-study Wilson |
| Kinh-only samples | *DPYD* | rs17376848 | rs17376848 | A | G | 16.00 | 11.57–21.71 | 1 | 100 | — | 16.00 | 11.57–21.71 | 0.00 | Single-study Wilson |
| Healthy/control samples only | *DPYD* | rs17376848 | rs17376848 | A | G | 16.00 | 11.57–21.71 | 1 | 100 | — | 16.00 | 11.57–21.71 | 0.00 | Single-study Wilson |
| Strict sensitivity set | *DPYD* | rs1801159 | rs1801159 | T | C | 22.50 | 17.26–28.77 | 1 | 100 | — | 22.50 | 17.26–28.77 | 0.00 | Single-study Wilson |
| Sample size ≥50 | *DPYD* | rs1801159 | rs1801159 | T | C | 22.50 | 17.26–28.77 | 1 | 100 | — | 22.50 | 17.26–28.77 | 0.00 | Single-study Wilson |
| Single-rsID representations only | *DPYD* | rs1801159 | rs1801159 | T | C | 22.50 | 17.26–28.77 | 1 | 100 | — | 22.50 | 17.26–28.77 | 0.00 | Single-study Wilson |
| HWE passed or not informative | *DPYD* | rs1801159 | rs1801159 | T | C | 22.50 | 17.26–28.77 | 1 | 100 | — | 22.50 | 17.26–28.77 | 0.00 | Single-study Wilson |
| Kinh-only samples | *DPYD* | rs1801159 | rs1801159 | T | C | 22.50 | 17.26–28.77 | 1 | 100 | — | 22.50 | 17.26–28.77 | 0.00 | Single-study Wilson |
| Healthy/control samples only | *DPYD* | rs1801159 | rs1801159 | T | C | 22.50 | 17.26–28.77 | 1 | 100 | — | 22.50 | 17.26–28.77 | 0.00 | Single-study Wilson |
| Strict sensitivity set | *DPYD* | rs1801160 | rs1801160 | C | T | 2.00 | 0.78–5.03 | 1 | 100 | — | 2.00 | 0.78–5.03 | 0.00 | Single-study Wilson |
| Sample size ≥50 | *DPYD* | rs1801160 | rs1801160 | C | T | 2.00 | 0.78–5.03 | 1 | 100 | — | 2.00 | 0.78–5.03 | 0.00 | Single-study Wilson |
| Single-rsID representations only | *DPYD* | rs1801160 | rs1801160 | C | T | 2.00 | 0.78–5.03 | 1 | 100 | — | 2.00 | 0.78–5.03 | 0.00 | Single-study Wilson |
| HWE passed or not informative | *DPYD* | rs1801160 | rs1801160 | C | T | 2.00 | 0.78–5.03 | 1 | 100 | — | 2.00 | 0.78–5.03 | 0.00 | Single-study Wilson |
| Kinh-only samples | *DPYD* | rs1801160 | rs1801160 | C | T | 2.00 | 0.78–5.03 | 1 | 100 | — | 2.00 | 0.78–5.03 | 0.00 | Single-study Wilson |
| Healthy/control samples only | *DPYD* | rs1801160 | rs1801160 | C | T | 2.00 | 0.78–5.03 | 1 | 100 | — | 2.00 | 0.78–5.03 | 0.00 | Single-study Wilson |
| Strict sensitivity set | *DPYD* | rs1801265 | rs1801265 | A | G | 17.00 | 12.43–22.82 | 1 | 100 | — | 17.00 | 12.43–22.82 | 0.00 | Single-study Wilson |
| Sample size ≥50 | *DPYD* | rs1801265 | rs1801265 | A | G | 17.00 | 12.43–22.82 | 1 | 100 | — | 17.00 | 12.43–22.82 | 0.00 | Single-study Wilson |
| Single-rsID representations only | *DPYD* | rs1801265 | rs1801265 | A | G | 17.00 | 12.43–22.82 | 1 | 100 | — | 17.00 | 12.43–22.82 | 0.00 | Single-study Wilson |
| HWE passed or not informative | *DPYD* | rs1801265 | rs1801265 | A | G | 17.00 | 12.43–22.82 | 1 | 100 | — | 17.00 | 12.43–22.82 | 0.00 | Single-study Wilson |
| Kinh-only samples | *DPYD* | rs1801265 | rs1801265 | A | G | 17.00 | 12.43–22.82 | 1 | 100 | — | 17.00 | 12.43–22.82 | 0.00 | Single-study Wilson |
| Healthy/control samples only | *DPYD* | rs1801265 | rs1801265 | A | G | 17.00 | 12.43–22.82 | 1 | 100 | — | 17.00 | 12.43–22.82 | 0.00 | Single-study Wilson |
| Strict sensitivity set | *DPYD* | rs2297595 | rs2297595 | T | C | 2.50 | 1.07–5.72 | 1 | 100 | — | 2.50 | 1.07–5.72 | 0.00 | Single-study Wilson |
| Sample size ≥50 | *DPYD* | rs2297595 | rs2297595 | T | C | 2.50 | 1.07–5.72 | 1 | 100 | — | 2.50 | 1.07–5.72 | 0.00 | Single-study Wilson |
| Single-rsID representations only | *DPYD* | rs2297595 | rs2297595 | T | C | 2.50 | 1.07–5.72 | 1 | 100 | — | 2.50 | 1.07–5.72 | 0.00 | Single-study Wilson |
| HWE passed or not informative | *DPYD* | rs2297595 | rs2297595 | T | C | 2.50 | 1.07–5.72 | 1 | 100 | — | 2.50 | 1.07–5.72 | 0.00 | Single-study Wilson |
| Kinh-only samples | *DPYD* | rs2297595 | rs2297595 | T | C | 2.50 | 1.07–5.72 | 1 | 100 | — | 2.50 | 1.07–5.72 | 0.00 | Single-study Wilson |
| Healthy/control samples only | *DPYD* | rs2297595 | rs2297595 | T | C | 2.50 | 1.07–5.72 | 1 | 100 | — | 2.50 | 1.07–5.72 | 0.00 | Single-study Wilson |
| Strict sensitivity set | *HLA-B* | *58:01 | — | *X | *58:01 | 6.01 | 3.74–9.55 | 1 | 133 | — | 6.02 | 3.74–9.55 | 0.01 | Single-study Wilson |
| Sample size ≥50 | *HLA-B* | *58:01 | — | *X | *58:01 | 6.01 | 3.74–9.55 | 1 | 133 | — | 6.02 | 3.74–9.55 | 0.01 | Single-study Wilson |
| HWE passed or not informative | *HLA-B* | *58:01 | — | *X | *58:01 | 6.01 | 3.74–9.55 | 1 | 133 | — | 6.02 | 3.74–9.55 | 0.01 | Single-study Wilson |
| Strict sensitivity set | *HNF4A* | G36S | — | G36 | S36 | 3.60 | 1.97–6.49 | 1 | 139 | — | 3.60 | 1.97–6.49 | 0.00 | Single-study Wilson |
| Sample size ≥50 | *HNF4A* | G36S | — | G36 | S36 | 3.60 | 1.97–6.49 | 1 | 139 | — | 3.60 | 1.97–6.49 | 0.00 | Single-study Wilson |
| HWE passed or not informative | *HNF4A* | G36S | — | G36 | S36 | 3.60 | 1.97–6.49 | 1 | 139 | — | 3.60 | 1.97–6.49 | 0.00 | Single-study Wilson |
| Kinh-only samples | *HNF4A* | G36S | — | G36 | S36 | 3.60 | 1.97–6.49 | 1 | 139 | — | 3.60 | 1.97–6.49 | 0.00 | Single-study Wilson |
| Strict sensitivity set | *HNF4A* | G60D | — | G60 | D60 | 0.72 | 0.20–2.58 | 1 | 139 | — | 0.72 | 0.20–2.58 | 0.00 | Single-study Wilson |
| Sample size ≥50 | *HNF4A* | G60D | — | G60 | D60 | 0.72 | 0.20–2.58 | 1 | 139 | — | 0.72 | 0.20–2.58 | 0.00 | Single-study Wilson |
| HWE passed or not informative | *HNF4A* | G60D | — | G60 | D60 | 0.72 | 0.20–2.58 | 1 | 139 | — | 0.72 | 0.20–2.58 | 0.00 | Single-study Wilson |
| Kinh-only samples | *HNF4A* | G60D | — | G60 | D60 | 0.72 | 0.20–2.58 | 1 | 139 | — | 0.72 | 0.20–2.58 | 0.00 | Single-study Wilson |
| Strict sensitivity set | *NAT2* | *12 | — | NON-*12 | *12 | 3.47 | 1.49–7.87 | 1 | 72 | — | 3.47 | 1.49–7.87 | 0.00 | Single-study Wilson |
| Sample size ≥50 | *NAT2* | *12 | — | NON-*12 | *12 | 3.47 | 1.49–7.87 | 1 | 72 | — | 3.47 | 1.49–7.87 | 0.00 | Single-study Wilson |
| HWE passed or not informative | *NAT2* | *12 | — | NON-*12 | *12 | 3.47 | 1.49–7.87 | 1 | 72 | — | 3.47 | 1.49–7.87 | 0.00 | Single-study Wilson |
| Kinh-only samples | *NAT2* | *12 | — | NON-*12 | *12 | 3.47 | 1.49–7.87 | 1 | 72 | — | 3.47 | 1.49–7.87 | 0.00 | Single-study Wilson |
| Strict sensitivity set | *NAT2* | *13 | — | NON-*13 | *13 | 30.56 | 23.62–38.50 | 1 | 72 | — | 30.56 | 23.62–38.50 | 0.00 | Single-study Wilson |
| Sample size ≥50 | *NAT2* | *13 | — | NON-*13 | *13 | 30.56 | 23.62–38.50 | 1 | 72 | — | 30.56 | 23.62–38.50 | 0.00 | Single-study Wilson |
| HWE passed or not informative | *NAT2* | *13 | — | NON-*13 | *13 | 30.56 | 23.62–38.50 | 1 | 72 | — | 30.56 | 23.62–38.50 | 0.00 | Single-study Wilson |
| Kinh-only samples | *NAT2* | *13 | — | NON-*13 | *13 | 30.56 | 23.62–38.50 | 1 | 72 | — | 30.56 | 23.62–38.50 | 0.00 | Single-study Wilson |
| Strict sensitivity set | *NAT2* | *19 | — | NON-*19 | *19 | 1.39 | 0.38–4.92 | 1 | 72 | — | 1.39 | 0.38–4.92 | 0.00 | Single-study Wilson |
| Sample size ≥50 | *NAT2* | *19 | — | NON-*19 | *19 | 1.39 | 0.38–4.92 | 1 | 72 | — | 1.39 | 0.38–4.92 | 0.00 | Single-study Wilson |
| HWE passed or not informative | *NAT2* | *19 | — | NON-*19 | *19 | 1.39 | 0.38–4.92 | 1 | 72 | — | 1.39 | 0.38–4.92 | 0.00 | Single-study Wilson |
| Kinh-only samples | *NAT2* | *19 | — | NON-*19 | *19 | 1.39 | 0.38–4.92 | 1 | 72 | — | 1.39 | 0.38–4.92 | 0.00 | Single-study Wilson |
| Strict sensitivity set | *NAT2* | *5 | — | NON-*5 | *5 | 2.78 | 1.08–6.92 | 1 | 72 | — | 2.78 | 1.09–6.92 | 0.00 | Single-study Wilson |
| Strict sensitivity set | *NAT2* | *5 | rs1801280 | T | C | 5.97 | 3.96–8.89 | 4 | 457 | 54.94 | 5.97 | 3.96–8.89 | 0.00 | Random-effects logit (DL) |
| Sample size ≥50 | *NAT2* | *5 | — | NON-*5 | *5 | 2.78 | 1.08–6.92 | 1 | 72 | — | 2.78 | 1.09–6.92 | 0.00 | Single-study Wilson |
| Sample size ≥50 | *NAT2* | *5 | rs1801280 | T | C | 5.97 | 3.96–8.89 | 4 | 457 | 54.94 | 5.97 | 3.96–8.89 | 0.00 | Random-effects logit (DL) |
| Single-rsID representations only | *NAT2* | *5 | rs1801280 | T | C | 5.97 | 3.96–8.89 | 4 | 457 | 54.94 | 5.97 | 3.96–8.89 | 0.00 | Random-effects logit (DL) |
| HWE passed or not informative | *NAT2* | *5 | — | NON-*5 | *5 | 2.78 | 1.08–6.92 | 1 | 72 | — | 2.78 | 1.09–6.92 | 0.00 | Single-study Wilson |
| HWE passed or not informative | *NAT2* | *5 | rs1801280 | T | C | 5.97 | 3.96–8.89 | 4 | 457 | 54.94 | 5.97 | 3.96–8.89 | 0.00 | Random-effects logit (DL) |
| Kinh-only samples | *NAT2* | *5 | — | NON-*5 | *5 | 2.78 | 1.08–6.92 | 1 | 72 | — | 2.78 | 1.09–6.92 | 0.00 | Single-study Wilson |
| Strict sensitivity set | *NAT2* | *6 | rs1799930 | G | A | 27.00 | 18.69–37.31 | 4 | 457 | 90.11 | 27.00 | 18.69–37.31 | 0.00 | Random-effects logit (DL) |
| Sample size ≥50 | *NAT2* | *6 | rs1799930 | G | A | 27.00 | 18.69–37.31 | 4 | 457 | 90.11 | 27.00 | 18.69–37.31 | 0.00 | Random-effects logit (DL) |
| Single-rsID representations only | *NAT2* | *6 | rs1799930 | G | A | 27.00 | 18.69–37.31 | 4 | 457 | 90.11 | 27.00 | 18.69–37.31 | 0.00 | Random-effects logit (DL) |
| Sample size ≥50 | *NAT2* | *6A | — | NON-*6A | *6A | 17.36 | 12.04–24.37 | 1 | 72 | — | 17.36 | 12.04–24.37 | 0.00 | Single-study Wilson |
| Kinh-only samples | *NAT2* | *6A | — | NON-*6A | *6A | 17.36 | 12.04–24.37 | 1 | 72 | — | 17.36 | 12.04–24.37 | 0.00 | Single-study Wilson |
| Strict sensitivity set | *NAT2* | *7 | — | NON-*7 | *7 | 0.00 | 0.00–2.60 | 1 | 72 | — | 0.00 | 0.00–2.60 | 0.00 | Single-study Wilson |
| Strict sensitivity set | *NAT2* | *7 | rs1799931 | G | A | 17.75 | 13.51–22.95 | 4 | 457 | 71.71 | 17.75 | 13.51–22.95 | 0.00 | Random-effects logit (DL) |
| Sample size ≥50 | *NAT2* | *7 | — | NON-*7 | *7 | 0.00 | 0.00–2.60 | 1 | 72 | — | 0.00 | 0.00–2.60 | 0.00 | Single-study Wilson |
| Sample size ≥50 | *NAT2* | *7 | rs1799931 | G | A | 17.75 | 13.51–22.95 | 4 | 457 | 71.71 | 17.75 | 13.51–22.95 | 0.00 | Random-effects logit (DL) |
| Single-rsID representations only | *NAT2* | *7 | rs1799931 | G | A | 17.75 | 13.51–22.95 | 4 | 457 | 71.71 | 17.75 | 13.51–22.95 | 0.00 | Random-effects logit (DL) |
| HWE passed or not informative | *NAT2* | *7 | — | NON-*7 | *7 | 0.00 | 0.00–2.60 | 1 | 72 | — | 0.00 | 0.00–2.60 | 0.00 | Single-study Wilson |
| HWE passed or not informative | *NAT2* | *7 | rs1799931 | G | A | 17.75 | 13.51–22.95 | 4 | 457 | 71.71 | 17.75 | 13.51–22.95 | 0.00 | Random-effects logit (DL) |
| Kinh-only samples | *NAT2* | *7 | — | NON-*7 | *7 | 0.00 | 0.00–2.60 | 1 | 72 | — | 0.00 | 0.00–2.60 | 0.00 | Single-study Wilson |
| Strict sensitivity set | *NAT2* | rs1208 | rs1208 | G | A | 91.00 | 86.22–94.23 | 1 | 100 | — | 91.00 | 86.22–94.23 | 0.00 | Single-study Wilson |
| Sample size ≥50 | *NAT2* | rs1208 | rs1208 | G | A | 91.00 | 86.22–94.23 | 1 | 100 | — | 91.00 | 86.22–94.23 | 0.00 | Single-study Wilson |
| Single-rsID representations only | *NAT2* | rs1208 | rs1208 | G | A | 91.00 | 86.22–94.23 | 1 | 100 | — | 91.00 | 86.22–94.23 | 0.00 | Single-study Wilson |
| HWE passed or not informative | *NAT2* | rs1208 | rs1208 | G | A | 91.00 | 86.22–94.23 | 1 | 100 | — | 91.00 | 86.22–94.23 | 0.00 | Single-study Wilson |
| Kinh-only samples | *NAT2* | rs1208 | rs1208 | G | A | 91.00 | 86.22–94.23 | 1 | 100 | — | 91.00 | 86.22–94.23 | 0.00 | Single-study Wilson |
| Healthy/control samples only | *NAT2* | rs1208 | rs1208 | G | A | 91.00 | 86.22–94.23 | 1 | 100 | — | 91.00 | 86.22–94.23 | 0.00 | Single-study Wilson |
| Strict sensitivity set | *NUDT15* | *2;*3 | rs116855232 | C | T | 4.50 | 2.39–8.33 | 1 | 100 | — | 4.50 | 2.39–8.33 | 0.00 | Single-study Wilson |
| Sample size ≥50 | *NUDT15* | *2;*3 | rs116855232 | C | T | 4.50 | 2.39–8.33 | 1 | 100 | — | 4.50 | 2.39–8.33 | 0.00 | Single-study Wilson |
| Single-rsID representations only | *NUDT15* | *2;*3 | rs116855232 | C | T | 4.50 | 2.39–8.33 | 1 | 100 | — | 4.50 | 2.39–8.33 | 0.00 | Single-study Wilson |
| HWE passed or not informative | *NUDT15* | *2;*3 | rs116855232 | C | T | 4.50 | 2.39–8.33 | 1 | 100 | — | 4.50 | 2.39–8.33 | 0.00 | Single-study Wilson |
| Kinh-only samples | *NUDT15* | *2;*3 | rs116855232 | C | T | 4.50 | 2.39–8.33 | 1 | 100 | — | 4.50 | 2.39–8.33 | 0.00 | Single-study Wilson |
| Healthy/control samples only | *NUDT15* | *2;*3 | rs116855232 | C | T | 4.50 | 2.39–8.33 | 1 | 100 | — | 4.50 | 2.39–8.33 | 0.00 | Single-study Wilson |
| Strict sensitivity set | *PON1* | Q192R | rs662 | C | T | 33.08 | 28.63–37.86 | 1 | 198 | — | 33.08 | 28.63–37.86 | 0.00 | Single-study Wilson |
| Sample size ≥50 | *PON1* | Q192R | rs662 | C | T | 33.08 | 28.63–37.86 | 1 | 198 | — | 33.08 | 28.63–37.86 | 0.00 | Single-study Wilson |
| Single-rsID representations only | *PON1* | Q192R | rs662 | C | T | 33.08 | 28.63–37.86 | 1 | 198 | — | 33.08 | 28.63–37.86 | 0.00 | Single-study Wilson |
| HWE passed or not informative | *PON1* | Q192R | rs662 | C | T | 33.08 | 28.63–37.86 | 1 | 198 | — | 33.08 | 28.63–37.86 | 0.00 | Single-study Wilson |
| Strict sensitivity set | *PSORS1C1* | rs9263726 | rs9263726 | G | A | 7.89 | 5.22–11.77 | 1 | 133 | — | 7.89 | 5.22–11.77 | 0.00 | Single-study Wilson |
| Sample size ≥50 | *PSORS1C1* | rs9263726 | rs9263726 | G | A | 7.89 | 5.22–11.77 | 1 | 133 | — | 7.89 | 5.22–11.77 | 0.00 | Single-study Wilson |
| Single-rsID representations only | *PSORS1C1* | rs9263726 | rs9263726 | G | A | 7.89 | 5.22–11.77 | 1 | 133 | — | 7.89 | 5.22–11.77 | 0.00 | Single-study Wilson |
| HWE passed or not informative | *PSORS1C1* | rs9263726 | rs9263726 | G | A | 7.89 | 5.22–11.77 | 1 | 133 | — | 7.89 | 5.22–11.77 | 0.00 | Single-study Wilson |
| Strict sensitivity set | *SLCO1B1* | *15 | rs4149056 | T | C | 16.35 | 11.94–21.97 | 1 | 104 | — | 16.35 | 11.94–21.97 | 0.00 | Single-study Wilson |
| Sample size ≥50 | *SLCO1B1* | *15 | rs4149056 | T | C | 16.35 | 11.94–21.97 | 1 | 104 | — | 16.35 | 11.94–21.97 | 0.00 | Single-study Wilson |
| Single-rsID representations only | *SLCO1B1* | *15 | rs4149056 | T | C | 16.35 | 11.94–21.97 | 1 | 104 | — | 16.35 | 11.94–21.97 | 0.00 | Single-study Wilson |
| HWE passed or not informative | *SLCO1B1* | *15 | rs4149056 | T | C | 16.35 | 11.94–21.97 | 1 | 104 | — | 16.35 | 11.94–21.97 | 0.00 | Single-study Wilson |
| Kinh-only samples | *SLCO1B1* | *15 | rs4149056 | T | C | 16.35 | 11.94–21.97 | 1 | 104 | — | 16.35 | 11.94–21.97 | 0.00 | Single-study Wilson |
| Healthy/control samples only | *SLCO1B1* | *15 | rs4149056 | T | C | 16.35 | 11.94–21.97 | 1 | 104 | — | 16.35 | 11.94–21.97 | 0.00 | Single-study Wilson |
| Strict sensitivity set | *SLCO1B1* | *5 | rs4149056 | T | C | 0.00 | 0.00–1.81 | 1 | 104 | — | 0.00 | 0.00–1.81 | 0.00 | Single-study Wilson |
| Sample size ≥50 | *SLCO1B1* | *5 | rs4149056 | T | C | 0.00 | 0.00–1.81 | 1 | 104 | — | 0.00 | 0.00–1.81 | 0.00 | Single-study Wilson |
| Single-rsID representations only | *SLCO1B1* | *5 | rs4149056 | T | C | 0.00 | 0.00–1.81 | 1 | 104 | — | 0.00 | 0.00–1.81 | 0.00 | Single-study Wilson |
| HWE passed or not informative | *SLCO1B1* | *5 | rs4149056 | T | C | 0.00 | 0.00–1.81 | 1 | 104 | — | 0.00 | 0.00–1.81 | 0.00 | Single-study Wilson |
| Kinh-only samples | *SLCO1B1* | *5 | rs4149056 | T | C | 0.00 | 0.00–1.81 | 1 | 104 | — | 0.00 | 0.00–1.81 | 0.00 | Single-study Wilson |
| Healthy/control samples only | *SLCO1B1* | *5 | rs4149056 | T | C | 0.00 | 0.00–1.81 | 1 | 104 | — | 0.00 | 0.00–1.81 | 0.00 | Single-study Wilson |
| Strict sensitivity set | *SLCO1B1* | c.388A>G | rs2306283 | A | G | 78.85 | 72.80–83.85 | 1 | 104 | — | 78.85 | 72.80–83.85 | 0.00 | Single-study Wilson |
| Sample size ≥50 | *SLCO1B1* | c.388A>G | rs2306283 | A | G | 78.85 | 72.80–83.85 | 1 | 104 | — | 78.85 | 72.80–83.85 | 0.00 | Single-study Wilson |
| Single-rsID representations only | *SLCO1B1* | c.388A>G | rs2306283 | A | G | 78.85 | 72.80–83.85 | 1 | 104 | — | 78.85 | 72.80–83.85 | 0.00 | Single-study Wilson |
| HWE passed or not informative | *SLCO1B1* | c.388A>G | rs2306283 | A | G | 78.85 | 72.80–83.85 | 1 | 104 | — | 78.85 | 72.80–83.85 | 0.00 | Single-study Wilson |
| Kinh-only samples | *SLCO1B1* | c.388A>G | rs2306283 | A | G | 78.85 | 72.80–83.85 | 1 | 104 | — | 78.85 | 72.80–83.85 | 0.00 | Single-study Wilson |
| Healthy/control samples only | *SLCO1B1* | c.388A>G | rs2306283 | A | G | 78.85 | 72.80–83.85 | 1 | 104 | — | 78.85 | 72.80–83.85 | 0.00 | Single-study Wilson |
| Strict sensitivity set | *SLCO1B1* | c.521T>C | rs4149056 | T | C | 12.73 | 7.36–21.13 | 2 | 204 | 75.83 | 12.73 | 7.36–21.13 | 0.00 | Random-effects logit (DL) |
| Sample size ≥50 | *SLCO1B1* | c.521T>C | rs4149056 | T | C | 12.73 | 7.36–21.13 | 2 | 204 | 75.83 | 12.73 | 7.36–21.13 | 0.00 | Random-effects logit (DL) |
| Single-rsID representations only | *SLCO1B1* | c.521T>C | rs4149056 | T | C | 12.73 | 7.36–21.13 | 2 | 204 | 75.83 | 12.73 | 7.36–21.13 | 0.00 | Random-effects logit (DL) |
| HWE passed or not informative | *SLCO1B1* | c.521T>C | rs4149056 | T | C | 12.73 | 7.36–21.13 | 2 | 204 | 75.83 | 12.73 | 7.36–21.13 | 0.00 | Random-effects logit (DL) |
| Kinh-only samples | *SLCO1B1* | c.521T>C | rs4149056 | T | C | 12.73 | 7.36–21.13 | 2 | 204 | 75.83 | 12.73 | 7.36–21.13 | 0.00 | Random-effects logit (DL) |
| Healthy/control samples only | *SLCO1B1* | c.521T>C | rs4149056 | T | C | 12.73 | 7.36–21.13 | 2 | 204 | 75.83 | 12.73 | 7.36–21.13 | 0.00 | Random-effects logit (DL) |
| Strict sensitivity set | *SLCO1B1* | rs2306283 | rs2306283 | A | G | 73.00 | 66.46–78.68 | 1 | 100 | — | 73.00 | 66.46–78.68 | 0.00 | Single-study Wilson |
| Sample size ≥50 | *SLCO1B1* | rs2306283 | rs2306283 | A | G | 73.00 | 66.46–78.68 | 1 | 100 | — | 73.00 | 66.46–78.68 | 0.00 | Single-study Wilson |
| Single-rsID representations only | *SLCO1B1* | rs2306283 | rs2306283 | A | G | 73.00 | 66.46–78.68 | 1 | 100 | — | 73.00 | 66.46–78.68 | 0.00 | Single-study Wilson |
| HWE passed or not informative | *SLCO1B1* | rs2306283 | rs2306283 | A | G | 73.00 | 66.46–78.68 | 1 | 100 | — | 73.00 | 66.46–78.68 | 0.00 | Single-study Wilson |
| Kinh-only samples | *SLCO1B1* | rs2306283 | rs2306283 | A | G | 73.00 | 66.46–78.68 | 1 | 100 | — | 73.00 | 66.46–78.68 | 0.00 | Single-study Wilson |
| Healthy/control samples only | *SLCO1B1* | rs2306283 | rs2306283 | A | G | 73.00 | 66.46–78.68 | 1 | 100 | — | 73.00 | 66.46–78.68 | 0.00 | Single-study Wilson |
| Strict sensitivity set | *TPMT* | rs1142345 | rs1142345 | T | C | 2.50 | 1.07–5.72 | 1 | 100 | — | 2.50 | 1.07–5.72 | 0.00 | Single-study Wilson |
| Sample size ≥50 | *TPMT* | rs1142345 | rs1142345 | T | C | 2.50 | 1.07–5.72 | 1 | 100 | — | 2.50 | 1.07–5.72 | 0.00 | Single-study Wilson |
| Single-rsID representations only | *TPMT* | rs1142345 | rs1142345 | T | C | 2.50 | 1.07–5.72 | 1 | 100 | — | 2.50 | 1.07–5.72 | 0.00 | Single-study Wilson |
| HWE passed or not informative | *TPMT* | rs1142345 | rs1142345 | T | C | 2.50 | 1.07–5.72 | 1 | 100 | — | 2.50 | 1.07–5.72 | 0.00 | Single-study Wilson |
| Kinh-only samples | *TPMT* | rs1142345 | rs1142345 | T | C | 2.50 | 1.07–5.72 | 1 | 100 | — | 2.50 | 1.07–5.72 | 0.00 | Single-study Wilson |
| Healthy/control samples only | *TPMT* | rs1142345 | rs1142345 | T | C | 2.50 | 1.07–5.72 | 1 | 100 | — | 2.50 | 1.07–5.72 | 0.00 | Single-study Wilson |
| Strict sensitivity set | *UGT1A1* | *28 | rs8175347 | *1 [(TA)6TAA] | *28 [(TA)7TAA] | 8.42 | 5.25–13.24 | 1 | 95 | — | 8.42 | 5.25–13.24 | 0.00 | Single-study Wilson |
| Strict sensitivity set | *UGT1A1* | *28 | rs8175347 | TA6 | TA7 | 6.00 | 2.78–12.48 | 1 | 50 | — | 6.00 | 2.78–12.48 | 0.00 | Single-study Wilson |
| Sample size ≥50 | *UGT1A1* | *28 | rs8175347 | *1 [(TA)6TAA] | *28 [(TA)7TAA] | 8.42 | 5.25–13.24 | 1 | 95 | — | 8.42 | 5.25–13.24 | 0.00 | Single-study Wilson |
| Sample size ≥50 | *UGT1A1* | *28 | rs8175347 | TA6 | TA7 | 6.00 | 2.78–12.48 | 1 | 50 | — | 6.00 | 2.78–12.48 | 0.00 | Single-study Wilson |
| Single-rsID representations only | *UGT1A1* | *28 | rs8175347 | *1 [(TA)6TAA] | *28 [(TA)7TAA] | 8.42 | 5.25–13.24 | 1 | 95 | — | 8.42 | 5.25–13.24 | 0.00 | Single-study Wilson |
| Single-rsID representations only | *UGT1A1* | *28 | rs8175347 | TA6 | TA7 | 6.00 | 2.78–12.48 | 1 | 50 | — | 6.00 | 2.78–12.48 | 0.00 | Single-study Wilson |
| HWE passed or not informative | *UGT1A1* | *28 | rs8175347 | *1 [(TA)6TAA] | *28 [(TA)7TAA] | 8.42 | 5.25–13.24 | 1 | 95 | — | 8.42 | 5.25–13.24 | 0.00 | Single-study Wilson |
| Kinh-only samples | *UGT1A1* | *28 | rs8175347 | *1 [(TA)6TAA] | *28 [(TA)7TAA] | 8.42 | 5.25–13.24 | 1 | 95 | — | 8.42 | 5.25–13.24 | 0.00 | Single-study Wilson |
| Healthy/control samples only | *UGT1A1* | *28 | rs8175347 | *1 [(TA)6TAA] | *28 [(TA)7TAA] | 8.42 | 5.25–13.24 | 1 | 95 | — | 8.42 | 5.25–13.24 | 0.00 | Single-study Wilson |
| Strict sensitivity set | *UGT1A1* | *6 | rs4148323 | G | A | 8.76 | 3.38–20.88 | 2 | 150 | 76.61 | 8.76 | 3.38–20.88 | 0.00 | Random-effects logit (DL) |
| Sample size ≥50 | *UGT1A1* | *6 | rs4148323 | G | A | 8.76 | 3.38–20.88 | 2 | 150 | 76.61 | 8.76 | 3.38–20.88 | 0.00 | Random-effects logit (DL) |
| Single-rsID representations only | *UGT1A1* | *6 | rs4148323 | G | A | 8.76 | 3.38–20.88 | 2 | 150 | 76.61 | 8.76 | 3.38–20.88 | 0.00 | Random-effects logit (DL) |
| HWE passed or not informative | *UGT1A1* | *6 | rs4148323 | G | A | 8.76 | 3.38–20.88 | 2 | 150 | 76.61 | 8.76 | 3.38–20.88 | 0.00 | Random-effects logit (DL) |
| Strict sensitivity set | *UGT1A1* | c.189C>T | rs191471887 | C | T | 0.00 | 0.00–3.70 | 1 | 50 | — | 0.00 | 0.00–3.70 | 0.00 | Single-study Wilson |
| Sample size ≥50 | *UGT1A1* | c.189C>T | rs191471887 | C | T | 0.00 | 0.00–3.70 | 1 | 50 | — | 0.00 | 0.00–3.70 | 0.00 | Single-study Wilson |
| Single-rsID representations only | *UGT1A1* | c.189C>T | rs191471887 | C | T | 0.00 | 0.00–3.70 | 1 | 50 | — | 0.00 | 0.00–3.70 | 0.00 | Single-study Wilson |
| HWE passed or not informative | *UGT1A1* | c.189C>T | rs191471887 | C | T | 0.00 | 0.00–3.70 | 1 | 50 | — | 0.00 | 0.00–3.70 | 0.00 | Single-study Wilson |
| Strict sensitivity set | *UGT1A1* | rs1042640C>G | rs1042640 | C | G | 25.00 | 19.51–31.43 | 1 | 100 | — | 25.00 | 19.51–31.43 | 0.00 | Single-study Wilson |
| Sample size ≥50 | *UGT1A1* | rs1042640C>G | rs1042640 | C | G | 25.00 | 19.51–31.43 | 1 | 100 | — | 25.00 | 19.51–31.43 | 0.00 | Single-study Wilson |
| Single-rsID representations only | *UGT1A1* | rs1042640C>G | rs1042640 | C | G | 25.00 | 19.51–31.43 | 1 | 100 | — | 25.00 | 19.51–31.43 | 0.00 | Single-study Wilson |
| HWE passed or not informative | *UGT1A1* | rs1042640C>G | rs1042640 | C | G | 25.00 | 19.51–31.43 | 1 | 100 | — | 25.00 | 19.51–31.43 | 0.00 | Single-study Wilson |
| Kinh-only samples | *UGT1A1* | rs1042640C>G | rs1042640 | C | G | 25.00 | 19.51–31.43 | 1 | 100 | — | 25.00 | 19.51–31.43 | 0.00 | Single-study Wilson |
| Healthy/control samples only | *UGT1A1* | rs1042640C>G | rs1042640 | C | G | 25.00 | 19.51–31.43 | 1 | 100 | — | 25.00 | 19.51–31.43 | 0.00 | Single-study Wilson |
| Strict sensitivity set | *UGT1A1* | rs10929303C>T | rs10929303 | C | T | 25.00 | 19.51–31.43 | 1 | 100 | — | 25.00 | 19.51–31.43 | 0.00 | Single-study Wilson |
| Sample size ≥50 | *UGT1A1* | rs10929303C>T | rs10929303 | C | T | 25.00 | 19.51–31.43 | 1 | 100 | — | 25.00 | 19.51–31.43 | 0.00 | Single-study Wilson |
| Single-rsID representations only | *UGT1A1* | rs10929303C>T | rs10929303 | C | T | 25.00 | 19.51–31.43 | 1 | 100 | — | 25.00 | 19.51–31.43 | 0.00 | Single-study Wilson |
| HWE passed or not informative | *UGT1A1* | rs10929303C>T | rs10929303 | C | T | 25.00 | 19.51–31.43 | 1 | 100 | — | 25.00 | 19.51–31.43 | 0.00 | Single-study Wilson |
| Kinh-only samples | *UGT1A1* | rs10929303C>T | rs10929303 | C | T | 25.00 | 19.51–31.43 | 1 | 100 | — | 25.00 | 19.51–31.43 | 0.00 | Single-study Wilson |
| Healthy/control samples only | *UGT1A1* | rs10929303C>T | rs10929303 | C | T | 25.00 | 19.51–31.43 | 1 | 100 | — | 25.00 | 19.51–31.43 | 0.00 | Single-study Wilson |
| Strict sensitivity set | *UGT1A1* | rs8330C>G | rs8330 | C | G | 25.00 | 19.51–31.43 | 1 | 100 | — | 25.00 | 19.51–31.43 | 0.00 | Single-study Wilson |
| Sample size ≥50 | *UGT1A1* | rs8330C>G | rs8330 | C | G | 25.00 | 19.51–31.43 | 1 | 100 | — | 25.00 | 19.51–31.43 | 0.00 | Single-study Wilson |
| Single-rsID representations only | *UGT1A1* | rs8330C>G | rs8330 | C | G | 25.00 | 19.51–31.43 | 1 | 100 | — | 25.00 | 19.51–31.43 | 0.00 | Single-study Wilson |
| HWE passed or not informative | *UGT1A1* | rs8330C>G | rs8330 | C | G | 25.00 | 19.51–31.43 | 1 | 100 | — | 25.00 | 19.51–31.43 | 0.00 | Single-study Wilson |
| Kinh-only samples | *UGT1A1* | rs8330C>G | rs8330 | C | G | 25.00 | 19.51–31.43 | 1 | 100 | — | 25.00 | 19.51–31.43 | 0.00 | Single-study Wilson |
| Healthy/control samples only | *UGT1A1* | rs8330C>G | rs8330 | C | G | 25.00 | 19.51–31.43 | 1 | 100 | — | 25.00 | 19.51–31.43 | 0.00 | Single-study Wilson |
| Strict sensitivity set | *VKORC1* | -1173C>T | rs9934438 | C | T | 85.33 | 80.88–88.89 | 1 | 150 | — | 85.33 | 80.88–88.89 | 0.00 | Single-study Wilson |
| Sample size ≥50 | *VKORC1* | -1173C>T | rs9934438 | C | T | 85.33 | 80.88–88.89 | 1 | 150 | — | 85.33 | 80.88–88.89 | 0.00 | Single-study Wilson |
| Single-rsID representations only | *VKORC1* | -1173C>T | rs9934438 | C | T | 85.33 | 80.88–88.89 | 1 | 150 | — | 85.33 | 80.88–88.89 | 0.00 | Single-study Wilson |
| Strict sensitivity set | *VKORC1* | -1639G>A | rs9923231 | G | A | 89.91 | 81.13–94.86 | 2 | 199 | 76.14 | 89.91 | 81.13–94.86 | 0.00 | Random-effects logit (DL) |
| Single-rsID representations only | *VKORC1* | -1639G>A | rs9923231 | G | A | 89.91 | 81.13–94.86 | 2 | 199 | 76.14 | 89.91 | 81.13–94.86 | 0.00 | Random-effects logit (DL) |
| Strict sensitivity set | *VKORC1* | 1173C>T | rs9934438 | C | T | 85.71 | 77.44–91.30 | 1 | 49 | — | 85.71 | 77.44–91.30 | 0.00 | Single-study Wilson |
| Single-rsID representations only | *VKORC1* | 1173C>T | rs9934438 | C | T | 85.71 | 77.44–91.30 | 1 | 49 | — | 85.71 | 77.44–91.30 | 0.00 | Single-study Wilson |
| HWE passed or not informative | *VKORC1* | 1173C>T | rs9934438 | C | T | 85.71 | 77.44–91.30 | 1 | 49 | — | 85.71 | 77.44–91.30 | 0.00 | Single-study Wilson |
| Strict sensitivity set | *VKORC1* | 1542G>C | rs8050894 | G | C | 85.71 | 77.44–91.30 | 1 | 49 | — | 85.71 | 77.44–91.30 | 0.00 | Single-study Wilson |
| Single-rsID representations only | *VKORC1* | 1542G>C | rs8050894 | G | C | 85.71 | 77.44–91.30 | 1 | 49 | — | 85.71 | 77.44–91.30 | 0.00 | Single-study Wilson |
| HWE passed or not informative | *VKORC1* | 1542G>C | rs8050894 | G | C | 85.71 | 77.44–91.30 | 1 | 49 | — | 85.71 | 77.44–91.30 | 0.00 | Single-study Wilson |
| Strict sensitivity set | *VKORC1* | 2255C>T | rs2359612 | C | T | 85.71 | 77.44–91.30 | 1 | 49 | — | 85.71 | 77.44–91.30 | 0.00 | Single-study Wilson |
| Single-rsID representations only | *VKORC1* | 2255C>T | rs2359612 | C | T | 85.71 | 77.44–91.30 | 1 | 49 | — | 85.71 | 77.44–91.30 | 0.00 | Single-study Wilson |
| HWE passed or not informative | *VKORC1* | 2255C>T | rs2359612 | C | T | 85.71 | 77.44–91.30 | 1 | 49 | — | 85.71 | 77.44–91.30 | 0.00 | Single-study Wilson |
| Strict sensitivity set | *VKORC1* | 3730G>A | rs7294 | G | A | 14.29 | 8.70–22.56 | 1 | 49 | — | 14.29 | 8.70–22.56 | 0.00 | Single-study Wilson |
| Single-rsID representations only | *VKORC1* | 3730G>A | rs7294 | G | A | 14.29 | 8.70–22.56 | 1 | 49 | — | 14.29 | 8.70–22.56 | 0.00 | Single-study Wilson |
| HWE passed or not informative | *VKORC1* | 3730G>A | rs7294 | G | A | 14.29 | 8.70–22.56 | 1 | 49 | — | 14.29 | 8.70–22.56 | 0.00 | Single-study Wilson |
| Strict sensitivity set | *VKORC1* | 497T>G | rs2884737 | T | G | 1.02 | 0.18–5.56 | 1 | 49 | — | 1.02 | 0.18–5.56 | 0.00 | Single-study Wilson |
| Single-rsID representations only | *VKORC1* | 497T>G | rs2884737 | T | G | 1.02 | 0.18–5.56 | 1 | 49 | — | 1.02 | 0.18–5.56 | 0.00 | Single-study Wilson |
| HWE passed or not informative | *VKORC1* | 497T>G | rs2884737 | T | G | 1.02 | 0.18–5.56 | 1 | 49 | — | 1.02 | 0.18–5.56 | 0.00 | Single-study Wilson |

Abbreviations: CI, confidence interval; DL, DerSimonian–Laird; HWE, Hardy–Weinberg equilibrium; I², heterogeneity; k, contributing study rows; N, participant sum; pp, percentage points; rsID, Reference SNP cluster identifier.
