# Supplementary material for: Vietnamese pharmacogenomic variation in global context: a systematic review and meta-analysis for clinical implementation
Source: Front Pharmacol. 2026 Sep 3;17:1921144. doi: 10.3389/fphar.2026.1921144 (PMC13582518; doi:10.3389/fphar.2026.1921144)
Supplement: Supplementary file 5 [file Table5.docx]

**Supplementary Table S5. Estimates meeting the predefined instability criteria in sensitivity analyses**

Rows are limited to sensitivity estimates with an absolute difference of at least 5 percentage points from the corrected primary source-level estimate. The criterion identifies estimates that warrant contextual interpretation; it does not establish statistical inconsistency or clinical significance.

| **Scenario** | **Gene** | **Allele/variant representation** | **rsID** | **Reference allele/state** | **Effect allele/state** | **Scenario estimate, %** | **95% CI** | **k** | **Total N** | **I², %** | **Primary estimate, %** | **Primary 95% CI** | **Absolute difference, pp** | **Analysis model** | **Interpretation** |
| --- | --- | --- | --- | --- | --- | --- | --- | --- | --- | --- | --- | --- | --- | --- | --- |
| HWE passed or not informative | *CYP3A5* | *3 | rs776746 | A | G | 72.21 | 65.12–78.34 | 5 | 441 | 79.32 | 60.81 | 44.96–74.67 | 11.40 | Random-effects logit (DL) | Estimate met the prespecified instability threshold (absolute shift ≥5 percentage points). |
| Kinh-only samples | *NAT2* | *6 | rs1799930 | G | A | 36.00 | 29.67–42.86 | 1 | 100 | — | 27.00 | 18.69–37.31 | 9.00 | Single-study Wilson | Estimate met the prespecified instability threshold (absolute shift ≥5 percentage points). |
| Healthy/control samples only | *NAT2* | *6 | rs1799930 | G | A | 36.00 | 29.67–42.86 | 1 | 100 | — | 27.00 | 18.69–37.31 | 9.00 | Single-study Wilson | Estimate met the prespecified instability threshold (absolute shift ≥5 percentage points). |
| Kinh-only samples | *CYP3A5* | *3 | rs776746 | A | G | 66.67 | 58.62–73.84 | 1 | 72 | — | 60.81 | 44.96–74.67 | 5.86 | Single-study Wilson | Estimate met the prespecified instability threshold (absolute shift ≥5 percentage points). |
| HWE passed or not informative | *NAT2* | *6 | rs1799930 | G | A | 32.88 | 29.53–36.40 | 3 | 357 | 0.00 | 27.00 | 18.69–37.31 | 5.88 | Random-effects logit (DL) | Estimate met the prespecified instability threshold (absolute shift ≥5 percentage points). |
| Sample size ≥50 | *CYP3A5* | *3 | rs776746 | A | G | 65.89 | 51.22–78.04 | 6 | 505 | 94.77 | 60.81 | 44.96–74.67 | 5.08 | Random-effects logit (DL) | Estimate met the prespecified instability threshold (absolute shift ≥5 percentage points). |

Abbreviations: CI, confidence interval; HWE, Hardy–Weinberg equilibrium; I², heterogeneity; k, contributing study rows; N, participant sum; pp, percentage points.
