# Supplementary material for: Vietnamese pharmacogenomic variation in global context: a systematic review and meta-analysis for clinical implementation
Source: Front Pharmacol. 2026 Sep 3;17:1921144. doi: 10.3389/fphar.2026.1921144 (PMC13582518; doi:10.3389/fphar.2026.1921144)
Supplement: Supplementary file 6 [file Table6.docx]

**Supplementary Table S6. Subgroup comparisons by Hardy–Weinberg status, ethnicity, health context, sample size, and genotyping method**

Subgroup comparisons were performed only where both levels were represented by analysable rows. Odds ratios compare the effect allele in level 1 with level 2. Benjamini–Hochberg adjustment was applied across the subgroup tests retained in this table; estimates should be interpreted cautiously because many subgroups contain few rows and are observational rather than randomized contrasts.

| **Subgroup variable** | **Gene** | **Allele/variant representation** | **rsID** | **Level 1** | **Level 2** | **Study rows, level 1** | **Study rows, level 2** | **Participants, level 1** | **Participants, level 2** | **Allele frequency, level 1, %** | **Allele frequency, level 2, %** | **Difference (level 1 – level 2), pp** | **OR (95% CI)** | **Raw p value** | **FDR-adjusted p value** | **FDR <0.05** |
| --- | --- | --- | --- | --- | --- | --- | --- | --- | --- | --- | --- | --- | --- | --- | --- | --- |
| Hardy–Weinberg status | *CYP3A5* | *3 | rs776746 | HWE passed or not informative | HWE warning | 5 | 2 | 441 | 102 | 72.34 | 28.43 | 43.90 | 6.58 (4.69–9.23) | 8.89e-28 | 4.18e-26 | Yes |
| Hardy–Weinberg status | *NAT2* | *6 | rs1799930 | HWE passed or not informative | HWE warning | 3 | 1 | 357 | 100 | 32.77 | 12.50 | 20.27 | 3.41 (2.18–5.34) | 7.49e-08 | 7.04e-07 | Yes |
| Hardy–Weinberg status | *CYP2C19* | *3 | rs4986893 | HWE passed or not informative | HWE warning | 18 | 2 | 2,039 | 740 | 4.95 | 8.04 | -3.09 | 0.60 (0.47–0.75) | 1.56e-05 | 0.000122 | Yes |
| Hardy–Weinberg status | *VKORC1* | -1639G>A | rs9923231 | HWE warning | HWE passed or not informative | 1 | 1 | 150 | 49 | 92.67 | 85.71 | 6.95 | 2.11 (1.03–4.30) | 0.0407 | 0.112 | No |
| Hardy–Weinberg status | *CYP2C19* | *2 | rs4244285 | HWE passed or not informative | HWE failed | 17 | 1 | 1,900 | 650 | 27.50 | 28.69 | -1.19 | 0.94 (0.82–1.08) | 0.408 | 0.676 | No |
| Hardy–Weinberg status | *UGT1A1* | *28 | rs8175347 | HWE passed or not informative | HWE warning | 1 | 1 | 95 | 50 | 8.42 | 6.00 | 2.42 | 1.44 (0.55–3.80) | 0.461 | 0.676 | No |
| Ethnicity reporting | *NAT2* | *6 | rs1799930 | Vietnamese, ethnicity unspecified | Kinh | 3 | 1 | 357 | 100 | 26.19 | 36.00 | -9.81 | 0.63 (0.45–0.88) | 0.00677 | 0.0318 | Yes |
| Ethnicity reporting | *UGT1A1* | *6 | rs4148323 | Kinh | Vietnamese, ethnicity unspecified | 1 | 1 | 100 | 50 | 13.00 | 5.00 | 8.00 | 2.84 (1.06–7.63) | 0.0387 | 0.112 | No |
| Ethnicity reporting | *CYP2C19* | *3 | rs4986893 | Vietnamese, ethnicity unspecified | Kinh | 16 | 4 | 2,342 | 437 | 6.04 | 4.35 | 1.69 | 1.41 (1.00–2.00) | 0.0498 | 0.127 | No |
| Ethnicity reporting | *CYP2C19* | *2 | rs4244285 | Vietnamese, ethnicity unspecified | Kinh | 16 | 3 | 2,342 | 272 | 28.18 | 24.82 | 3.36 | 1.19 (0.97–1.46) | 0.0977 | 0.23 | No |
| Ethnicity reporting | *NAT2* | *5 | rs1801280 | Vietnamese, ethnicity unspecified | Kinh | 3 | 1 | 357 | 100 | 5.32 | 8.00 | -2.68 | 0.65 (0.35–1.19) | 0.159 | 0.324 | No |
| Ethnicity reporting | *NAT2* | *7 | rs1799931 | Vietnamese, ethnicity unspecified | Kinh | 3 | 1 | 357 | 100 | 18.63 | 14.50 | 4.13 | 1.35 (0.87–2.09) | 0.178 | 0.348 | No |
| Ethnicity reporting | *CYP2C9* | *3 | rs1057910 | Kinh | Vietnamese, ethnicity unspecified | 3 | 3 | 357 | 381 | 2.66 | 3.28 | -0.62 | 0.81 (0.44–1.48) | 0.485 | 0.676 | No |
| Ethnicity reporting | *CYP2C9* | *2 | rs1799853 | Kinh | Vietnamese, ethnicity unspecified | 2 | 1 | 257 | 92 | 0.00 | 0.00 | 0.00 | 0.36 (0.01–18.14) | 0.608 | 0.715 | No |
| Ethnicity reporting | *UGT1A1* | *28 | rs8175347 | Kinh | Vietnamese, ethnicity unspecified | 1 | 1 | 95 | 50 | 8.42 | 6.00 | 2.42 | 1.44 (0.55–3.80) | 0.461 | 0.676 | No |
| Ethnicity reporting | *CYP3A5* | *3 | rs776746 | Vietnamese, ethnicity unspecified | Kinh | 6 | 1 | 471 | 72 | 63.69 | 66.67 | -2.97 | 0.88 (0.61–1.27) | 0.489 | 0.676 | No |
| Ethnicity reporting | *CYP2D6* | *5 | — | Kinh | Vietnamese, ethnicity unspecified | 1 | 1 | 136 | 126 | 8.09 | 6.75 | 1.34 | 1.22 (0.63–2.35) | 0.559 | 0.709 | No |
| Ethnicity reporting | *CYP2C19* | *17 | rs12248560 | Vietnamese, ethnicity unspecified | Kinh | 2 | 1 | 322 | 100 | 1.40 | 1.00 | 0.40 | 1.40 (0.30–6.55) | 0.666 | 0.758 | No |
| Ethnicity reporting | *CYP2C9* | *13 | rs72558187 | Vietnamese, ethnicity unspecified | Kinh | 1 | 1 | 127 | 100 | 0.79 | 0.50 | 0.29 | 1.58 (0.14–17.54) | 0.71 | 0.758 | No |
| Genotyping-method category | *NAT2* | *6 | rs1799930 | Sequencing-based | PCR-RFLP | 3 | 1 | 357 | 100 | 32.77 | 12.50 | 20.27 | 3.41 (2.18–5.34) | 7.49e-08 | 7.04e-07 | Yes |
| Genotyping-method category | *CYP3A5* | *3 | rs776746 | Sequencing-based | Other/unclear method | 4 | 1 | 280 | 127 | 65.00 | 78.35 | -13.35 | 0.51 (0.36–0.73) | 0.000154 | 0.00103 | Yes |
| Genotyping-method category | *NAT2* | *7 | rs1799931 | Sequencing-based | PCR-RFLP | 3 | 1 | 357 | 100 | 15.55 | 25.50 | -9.95 | 0.54 (0.37–0.78) | 0.00126 | 0.0074 | Yes |
| Genotyping-method category | *NAT2* | *5 | rs1801280 | Sequencing-based | PCR-RFLP | 3 | 1 | 357 | 100 | 7.00 | 2.00 | 5.00 | 3.69 (1.32–10.34) | 0.0131 | 0.0558 | No |
| Genotyping-method category | *VKORC1* | -1639G>A | rs9923231 | Sequencing-based | MALDI-TOF/MassARRAY | 1 | 1 | 150 | 49 | 92.67 | 85.71 | 6.95 | 2.11 (1.03–4.30) | 0.0407 | 0.112 | No |
| Genotyping-method category | *CYP2C19* | *3 | rs4986893 | Sequencing-based | Real-time PCR/TaqMan | 9 | 6 | 1,074 | 1,104 | 5.21 | 6.25 | -1.04 | 0.83 (0.64–1.07) | 0.142 | 0.304 | No |
| Genotyping-method category | *CYP2C19* | *17 | rs12248560 | Sequencing-based | Other/unclear method | 2 | 1 | 295 | 127 | 1.02 | 1.97 | -0.95 | 0.51 (0.15–1.69) | 0.272 | 0.512 | No |
| Genotyping-method category | *CYP2C9* | *2 | rs1799853 | Sequencing-based | Real-time PCR/TaqMan | 2 | 1 | 257 | 92 | 0.00 | 0.00 | 0.00 | 0.36 (0.01–18.14) | 0.608 | 0.715 | No |
| Genotyping-method category | *CYP2D6* | *5 | — | Sequencing-based | Other/unclear method | 1 | 1 | 136 | 126 | 8.09 | 6.75 | 1.34 | 1.22 (0.63–2.35) | 0.559 | 0.709 | No |
| Genotyping-method category | *CYP2C9* | *13 | rs72558187 | Other/unclear method | Sequencing-based | 1 | 1 | 127 | 100 | 0.79 | 0.50 | 0.29 | 1.58 (0.14–17.54) | 0.71 | 0.758 | No |
| Genotyping-method category | *CYP2C9* | *3 | rs1057910 | Sequencing-based | Real-time PCR/TaqMan | 5 | 1 | 646 | 92 | 2.94 | 3.26 | -0.32 | 0.90 (0.37–2.16) | 0.812 | 0.848 | No |
| Genotyping-method category | *CYP2C19* | *2 | rs4244285 | Real-time PCR/TaqMan | Sequencing-based | 6 | 8 | 1,104 | 909 | 27.63 | 27.50 | 0.12 | 1.01 (0.88–1.16) | 0.93 | 0.93 | No |
| Sampling/health context | *CYP3A5* | *3 | rs776746 | Patient/clinical sample | Unclear context | 4 | 3 | 276 | 267 | 52.36 | 76.22 | -23.86 | 0.34 (0.26–0.44) | 7.05e-16 | 1.66e-14 | Yes |
| Sampling/health context | *NAT2* | *6 | rs1799930 | Patient/clinical sample | Healthy/control | 2 | 2 | 257 | 200 | 31.52 | 24.25 | 7.27 | 1.44 (1.07–1.93) | 0.0158 | 0.0619 | No |
| Sampling/health context | *UGT1A1* | *6 | rs4148323 | Healthy/control | Patient/clinical sample | 1 | 1 | 100 | 50 | 13.00 | 5.00 | 8.00 | 2.84 (1.06–7.63) | 0.0387 | 0.112 | No |
| Sampling/health context | *CYP2C19* | *3 | rs4986893 | Patient/clinical sample | Healthy/control | 14 | 3 | 2,125 | 365 | 5.76 | 3.97 | 1.79 | 1.48 (1.00–2.19) | 0.0512 | 0.127 | No |
| Sampling/health context | *NAT2* | *7 | rs1799931 | Patient/clinical sample | Healthy/control | 2 | 2 | 257 | 200 | 15.95 | 20.00 | -4.05 | 0.76 (0.54–1.07) | 0.113 | 0.252 | No |
| Sampling/health context | *CYP2C19* | *17 | rs12248560 | Patient/clinical sample | Unclear context | 1 | 1 | 195 | 127 | 1.03 | 1.97 | -0.94 | 0.52 (0.14–1.94) | 0.328 | 0.57 | No |
| Sampling/health context | *CYP2C9* | *3 | rs1057910 | Patient/clinical sample | Healthy/control | 3 | 1 | 381 | 100 | 3.28 | 2.50 | 0.78 | 1.32 (0.50–3.50) | 0.573 | 0.709 | No |
| Sampling/health context | *NAT2* | *5 | rs1801280 | Patient/clinical sample | Healthy/control | 2 | 2 | 257 | 200 | 6.61 | 5.00 | 1.61 | 1.35 (0.76–2.38) | 0.306 | 0.553 | No |
| Sampling/health context | *UGT1A1* | *28 | rs8175347 | Healthy/control | Patient/clinical sample | 1 | 1 | 95 | 50 | 8.42 | 6.00 | 2.42 | 1.44 (0.55–3.80) | 0.461 | 0.676 | No |
| Sampling/health context | *CYP2C19* | *2 | rs4244285 | Patient/clinical sample | Unclear context | 14 | 3 | 2,125 | 289 | 28.40 | 27.16 | 1.24 | 1.06 (0.88–1.29) | 0.535 | 0.709 | No |
| Sampling/health context | *CYP2C9* | *13 | rs72558187 | Unclear context | Healthy/control | 1 | 1 | 127 | 100 | 0.79 | 0.50 | 0.29 | 1.58 (0.14–17.54) | 0.71 | 0.758 | No |
| Sampling/health context | *CYP2C9* | *2 | rs1799853 | Patient/clinical sample | Healthy/control | 2 | 0 | 249 | 0 | 0.00 | — | — | 0.00 (0.00–0.12) | 0.00483 | 0.0252 | Yes |
| Study-row sample size | *CYP3A5* | *3 | rs776746 | n ≥50 | n 30–49 | 6 | 1 | 505 | 38 | 66.83 | 27.63 | 39.20 | 5.28 (3.14–8.87) | 3.5e-10 | 5.48e-09 | Yes |
| Study-row sample size | *VKORC1* | -1639G>A | rs9923231 | n ≥50 | n 30–49 | 1 | 1 | 150 | 49 | 92.67 | 85.71 | 6.95 | 2.11 (1.03–4.30) | 0.0407 | 0.112 | No |
| Study-row sample size | *CYP2C19* | *3 | rs4986893 | n ≥50 | n 30–49 | 19 | 1 | 2,749 | 30 | 5.80 | 3.33 | 2.47 | 1.79 (0.43–7.35) | 0.421 | 0.676 | No |
| Study-row sample size | *CYP2C19* | *2 | rs4244285 | n ≥50 | n 30–49 | 18 | 1 | 2,584 | 30 | 27.84 | 26.67 | 1.18 | 1.06 (0.60–1.89) | 0.84 | 0.858 | No |

Abbreviations: AF, allele frequency; CI, confidence interval; FDR, false discovery rate; HWE, Hardy–Weinberg equilibrium; OR, odds ratio; pp, percentage points.
