# Supplementary material for: Vietnamese pharmacogenomic variation in global context: a systematic review and meta-analysis for clinical implementation
Source: Front Pharmacol. 2026 Sep 3;17:1921144. doi: 10.3389/fphar.2026.1921144 (PMC13582518; doi:10.3389/fphar.2026.1921144)
Supplement: Supplementary file 7 [file Table7.docx]

**Supplementary Table S7. Full functional and clinical annotation of Vietnamese pharmacogenetic variant representations**

Clinical priority integrates gene–drug guidance, functional consequence, preventability or severity of the associated outcome, estimated Vietnamese frequency, and assay complexity. It is a representation-level annotation and must not be interpreted as a count of independent loci or as a direct prescribing recommendation for an unphenotyped individual.

CYP2D6 star alleles are kept distinct from single-marker proxies. CYP2D6*10 is reported at 43.75% (95% CI 37.98–49.69) and CYP2D6*4 at 0.74% (0.20–2.64); rs1065852 and rs3892097 remain source-level markers only. Phenotype assignment requires resolved diplotypes and, where relevant, copy-number and structural-variant assessment.

| **Gene** | **Allele/variant representation** | **rsID** | **Variant class** | **Functional interpretation** | **Expected phenotype implication** | **Relevant drug(s)** | **CPIC guidance** | **DPWG guidance** | **Source-level Vietnamese estimate** | **Clinical priority** | **Use in main manuscript** | **Annotation confidence** | **Representation/interpretation caveat** |
| --- | --- | --- | --- | --- | --- | --- | --- | --- | --- | --- | --- | --- | --- |
| *CYP2B6* | 516G>T | rs3745274 | Single-rsID variant | Decreased function | Reduced CYP2B6 activity; contributes to slower efavirenz metabolism and higher efavirenz exposure depending on diplotype. | Efavirenz; nevirapine; bupropion; methadone | Guideline available | No direct/unclear guideline | 27.08% (20.49–34.87) | High | Contributes to harmonized Table 4/Figure 5 estimate: 24.76% (95% CI 20.48–29.61), k=2, N=172. | High | Synonymous source labels sharing the same rsID and effect-allele orientation were collapsed only for the main-text harmonized estimate. |
| *CYP2B6* | rs3745274 | rs3745274 | Single-rsID variant | Decreased function | Reduced CYP2B6 activity; contributes to slower efavirenz metabolism and higher efavirenz exposure depending on diplotype. | Efavirenz; nevirapine; bupropion; methadone | Guideline available | No direct/unclear guideline | 23.00% (17.71–29.31) | High | Contributes to harmonized Table 4/Figure 5 estimate: 24.76% (95% CI 20.48–29.61), k=2, N=172. | High | Synonymous source labels sharing the same rsID and effect-allele orientation were collapsed only for the main-text harmonized estimate. |
| *CYP2C19* | *2 | rs4244285 | Single-rsID variant | No function | Loss-of-function allele; contributes to intermediate or poor metabolizer phenotype depending on diplotype | Clopidogrel; proton pump inhibitors; selected antidepressants | Guideline available | Guideline available | 27.90% (26.57–29.27) | High | Table 4/Figure 5 primary representative: 27.90% (95% CI 26.57–29.27). | High | A single variant may be insufficient for phenotype assignment; interpret in the relevant haplotype, diplotype, structural, and drug-specific context. |
| *CYP2C19* | *3 | rs4986893 | Single-rsID variant | No function | Loss-of-function allele; contributes to poor metabolizer phenotype when combined with another no-function allele | Clopidogrel; proton pump inhibitors; selected antidepressants | Guideline available | Guideline available | 5.74% (4.82–6.82) | High | Table 4/Figure 5 primary representative: 5.74% (95% CI 4.82–6.82). | High | A single variant may be insufficient for phenotype assignment; interpret in the relevant haplotype, diplotype, structural, and drug-specific context. |
| *CYP2D6* | *10 | — | Structural/CNV or complex star-allele representation | Decreased function | Reduced-function allele; common in East Asian populations and contributes to intermediate metabolizer phenotype | Codeine; tramadol; antidepressants; antipsychotics; tamoxifen | Guideline available | Guideline available | 43.75% (37.98–49.69) | High | Primary CYP2D6*10 star-allele estimate in Table 4/Figure 5: 43.75% (95% CI 37.98–49.69). | High | Interpret within a resolved diplotype and activity-score framework; assay coverage for copy number, hybrids, and tandem arrangements must be stated. |
| *CYP2D6* | rs1065852 C>T marker | rs1065852 | Single-rsID variant | Single-marker proxy; not sufficient to define CYP2D6*10 | May occur within reduced-function haplotypes, but genotype-to-phenotype translation requires a resolved CYP2D6 diplotype and structural assessment. | Codeine; tramadol; antidepressants; antipsychotics; tamoxifen | Guideline available | Guideline available | 45.07% (37.12–53.28) | High | Not displayed as CYP2D6*10; single-marker proxy only. Table 4/Figure 5 use the source-supported star-allele estimate. | High | rs1065852 alone does not define CYP2D6*10. Full star-allele, phase, copy-number, and structural context is required. |
| *CYP2D6* | *10B | — | Haplotype/star-allele representation | Decreased function or reduced-function structural allele | May contribute to intermediate metabolizer phenotype depending on diplotype and structural configuration. | Codeine; tramadol; antidepressants; antipsychotics; tamoxifen; atomoxetine; ondansetron | Guideline available | Guideline available | 55.95% (49.78–61.95) | High | Supplementary annotation; not displayed as a separate main-text representative. | Moderate | Interpret within a resolved diplotype and activity-score framework; assay coverage for copy number, hybrids, and tandem arrangements must be stated. |
| *CYP2D6* | *36-*10 | — | Structural/CNV or complex star-allele representation | Decreased function or reduced-function structural allele | May contribute to intermediate metabolizer phenotype depending on diplotype and structural configuration. | Codeine; tramadol; antidepressants; antipsychotics; tamoxifen; atomoxetine; ondansetron | Guideline available | Guideline available | 11.40% (8.15–15.72) | High | Supplementary annotation; not displayed as a separate main-text representative. | Moderate | Interpret within a resolved diplotype and activity-score framework; assay coverage for copy number, hybrids, and tandem arrangements must be stated. |
| *CYP2D6* | *5 | — | Structural/CNV or complex star-allele representation | No function | Gene deletion; contributes to poor metabolizer phenotype depending on diplotype | Codeine; tramadol; antidepressants; antipsychotics; tamoxifen | Guideline available | Guideline available | 7.75% (4.38–13.34) | High | Source-level deletion representation; retained for traceability and not displayed independently. | High | Interpret within a resolved diplotype and activity-score framework; assay coverage for copy number, hybrids, and tandem arrangements must be stated. |
| *CYP2D6* | *5 | — | Structural/CNV or complex star-allele representation | No function | Gene deletion; contributes to poor metabolizer phenotype depending on diplotype | Codeine; tramadol; antidepressants; antipsychotics; tamoxifen | Guideline available | Guideline available | 7.47% (5.51–10.06) | High | Primary pooled whole-gene-deletion estimate in Table 4/Figure 5: 7.47% (95% CI 5.51–10.06). | High | Interpret within a resolved diplotype and activity-score framework; assay coverage for copy number, hybrids, and tandem arrangements must be stated. |
| *CYP3A5* | *3 | rs776746 | Single-rsID variant | No function | Non-expresser allele; associated with lower CYP3A5 expression | Tacrolimus | Guideline available | Guideline available | 60.81% (44.96–74.67) | High | Table 4/Figure 5 primary representative: 60.81% (95% CI 44.96–74.67). | High | A single variant may be insufficient for phenotype assignment; interpret in the relevant haplotype, diplotype, structural, and drug-specific context. |
| *CYP4F2* | V433M | rs2108622 | Single-rsID variant | Decreased vitamin K oxidation / warfarin dose modifier | Associated with altered warfarin dose requirements; effect is modest compared with VKORC1 and CYP2C9. | Warfarin | Guideline available | No direct/unclear guideline | 16.50% (12.00–22.27) | High | Table 4/Figure 5 primary representative (V433M): 16.50% (95% CI 12.00–22.27). | Moderate | A single variant may be insufficient for phenotype assignment; interpret in the relevant haplotype, diplotype, structural, and drug-specific context. |
| *HLA-B* | *58:01 | — | Non-rsID representation | Risk allele | Strongly associated with allopurinol-induced severe cutaneous adverse reactions | Allopurinol | Guideline available | Guideline available | 6.02% (3.74–9.55) | High | Table 4/Figure 5 primary representative: 6.02% (95% CI 3.74–9.55). | High | A single variant may be insufficient for phenotype assignment; interpret in the relevant haplotype, diplotype, structural, and drug-specific context. |
| *SLCO1B1* | *15 | rs4149056 | Single-rsID variant | Decreased function | Reduced transporter function depending on haplotype definition | Simvastatin; atorvastatin; rosuvastatin | Guideline available | Guideline available | 16.35% (11.94–21.97) | High | Source-reported *15 haplotype/star-allele label involving rs4149056; not displayed as an independent c.521T>C estimate. | Moderate | The source label *15 is haplotype-dependent and is not interchangeable with a stand-alone rs4149056 frequency. |
| *SLCO1B1* | c.521T>C | rs4149056 | Single-rsID variant | Decreased function | Reduced hepatic uptake transporter function; increased systemic statin exposure and myopathy risk | Simvastatin; atorvastatin; rosuvastatin | Guideline available | Guideline available | 12.73% (7.36–21.13) | High | Primary c.521T>C estimate in Table 4/Figure 5: 12.73% (95% CI 7.36–21.13). | High | The c.521T>C estimate excludes source-reported *5/*15 rows to avoid double-counting non-independent representations. |
| *UGT1A1* | *28 | rs8175347 | Single-rsID variant | Decreased function | Reduced UGT1A1 expression; increased risk of irinotecan toxicity and hyperbilirubinemia-related drug effects | Irinotecan; atazanavir | Guideline available | Guideline available | 8.42% (5.25–13.24) | High | Primary source representation in Table 4/Figure 5: 8.42% (95% CI 5.25–13.24). | High | TA-repeat nomenclature and assay definition should be reported explicitly; source-level representations are retained separately. |
| *UGT1A1* | *28 | rs8175347 | Single-rsID variant | Decreased function | Reduced UGT1A1 expression; increased risk of irinotecan toxicity and hyperbilirubinemia-related drug effects | Irinotecan; atazanavir | Guideline available | Guideline available | 6.00% (2.78–12.48) | High | Additional source-level TA-repeat representation; retained for traceability and not displayed independently. | High | TA-repeat nomenclature and assay definition should be reported explicitly; source-level representations are retained separately. |
| *UGT1A1* | *6 | rs4148323 | Single-rsID variant | Decreased function | Reduced glucuronidation activity; increased risk of irinotecan toxicity in susceptible genotypes | Irinotecan; atazanavir | Guideline available | Guideline available | 8.76% (3.38–20.88) | High | Table 4/Figure 5 primary representative: 8.76% (95% CI 3.38–20.88). | High | A single variant may be insufficient for phenotype assignment; interpret in the relevant haplotype, diplotype, structural, and drug-specific context. |
| *VKORC1* | -1173C>T | rs9934438 | Single-rsID variant | Reduced VKORC1 expression / warfarin sensitivity tag | Strong linkage/tagging marker for warfarin sensitivity haplotype; associated with lower warfarin dose requirement. | Warfarin | Guideline available | Guideline available | 85.33% (80.88–88.89) | High | Contributes to harmonized Table 2/Table 4 estimate: 85.43% (95% CI 81.61–88.56), k=2, N=199. | High | Synonymous source labels sharing the same rsID and effect-allele orientation were collapsed only for the main-text harmonized estimate. |
| *VKORC1* | -1639G>A | rs9923231 | Single-rsID variant | Reduced VKORC1 expression | Associated with increased warfarin sensitivity and lower dose requirement | Warfarin | Guideline available | Guideline available | 89.91% (81.13–94.86) | High | Table 4/Figure 5 primary representative: 89.91% (95% CI 81.13–94.86). | High | A single variant may be insufficient for phenotype assignment; interpret in the relevant haplotype, diplotype, structural, and drug-specific context. |
| *VKORC1* | 1173C>T | rs9934438 | Single-rsID variant | Reduced VKORC1 expression / warfarin sensitivity tag | Strong linkage/tagging marker for warfarin sensitivity haplotype; associated with lower warfarin dose requirement. | Warfarin | Guideline available | Guideline available | 85.71% (77.44–91.30) | High | Contributes to harmonized Table 2/Table 4 estimate: 85.43% (95% CI 81.61–88.56), k=2, N=199. | High | Synonymous source labels sharing the same rsID and effect-allele orientation were collapsed only for the main-text harmonized estimate. |
| *CYP2C19* | *17 | rs11188072 | Single-rsID variant | Increased function | Increased-function allele; contributes to rapid or ultrarapid metabolizer phenotype depending on diplotype | Clopidogrel; proton pump inhibitors; selected antidepressants | Guideline available | Guideline available | 1.04% (0.29–3.72) | Moderate | Supplementary marker only; not treated as equivalent to the defining rs12248560 increased-function allele. | High | The defining CYP2C19*17 promoter variant is rs12248560; this source-labelled marker is retained only for traceability. |
| *CYP2C19* | *17 | rs12248560 | Single-rsID variant | Increased function | Increased-function allele; contributes to rapid or ultrarapid metabolizer phenotype depending on diplotype | Clopidogrel; proton pump inhibitors; selected antidepressants | Guideline available | Guideline available | 1.37% (0.76–2.46) | Moderate | Primary CYP2C19*17 representation in Table 4/Figure 5: 1.37% (95% CI 0.76–2.46). | High | Source-level representation retained for traceability; it should not be interpreted as an independent locus when another synonymous or structurally related representation is present. |
| *CYP2C19* | *5 | rs56337013 | Single-rsID variant | No function | Loss-of-function CYP2C19 allele; may contribute to intermediate or poor metabolizer phenotype depending on diplotype. | Clopidogrel; proton pump inhibitors; antidepressants | Guideline available | Guideline available | 0.50% (0.09–2.78) | Moderate | Supplementary annotation; not displayed as a separate main-text representative. | Moderate | Source-level representation retained for traceability; it should not be interpreted as an independent locus when another synonymous or structurally related representation is present. |
| *CYP2C9* | *2 | rs1799853 | Single-rsID variant | Decreased function | Reduced CYP2C9 activity; contributes to intermediate or poor metabolizer phenotype depending on diplotype | Warfarin; phenytoin; NSAIDs; siponimod | Guideline available | Guideline available | 0.22% (0.04–1.08) | Moderate | Table 4/Figure 5 primary representative: 0.22% (95% CI 0.04–1.08). | High | A single variant may be insufficient for phenotype assignment; interpret in the relevant haplotype, diplotype, structural, and drug-specific context. |
| *CYP2C9* | *3 | rs1057910 | Single-rsID variant | Decreased function | Reduced CYP2C9 activity; clinically relevant for warfarin and phenytoin dosing | Warfarin; phenytoin; NSAIDs; siponimod | Guideline available | Guideline available | 3.27% (2.46–4.32) | Moderate | Table 4/Figure 5 primary representative: 3.27% (95% CI 2.46–4.32). | High | A single variant may be insufficient for phenotype assignment; interpret in the relevant haplotype, diplotype, structural, and drug-specific context. |
| *CYP2D6* | *10BX2 | — | Structural/CNV or complex star-allele representation | Duplicated decreased-function allele | May increase copy number of a reduced-function CYP2D6 allele; net phenotype depends on the full diplotype and copy-number structure. | Codeine; tramadol; antidepressants; antipsychotics; tamoxifen; atomoxetine; ondansetron | Guideline available | Guideline available | 0.40% (0.07–2.21) | Moderate | Supplementary annotation; not displayed as a separate main-text representative. | Moderate | Interpret within a resolved diplotype and activity-score framework; assay coverage for copy number, hybrids, and tandem arrangements must be stated. |
| *CYP2D6* | *13 | — | Structural/CNV or complex star-allele representation | No function or likely no function | May contribute to poor metabolizer phenotype when present with another no-function allele; exact phenotype requires diplotype interpretation. | Codeine; tramadol; antidepressants; antipsychotics; tamoxifen; atomoxetine; ondansetron | Guideline available | Guideline available | 0.74% (0.20–2.64) | Moderate | Supplementary annotation; not displayed as a separate main-text representative. | Moderate | Interpret within a resolved diplotype and activity-score framework; assay coverage for copy number, hybrids, and tandem arrangements must be stated. |
| *CYP2D6* | *14 | — | Structural/CNV or complex star-allele representation | Decreased function or reduced-function structural allele | May contribute to intermediate metabolizer phenotype depending on diplotype and structural configuration. | Codeine; tramadol; antidepressants; antipsychotics; tamoxifen; atomoxetine; ondansetron | Guideline available | Guideline available | 0.37% (0.06–2.05) | Moderate | Supplementary annotation; not displayed as a separate main-text representative. | Moderate | Interpret within a resolved diplotype and activity-score framework; assay coverage for copy number, hybrids, and tandem arrangements must be stated. |
| *CYP2D6* | *14B | — | Haplotype/star-allele representation | Decreased function or reduced-function structural allele | May contribute to intermediate metabolizer phenotype depending on diplotype and structural configuration. | Codeine; tramadol; antidepressants; antipsychotics; tamoxifen; atomoxetine; ondansetron | Guideline available | Guideline available | 0.79% (0.22–2.85) | Moderate | Supplementary annotation; not displayed as a separate main-text representative. | Moderate | Interpret within a resolved diplotype and activity-score framework; assay coverage for copy number, hybrids, and tandem arrangements must be stated. |
| *CYP2D6* | *15 | — | Structural/CNV or complex star-allele representation | No function or likely no function | May contribute to poor metabolizer phenotype when present with another no-function allele; exact phenotype requires diplotype interpretation. | Codeine; tramadol; antidepressants; antipsychotics; tamoxifen; atomoxetine; ondansetron | Guideline available | Guideline available | 0.37% (0.06–2.05) | Moderate | Supplementary annotation; not displayed as a separate main-text representative. | Moderate | Interpret within a resolved diplotype and activity-score framework; assay coverage for copy number, hybrids, and tandem arrangements must be stated. |
| *CYP2D6* | *1XN | — | Structural/CNV or complex star-allele representation | Increased function / copy-number gain | Increased CYP2D6 activity may contribute to ultrarapid metabolizer phenotype depending on duplicated allele activity. | Codeine; tramadol; antidepressants; antipsychotics; tamoxifen; atomoxetine; ondansetron | Guideline available | Guideline available | 0.40% (0.07–2.21) | Moderate | Supplementary annotation; not displayed as a separate main-text representative. | Moderate | Interpret within a resolved diplotype and activity-score framework; assay coverage for copy number, hybrids, and tandem arrangements must be stated. |
| *CYP2D6* | *36 | — | Structural/CNV or complex star-allele representation | No function or nonfunctional hybrid | Hybrid allele; interpretation depends on copy-number and tandem structure | Codeine; tramadol; antidepressants; antipsychotics; tamoxifen | Guideline available | Guideline available | 0.37% (0.06–2.05) | Moderate | Table 4/Figure 5 primary complex-allele representative: 0.37% (95% CI 0.06–2.05). | Moderate | Interpret within a resolved diplotype and activity-score framework; assay coverage for copy number, hybrids, and tandem arrangements must be stated. |
| *CYP2D6* | *36-*36-*10 | — | Structural/CNV or complex star-allele representation | Decreased function or reduced-function structural allele | May contribute to intermediate metabolizer phenotype depending on diplotype and structural configuration. | Codeine; tramadol; antidepressants; antipsychotics; tamoxifen; atomoxetine; ondansetron | Guideline available | Guideline available | 0.37% (0.06–2.05) | Moderate | Supplementary annotation; not displayed as a separate main-text representative. | Moderate | Interpret within a resolved diplotype and activity-score framework; assay coverage for copy number, hybrids, and tandem arrangements must be stated. |
| *CYP2D6* | *4 | — | Structural/CNV or complex star-allele representation | No function | Loss-of-function allele; contributes to poor metabolizer phenotype depending on diplotype | Codeine; tramadol; antidepressants; antipsychotics; tamoxifen | Guideline available | Guideline available | 0.74% (0.20–2.64) | Moderate | Primary CYP2D6*4 star-allele estimate in Table 4/Figure 5: 0.74% (95% CI 0.20–2.64). | High | Interpret within a resolved diplotype and activity-score framework; assay coverage for copy number, hybrids, and tandem arrangements must be stated. |
| *CYP2D6* | rs3892097 G>A marker | rs3892097 | Single-rsID variant | Defining marker within CYP2D6*4; not a stand-alone star-allele call | May indicate a no-function allele in the appropriate haplotype, but phenotype assignment requires star-allele and structural context. | Codeine; tramadol; antidepressants; antipsychotics; tamoxifen | Guideline available | Guideline available | 1.45% (0.40–5.13) | Moderate | Not displayed as CYP2D6*4; single-marker proxy only. Table 4/Figure 5 use the source-supported star-allele estimate. | High | rs3892097 is a defining marker but is not sufficient to resolve all CYP2D6*4 structural contexts; do not infer phenotype from this SNP alone. |
| *CYP2D6* | *41 | — | Haplotype/star-allele representation | Decreased function or reduced-function structural allele | May contribute to intermediate metabolizer phenotype depending on diplotype and structural configuration. | Codeine; tramadol; antidepressants; antipsychotics; tamoxifen; atomoxetine; ondansetron | Guideline available | Guideline available | 4.37% (2.45–7.65) | Moderate | Supplementary annotation; not displayed as a separate main-text representative. | Moderate | Interpret within a resolved diplotype and activity-score framework; assay coverage for copy number, hybrids, and tandem arrangements must be stated. |
| *CYP2D6* | *49 | — | Haplotype/star-allele representation | Decreased function or reduced-function structural allele | May contribute to intermediate metabolizer phenotype depending on diplotype and structural configuration. | Codeine; tramadol; antidepressants; antipsychotics; tamoxifen; atomoxetine; ondansetron | Guideline available | Guideline available | 1.19% (0.41–3.44) | Moderate | Supplementary annotation; not displayed as a separate main-text representative. | Moderate | Interpret within a resolved diplotype and activity-score framework; assay coverage for copy number, hybrids, and tandem arrangements must be stated. |
| *CYP2D6* | *60 | — | Structural/CNV or complex star-allele representation | No function or likely no function | May contribute to poor metabolizer phenotype when present with another no-function allele; exact phenotype requires diplotype interpretation. | Codeine; tramadol; antidepressants; antipsychotics; tamoxifen; atomoxetine; ondansetron | Guideline available | Guideline available | 0.74% (0.20–2.64) | Moderate | Supplementary annotation; not displayed as a separate main-text representative. | Moderate | Interpret within a resolved diplotype and activity-score framework; assay coverage for copy number, hybrids, and tandem arrangements must be stated. |
| *CYP2D6* | *65 | — | Structural/CNV or complex star-allele representation | Decreased function or reduced-function structural allele | May contribute to intermediate metabolizer phenotype depending on diplotype and structural configuration. | Codeine; tramadol; antidepressants; antipsychotics; tamoxifen; atomoxetine; ondansetron | Guideline available | Guideline available | 2.94% (1.50–5.70) | Moderate | Supplementary annotation; not displayed as a separate main-text representative. | Moderate | Interpret within a resolved diplotype and activity-score framework; assay coverage for copy number, hybrids, and tandem arrangements must be stated. |
| *CYP2D6* | *67 | — | Structural/CNV or complex star-allele representation | Decreased function or reduced-function structural allele | May contribute to intermediate metabolizer phenotype depending on diplotype and structural configuration. | Codeine; tramadol; antidepressants; antipsychotics; tamoxifen; atomoxetine; ondansetron | Guideline available | Guideline available | 0.37% (0.06–2.05) | Moderate | Supplementary annotation; not displayed as a separate main-text representative. | Moderate | Interpret within a resolved diplotype and activity-score framework; assay coverage for copy number, hybrids, and tandem arrangements must be stated. |
| *CYP2D6* | *68-*4 | — | Structural/CNV or complex star-allele representation | No function or likely no function | May contribute to poor metabolizer phenotype when present with another no-function allele; exact phenotype requires diplotype interpretation. | Codeine; tramadol; antidepressants; antipsychotics; tamoxifen; atomoxetine; ondansetron | Guideline available | Guideline available | 0.37% (0.06–2.05) | Moderate | Supplementary annotation; not displayed as a separate main-text representative. | Moderate | Interpret within a resolved diplotype and activity-score framework; assay coverage for copy number, hybrids, and tandem arrangements must be stated. |
| *CYP2D6* | *86 | — | Structural/CNV or complex star-allele representation | No function or likely no function | May contribute to poor metabolizer phenotype when present with another no-function allele; exact phenotype requires diplotype interpretation. | Codeine; tramadol; antidepressants; antipsychotics; tamoxifen; atomoxetine; ondansetron | Guideline available | Guideline available | 0.37% (0.06–2.05) | Moderate | Supplementary annotation; not displayed as a separate main-text representative. | Moderate | Interpret within a resolved diplotype and activity-score framework; assay coverage for copy number, hybrids, and tandem arrangements must be stated. |
| *CYP3A5* | *6 | rs10264272 | Single-rsID variant | No function | No-function CYP3A5 allele; contributes to CYP3A5 non-expresser phenotype depending on diplotype. | Tacrolimus | Guideline available | Guideline available | 0.00% (0.00–2.60) | Moderate | Supplementary annotation; not displayed as a separate main-text representative. | High | Source-level representation retained for traceability; it should not be interpreted as an independent locus when another synonymous or structurally related representation is present. |
| *CYP3A5* | *7 | rs41303343 | Single-rsID variant | No function | No-function CYP3A5 allele; contributes to CYP3A5 non-expresser phenotype depending on diplotype. | Tacrolimus | Guideline available | Guideline available | 0.00% (0.00–2.60) | Moderate | Supplementary annotation; not displayed as a separate main-text representative. | High | Source-level representation retained for traceability; it should not be interpreted as an independent locus when another synonymous or structurally related representation is present. |
| *CYP3A5* | *8 | rs55817950 | Single-rsID variant | No function | No-function CYP3A5 allele; contributes to CYP3A5 non-expresser phenotype depending on diplotype. | Tacrolimus | Guideline available | Guideline available | 0.00% (0.00–2.60) | Moderate | Supplementary annotation; not displayed as a separate main-text representative. | High | Source-level representation retained for traceability; it should not be interpreted as an independent locus when another synonymous or structurally related representation is present. |
| *NUDT15* | rs116855232-containing alleles (*2/*3) | rs116855232 | Single-rsID variant | No or decreased function | Reduced NUDT15 activity; increased thiopurine myelotoxicity risk, especially relevant in Asian populations | Azathioprine; mercaptopurine; thioguanine | Guideline available | Guideline available | 4.50% (2.39–8.33) | Moderate | Table 4/Figure 5 primary representation: 4.50% (95% CI 2.39–8.33). | High | A single variant may be insufficient for phenotype assignment; interpret in the relevant haplotype, diplotype, structural, and drug-specific context. |
| *SLCO1B1* | *5 | rs4149056 | Single-rsID variant | Decreased function | Reduced hepatic uptake transporter function; increased systemic statin exposure and myopathy risk | Simvastatin; atorvastatin; rosuvastatin | Guideline available | Guideline available | 0.00% (0.00–1.81) | Moderate | Source-reported *5 haplotype/star-allele label involving rs4149056; not displayed as an independent c.521T>C estimate. | High | The source label *5 is haplotype-dependent and is not interchangeable with a stand-alone rs4149056 frequency. |
| *TPMT* | c.719A>G | rs1142345 | Single-rsID variant | No or decreased function | Reduced thiopurine methyltransferase activity; increased thiopurine myelotoxicity risk | Azathioprine; mercaptopurine; thioguanine | Guideline available | Guideline available | 2.50% (1.07–5.72) | Moderate | Table 4/Figure 5 primary representative (c.719A>G): 2.50% (95% CI 1.07–5.72). | High | A single variant may be insufficient for phenotype assignment; interpret in the relevant haplotype, diplotype, structural, and drug-specific context. |
| *CYP2D6* | *2 | — | Haplotype/star-allele representation | Normal function | Typically contributes to normal CYP2D6 activity, but phenotype depends on diplotype and copy number. | Codeine; tramadol; antidepressants; antipsychotics; tamoxifen; atomoxetine; ondansetron | Guideline available | Guideline available | 7.94% (5.20–11.94) | Potentially actionable | Supplementary annotation; not displayed as a separate main-text representative. | Moderate | Interpret within a resolved diplotype and activity-score framework; assay coverage for copy number, hybrids, and tandem arrangements must be stated. |
| *CYP2D6* | *2 | — | Structural/CNV or complex star-allele representation | Normal function | Typically contributes to normal CYP2D6 activity, but phenotype depends on diplotype and copy number. | Codeine; tramadol; antidepressants; antipsychotics; tamoxifen; atomoxetine; ondansetron | Guideline available | Guideline available | 7.72% (5.10–11.51) | Potentially actionable | Supplementary annotation; not displayed as a separate main-text representative. | Moderate | Interpret within a resolved diplotype and activity-score framework; assay coverage for copy number, hybrids, and tandem arrangements must be stated. |
| *ABCB1* | 3435C>T | rs1045642 | Single-rsID variant | Uncertain or context-dependent | May influence P-glycoprotein expression or drug disposition; clinical actionability is less established | Digoxin; tacrolimus; antiepileptics; multiple P-gp substrates | No direct guideline | No direct guideline | 40.28% (32.62–48.44) | Population evidence / exploratory | Supplementary annotation; not displayed as a separate main-text representative. | Low | A single variant may be insufficient for phenotype assignment; interpret in the relevant haplotype, diplotype, structural, and drug-specific context. |
| *ABCG2* | rs2231142 | rs2231142 | Single-rsID variant | Decreased function | Reduced ABCG2 transport activity; may influence exposure to selected transporter substrates | Rosuvastatin; allopurinol; selected kinase inhibitors | No direct guideline | Drug-specific/variable | 36.00% (29.67–42.86) | Population evidence / exploratory | Supplementary annotation; not displayed as a separate main-text representative. | Moderate | A single variant may be insufficient for phenotype assignment; interpret in the relevant haplotype, diplotype, structural, and drug-specific context. |
| *CYP1B1* | Dup | — | Structural/CNV or complex star-allele representation | Copy-number gain / structural marker; clinical PGx function not established | No validated drug-response phenotype assignment for routine prescribing. | Not established | No direct guideline | No direct/unclear guideline | 0.97% (0.33–2.82) | Population evidence / exploratory | Supplementary annotation; not displayed as a separate main-text representative. | Low | A single variant may be insufficient for phenotype assignment; interpret in the relevant haplotype, diplotype, structural, and drug-specific context. |
| *CYP2A6* | *11 | rs28399447 | Single-rsID variant | Decreased or uncertain function | May reduce CYP2A6 activity depending on haplotype context; clinical dosing actionability remains limited. | Nicotine; tegafur and selected CYP2A6 substrates | No direct guideline | No direct/unclear guideline | 1.50% (0.51–4.32) | Population evidence / exploratory | Supplementary annotation; not displayed as a separate main-text representative. | Moderate | A single variant may be insufficient for phenotype assignment; interpret in the relevant haplotype, diplotype, structural, and drug-specific context. |
| *CYP2A6* | *15 | rs199916117 | Single-rsID variant | Decreased or uncertain function | May reduce CYP2A6 activity depending on haplotype context; clinical dosing actionability remains limited. | Nicotine; tegafur and selected CYP2A6 substrates | No direct guideline | No direct/unclear guideline | 0.50% (0.09–2.78) | Population evidence / exploratory | Supplementary annotation; not displayed as a separate main-text representative. | Low | A single variant may be insufficient for phenotype assignment; interpret in the relevant haplotype, diplotype, structural, and drug-specific context. |
| *CYP2A6* | *4 | — | Structural/CNV or complex star-allele representation | No function / gene deletion | Reduced or absent CYP2A6 activity; may affect nicotine metabolism and selected CYP2A6 substrates, but prescribing actionability is not guideline-level. | Nicotine; tegafur and selected CYP2A6 substrates | No direct guideline | No direct/unclear guideline | 11.81% (7.50–18.09) | Population evidence / exploratory | Supplementary annotation; not displayed as a separate main-text representative. | Moderate | A single variant may be insufficient for phenotype assignment; interpret in the relevant haplotype, diplotype, structural, and drug-specific context. |
| *CYP2A6* | *5 | rs5031017 | Single-rsID variant | Decreased or uncertain function | May reduce CYP2A6 activity depending on haplotype context; clinical dosing actionability remains limited. | Nicotine; tegafur and selected CYP2A6 substrates | No direct guideline | No direct/unclear guideline | 14.58% (9.74–21.27) | Population evidence / exploratory | Supplementary annotation; not displayed as a separate main-text representative. | Moderate | A single variant may be insufficient for phenotype assignment; interpret in the relevant haplotype, diplotype, structural, and drug-specific context. |
| *CYP2A6* | Del | — | Structural/CNV or complex star-allele representation | No function / gene deletion | Reduced or absent CYP2A6 activity; may affect nicotine metabolism and selected CYP2A6 substrates, but prescribing actionability is not guideline-level. | Nicotine; tegafur and selected CYP2A6 substrates | No direct guideline | No direct/unclear guideline | 11.04% (8.01–15.03) | Population evidence / exploratory | Supplementary annotation; not displayed as a separate main-text representative. | Moderate | A single variant may be insufficient for phenotype assignment; interpret in the relevant haplotype, diplotype, structural, and drug-specific context. |
| *CYP2A6* | rs1809810 | rs1809810 | Single-rsID variant | Uncertain function | Population marker in CYP2A6; independent clinical actionability is not established. | Nicotine; tegafur and selected CYP2A6 substrates | No direct guideline | No direct/unclear guideline | 2.00% (0.78–5.03) | Population evidence / exploratory | Supplementary annotation; not displayed as a separate main-text representative. | Low | A single variant may be insufficient for phenotype assignment; interpret in the relevant haplotype, diplotype, structural, and drug-specific context. |
| *CYP2B6* | *2 | rs8192709 | Single-rsID variant | Uncertain or context-dependent function | Population marker within CYP2B6; independent clinical dosing actionability is not established. | Efavirenz; bupropion; methadone | No direct guideline | No direct/unclear guideline | 5.50% (3.10–9.58) | Population evidence / exploratory | Supplementary annotation; not displayed as a separate main-text representative. | Low | A single variant may be insufficient for phenotype assignment; interpret in the relevant haplotype, diplotype, structural, and drug-specific context. |
| *CYP2B6* | *4 | rs2279343 | Single-rsID variant | Increased or normal-to-increased function | May contribute to increased CYP2B6 activity in some allele definitions; independent dosing actionability is less direct than rs3745274-based phenotype assignment. | Efavirenz; bupropion; methadone | No direct/unclear guideline | No direct/unclear guideline | 35.42% (28.08–43.51) | Population evidence / exploratory | Supplementary annotation; not displayed as a separate main-text representative. | Moderate | A single variant may be insufficient for phenotype assignment; interpret in the relevant haplotype, diplotype, structural, and drug-specific context. |
| *CYP2B6* | *5 | rs3211371 | Single-rsID variant | Decreased or uncertain function | May alter CYP2B6 activity; variant-specific dosing actionability is not established as a standalone marker. | Efavirenz; bupropion; methadone | No direct/unclear guideline | No direct/unclear guideline | 0.00% (0.00–2.60) | Population evidence / exploratory | Supplementary annotation; not displayed as a separate main-text representative. | Low | A single variant may be insufficient for phenotype assignment; interpret in the relevant haplotype, diplotype, structural, and drug-specific context. |
| *CYP2C19* | *26 | rs375781227 | Single-rsID variant | Uncertain or decreased function candidate | Rare CYP2C19 allele; phenotype effect should be confirmed against PharmVar before clinical use. | Clopidogrel; proton pump inhibitors; antidepressants | No direct/unclear guideline | No direct/unclear guideline | 0.50% (0.09–2.78) | Population evidence / exploratory | Supplementary annotation; not displayed as a separate main-text representative. | Low | A single variant may be insufficient for phenotype assignment; interpret in the relevant haplotype, diplotype, structural, and drug-specific context. |
| *CYP2C19* | rs3758581 | rs3758581 | Single-rsID variant | Uncertain function / population marker | Not a primary CYP2C19 star-allele marker for routine clinical interpretation in this dataset. | Clopidogrel; proton pump inhibitors; antidepressants | No direct guideline | No direct/unclear guideline | 97.50% (94.28–98.93) | Population evidence / exploratory | Supplementary annotation; not displayed as a separate main-text representative. | Low | A single variant may be insufficient for phenotype assignment; interpret in the relevant haplotype, diplotype, structural, and drug-specific context. |
| *CYP2C8* | *11 | rs78637571 | Single-rsID variant | Uncertain or decreased function candidate | Potential CYP2C8 star-allele marker; clinical phenotype effect should be verified before implementation. | Paclitaxel; amodiaquine; repaglinide; NSAIDs | No direct guideline | No direct/unclear guideline | 1.00% (0.27–3.57) | Population evidence / exploratory | Supplementary annotation; not displayed as a separate main-text representative. | Low | A single variant may be insufficient for phenotype assignment; interpret in the relevant haplotype, diplotype, structural, and drug-specific context. |
| *CYP2C9* | *13 | rs72558187 | Single-rsID variant | Decreased function | Reduced CYP2C9 activity reported for CYP2C9*13; may contribute to lower metabolic capacity depending on diplotype, but guideline dosing support is less established than *2/*3. | Warfarin; phenytoin; NSAIDs | No direct/unclear guideline | No direct/unclear guideline | 0.79% (0.22–2.83) | Population evidence / exploratory | Supplementary annotation; not displayed as a separate main-text representative. | Moderate | A single variant may be insufficient for phenotype assignment; interpret in the relevant haplotype, diplotype, structural, and drug-specific context. |
| *CYP2C9* | *13 | rs72558187 | Single-rsID variant | Decreased function | Reduced CYP2C9 activity reported for CYP2C9*13; may contribute to lower metabolic capacity depending on diplotype, but guideline dosing support is less established than *2/*3. | Warfarin; phenytoin; NSAIDs | No direct/unclear guideline | No direct/unclear guideline | 0.50% (0.09–2.78) | Population evidence / exploratory | Supplementary annotation; not displayed as a separate main-text representative. | Moderate | A single variant may be insufficient for phenotype assignment; interpret in the relevant haplotype, diplotype, structural, and drug-specific context. |
| *CYP2C9* | Del | — | Structural/CNV or complex star-allele representation | Structural marker; function not established | No guideline-level phenotype assignment for routine prescribing. | Warfarin; phenytoin; NSAIDs | No direct guideline | No direct/unclear guideline | 0.97% (0.33–2.82) | Population evidence / exploratory | Supplementary annotation; not displayed as a separate main-text representative. | Low | A single variant may be insufficient for phenotype assignment; interpret in the relevant haplotype, diplotype, structural, and drug-specific context. |
| *CYP2D6* | *21B | — | Haplotype/star-allele representation | Uncertain or not established | No guideline-level phenotype assignment is currently assigned in this curation file. | Not established | No direct guideline | No direct/unclear guideline | 0.40% (0.07–2.21) | Population evidence / exploratory | Supplementary annotation; not displayed as a separate main-text representative. | Low | Interpret within a resolved diplotype and activity-score framework; assay coverage for copy number, hybrids, and tandem arrangements must be stated. |
| *CYP2D6* | G4268C | rs1135840 | Single-rsID variant | Haplotype-defining marker; standalone function not assigned | Variant may occur within CYP2D6 star-allele definitions; it should not be interpreted independently as a prescribing marker. | Codeine; tramadol; antidepressants; antipsychotics; tamoxifen; atomoxetine; ondansetron | No direct/unclear guideline | No direct/unclear guideline | 73.25% (68.71–77.35) | Population evidence / exploratory | Supplementary annotation; not displayed as a separate main-text representative. | Low | A single variant may be insufficient for phenotype assignment; interpret in the relevant haplotype, diplotype, structural, and drug-specific context. |
| *CYP2E1* | *5B | rs2031920 | Single-rsID variant | Uncertain or altered transcription/activity marker | CYP2E1 polymorphisms may affect enzyme expression or activity, but routine genotype-guided prescribing is not established. | Ethanol-related metabolism; anesthetics; selected xenobiotics | No direct guideline | No direct/unclear guideline | 17.19% (12.51–23.15) | Population evidence / exploratory | Supplementary annotation; not displayed as a separate main-text representative. | Low | A single variant may be insufficient for phenotype assignment; interpret in the relevant haplotype, diplotype, structural, and drug-specific context. |
| *CYP2E1* | *7B | rs6413420 | Single-rsID variant | Uncertain or altered transcription/activity marker | CYP2E1 polymorphisms may affect enzyme expression or activity, but routine genotype-guided prescribing is not established. | Ethanol-related metabolism; anesthetics; selected xenobiotics | No direct guideline | No direct/unclear guideline | 0.00% (0.00–1.96) | Population evidence / exploratory | Supplementary annotation; not displayed as a separate main-text representative. | Low | A single variant may be insufficient for phenotype assignment; interpret in the relevant haplotype, diplotype, structural, and drug-specific context. |
| *CYP2E1* | Del | — | Structural/CNV or complex star-allele representation | Uncertain or altered transcription/activity marker | CYP2E1 polymorphisms may affect enzyme expression or activity, but routine genotype-guided prescribing is not established. | Ethanol-related metabolism; anesthetics; selected xenobiotics | No direct guideline | No direct/unclear guideline | 0.32% (0.06–1.82) | Population evidence / exploratory | Supplementary annotation; not displayed as a separate main-text representative. | Low | A single variant may be insufficient for phenotype assignment; interpret in the relevant haplotype, diplotype, structural, and drug-specific context. |
| *CYP2E1* | Dup | — | Structural/CNV or complex star-allele representation | Uncertain or altered transcription/activity marker | CYP2E1 polymorphisms may affect enzyme expression or activity, but routine genotype-guided prescribing is not established. | Ethanol-related metabolism; anesthetics; selected xenobiotics | No direct guideline | No direct/unclear guideline | 0.97% (0.33–2.82) | Population evidence / exploratory | Supplementary annotation; not displayed as a separate main-text representative. | Low | A single variant may be insufficient for phenotype assignment; interpret in the relevant haplotype, diplotype, structural, and drug-specific context. |
| *CYP2J2* | *8 | — | Non-rsID representation | Uncertain function | No routine pharmacogenetic prescribing implication is established. | Not established | No direct guideline | No direct/unclear guideline | 0.00% (0.00–1.19) | Population evidence / exploratory | Supplementary annotation; not displayed as a separate main-text representative. | Low | A single variant may be insufficient for phenotype assignment; interpret in the relevant haplotype, diplotype, structural, and drug-specific context. |
| *CYP2J2* | *9 | — | Non-rsID representation | Uncertain function | No routine pharmacogenetic prescribing implication is established. | Not established | No direct guideline | No direct/unclear guideline | 0.00% (0.00–1.19) | Population evidence / exploratory | Supplementary annotation; not displayed as a separate main-text representative. | Low | A single variant may be insufficient for phenotype assignment; interpret in the relevant haplotype, diplotype, structural, and drug-specific context. |
| *CYP3A4* | *18 | — | Haplotype/star-allele representation | Altered function candidate | Reported CYP3A4 star allele; routine clinical dosing actionability is not established. | Tacrolimus; statins; calcium-channel blockers; many CYP3A4 substrates | No direct guideline | No direct/unclear guideline | 0.79% (0.22–2.83) | Population evidence / exploratory | Supplementary annotation; not displayed as a separate main-text representative. | Low | A single variant may be insufficient for phenotype assignment; interpret in the relevant haplotype, diplotype, structural, and drug-specific context. |
| *CYP3A4* | *1B | rs2740574 | Single-rsID variant | Regulatory variant; uncertain clinical function | May influence CYP3A4 expression in some contexts; routine genotype-guided dosing is not established. | Tacrolimus; statins; calcium-channel blockers; many CYP3A4 substrates | No direct guideline | No direct/unclear guideline | 2.08% (0.71–5.95) | Population evidence / exploratory | Supplementary annotation; not displayed as a separate main-text representative. | Low | A single variant may be insufficient for phenotype assignment; interpret in the relevant haplotype, diplotype, structural, and drug-specific context. |
| *DPYD* | rs17376848 | rs17376848 | Single-rsID variant | Uncertain or not guideline-defining DPYD marker | This variant is not one of the core DPYD no-function/decreased-function alleles typically used for fluoropyrimidine dose reduction; interpret as exploratory unless separately validated. | Fluorouracil; capecitabine; tegafur | No direct guideline | No direct/unclear guideline | 16.00% (11.57–21.71) | Population evidence / exploratory | Supplementary annotation; not displayed as a separate main-text representative. | Low | A single variant may be insufficient for phenotype assignment; interpret in the relevant haplotype, diplotype, structural, and drug-specific context. |
| *DPYD* | rs1801159 | rs1801159 | Single-rsID variant | Uncertain or not guideline-defining DPYD marker | This variant is not one of the core DPYD no-function/decreased-function alleles typically used for fluoropyrimidine dose reduction; interpret as exploratory unless separately validated. | Fluorouracil; capecitabine; tegafur | No direct guideline | No direct/unclear guideline | 22.50% (17.26–28.77) | Population evidence / exploratory | Supplementary annotation; not displayed as a separate main-text representative. | Low | A single variant may be insufficient for phenotype assignment; interpret in the relevant haplotype, diplotype, structural, and drug-specific context. |
| *DPYD* | rs1801160 | rs1801160 | Single-rsID variant | Uncertain or not guideline-defining DPYD marker | This variant is not one of the core DPYD no-function/decreased-function alleles typically used for fluoropyrimidine dose reduction; interpret as exploratory unless separately validated. | Fluorouracil; capecitabine; tegafur | No direct guideline | No direct/unclear guideline | 2.00% (0.78–5.03) | Population evidence / exploratory | Supplementary annotation; not displayed as a separate main-text representative. | Low | A single variant may be insufficient for phenotype assignment; interpret in the relevant haplotype, diplotype, structural, and drug-specific context. |
| *DPYD* | rs1801265 | rs1801265 | Single-rsID variant | Uncertain or not guideline-defining DPYD marker | This variant is not one of the core DPYD no-function/decreased-function alleles typically used for fluoropyrimidine dose reduction; interpret as exploratory unless separately validated. | Fluorouracil; capecitabine; tegafur | No direct guideline | No direct/unclear guideline | 17.00% (12.43–22.82) | Population evidence / exploratory | Supplementary annotation; not displayed as a separate main-text representative. | Low | A single variant may be insufficient for phenotype assignment; interpret in the relevant haplotype, diplotype, structural, and drug-specific context. |
| *DPYD* | rs2297595 | rs2297595 | Single-rsID variant | Uncertain or not guideline-defining DPYD marker | This variant is not one of the core DPYD no-function/decreased-function alleles typically used for fluoropyrimidine dose reduction; interpret as exploratory unless separately validated. | Fluorouracil; capecitabine; tegafur | No direct guideline | No direct/unclear guideline | 2.50% (1.07–5.72) | Population evidence / exploratory | Supplementary annotation; not displayed as a separate main-text representative. | Low | A single variant may be insufficient for phenotype assignment; interpret in the relevant haplotype, diplotype, structural, and drug-specific context. |
| *HNF4A* | G36S | — | Non-rsID representation | Diabetes-related genetic marker; pharmacogenetic function not established | No routine drug-response phenotype assignment for this variant in the current PGx framework. | Not established | No direct guideline | No direct/unclear guideline | 3.60% (1.97–6.49) | Population evidence / exploratory | Supplementary annotation; not displayed as a separate main-text representative. | Low | A single variant may be insufficient for phenotype assignment; interpret in the relevant haplotype, diplotype, structural, and drug-specific context. |
| *HNF4A* | G60D | — | Non-rsID representation | Diabetes-related genetic marker; pharmacogenetic function not established | No routine drug-response phenotype assignment for this variant in the current PGx framework. | Not established | No direct guideline | No direct/unclear guideline | 0.72% (0.20–2.58) | Population evidence / exploratory | Supplementary annotation; not displayed as a separate main-text representative. | Low | A single variant may be insufficient for phenotype assignment; interpret in the relevant haplotype, diplotype, structural, and drug-specific context. |
| *NAT2* | *12 | — | Haplotype/star-allele representation | Normal or rapid-acetylator haplotype marker | May contribute to normal/rapid acetylator phenotype depending on haplotype context. | Isoniazid; hydralazine; sulfonamides; selected aromatic amines | No direct guideline | No direct/unclear guideline | 3.47% (1.49–7.87) | Population evidence / exploratory | Supplementary annotation; not displayed as a separate main-text representative. | Moderate | A single variant may be insufficient for phenotype assignment; interpret in the relevant haplotype, diplotype, structural, and drug-specific context. |
| *NAT2* | *13 | — | Haplotype/star-allele representation | Normal or rapid-acetylator haplotype marker | May contribute to normal/rapid acetylator phenotype depending on haplotype context. | Isoniazid; hydralazine; sulfonamides; selected aromatic amines | No direct guideline | No direct/unclear guideline | 30.56% (23.62–38.50) | Population evidence / exploratory | Supplementary annotation; not displayed as a separate main-text representative. | Moderate | A single variant may be insufficient for phenotype assignment; interpret in the relevant haplotype, diplotype, structural, and drug-specific context. |
| *NAT2* | *19 | — | Haplotype/star-allele representation | Uncertain function haplotype marker | NAT2 phenotype effect requires haplotype-level assignment; do not interpret as a standalone marker. | Isoniazid; hydralazine; sulfonamides; selected aromatic amines | No direct guideline | No direct/unclear guideline | 1.39% (0.38–4.92) | Population evidence / exploratory | Supplementary annotation; not displayed as a separate main-text representative. | Low | A single variant may be insufficient for phenotype assignment; interpret in the relevant haplotype, diplotype, structural, and drug-specific context. |
| *NAT2* | *5 | — | Haplotype/star-allele representation | Decreased function / slow-acetylator allele marker | Contributes to NAT2 slow-acetylator phenotype depending on haplotype; may influence isoniazid exposure and hepatotoxicity risk. | Isoniazid; hydralazine; sulfonamides; selected aromatic amines | No direct guideline | No direct/unclear guideline | 2.78% (1.09–6.92) | Population evidence / exploratory | Supplementary annotation; not displayed as a separate main-text representative. | Moderate | A single variant may be insufficient for phenotype assignment; interpret in the relevant haplotype, diplotype, structural, and drug-specific context. |
| *NAT2* | *5 | rs1801280 | Single-rsID variant | Decreased function / slow-acetylator allele marker | Contributes to NAT2 slow-acetylator phenotype depending on haplotype; may influence isoniazid exposure and hepatotoxicity risk. | Isoniazid; hydralazine; sulfonamides; selected aromatic amines | No direct guideline | No direct/unclear guideline | 5.97% (3.96–8.89) | Population evidence / exploratory | Supplementary annotation; not displayed as a separate main-text representative. | Moderate | A single variant may be insufficient for phenotype assignment; interpret in the relevant haplotype, diplotype, structural, and drug-specific context. |
| *NAT2* | *6 | rs1799930 | Single-rsID variant | Decreased function / slow-acetylator allele marker | Contributes to NAT2 slow-acetylator phenotype depending on haplotype; may influence isoniazid exposure and hepatotoxicity risk. | Isoniazid; hydralazine; sulfonamides; selected aromatic amines | No direct guideline | No direct/unclear guideline | 27.00% (18.69–37.31) | Population evidence / exploratory | Supplementary annotation; not displayed as a separate main-text representative. | Moderate | A single variant may be insufficient for phenotype assignment; interpret in the relevant haplotype, diplotype, structural, and drug-specific context. |
| *NAT2* | *6A | — | Haplotype/star-allele representation | Decreased function / slow-acetylator allele marker | Contributes to NAT2 slow-acetylator phenotype depending on haplotype; may influence isoniazid exposure and hepatotoxicity risk. | Isoniazid; hydralazine; sulfonamides; selected aromatic amines | No direct guideline | No direct/unclear guideline | 17.36% (12.04–24.37) | Population evidence / exploratory | Supplementary annotation; not displayed as a separate main-text representative. | Moderate | A single variant may be insufficient for phenotype assignment; interpret in the relevant haplotype, diplotype, structural, and drug-specific context. |
| *NAT2* | *7 | — | Haplotype/star-allele representation | Decreased function / slow-acetylator allele marker | Contributes to NAT2 slow-acetylator phenotype depending on haplotype; may influence isoniazid exposure and hepatotoxicity risk. | Isoniazid; hydralazine; sulfonamides; selected aromatic amines | No direct guideline | No direct/unclear guideline | 0.00% (0.00–2.60) | Population evidence / exploratory | Supplementary annotation; not displayed as a separate main-text representative. | Moderate | A single variant may be insufficient for phenotype assignment; interpret in the relevant haplotype, diplotype, structural, and drug-specific context. |
| *NAT2* | *7 | rs1799931 | Single-rsID variant | Decreased function / slow-acetylator allele marker | Contributes to NAT2 slow-acetylator phenotype depending on haplotype; may influence isoniazid exposure and hepatotoxicity risk. | Isoniazid; hydralazine; sulfonamides; selected aromatic amines | No direct guideline | No direct/unclear guideline | 17.75% (13.51–22.95) | Population evidence / exploratory | Supplementary annotation; not displayed as a separate main-text representative. | Moderate | A single variant may be insufficient for phenotype assignment; interpret in the relevant haplotype, diplotype, structural, and drug-specific context. |
| *NAT2* | rs1208 | rs1208 | Single-rsID variant | Normal or rapid-acetylator haplotype marker | May contribute to normal/rapid acetylator phenotype depending on haplotype context. | Isoniazid; hydralazine; sulfonamides; selected aromatic amines | No direct guideline | No direct/unclear guideline | 91.00% (86.22–94.23) | Population evidence / exploratory | Supplementary annotation; not displayed as a separate main-text representative. | Moderate | A single variant may be insufficient for phenotype assignment; interpret in the relevant haplotype, diplotype, structural, and drug-specific context. |
| *PON1* | Q192R | rs662 | Single-rsID variant | Uncertain or context-dependent function | PON1 Q192R has been studied for clopidogrel response, but evidence is inconsistent and not guideline-actionable. | Clopidogrel | No direct guideline | No direct/unclear guideline | 33.08% (28.63–37.86) | Population evidence / exploratory | Supplementary annotation; not displayed as a separate main-text representative. | Low | A single variant may be insufficient for phenotype assignment; interpret in the relevant haplotype, diplotype, structural, and drug-specific context. |
| *PSORS1C1* | rs9263726 | rs9263726 | Single-rsID variant | Risk-marker candidate | Reported as a potential marker for allopurinol-related severe cutaneous adverse reactions in some populations, but not a broadly established prescribing marker compared with HLA-B*58:01. | Allopurinol | No direct guideline | No direct/unclear guideline | 7.89% (5.22–11.77) | Population evidence / exploratory | Supplementary annotation; not displayed as a separate main-text representative. | Low | A single variant may be insufficient for phenotype assignment; interpret in the relevant haplotype, diplotype, structural, and drug-specific context. |
| *SLCO1B1* | c.388A>G | rs2306283 | Single-rsID variant | Normal or context-dependent transporter function | SLCO1B1 c.388A>G may contribute to haplotype definitions but is not the primary CPIC statin myopathy risk marker by itself. | Simvastatin; atorvastatin; rosuvastatin | No direct guideline | No direct/unclear guideline | 78.85% (72.80–83.85) | Population evidence / exploratory | Contributes to harmonized Table 2 estimate: 75.95% (95% CI 69.75–81.22), k=2, N=204. | Moderate | Synonymous source labels sharing the same rsID and effect-allele orientation were collapsed only for the main-text harmonized estimate. |
| *SLCO1B1* | rs2306283 | rs2306283 | Single-rsID variant | Normal or context-dependent transporter function | SLCO1B1 c.388A>G may contribute to haplotype definitions but is not the primary CPIC statin myopathy risk marker by itself. | Simvastatin; atorvastatin; rosuvastatin | No direct guideline | No direct/unclear guideline | 73.00% (66.46–78.68) | Population evidence / exploratory | Contributes to harmonized Table 2 estimate: 75.95% (95% CI 69.75–81.22), k=2, N=204. | Moderate | Synonymous source labels sharing the same rsID and effect-allele orientation were collapsed only for the main-text harmonized estimate. |
| *UGT1A1* | c.189C>T | rs191471887 | Single-rsID variant | Uncertain function | Rare UGT1A1 coding/promoter-region marker; no validated routine dosing implication assigned here. | Irinotecan; atazanavir | No direct guideline | No direct/unclear guideline | 0.00% (0.00–3.70) | Population evidence / exploratory | Supplementary annotation; not displayed as a separate main-text representative. | Low | A single variant may be insufficient for phenotype assignment; interpret in the relevant haplotype, diplotype, structural, and drug-specific context. |
| *UGT1A1* | rs1042640C>G | rs1042640 | Single-rsID variant | Uncertain or haplotype-associated UGT1A1 marker | May be associated with UGT1A1 haplotype or expression context, but not a primary standalone guideline marker compared with *6 and *28. | Irinotecan; atazanavir | No direct guideline | No direct/unclear guideline | 25.00% (19.51–31.43) | Population evidence / exploratory | Supplementary annotation; not displayed as a separate main-text representative. | Low | A single variant may be insufficient for phenotype assignment; interpret in the relevant haplotype, diplotype, structural, and drug-specific context. |
| *UGT1A1* | rs10929303C>T | rs10929303 | Single-rsID variant | Uncertain or haplotype-associated UGT1A1 marker | May be associated with UGT1A1 haplotype or expression context, but not a primary standalone guideline marker compared with *6 and *28. | Irinotecan; atazanavir | No direct guideline | No direct/unclear guideline | 25.00% (19.51–31.43) | Population evidence / exploratory | Supplementary annotation; not displayed as a separate main-text representative. | Low | A single variant may be insufficient for phenotype assignment; interpret in the relevant haplotype, diplotype, structural, and drug-specific context. |
| *UGT1A1* | rs8330C>G | rs8330 | Single-rsID variant | Uncertain or haplotype-associated UGT1A1 marker | May be associated with UGT1A1 haplotype or expression context, but not a primary standalone guideline marker compared with *6 and *28. | Irinotecan; atazanavir | No direct guideline | No direct/unclear guideline | 25.00% (19.51–31.43) | Population evidence / exploratory | Supplementary annotation; not displayed as a separate main-text representative. | Low | A single variant may be insufficient for phenotype assignment; interpret in the relevant haplotype, diplotype, structural, and drug-specific context. |
| *VKORC1* | 1542G>C | rs8050894 | Single-rsID variant | VKORC1 haplotype marker / warfarin sensitivity tag | Associated with VKORC1 warfarin-dose haplotype in some datasets; not preferred as the primary dosing marker over -1639G>A or rs9934438. | Warfarin | No direct/unclear guideline | No direct/unclear guideline | 85.71% (77.44–91.30) | Population evidence / exploratory | Supplementary annotation; not displayed as a separate main-text representative. | Moderate | A single variant may be insufficient for phenotype assignment; interpret in the relevant haplotype, diplotype, structural, and drug-specific context. |
| *VKORC1* | 2255C>T | rs2359612 | Single-rsID variant | VKORC1 haplotype marker / warfarin sensitivity tag | Associated with VKORC1 warfarin-dose haplotype in some datasets; not preferred as the primary dosing marker over -1639G>A or rs9934438. | Warfarin | No direct/unclear guideline | No direct/unclear guideline | 85.71% (77.44–91.30) | Population evidence / exploratory | Supplementary annotation; not displayed as a separate main-text representative. | Moderate | A single variant may be insufficient for phenotype assignment; interpret in the relevant haplotype, diplotype, structural, and drug-specific context. |
| *VKORC1* | 3730G>A | rs7294 | Single-rsID variant | VKORC1 haplotype marker; independent actionability uncertain | May be part of VKORC1 haplotype background; independent clinical dosing role is less established than -1639G>A/rs9934438. | Warfarin | No direct guideline | No direct/unclear guideline | 14.29% (8.70–22.56) | Population evidence / exploratory | Supplementary annotation; not displayed as a separate main-text representative. | Low | A single variant may be insufficient for phenotype assignment; interpret in the relevant haplotype, diplotype, structural, and drug-specific context. |
| *VKORC1* | 497T>G | rs2884737 | Single-rsID variant | VKORC1 haplotype marker; independent actionability uncertain | May be part of VKORC1 haplotype background; independent clinical dosing role is less established than -1639G>A/rs9934438. | Warfarin | No direct guideline | No direct/unclear guideline | 1.02% (0.18–5.56) | Population evidence / exploratory | Supplementary annotation; not displayed as a separate main-text representative. | Low | A single variant may be insufficient for phenotype assignment; interpret in the relevant haplotype, diplotype, structural, and drug-specific context. |

Abbreviations: CPIC, Clinical Pharmacogenetics Implementation Consortium; DPWG, Dutch Pharmacogenetics Working Group; PGx, pharmacogenomics. Guideline availability refers to the relevant gene–drug context and does not imply that every listed variant alone is sufficient for phenotype assignment.
