# Supplementary material for: Vietnamese pharmacogenomic variation in global context: a systematic review and meta-analysis for clinical implementation
Source: Front Pharmacol. 2026 Sep 3;17:1921144. doi: 10.3389/fphar.2026.1921144 (PMC13582518; doi:10.3389/fphar.2026.1921144)
Supplement: Supplementary file 8 [file Table8.docx]

**Supplementary Table S8. Non-rsID, star-allele, haplotype, copy-number, HLA, and structural representations retained for clinical interpretation**

The 42 representations in this table were preserved in their clinically meaningful source form rather than forced into single-rsID benchmarking. Frequencies therefore describe the reported allele, haplotype, copy-number state, tandem arrangement, hybrid, repeat polymorphism, or HLA allele—not an interchangeable single-marker surrogate.

For CYP2D6, implementation requires an assay that states star-allele coverage, copy-number resolution, hybrid/tandem detection, phase assumptions, and residual uncertainty. Duplicate source representations of the same biological construct are retained for traceability but are explicitly distinguished from the pooled primary representation.

| **Gene** | **Allele/variant representation** | **Representation class** | **Functional interpretation** | **Expected phenotype implication** | **Relevant drug(s)** | **Guideline context** | **Source-level Vietnamese estimate** | **Clinical priority** | **Use in main manuscript** | **Why direct single-rsID benchmarking was not used** | **Assay/reporting recommendation** |
| --- | --- | --- | --- | --- | --- | --- | --- | --- | --- | --- | --- |
| *CYP2D6* | *10 | Structural/CNV or complex star-allele representation | Decreased function | Reduced-function allele; common in East Asian populations and contributes to intermediate metabolizer phenotype | Codeine; tramadol; antidepressants; antipsychotics; tamoxifen | CPIC: Guideline available; DPWG: Guideline available | 43.75% (37.98–49.69) | High | Primary Table 4/Figure 5 star-allele estimate: 43.75% (95% CI 37.98–49.69). | The reported unit is a star allele, haplotype, copy-number state, hybrid, deletion, or tandem structure rather than one biologically equivalent biallelic rsID. | Use a validated CYP2D6 assay with star-allele, copy-number, hybrid/tandem, and phase resolution; report diplotype, activity score, assay coverage, and residual uncertainty. |
| *CYP2D6* | *10B | Haplotype/star-allele representation | Decreased function or reduced-function structural allele | May contribute to intermediate metabolizer phenotype depending on diplotype and structural configuration. | Codeine; tramadol; antidepressants; antipsychotics; tamoxifen; atomoxetine; ondansetron | CPIC: Guideline available; DPWG: Guideline available | 55.95% (49.78–61.95) | High | Supplementary complex-variant evidence; not displayed as a separate main-text representative. | The reported unit is a star allele, haplotype, copy-number state, hybrid, deletion, or tandem structure rather than one biologically equivalent biallelic rsID. | Use a validated CYP2D6 assay with star-allele, copy-number, hybrid/tandem, and phase resolution; report diplotype, activity score, assay coverage, and residual uncertainty. |
| *CYP2D6* | *36-*10 | Structural/CNV or complex star-allele representation | Decreased function or reduced-function structural allele | May contribute to intermediate metabolizer phenotype depending on diplotype and structural configuration. | Codeine; tramadol; antidepressants; antipsychotics; tamoxifen; atomoxetine; ondansetron | CPIC: Guideline available; DPWG: Guideline available | 11.40% (8.15–15.72) | High | Supplementary complex-variant evidence; not displayed as a separate main-text representative. | The reported unit is a star allele, haplotype, copy-number state, hybrid, deletion, or tandem structure rather than one biologically equivalent biallelic rsID. | Use a validated CYP2D6 assay with star-allele, copy-number, hybrid/tandem, and phase resolution; report diplotype, activity score, assay coverage, and residual uncertainty. |
| *CYP2D6* | *5 | Structural/CNV or complex star-allele representation | No function | Gene deletion; contributes to poor metabolizer phenotype depending on diplotype | Codeine; tramadol; antidepressants; antipsychotics; tamoxifen | CPIC: Guideline available; DPWG: Guideline available | 7.75% (4.38–13.34) | High | Source-level deletion representation retained for traceability; not displayed independently. | The reported unit is a star allele, haplotype, copy-number state, hybrid, deletion, or tandem structure rather than one biologically equivalent biallelic rsID. | Use a validated CYP2D6 assay with star-allele, copy-number, hybrid/tandem, and phase resolution; report diplotype, activity score, assay coverage, and residual uncertainty. |
| *CYP2D6* | *5 | Structural/CNV or complex star-allele representation | No function | Gene deletion; contributes to poor metabolizer phenotype depending on diplotype | Codeine; tramadol; antidepressants; antipsychotics; tamoxifen | CPIC: Guideline available; DPWG: Guideline available | 7.47% (5.51–10.06) | High | Primary pooled Table 4/Figure 5 estimate: 7.47% (95% CI 5.51–10.06). | The reported unit is a star allele, haplotype, copy-number state, hybrid, deletion, or tandem structure rather than one biologically equivalent biallelic rsID. | Use a validated CYP2D6 assay with star-allele, copy-number, hybrid/tandem, and phase resolution; report diplotype, activity score, assay coverage, and residual uncertainty. |
| *HLA-B* | *58:01 | Non-rsID representation | Risk allele | Strongly associated with allopurinol-induced severe cutaneous adverse reactions | Allopurinol | CPIC: Guideline available; DPWG: Guideline available | 6.02% (3.74–9.55) | High | Primary Table 4/Figure 5 representative: 6.02% (95% CI 3.74–9.55). | HLA allele nomenclature is not reducible to a single biallelic rsID frequency. | Use validated HLA typing with explicit allele resolution and quality control; report the allele using current HLA nomenclature. |
| *CYP2D6* | *10BX2 | Structural/CNV or complex star-allele representation | Duplicated decreased-function allele | May increase copy number of a reduced-function CYP2D6 allele; net phenotype depends on the full diplotype and copy-number structure. | Codeine; tramadol; antidepressants; antipsychotics; tamoxifen; atomoxetine; ondansetron | CPIC: Guideline available; DPWG: Guideline available | 0.40% (0.07–2.21) | Moderate | Supplementary complex-variant evidence; not displayed as a separate main-text representative. | The reported unit is a star allele, haplotype, copy-number state, hybrid, deletion, or tandem structure rather than one biologically equivalent biallelic rsID. | Use a validated CYP2D6 assay with star-allele, copy-number, hybrid/tandem, and phase resolution; report diplotype, activity score, assay coverage, and residual uncertainty. |
| *CYP2D6* | *13 | Structural/CNV or complex star-allele representation | No function or likely no function | May contribute to poor metabolizer phenotype when present with another no-function allele; exact phenotype requires diplotype interpretation. | Codeine; tramadol; antidepressants; antipsychotics; tamoxifen; atomoxetine; ondansetron | CPIC: Guideline available; DPWG: Guideline available | 0.74% (0.20–2.64) | Moderate | Supplementary complex-variant evidence; not displayed as a separate main-text representative. | The reported unit is a star allele, haplotype, copy-number state, hybrid, deletion, or tandem structure rather than one biologically equivalent biallelic rsID. | Use a validated CYP2D6 assay with star-allele, copy-number, hybrid/tandem, and phase resolution; report diplotype, activity score, assay coverage, and residual uncertainty. |
| *CYP2D6* | *14 | Structural/CNV or complex star-allele representation | Decreased function or reduced-function structural allele | May contribute to intermediate metabolizer phenotype depending on diplotype and structural configuration. | Codeine; tramadol; antidepressants; antipsychotics; tamoxifen; atomoxetine; ondansetron | CPIC: Guideline available; DPWG: Guideline available | 0.37% (0.06–2.05) | Moderate | Supplementary complex-variant evidence; not displayed as a separate main-text representative. | The reported unit is a star allele, haplotype, copy-number state, hybrid, deletion, or tandem structure rather than one biologically equivalent biallelic rsID. | Use a validated CYP2D6 assay with star-allele, copy-number, hybrid/tandem, and phase resolution; report diplotype, activity score, assay coverage, and residual uncertainty. |
| *CYP2D6* | *14B | Haplotype/star-allele representation | Decreased function or reduced-function structural allele | May contribute to intermediate metabolizer phenotype depending on diplotype and structural configuration. | Codeine; tramadol; antidepressants; antipsychotics; tamoxifen; atomoxetine; ondansetron | CPIC: Guideline available; DPWG: Guideline available | 0.79% (0.22–2.85) | Moderate | Supplementary complex-variant evidence; not displayed as a separate main-text representative. | The reported unit is a star allele, haplotype, copy-number state, hybrid, deletion, or tandem structure rather than one biologically equivalent biallelic rsID. | Use a validated CYP2D6 assay with star-allele, copy-number, hybrid/tandem, and phase resolution; report diplotype, activity score, assay coverage, and residual uncertainty. |
| *CYP2D6* | *15 | Structural/CNV or complex star-allele representation | No function or likely no function | May contribute to poor metabolizer phenotype when present with another no-function allele; exact phenotype requires diplotype interpretation. | Codeine; tramadol; antidepressants; antipsychotics; tamoxifen; atomoxetine; ondansetron | CPIC: Guideline available; DPWG: Guideline available | 0.37% (0.06–2.05) | Moderate | Supplementary complex-variant evidence; not displayed as a separate main-text representative. | The reported unit is a star allele, haplotype, copy-number state, hybrid, deletion, or tandem structure rather than one biologically equivalent biallelic rsID. | Use a validated CYP2D6 assay with star-allele, copy-number, hybrid/tandem, and phase resolution; report diplotype, activity score, assay coverage, and residual uncertainty. |
| *CYP2D6* | *1XN | Structural/CNV or complex star-allele representation | Increased function / copy-number gain | Increased CYP2D6 activity may contribute to ultrarapid metabolizer phenotype depending on duplicated allele activity. | Codeine; tramadol; antidepressants; antipsychotics; tamoxifen; atomoxetine; ondansetron | CPIC: Guideline available; DPWG: Guideline available | 0.40% (0.07–2.21) | Moderate | Supplementary complex-variant evidence; not displayed as a separate main-text representative. | The reported unit is a star allele, haplotype, copy-number state, hybrid, deletion, or tandem structure rather than one biologically equivalent biallelic rsID. | Use a validated CYP2D6 assay with star-allele, copy-number, hybrid/tandem, and phase resolution; report diplotype, activity score, assay coverage, and residual uncertainty. |
| *CYP2D6* | *36 | Structural/CNV or complex star-allele representation | No function or nonfunctional hybrid | Hybrid allele; interpretation depends on copy-number and tandem structure | Codeine; tramadol; antidepressants; antipsychotics; tamoxifen | CPIC: Guideline available; DPWG: Guideline available | 0.37% (0.06–2.05) | Moderate | Primary complex-allele representative in Table 4/Figure 5: 0.37% (95% CI 0.06–2.05). | The reported unit is a star allele, haplotype, copy-number state, hybrid, deletion, or tandem structure rather than one biologically equivalent biallelic rsID. | Use a validated CYP2D6 assay with star-allele, copy-number, hybrid/tandem, and phase resolution; report diplotype, activity score, assay coverage, and residual uncertainty. |
| *CYP2D6* | *36-*36-*10 | Structural/CNV or complex star-allele representation | Decreased function or reduced-function structural allele | May contribute to intermediate metabolizer phenotype depending on diplotype and structural configuration. | Codeine; tramadol; antidepressants; antipsychotics; tamoxifen; atomoxetine; ondansetron | CPIC: Guideline available; DPWG: Guideline available | 0.37% (0.06–2.05) | Moderate | Supplementary complex-variant evidence; not displayed as a separate main-text representative. | The reported unit is a star allele, haplotype, copy-number state, hybrid, deletion, or tandem structure rather than one biologically equivalent biallelic rsID. | Use a validated CYP2D6 assay with star-allele, copy-number, hybrid/tandem, and phase resolution; report diplotype, activity score, assay coverage, and residual uncertainty. |
| *CYP2D6* | *4 | Structural/CNV or complex star-allele representation | No function | Loss-of-function allele; contributes to poor metabolizer phenotype depending on diplotype | Codeine; tramadol; antidepressants; antipsychotics; tamoxifen | CPIC: Guideline available; DPWG: Guideline available | 0.74% (0.20–2.64) | Moderate | Primary Table 4/Figure 5 star-allele estimate: 0.74% (95% CI 0.20–2.64). | The reported unit is a star allele, haplotype, copy-number state, hybrid, deletion, or tandem structure rather than one biologically equivalent biallelic rsID. | Use a validated CYP2D6 assay with star-allele, copy-number, hybrid/tandem, and phase resolution; report diplotype, activity score, assay coverage, and residual uncertainty. |
| *CYP2D6* | *41 | Haplotype/star-allele representation | Decreased function or reduced-function structural allele | May contribute to intermediate metabolizer phenotype depending on diplotype and structural configuration. | Codeine; tramadol; antidepressants; antipsychotics; tamoxifen; atomoxetine; ondansetron | CPIC: Guideline available; DPWG: Guideline available | 4.37% (2.45–7.65) | Moderate | Supplementary complex-variant evidence; not displayed as a separate main-text representative. | The reported unit is a star allele, haplotype, copy-number state, hybrid, deletion, or tandem structure rather than one biologically equivalent biallelic rsID. | Use a validated CYP2D6 assay with star-allele, copy-number, hybrid/tandem, and phase resolution; report diplotype, activity score, assay coverage, and residual uncertainty. |
| *CYP2D6* | *49 | Haplotype/star-allele representation | Decreased function or reduced-function structural allele | May contribute to intermediate metabolizer phenotype depending on diplotype and structural configuration. | Codeine; tramadol; antidepressants; antipsychotics; tamoxifen; atomoxetine; ondansetron | CPIC: Guideline available; DPWG: Guideline available | 1.19% (0.41–3.44) | Moderate | Supplementary complex-variant evidence; not displayed as a separate main-text representative. | The reported unit is a star allele, haplotype, copy-number state, hybrid, deletion, or tandem structure rather than one biologically equivalent biallelic rsID. | Use a validated CYP2D6 assay with star-allele, copy-number, hybrid/tandem, and phase resolution; report diplotype, activity score, assay coverage, and residual uncertainty. |
| *CYP2D6* | *60 | Structural/CNV or complex star-allele representation | No function or likely no function | May contribute to poor metabolizer phenotype when present with another no-function allele; exact phenotype requires diplotype interpretation. | Codeine; tramadol; antidepressants; antipsychotics; tamoxifen; atomoxetine; ondansetron | CPIC: Guideline available; DPWG: Guideline available | 0.74% (0.20–2.64) | Moderate | Supplementary complex-variant evidence; not displayed as a separate main-text representative. | The reported unit is a star allele, haplotype, copy-number state, hybrid, deletion, or tandem structure rather than one biologically equivalent biallelic rsID. | Use a validated CYP2D6 assay with star-allele, copy-number, hybrid/tandem, and phase resolution; report diplotype, activity score, assay coverage, and residual uncertainty. |
| *CYP2D6* | *65 | Structural/CNV or complex star-allele representation | Decreased function or reduced-function structural allele | May contribute to intermediate metabolizer phenotype depending on diplotype and structural configuration. | Codeine; tramadol; antidepressants; antipsychotics; tamoxifen; atomoxetine; ondansetron | CPIC: Guideline available; DPWG: Guideline available | 2.94% (1.50–5.70) | Moderate | Supplementary complex-variant evidence; not displayed as a separate main-text representative. | The reported unit is a star allele, haplotype, copy-number state, hybrid, deletion, or tandem structure rather than one biologically equivalent biallelic rsID. | Use a validated CYP2D6 assay with star-allele, copy-number, hybrid/tandem, and phase resolution; report diplotype, activity score, assay coverage, and residual uncertainty. |
| *CYP2D6* | *67 | Structural/CNV or complex star-allele representation | Decreased function or reduced-function structural allele | May contribute to intermediate metabolizer phenotype depending on diplotype and structural configuration. | Codeine; tramadol; antidepressants; antipsychotics; tamoxifen; atomoxetine; ondansetron | CPIC: Guideline available; DPWG: Guideline available | 0.37% (0.06–2.05) | Moderate | Supplementary complex-variant evidence; not displayed as a separate main-text representative. | The reported unit is a star allele, haplotype, copy-number state, hybrid, deletion, or tandem structure rather than one biologically equivalent biallelic rsID. | Use a validated CYP2D6 assay with star-allele, copy-number, hybrid/tandem, and phase resolution; report diplotype, activity score, assay coverage, and residual uncertainty. |
| *CYP2D6* | *68-*4 | Structural/CNV or complex star-allele representation | No function or likely no function | May contribute to poor metabolizer phenotype when present with another no-function allele; exact phenotype requires diplotype interpretation. | Codeine; tramadol; antidepressants; antipsychotics; tamoxifen; atomoxetine; ondansetron | CPIC: Guideline available; DPWG: Guideline available | 0.37% (0.06–2.05) | Moderate | Supplementary complex-variant evidence; not displayed as a separate main-text representative. | The reported unit is a star allele, haplotype, copy-number state, hybrid, deletion, or tandem structure rather than one biologically equivalent biallelic rsID. | Use a validated CYP2D6 assay with star-allele, copy-number, hybrid/tandem, and phase resolution; report diplotype, activity score, assay coverage, and residual uncertainty. |
| *CYP2D6* | *86 | Structural/CNV or complex star-allele representation | No function or likely no function | May contribute to poor metabolizer phenotype when present with another no-function allele; exact phenotype requires diplotype interpretation. | Codeine; tramadol; antidepressants; antipsychotics; tamoxifen; atomoxetine; ondansetron | CPIC: Guideline available; DPWG: Guideline available | 0.37% (0.06–2.05) | Moderate | Supplementary complex-variant evidence; not displayed as a separate main-text representative. | The reported unit is a star allele, haplotype, copy-number state, hybrid, deletion, or tandem structure rather than one biologically equivalent biallelic rsID. | Use a validated CYP2D6 assay with star-allele, copy-number, hybrid/tandem, and phase resolution; report diplotype, activity score, assay coverage, and residual uncertainty. |
| *CYP2D6* | *2 | Haplotype/star-allele representation | Normal function | Typically contributes to normal CYP2D6 activity, but phenotype depends on diplotype and copy number. | Codeine; tramadol; antidepressants; antipsychotics; tamoxifen; atomoxetine; ondansetron | CPIC: Guideline available; DPWG: Guideline available | 7.94% (5.20–11.94) | Potentially actionable | Supplementary complex-variant evidence; not displayed as a separate main-text representative. | The reported unit is a star allele, haplotype, copy-number state, hybrid, deletion, or tandem structure rather than one biologically equivalent biallelic rsID. | Use a validated CYP2D6 assay with star-allele, copy-number, hybrid/tandem, and phase resolution; report diplotype, activity score, assay coverage, and residual uncertainty. |
| *CYP2D6* | *2 | Structural/CNV or complex star-allele representation | Normal function | Typically contributes to normal CYP2D6 activity, but phenotype depends on diplotype and copy number. | Codeine; tramadol; antidepressants; antipsychotics; tamoxifen; atomoxetine; ondansetron | CPIC: Guideline available; DPWG: Guideline available | 7.72% (5.10–11.51) | Potentially actionable | Supplementary complex-variant evidence; not displayed as a separate main-text representative. | The reported unit is a star allele, haplotype, copy-number state, hybrid, deletion, or tandem structure rather than one biologically equivalent biallelic rsID. | Use a validated CYP2D6 assay with star-allele, copy-number, hybrid/tandem, and phase resolution; report diplotype, activity score, assay coverage, and residual uncertainty. |
| *CYP1B1* | Dup | Structural/CNV or complex star-allele representation | Copy-number gain / structural marker; clinical PGx function not established | No validated drug-response phenotype assignment for routine prescribing. | Not established | CPIC: No direct guideline; DPWG: No direct/unclear guideline | 0.97% (0.33–2.82) | Population evidence / exploratory | Supplementary complex-variant evidence; not displayed as a separate main-text representative. | The reported unit is a star allele, haplotype, copy-number state, hybrid, deletion, or tandem structure rather than one biologically equivalent biallelic rsID. | Validate copy-number/structural detection and state the detectable configurations; retain the source representation in reports. |
| *CYP2A6* | *4 | Structural/CNV or complex star-allele representation | No function / gene deletion | Reduced or absent CYP2A6 activity; may affect nicotine metabolism and selected CYP2A6 substrates, but prescribing actionability is not guideline-level. | Nicotine; tegafur and selected CYP2A6 substrates | CPIC: No direct guideline; DPWG: No direct/unclear guideline | 11.81% (7.50–18.09) | Population evidence / exploratory | Supplementary complex-variant evidence; not displayed as a separate main-text representative. | The reported unit is a star allele, haplotype, copy-number state, hybrid, deletion, or tandem structure rather than one biologically equivalent biallelic rsID. | Use an assay capable of deletion/CNV and star-allele discrimination; report which structural alleles are detectable. |
| *CYP2A6* | Del | Structural/CNV or complex star-allele representation | No function / gene deletion | Reduced or absent CYP2A6 activity; may affect nicotine metabolism and selected CYP2A6 substrates, but prescribing actionability is not guideline-level. | Nicotine; tegafur and selected CYP2A6 substrates | CPIC: No direct guideline; DPWG: No direct/unclear guideline | 11.04% (8.01–15.03) | Population evidence / exploratory | Supplementary complex-variant evidence; not displayed as a separate main-text representative. | The reported unit is a star allele, haplotype, copy-number state, hybrid, deletion, or tandem structure rather than one biologically equivalent biallelic rsID. | Use an assay capable of deletion/CNV and star-allele discrimination; report which structural alleles are detectable. |
| *CYP2C9* | Del | Structural/CNV or complex star-allele representation | Structural marker; function not established | No guideline-level phenotype assignment for routine prescribing. | Warfarin; phenytoin; NSAIDs | CPIC: No direct guideline; DPWG: No direct/unclear guideline | 0.97% (0.33–2.82) | Population evidence / exploratory | Supplementary complex-variant evidence; not displayed as a separate main-text representative. | The reported unit is a star allele, haplotype, copy-number state, hybrid, deletion, or tandem structure rather than one biologically equivalent biallelic rsID. | Validate copy-number/structural detection and state the detectable configurations; retain the source representation in reports. |
| *CYP2D6* | *21B | Haplotype/star-allele representation | Uncertain or not established | No guideline-level phenotype assignment is currently assigned in this curation file. | Not established | CPIC: No direct guideline; DPWG: No direct/unclear guideline | 0.40% (0.07–2.21) | Population evidence / exploratory | Supplementary complex-variant evidence; not displayed as a separate main-text representative. | The reported unit is a star allele, haplotype, copy-number state, hybrid, deletion, or tandem structure rather than one biologically equivalent biallelic rsID. | Use a validated CYP2D6 assay with star-allele, copy-number, hybrid/tandem, and phase resolution; report diplotype, activity score, assay coverage, and residual uncertainty. |
| *CYP2E1* | Del | Structural/CNV or complex star-allele representation | Uncertain or altered transcription/activity marker | CYP2E1 polymorphisms may affect enzyme expression or activity, but routine genotype-guided prescribing is not established. | Ethanol-related metabolism; anesthetics; selected xenobiotics | CPIC: No direct guideline; DPWG: No direct/unclear guideline | 0.32% (0.06–1.82) | Population evidence / exploratory | Supplementary complex-variant evidence; not displayed as a separate main-text representative. | The reported unit is a star allele, haplotype, copy-number state, hybrid, deletion, or tandem structure rather than one biologically equivalent biallelic rsID. | Validate copy-number/structural detection and state the detectable configurations; retain the source representation in reports. |
| *CYP2E1* | Dup | Structural/CNV or complex star-allele representation | Uncertain or altered transcription/activity marker | CYP2E1 polymorphisms may affect enzyme expression or activity, but routine genotype-guided prescribing is not established. | Ethanol-related metabolism; anesthetics; selected xenobiotics | CPIC: No direct guideline; DPWG: No direct/unclear guideline | 0.97% (0.33–2.82) | Population evidence / exploratory | Supplementary complex-variant evidence; not displayed as a separate main-text representative. | The reported unit is a star allele, haplotype, copy-number state, hybrid, deletion, or tandem structure rather than one biologically equivalent biallelic rsID. | Validate copy-number/structural detection and state the detectable configurations; retain the source representation in reports. |
| *CYP2J2* | *8 | Non-rsID representation | Uncertain function | No routine pharmacogenetic prescribing implication is established. | Not established | CPIC: No direct guideline; DPWG: No direct/unclear guideline | 0.00% (0.00–1.19) | Population evidence / exploratory | Supplementary complex-variant evidence; not displayed as a separate main-text representative. | No validated one-to-one biallelic rsID representation was available for direct 1000 Genomes comparison. | Retain the source nomenclature and avoid conversion to a single-rsID proxy without a validated one-to-one mapping. |
| *CYP2J2* | *9 | Non-rsID representation | Uncertain function | No routine pharmacogenetic prescribing implication is established. | Not established | CPIC: No direct guideline; DPWG: No direct/unclear guideline | 0.00% (0.00–1.19) | Population evidence / exploratory | Supplementary complex-variant evidence; not displayed as a separate main-text representative. | No validated one-to-one biallelic rsID representation was available for direct 1000 Genomes comparison. | Retain the source nomenclature and avoid conversion to a single-rsID proxy without a validated one-to-one mapping. |
| *CYP3A4* | *18 | Haplotype/star-allele representation | Altered function candidate | Reported CYP3A4 star allele; routine clinical dosing actionability is not established. | Tacrolimus; statins; calcium-channel blockers; many CYP3A4 substrates | CPIC: No direct guideline; DPWG: No direct/unclear guideline | 0.79% (0.22–2.83) | Population evidence / exploratory | Supplementary complex-variant evidence; not displayed as a separate main-text representative. | The reported unit is a star allele, haplotype, copy-number state, hybrid, deletion, or tandem structure rather than one biologically equivalent biallelic rsID. | Retain haplotype/star-allele nomenclature and document defining variants and phasing assumptions. |
| *HNF4A* | G36S | Non-rsID representation | Diabetes-related genetic marker; pharmacogenetic function not established | No routine drug-response phenotype assignment for this variant in the current PGx framework. | Not established | CPIC: No direct guideline; DPWG: No direct/unclear guideline | 3.60% (1.97–6.49) | Population evidence / exploratory | Supplementary complex-variant evidence; not displayed as a separate main-text representative. | No validated one-to-one biallelic rsID representation was available for direct 1000 Genomes comparison. | Retain the source nomenclature and avoid conversion to a single-rsID proxy without a validated one-to-one mapping. |
| *HNF4A* | G60D | Non-rsID representation | Diabetes-related genetic marker; pharmacogenetic function not established | No routine drug-response phenotype assignment for this variant in the current PGx framework. | Not established | CPIC: No direct guideline; DPWG: No direct/unclear guideline | 0.72% (0.20–2.58) | Population evidence / exploratory | Supplementary complex-variant evidence; not displayed as a separate main-text representative. | No validated one-to-one biallelic rsID representation was available for direct 1000 Genomes comparison. | Retain the source nomenclature and avoid conversion to a single-rsID proxy without a validated one-to-one mapping. |
| *NAT2* | *12 | Haplotype/star-allele representation | Normal or rapid-acetylator haplotype marker | May contribute to normal/rapid acetylator phenotype depending on haplotype context. | Isoniazid; hydralazine; sulfonamides; selected aromatic amines | CPIC: No direct guideline; DPWG: No direct/unclear guideline | 3.47% (1.49–7.87) | Population evidence / exploratory | Supplementary complex-variant evidence; not displayed as a separate main-text representative. | The reported unit is a star allele, haplotype, copy-number state, hybrid, deletion, or tandem structure rather than one biologically equivalent biallelic rsID. | Assign haplotypes/diplotypes using phased or validated inference; do not infer acetylator phenotype from one source label alone. |
| *NAT2* | *13 | Haplotype/star-allele representation | Normal or rapid-acetylator haplotype marker | May contribute to normal/rapid acetylator phenotype depending on haplotype context. | Isoniazid; hydralazine; sulfonamides; selected aromatic amines | CPIC: No direct guideline; DPWG: No direct/unclear guideline | 30.56% (23.62–38.50) | Population evidence / exploratory | Supplementary complex-variant evidence; not displayed as a separate main-text representative. | The reported unit is a star allele, haplotype, copy-number state, hybrid, deletion, or tandem structure rather than one biologically equivalent biallelic rsID. | Assign haplotypes/diplotypes using phased or validated inference; do not infer acetylator phenotype from one source label alone. |
| *NAT2* | *19 | Haplotype/star-allele representation | Uncertain function haplotype marker | NAT2 phenotype effect requires haplotype-level assignment; do not interpret as a standalone marker. | Isoniazid; hydralazine; sulfonamides; selected aromatic amines | CPIC: No direct guideline; DPWG: No direct/unclear guideline | 1.39% (0.38–4.92) | Population evidence / exploratory | Supplementary complex-variant evidence; not displayed as a separate main-text representative. | The reported unit is a star allele, haplotype, copy-number state, hybrid, deletion, or tandem structure rather than one biologically equivalent biallelic rsID. | Assign haplotypes/diplotypes using phased or validated inference; do not infer acetylator phenotype from one source label alone. |
| *NAT2* | *5 | Haplotype/star-allele representation | Decreased function / slow-acetylator allele marker | Contributes to NAT2 slow-acetylator phenotype depending on haplotype; may influence isoniazid exposure and hepatotoxicity risk. | Isoniazid; hydralazine; sulfonamides; selected aromatic amines | CPIC: No direct guideline; DPWG: No direct/unclear guideline | 2.78% (1.09–6.92) | Population evidence / exploratory | Supplementary complex-variant evidence; not displayed as a separate main-text representative. | The reported unit is a star allele, haplotype, copy-number state, hybrid, deletion, or tandem structure rather than one biologically equivalent biallelic rsID. | Assign haplotypes/diplotypes using phased or validated inference; do not infer acetylator phenotype from one source label alone. |
| *NAT2* | *6A | Haplotype/star-allele representation | Decreased function / slow-acetylator allele marker | Contributes to NAT2 slow-acetylator phenotype depending on haplotype; may influence isoniazid exposure and hepatotoxicity risk. | Isoniazid; hydralazine; sulfonamides; selected aromatic amines | CPIC: No direct guideline; DPWG: No direct/unclear guideline | 17.36% (12.04–24.37) | Population evidence / exploratory | Supplementary complex-variant evidence; not displayed as a separate main-text representative. | The reported unit is a star allele, haplotype, copy-number state, hybrid, deletion, or tandem structure rather than one biologically equivalent biallelic rsID. | Assign haplotypes/diplotypes using phased or validated inference; do not infer acetylator phenotype from one source label alone. |
| *NAT2* | *7 | Haplotype/star-allele representation | Decreased function / slow-acetylator allele marker | Contributes to NAT2 slow-acetylator phenotype depending on haplotype; may influence isoniazid exposure and hepatotoxicity risk. | Isoniazid; hydralazine; sulfonamides; selected aromatic amines | CPIC: No direct guideline; DPWG: No direct/unclear guideline | 0.00% (0.00–2.60) | Population evidence / exploratory | Supplementary complex-variant evidence; not displayed as a separate main-text representative. | The reported unit is a star allele, haplotype, copy-number state, hybrid, deletion, or tandem structure rather than one biologically equivalent biallelic rsID. | Assign haplotypes/diplotypes using phased or validated inference; do not infer acetylator phenotype from one source label alone. |

Abbreviations: CNV, copy-number variant; HLA, human leukocyte antigen; PGx, pharmacogenomics; rsID, Reference SNP cluster identifier.
