# Supplementary material for: Vietnamese pharmacogenomic variation in global context: a systematic review and meta-analysis for clinical implementation
Source: Front Pharmacol. 2026 Sep 3;17:1921144. doi: 10.3389/fphar.2026.1921144 (PMC13582518; doi:10.3389/fphar.2026.1921144)
Supplement: Supplementary file 9 [file Table9.docx]

**Supplementary Table S9. Full allele-orientation audit and pairwise Vietnamese-versus-1000 Genomes comparison results**

The principal complete-case matrix contains 20 orientation-validated variants. Vietnamese frequencies in S9A–S9B are allele-count estimates from non-overlapping analysis rows and may differ slightly from the random-effects pooled estimates in Supplementary Table S3. The East Asian superpopulation includes the Kinh in Ho Chi Minh City (KHV) sample, so Vietnamese–EAS comparisons are not fully independent.

Pairwise odds ratios compare the harmonized effect allele in Vietnam with each 1000 Genomes Phase 3 superpopulation. OR >1 indicates a higher effect-allele frequency in Vietnam. Difference is Vietnam minus comparator in percentage points. A Haldane–Anscombe correction of 0.5 was used when a 2×2 cell was zero. Benjamini–Hochberg correction was applied across all 100 variant–population comparisons.

**Table S9A. Allele-orientation and count audit for the 20 principal global-comparison variants**

| **Gene/variant** | **rsID** | **Reference allele** | **Effect allele** | **VNM effect count/AN** | **VNM, %** | **AFR effect count/AN** | **AFR, %** | **AMR effect count/AN** | **AMR, %** | **EAS effect count/AN** | **EAS, %** | **EUR effect count/AN** | **EUR, %** | **SAS effect count/AN** | **SAS, %** | **Vietnamese source study ID(s)** | **Orientation/harmonization note** |
| --- | --- | --- | --- | --- | --- | --- | --- | --- | --- | --- | --- | --- | --- | --- | --- | --- | --- |
| CYP2C19*2 | rs4244285 | G | A | 1455/5228 | 27.83 | 225/1322 | 17.02 | 73/694 | 10.52 | 314/1008 | 31.15 | 146/1006 | 14.51 | 350/978 | 35.79 | S0007, S0014, S0018, S0021, S0022, S0025, S0026, S0028, S0030, S0031, S0032, S0045, S0048, S0054, S0060, S0080, S0088, S0098, S0104 | Effect-allele orientation matched across Vietnamese and 1000 Genomes data. |
| CYP2C19*3 | rs4986893 | G | A | 321/5558 | 5.78 | 3/1322 | 0.23 | 0/694 | 0.00 | 56/1008 | 5.56 | 0/1006 | 0.00 | 12/978 | 1.23 | S0007, S0014, S0018, S0021, S0022, S0025, S0026, S0028, S0030, S0031, S0032, S0045, S0048, S0054, S0060, S0061, S0080, S0088, S0098, S0104 | Effect-allele orientation matched across Vietnamese and 1000 Genomes data. |
| CYP2C19*17 | rs12248560 | C | T | 11/844 | 1.30 | 311/1322 | 23.52 | 83/694 | 11.96 | 15/1008 | 1.49 | 225/1006 | 22.37 | 133/978 | 13.60 | S0018, S0060, S0104 | Effect-allele orientation matched across Vietnamese and 1000 Genomes data. |
| CYP2C9*2 | rs1799853 | C | T | 0/698 | 0.00 | 11/1322 | 0.83 | 69/694 | 9.94 | 1/1008 | 0.10 | 125/1006 | 12.43 | 34/978 | 3.48 | S0004, S0011, S0033 | Effect-allele orientation matched across Vietnamese and 1000 Genomes data. |
| CYP2C9*3 | rs1057910 | A | C | 44/1476 | 2.98 | 3/1322 | 0.23 | 3/694 | 0.43 | 34/1008 | 3.37 | 73/1006 | 7.26 | 107/978 | 10.94 | S0004, S0011, S0012, S0025, S0033, S0043 | Effect-allele orientation matched across Vietnamese and 1000 Genomes data. |
| CYP3A5*3 | rs776746 | A | G | 696/1086 | 64.09 | 238/1322 | 18.00 | 553/694 | 79.68 | 719/1008 | 71.33 | 949/1006 | 94.33 | 653/978 | 66.77 | S0008, S0014, S0033, S0036, S0044, S0060, S0089 | Effect-allele orientation matched across Vietnamese and 1000 Genomes data. |
| SLCO1B1 c.521T>C | rs4149056 | T | C | 53/408 | 12.99 | 19/1322 | 1.44 | 93/694 | 13.40 | 124/1008 | 12.30 | 162/1006 | 16.10 | 42/978 | 4.29 | S0025, S0073 | Used only rows explicitly reporting c.521T>C; source-labelled *5 and *15 representations were not double-counted. |
| SLCO1B1 c.388A>G | rs2306283 | A | G | 310/408 | 75.98 | 1081/1322 | 81.77 | 328/694 | 47.26 | 768/1008 | 76.19 | 405/1006 | 40.26 | 535/978 | 54.70 | S0025, S0073 | Combined source labels “c.388A>G” and “rs2306283” with identical A>G orientation. |
| NAT2*5 | rs1801280 | T | C | 54/914 | 5.91 | 386/1322 | 29.20 | 251/694 | 36.17 | 38/1008 | 3.77 | 452/1006 | 44.93 | 339/978 | 34.66 | S0009, S0025, S0067, S0092 | Effect-allele orientation matched across Vietnamese and 1000 Genomes data. |
| NAT2*7 | rs1799931 | G | A | 162/914 | 17.72 | 38/1322 | 2.87 | 78/694 | 11.24 | 181/1008 | 17.96 | 23/1006 | 2.29 | 67/978 | 6.85 | S0009, S0025, S0067, S0092 | Effect-allele orientation matched across Vietnamese and 1000 Genomes data. |
| UGT1A1*6 | rs4148323 | G | A | 31/300 | 10.33 | 1/1322 | 0.08 | 8/694 | 1.15 | 139/1008 | 13.79 | 7/1006 | 0.70 | 17/978 | 1.74 | S0025, S0049 | Effect-allele orientation matched across Vietnamese and 1000 Genomes data. |
| ABCB1 3435C>T | rs1045642 | C | T | 58/144 | 40.28 | 1124/1322 | 85.02 | 397/694 | 57.20 | 607/1008 | 60.22 | 485/1006 | 48.21 | 416/978 | 42.54 | S0014 | Effect-allele orientation matched across Vietnamese and 1000 Genomes data. |
| VKORC1 -1639G>A | rs9923231 | G | A | 362/398 | 90.95 | 71/1322 | 5.37 | 285/694 | 41.07 | 892/1008 | 88.49 | 390/1006 | 38.77 | 142/978 | 14.52 | S0043, S0066 | Effect-allele orientation matched across Vietnamese and 1000 Genomes data. |
| VKORC1 497T>G | rs2884737 | T | G | 1/98 | 1.02 | 12/1322 | 0.91 | 122/694 | 17.58 | 1/1008 | 0.10 | 257/1006 | 25.55 | 66/978 | 6.75 | S0066 | Effect-allele orientation matched across Vietnamese and 1000 Genomes data. |
| CYP2B6 516G>T | rs3745274 | G | T | 85/344 | 24.71 | 494/1322 | 37.37 | 259/694 | 37.32 | 217/1008 | 21.53 | 237/1006 | 23.56 | 373/978 | 38.14 | S0014, S0025 | Combined source labels “516G>T” and “rs3745274” with identical G>T orientation. |
| VKORC1 1173C>T | rs9934438 | C | T | 340/398 | 85.43 | 71/1322 | 5.37 | 286/694 | 41.21 | 892/1008 | 88.49 | 390/1006 | 38.77 | 142/978 | 14.52 | S0043, S0066 | Combined source labels “-1173C>T” and “1173C>T” with identical C>T orientation. |
| VKORC1 1542G>C | rs8050894 | G | C | 84/98 | 85.71 | 338/1322 | 25.57 | 307/694 | 44.24 | 892/1008 | 88.49 | 402/1006 | 39.96 | 145/978 | 14.83 | S0066 | Effect-allele orientation matched across Vietnamese and 1000 Genomes data. |
| VKORC1 3730G>A | rs7294 | G | A | 14/98 | 14.29 | 600/1322 | 45.39 | 279/694 | 40.20 | 113/1008 | 11.21 | 368/1006 | 36.58 | 742/978 | 75.87 | S0066 | Effect-allele orientation matched across Vietnamese and 1000 Genomes data. |
| NAT2 rs1208 | rs1208 | G | A | 182/200 | 91.00 | 800/1322 | 60.51 | 435/694 | 62.68 | 968/1008 | 96.03 | 565/1006 | 56.16 | 623/978 | 63.70 | S0025 | Effect-allele orientation matched across Vietnamese and 1000 Genomes data. |
| VKORC1 2255C>T | rs2359612 | C | T | 84/98 | 85.71 | 1085/1322 | 82.07 | 399/694 | 57.49 | 892/1008 | 88.49 | 616/1006 | 61.23 | 836/978 | 85.48 | S0066 | T-allele orientation independently verified; EAS includes KHV, so the comparison is not fully independent. |

Population abbreviations: AFR, African; AMR, admixed American; EAS, East Asian; EUR, European; SAS, South Asian; VNM, Vietnamese. AN, allele number.

**Table S9B. Pairwise Vietnamese-versus-1000 Genomes comparisons for the 20 principal variants**

| **Gene/variant** | **rsID** | **Effect allele** | **Comparator** | **VNM effect count/AN** | **Comparator effect count/AN** | **VNM, %** | **Comparator, %** | **Difference, pp** | **Odds ratio** | **95% CI** | **Raw p value** | **FDR-adjusted p value** | **FDR <0.05** | **Haldane correction** |
| --- | --- | --- | --- | --- | --- | --- | --- | --- | --- | --- | --- | --- | --- | --- |
| CYP2C19*2 | rs4244285 | A | AFR | 1455/5228 | 225/1322 | 27.83 | 17.02 | +10.81 | 1.880 | 1.609–2.197 | 1.88e-15 | 6.47e-15 | Yes | No |
| CYP2C19*2 | rs4244285 | A | AMR | 1455/5228 | 73/694 | 27.83 | 10.52 | +17.31 | 3.281 | 2.555–4.212 | 1.21e-20 | 5.02e-20 | Yes | No |
| CYP2C19*2 | rs4244285 | A | EAS | 1455/5228 | 314/1008 | 27.83 | 31.15 | -3.32 | 0.852 | 0.736–0.987 | 0.0324 | 0.0438 | Yes | No |
| CYP2C19*2 | rs4244285 | A | EUR | 1455/5228 | 146/1006 | 27.83 | 14.51 | +13.32 | 2.272 | 1.887–2.735 | 4.49e-18 | 1.73e-17 | Yes | No |
| CYP2C19*2 | rs4244285 | A | SAS | 1455/5228 | 350/978 | 27.83 | 35.79 | -7.96 | 0.692 | 0.599–0.799 | 5.43e-07 | 1.13e-06 | Yes | No |
| CYP2C19*3 | rs4986893 | A | AFR | 321/5558 | 3/1322 | 5.78 | 0.23 | +5.55 | 26.949 | 8.632–84.136 | 1.42e-08 | 3.23e-08 | Yes | No |
| CYP2C19*3 | rs4986893 | A | AMR | 321/5558 | 0/694 | 5.78 | 0.00 | +5.78 | 85.263 | 5.315–1367.691 | 0.0017 | 0.0025 | Yes | Yes |
| CYP2C19*3 | rs4986893 | A | EAS | 321/5558 | 56/1008 | 5.78 | 5.56 | +0.22 | 1.042 | 0.778–1.396 | 0.7825 | 0.8237 | No | No |
| CYP2C19*3 | rs4986893 | A | EUR | 321/5558 | 0/1006 | 5.78 | 0.00 | +5.78 | 123.566 | 7.706–1981.506 | 6.68e-04 | 0.0010 | Yes | Yes |
| CYP2C19*3 | rs4986893 | A | SAS | 321/5558 | 12/978 | 5.78 | 1.23 | +4.55 | 4.934 | 2.762–8.816 | 7.02e-08 | 1.49e-07 | Yes | No |
| CYP2C19*17 | rs12248560 | T | AFR | 11/844 | 311/1322 | 1.30 | 23.52 | -22.22 | 0.043 | 0.023–0.079 | 3.51e-24 | 1.85e-23 | Yes | No |
| CYP2C19*17 | rs12248560 | T | AMR | 11/844 | 83/694 | 1.30 | 11.96 | -10.66 | 0.097 | 0.051–0.184 | 7.71e-13 | 2.20e-12 | Yes | No |
| CYP2C19*17 | rs12248560 | T | EAS | 11/844 | 15/1008 | 1.30 | 1.49 | -0.18 | 0.874 | 0.399–1.914 | 0.7366 | 0.7836 | No | No |
| CYP2C19*17 | rs12248560 | T | EUR | 11/844 | 225/1006 | 1.30 | 22.37 | -21.06 | 0.046 | 0.025–0.085 | 6.49e-23 | 3.24e-22 | Yes | No |
| CYP2C19*17 | rs12248560 | T | SAS | 11/844 | 133/978 | 1.30 | 13.60 | -12.30 | 0.084 | 0.045–0.156 | 5.95e-15 | 1.98e-14 | Yes | No |
| CYP2C9*2 | rs1799853 | T | AFR | 0/698 | 11/1322 | 0.00 | 0.83 | -0.83 | 0.082 | 0.005–1.387 | 0.0830 | 0.1078 | No | Yes |
| CYP2C9*2 | rs1799853 | T | AMR | 0/698 | 69/694 | 0.00 | 9.94 | -9.94 | 0.006 | 0.000–0.104 | 3.83e-04 | 6.07e-04 | Yes | Yes |
| CYP2C9*2 | rs1799853 | T | EAS | 0/698 | 1/1008 | 0.00 | 0.10 | -0.10 | 0.481 | 0.020–11.820 | 0.6540 | 0.7187 | No | Yes |
| CYP2C9*2 | rs1799853 | T | EUR | 0/698 | 125/1006 | 0.00 | 12.43 | -12.43 | 0.005 | 0.000–0.081 | 1.89e-04 | 3.16e-04 | Yes | Yes |
| CYP2C9*2 | rs1799853 | T | SAS | 0/698 | 34/978 | 0.00 | 3.48 | -3.48 | 0.020 | 0.001–0.320 | 0.0058 | 0.0082 | Yes | Yes |
| CYP2C9*3 | rs1057910 | C | AFR | 44/1476 | 3/1322 | 2.98 | 0.23 | +2.75 | 13.509 | 4.185–43.611 | 1.34e-05 | 2.36e-05 | Yes | No |
| CYP2C9*3 | rs1057910 | C | AMR | 44/1476 | 3/694 | 2.98 | 0.43 | +2.55 | 7.077 | 2.190–22.873 | 0.0011 | 0.0016 | Yes | No |
| CYP2C9*3 | rs1057910 | C | EAS | 44/1476 | 34/1008 | 2.98 | 3.37 | -0.39 | 0.880 | 0.559–1.387 | 0.5825 | 0.6545 | No | No |
| CYP2C9*3 | rs1057910 | C | EUR | 44/1476 | 73/1006 | 2.98 | 7.26 | -4.28 | 0.393 | 0.268–0.576 | 1.73e-06 | 3.39e-06 | Yes | No |
| CYP2C9*3 | rs1057910 | C | SAS | 44/1476 | 107/978 | 2.98 | 10.94 | -7.96 | 0.250 | 0.174–0.359 | 5.29e-14 | 1.71e-13 | Yes | No |
| CYP3A5*3 | rs776746 | G | AFR | 696/1086 | 238/1322 | 64.09 | 18.00 | +46.09 | 8.128 | 6.740–9.802 | 1.20e-106 | 4.02e-105 | Yes | No |
| CYP3A5*3 | rs776746 | G | AMR | 696/1086 | 553/694 | 64.09 | 79.68 | -15.59 | 0.455 | 0.364–0.568 | 4.14e-12 | 1.12e-11 | Yes | No |
| CYP3A5*3 | rs776746 | G | EAS | 696/1086 | 719/1008 | 64.09 | 71.33 | -7.24 | 0.717 | 0.597–0.863 | 4.14e-04 | 6.46e-04 | Yes | No |
| CYP3A5*3 | rs776746 | G | EUR | 696/1086 | 949/1006 | 64.09 | 94.33 | -30.25 | 0.107 | 0.080–0.144 | 6.44e-50 | 9.19e-49 | Yes | No |
| CYP3A5*3 | rs776746 | G | SAS | 696/1086 | 653/978 | 64.09 | 66.77 | -2.68 | 0.888 | 0.741–1.065 | 0.2014 | 0.2426 | No | No |
| SLCO1B1 c.521T>C | rs4149056 | C | AFR | 53/408 | 19/1322 | 12.99 | 1.44 | +11.55 | 10.239 | 5.984–17.518 | 2.08e-17 | 7.71e-17 | Yes | No |
| SLCO1B1 c.521T>C | rs4149056 | C | AMR | 53/408 | 93/694 | 12.99 | 13.40 | -0.41 | 0.965 | 0.672–1.386 | 0.8462 | 0.8814 | No | No |
| SLCO1B1 c.521T>C | rs4149056 | C | EAS | 53/408 | 124/1008 | 12.99 | 12.30 | +0.69 | 1.064 | 0.754–1.502 | 0.7227 | 0.7771 | No | No |
| SLCO1B1 c.521T>C | rs4149056 | C | EUR | 53/408 | 162/1006 | 12.99 | 16.10 | -3.11 | 0.778 | 0.557–1.086 | 0.1404 | 0.1733 | No | No |
| SLCO1B1 c.521T>C | rs4149056 | C | SAS | 53/408 | 42/978 | 12.99 | 4.29 | +8.70 | 3.327 | 2.180–5.079 | 2.53e-08 | 5.63e-08 | Yes | No |
| SLCO1B1 c.388A>G | rs2306283 | G | AFR | 310/408 | 1081/1322 | 75.98 | 81.77 | -5.79 | 0.705 | 0.540–0.921 | 0.0102 | 0.0142 | Yes | No |
| SLCO1B1 c.388A>G | rs2306283 | G | AMR | 310/408 | 328/694 | 75.98 | 47.26 | +28.72 | 3.530 | 2.690–4.632 | 9.07e-20 | 3.63e-19 | Yes | No |
| SLCO1B1 c.388A>G | rs2306283 | G | EAS | 310/408 | 768/1008 | 75.98 | 76.19 | -0.21 | 0.989 | 0.755–1.294 | 0.9331 | 0.9425 | No | No |
| SLCO1B1 c.388A>G | rs2306283 | G | EUR | 310/408 | 405/1006 | 75.98 | 40.26 | +35.72 | 4.694 | 3.620–6.086 | 1.86e-31 | 1.16e-30 | Yes | No |
| SLCO1B1 c.388A>G | rs2306283 | G | SAS | 310/408 | 535/978 | 75.98 | 54.70 | +21.28 | 2.619 | 2.020–3.396 | 3.67e-13 | 1.08e-12 | Yes | No |
| NAT2*5 | rs1801280 | C | AFR | 54/914 | 386/1322 | 5.91 | 29.20 | -23.29 | 0.152 | 0.113–0.205 | 7.08e-35 | 5.06e-34 | Yes | No |
| NAT2*5 | rs1801280 | C | AMR | 54/914 | 251/694 | 5.91 | 36.17 | -30.26 | 0.111 | 0.081–0.152 | 1.69e-42 | 1.41e-41 | Yes | No |
| NAT2*5 | rs1801280 | C | EAS | 54/914 | 38/1008 | 5.91 | 3.77 | +2.14 | 1.603 | 1.048–2.452 | 0.0296 | 0.0405 | Yes | No |
| NAT2*5 | rs1801280 | C | EUR | 54/914 | 452/1006 | 5.91 | 44.93 | -39.02 | 0.077 | 0.057–0.104 | 2.63e-62 | 4.38e-61 | Yes | No |
| NAT2*5 | rs1801280 | C | SAS | 54/914 | 339/978 | 5.91 | 34.66 | -28.75 | 0.118 | 0.087–0.161 | 7.79e-43 | 7.08e-42 | Yes | No |
| NAT2*7 | rs1799931 | A | AFR | 162/914 | 38/1322 | 17.72 | 2.87 | +14.85 | 7.279 | 5.055–10.481 | 1.38e-26 | 7.65e-26 | Yes | No |
| NAT2*7 | rs1799931 | A | AMR | 162/914 | 78/694 | 17.72 | 11.24 | +6.49 | 1.701 | 1.273–2.274 | 3.34e-04 | 5.39e-04 | Yes | No |
| NAT2*7 | rs1799931 | A | EAS | 162/914 | 181/1008 | 17.72 | 17.96 | -0.23 | 0.984 | 0.779–1.244 | 0.8944 | 0.9221 | No | No |
| NAT2*7 | rs1799931 | A | EUR | 162/914 | 23/1006 | 17.72 | 2.29 | +15.44 | 9.207 | 5.889–14.396 | 2.13e-22 | 9.68e-22 | Yes | No |
| NAT2*7 | rs1799931 | A | SAS | 162/914 | 67/978 | 17.72 | 6.85 | +10.87 | 2.929 | 2.169–3.956 | 2.44e-12 | 6.77e-12 | Yes | No |
| UGT1A1*6 | rs4148323 | A | AFR | 31/300 | 1/1322 | 10.33 | 0.08 | +10.26 | 152.234 | 20.692–1120.011 | 7.99e-07 | 1.60e-06 | Yes | No |
| UGT1A1*6 | rs4148323 | A | AMR | 31/300 | 8/694 | 10.33 | 1.15 | +9.18 | 9.882 | 4.485–21.773 | 1.32e-08 | 3.06e-08 | Yes | No |
| UGT1A1*6 | rs4148323 | A | EAS | 31/300 | 139/1008 | 10.33 | 13.79 | -3.46 | 0.720 | 0.477–1.088 | 0.1194 | 0.1492 | No | No |
| UGT1A1*6 | rs4148323 | A | EUR | 31/300 | 7/1006 | 10.33 | 0.70 | +9.64 | 16.447 | 7.163–37.762 | 4.03e-11 | 9.83e-11 | Yes | No |
| UGT1A1*6 | rs4148323 | A | SAS | 31/300 | 17/978 | 10.33 | 1.74 | +8.60 | 6.515 | 3.551–11.951 | 1.42e-09 | 3.37e-09 | Yes | No |
| ABCB1 3435C>T | rs1045642 | T | AFR | 58/144 | 1124/1322 | 40.28 | 85.02 | -44.74 | 0.119 | 0.082–0.171 | 3.40e-30 | 2.00e-29 | Yes | No |
| ABCB1 3435C>T | rs1045642 | T | AMR | 58/144 | 397/694 | 40.28 | 57.20 | -16.93 | 0.505 | 0.350–0.727 | 2.43e-04 | 3.98e-04 | Yes | No |
| ABCB1 3435C>T | rs1045642 | T | EAS | 58/144 | 607/1008 | 40.28 | 60.22 | -19.94 | 0.446 | 0.312–0.636 | 8.60e-06 | 1.56e-05 | Yes | No |
| ABCB1 3435C>T | rs1045642 | T | EUR | 58/144 | 485/1006 | 40.28 | 48.21 | -7.93 | 0.724 | 0.508–1.033 | 0.0754 | 0.0992 | No | No |
| ABCB1 3435C>T | rs1045642 | T | SAS | 58/144 | 416/978 | 40.28 | 42.54 | -2.26 | 0.911 | 0.638–1.301 | 0.6086 | 0.6763 | No | No |
| VKORC1 -1639G>A | rs9923231 | A | AFR | 362/398 | 71/1322 | 90.95 | 5.37 | +85.58 | 177.176 | 116.677–269.045 | 2.43e-130 | 1.22e-128 | Yes | No |
| VKORC1 -1639G>A | rs9923231 | A | AMR | 362/398 | 285/694 | 90.95 | 41.07 | +49.89 | 14.431 | 9.924–20.984 | 2.27e-44 | 2.27e-43 | Yes | No |
| VKORC1 -1639G>A | rs9923231 | A | EAS | 362/398 | 892/1008 | 90.95 | 88.49 | +2.46 | 1.308 | 0.882–1.938 | 0.1814 | 0.2212 | No | No |
| VKORC1 -1639G>A | rs9923231 | A | EUR | 362/398 | 390/1006 | 90.95 | 38.77 | +52.19 | 15.883 | 11.023–22.885 | 8.25e-50 | 1.03e-48 | Yes | No |
| VKORC1 -1639G>A | rs9923231 | A | SAS | 362/398 | 142/978 | 90.95 | 14.52 | +76.44 | 59.200 | 40.244–87.086 | 2.13e-95 | 4.26e-94 | Yes | No |
| VKORC1 497T>G | rs2884737 | G | AFR | 1/98 | 12/1322 | 1.02 | 0.91 | +0.11 | 1.125 | 0.145–8.746 | 0.9101 | 0.9286 | No | No |
| VKORC1 497T>G | rs2884737 | G | AMR | 1/98 | 122/694 | 1.02 | 17.58 | -16.56 | 0.048 | 0.007–0.350 | 0.0027 | 0.0039 | Yes | No |
| VKORC1 497T>G | rs2884737 | G | EAS | 1/98 | 1/1008 | 1.02 | 0.10 | +0.92 | 10.381 | 0.644–167.285 | 0.0989 | 0.1269 | No | No |
| VKORC1 497T>G | rs2884737 | G | EUR | 1/98 | 257/1006 | 1.02 | 25.55 | -24.53 | 0.030 | 0.004–0.217 | 5.05e-04 | 7.77e-04 | Yes | No |
| VKORC1 497T>G | rs2884737 | G | SAS | 1/98 | 66/978 | 1.02 | 6.75 | -5.73 | 0.142 | 0.020–1.038 | 0.0544 | 0.0726 | No | No |
| CYP2B6 516G>T | rs3745274 | T | AFR | 85/344 | 494/1322 | 24.71 | 37.37 | -12.66 | 0.550 | 0.420–0.720 | 1.35e-05 | 2.36e-05 | Yes | No |
| CYP2B6 516G>T | rs3745274 | T | AMR | 85/344 | 259/694 | 24.71 | 37.32 | -12.61 | 0.551 | 0.413–0.736 | 5.45e-05 | 9.23e-05 | Yes | No |
| CYP2B6 516G>T | rs3745274 | T | EAS | 85/344 | 217/1008 | 24.71 | 21.53 | +3.18 | 1.196 | 0.897–1.595 | 0.2216 | 0.2638 | No | No |
| CYP2B6 516G>T | rs3745274 | T | EUR | 85/344 | 237/1006 | 24.71 | 23.56 | +1.15 | 1.065 | 0.801–1.416 | 0.6656 | 0.7234 | No | No |
| CYP2B6 516G>T | rs3745274 | T | SAS | 85/344 | 373/978 | 24.71 | 38.14 | -13.43 | 0.532 | 0.404–0.702 | 8.08e-06 | 1.50e-05 | Yes | No |
| VKORC1 1173C>T | rs9934438 | T | AFR | 340/398 | 71/1322 | 85.43 | 5.37 | +80.06 | 103.288 | 71.557–149.090 | 2.13e-135 | 2.13e-133 | Yes | No |
| VKORC1 1173C>T | rs9934438 | T | AMR | 340/398 | 286/694 | 85.43 | 41.21 | +44.22 | 8.363 | 6.092–11.480 | 1.99e-39 | 1.53e-38 | Yes | No |
| VKORC1 1173C>T | rs9934438 | T | EAS | 340/398 | 892/1008 | 85.43 | 88.49 | -3.06 | 0.762 | 0.543–1.070 | 0.1167 | 0.1477 | No | No |
| VKORC1 1173C>T | rs9934438 | T | EUR | 340/398 | 390/1006 | 85.43 | 38.77 | +46.66 | 9.259 | 6.818–12.573 | 4.07e-46 | 4.52e-45 | Yes | No |
| VKORC1 1173C>T | rs9934438 | T | SAS | 340/398 | 142/978 | 85.43 | 14.52 | +70.91 | 34.512 | 24.801–48.025 | 5.78e-98 | 1.44e-96 | Yes | No |
| VKORC1 1542G>C | rs8050894 | C | AFR | 84/98 | 338/1322 | 85.71 | 25.57 | +60.15 | 17.467 | 9.788–31.171 | 3.66e-22 | 1.59e-21 | Yes | No |
| VKORC1 1542G>C | rs8050894 | C | AMR | 84/98 | 307/694 | 85.71 | 44.24 | +41.48 | 7.564 | 4.212–13.580 | 1.24e-11 | 3.10e-11 | Yes | No |
| VKORC1 1542G>C | rs8050894 | C | EAS | 84/98 | 892/1008 | 85.71 | 88.49 | -2.78 | 0.780 | 0.429–1.419 | 0.4161 | 0.4728 | No | No |
| VKORC1 1542G>C | rs8050894 | C | EUR | 84/98 | 402/1006 | 85.71 | 39.96 | +45.75 | 9.015 | 5.049–16.096 | 1.05e-13 | 3.18e-13 | Yes | No |
| VKORC1 1542G>C | rs8050894 | C | SAS | 84/98 | 145/978 | 85.71 | 14.83 | +70.89 | 34.469 | 19.056–62.347 | 1.17e-31 | 7.78e-31 | Yes | No |
| VKORC1 3730G>A | rs7294 | A | AFR | 14/98 | 600/1322 | 14.29 | 45.39 | -31.10 | 0.201 | 0.113–0.357 | 4.59e-08 | 9.98e-08 | Yes | No |
| VKORC1 3730G>A | rs7294 | A | AMR | 14/98 | 279/694 | 14.29 | 40.20 | -25.92 | 0.248 | 0.138–0.445 | 3.06e-06 | 5.89e-06 | Yes | No |
| VKORC1 3730G>A | rs7294 | A | EAS | 14/98 | 113/1008 | 14.29 | 11.21 | +3.08 | 1.320 | 0.725–2.402 | 0.3633 | 0.4225 | No | No |
| VKORC1 3730G>A | rs7294 | A | EUR | 14/98 | 368/1006 | 14.29 | 36.58 | -22.29 | 0.289 | 0.162–0.516 | 2.74e-05 | 4.72e-05 | Yes | No |
| VKORC1 3730G>A | rs7294 | A | SAS | 14/98 | 742/978 | 14.29 | 75.87 | -61.58 | 0.053 | 0.030–0.095 | 6.83e-23 | 3.25e-22 | Yes | No |
| NAT2 rs1208 | rs1208 | A | AFR | 182/200 | 800/1322 | 91.00 | 60.51 | +30.49 | 6.598 | 4.015–10.841 | 9.68e-14 | 3.02e-13 | Yes | No |
| NAT2 rs1208 | rs1208 | A | AMR | 182/200 | 435/694 | 91.00 | 62.68 | +28.32 | 6.020 | 3.622–10.007 | 4.38e-12 | 1.15e-11 | Yes | No |
| NAT2 rs1208 | rs1208 | A | EAS | 182/200 | 968/1008 | 91.00 | 96.03 | -5.03 | 0.418 | 0.234–0.745 | 0.0031 | 0.0044 | Yes | No |
| NAT2 rs1208 | rs1208 | A | EUR | 182/200 | 565/1006 | 91.00 | 56.16 | +34.84 | 7.892 | 4.787–13.012 | 5.61e-16 | 2.00e-15 | Yes | No |
| NAT2 rs1208 | rs1208 | A | SAS | 182/200 | 623/978 | 91.00 | 63.70 | +27.30 | 5.762 | 3.489–9.514 | 7.70e-12 | 1.97e-11 | Yes | No |
| VKORC1 2255C>T | rs2359612 | T | AFR | 84/98 | 1085/1322 | 85.71 | 82.07 | +3.64 | 1.311 | 0.732–2.348 | 0.3632 | 0.4225 | No | No |
| VKORC1 2255C>T | rs2359612 | T | AMR | 84/98 | 399/694 | 85.71 | 57.49 | +28.22 | 4.436 | 2.470–7.967 | 6.12e-07 | 1.25e-06 | Yes | No |
| VKORC1 2255C>T | rs2359612 | T | EAS | 84/98 | 892/1008 | 85.71 | 88.49 | -2.78 | 0.780 | 0.429–1.419 | 0.4161 | 0.4728 | No | No |
| VKORC1 2255C>T | rs2359612 | T | EUR | 84/98 | 616/1006 | 85.71 | 61.23 | +24.48 | 3.799 | 2.127–6.784 | 6.44e-06 | 1.21e-05 | Yes | No |
| VKORC1 2255C>T | rs2359612 | T | SAS | 84/98 | 836/978 | 85.71 | 85.48 | +0.23 | 1.019 | 0.563–1.844 | 0.9500 | 0.9500 | No | No |

FDR, false discovery rate; OR, odds ratio; pp, percentage points. The 95% confidence interval is for the odds ratio. Raw and FDR-adjusted p values are two-sided.

**Table S9C. Global-comparison eligibility and representation status for all 108 source-level variant representations**

| **Gene** | **Allele/variant representation** | **rsID** | **Variant class** | **Source-level Vietnamese estimate** | **Principal global-comparison status** | **Reason/status detail** | **Representation note** |
| --- | --- | --- | --- | --- | --- | --- | --- |
| *CYP2B6* | 516G>T | rs3745274 | Single-rsID variant | 27.08% (20.49–34.87); k=1, N=72 | Included through a harmonized rsID/effect-allele group | Contributed to the principal comparison as CYP2B6 516G>T (two synonymous source labels harmonized) after label and effect-allele harmonization. | Source-level estimates remain separate in Table S3; allele counts were combined only after confirming matching orientation. |
| *CYP2B6* | rs3745274 | rs3745274 | Single-rsID variant | 23.00% (17.71–29.31); k=1, N=100 | Included through a harmonized rsID/effect-allele group | Contributed to the principal comparison as CYP2B6 516G>T (two synonymous source labels harmonized) after label and effect-allele harmonization. | Source-level estimates remain separate in Table S3; allele counts were combined only after confirming matching orientation. |
| *CYP2C19* | *2 | rs4244285 | Star-allele label represented by a single defining rsID | 27.90% (26.57–29.27); k=19, N=2,614 | Included in the principal 20-variant global comparison | Orientation-validated representation used as CYP2C19*2 in Tables S9A–S9B and main Table 3. | Pairwise testing used non-overlapping Vietnamese allele counts and 1000 Genomes Phase 3 superpopulation counts. |
| *CYP2C19* | *3 | rs4986893 | Star-allele label represented by a single defining rsID | 5.74% (4.82–6.82); k=20, N=2,779 | Included in the principal 20-variant global comparison | Orientation-validated representation used as CYP2C19*3 in Tables S9A–S9B and main Table 3. | Pairwise testing used non-overlapping Vietnamese allele counts and 1000 Genomes Phase 3 superpopulation counts. |
| *CYP2D6* | *10 | — | Star-allele/haplotype representation | 43.75% (37.98–49.69); k=1, N=136 | Not directly comparable with a single-rsID reference panel | No unique biallelic rsID/effect-allele representation was available for a valid direct 1000 Genomes comparison. | Retained for clinical interpretation in Tables S7–S8; no single-marker proxy was imposed. |
| *CYP2D6* | rs1065852 C>T marker (source labelled CYP2D6*10) | rs1065852 | Single-rsID variant | 45.07% (37.12–53.28); k=1, N=71 | Not used as a star-allele proxy | The single marker was retained for source traceability but was not used to estimate the corresponding CYP2D6 star allele or assign phenotype. The star-allele representation in Table S8/Table 4 is primary. | CYP2D6 phenotype assignment requires haplotype, copy-number, and structural-variant resolution. |
| *CYP2D6* | *10B | — | Star-allele/haplotype representation | 55.95% (49.78–61.95); k=1, N=126 | Not directly comparable with a single-rsID reference panel | No unique biallelic rsID/effect-allele representation was available for a valid direct 1000 Genomes comparison. | Retained for clinical interpretation in Tables S7–S8; no single-marker proxy was imposed. |
| *CYP2D6* | *36-*10 | — | Tandem structural arrangement | 11.40% (8.15–15.72); k=1, N=136 | Not directly comparable with a single-rsID reference panel | No unique biallelic rsID/effect-allele representation was available for a valid direct 1000 Genomes comparison. | Retained for clinical interpretation in Tables S7–S8; no single-marker proxy was imposed. |
| *CYP2D6* | *5 | — | Whole-gene deletion / copy-number variant | 7.75% (4.38–13.34); k=1, N=71 | Not directly comparable with a single-rsID reference panel | No unique biallelic rsID/effect-allele representation was available for a valid direct 1000 Genomes comparison. | Retained for clinical interpretation in Tables S7–S8; no single-marker proxy was imposed. |
| *CYP2D6* | *5 | — | Whole-gene deletion / copy-number variant | 7.47% (5.51–10.06); k=2, N=262 | Not directly comparable with a single-rsID reference panel | No unique biallelic rsID/effect-allele representation was available for a valid direct 1000 Genomes comparison. | Retained for clinical interpretation in Tables S7–S8; no single-marker proxy was imposed. |
| *CYP3A5* | *3 | rs776746 | Star-allele label represented by a single defining rsID | 60.81% (44.96–74.67); k=7, N=543 | Included in the principal 20-variant global comparison | Orientation-validated representation used as CYP3A5*3 in Tables S9A–S9B and main Table 3. | Pairwise testing used non-overlapping Vietnamese allele counts and 1000 Genomes Phase 3 superpopulation counts. |
| *CYP4F2* | rs2108622 | rs2108622 | Single-rsID variant | 16.50% (12.00–22.27); k=1, N=100 | Not included in the principal 20-variant matrix | A source-level rsID was available, but the representation was not retained in the complete principal matrix after orientation, comparator-availability, redundancy, and analysis-readiness checks. | The Vietnamese source-level estimate remains available in Table S3 and the clinical annotation in Table S7. |
| *HLA-B* | *58:01 | — | HLA allele | 6.02% (3.74–9.55); k=1, N=133 | Not directly comparable with a single-rsID reference panel | No unique biallelic rsID/effect-allele representation was available for a valid direct 1000 Genomes comparison. | Retained for clinical interpretation in Tables S7–S8; no single-marker proxy was imposed. |
| *SLCO1B1* | *15 | rs4149056 | Star-allele/haplotype label with an rsID component | 16.35% (11.94–21.97); k=1, N=104 | Not used as an independent comparison row | The source-reported SLCO1B1 *5/*15 labels involve rs4149056 but were not treated as independent c.521T>C observations. The explicit c.521T>C rows define the principal comparison. | Retained to document source nomenclature and prevent double-counting of the same rsID component. |
| *SLCO1B1* | c.521T>C | rs4149056 | Single-rsID variant | 12.73% (7.36–21.13); k=2, N=204 | Included in the principal 20-variant global comparison | Orientation-validated representation used as SLCO1B1 c.521T>C in Tables S9A–S9B and main Table 3. | Pairwise testing used non-overlapping Vietnamese allele counts and 1000 Genomes Phase 3 superpopulation counts. |
| *UGT1A1* | *28 | rs8175347 | Star-allele label represented by a single defining rsID | 8.42% (5.25–13.24); k=1, N=95 | Not included in the principal 20-variant matrix | A source-level rsID was available, but the representation was not retained in the complete principal matrix after orientation, comparator-availability, redundancy, and analysis-readiness checks. | The Vietnamese source-level estimate remains available in Table S3 and the clinical annotation in Table S7. |
| *UGT1A1* | *28 | rs8175347 | Star-allele label represented by a single defining rsID | 6.00% (2.78–12.48); k=1, N=50 | Not included in the principal 20-variant matrix | A source-level rsID was available, but the representation was not retained in the complete principal matrix after orientation, comparator-availability, redundancy, and analysis-readiness checks. | The Vietnamese source-level estimate remains available in Table S3 and the clinical annotation in Table S7. |
| *UGT1A1* | *6 | rs4148323 | Star-allele label represented by a single defining rsID | 8.76% (3.38–20.88); k=2, N=150 | Included in the principal 20-variant global comparison | Orientation-validated representation used as UGT1A1*6 in Tables S9A–S9B and main Table 3. | Pairwise testing used non-overlapping Vietnamese allele counts and 1000 Genomes Phase 3 superpopulation counts. |
| *VKORC1* | -1173C>T | rs9934438 | Single-rsID variant | 85.33% (80.88–88.89); k=1, N=150 | Included through a harmonized rsID/effect-allele group | Contributed to the principal comparison as VKORC1 1173C>T (minus-sign label variants harmonized) after label and effect-allele harmonization. | Source-level estimates remain separate in Table S3; allele counts were combined only after confirming matching orientation. |
| *VKORC1* | -1639G>A | rs9923231 | Single-rsID variant | 89.91% (81.13–94.86); k=2, N=199 | Included in the principal 20-variant global comparison | Orientation-validated representation used as VKORC1 -1639G>A in Tables S9A–S9B and main Table 3. | Pairwise testing used non-overlapping Vietnamese allele counts and 1000 Genomes Phase 3 superpopulation counts. |
| *VKORC1* | 1173C>T | rs9934438 | Single-rsID variant | 85.71% (77.44–91.30); k=1, N=49 | Included through a harmonized rsID/effect-allele group | Contributed to the principal comparison as VKORC1 1173C>T (minus-sign label variants harmonized) after label and effect-allele harmonization. | Source-level estimates remain separate in Table S3; allele counts were combined only after confirming matching orientation. |
| *CYP2C19* | *17 | rs11188072 | Star-allele label represented by a single defining rsID | 1.04% (0.29–3.72); k=1, N=96 | Not included in the principal 20-variant matrix | A source-level rsID was available, but the representation was not retained in the complete principal matrix after orientation, comparator-availability, redundancy, and analysis-readiness checks. | The Vietnamese source-level estimate remains available in Table S3 and the clinical annotation in Table S7. |
| *CYP2C19* | *17 | rs12248560 | Star-allele label represented by a single defining rsID | 1.37% (0.76–2.46); k=3, N=422 | Included in the principal 20-variant global comparison | Orientation-validated representation used as CYP2C19*17 in Tables S9A–S9B and main Table 3. | Pairwise testing used non-overlapping Vietnamese allele counts and 1000 Genomes Phase 3 superpopulation counts. |
| *CYP2C19* | *5 | rs56337013 | Star-allele label represented by a single defining rsID | 0.50% (0.09–2.78); k=1, N=100 | Not included in the principal 20-variant matrix | A source-level rsID was available, but the representation was not retained in the complete principal matrix after orientation, comparator-availability, redundancy, and analysis-readiness checks. | The Vietnamese source-level estimate remains available in Table S3 and the clinical annotation in Table S7. |
| *CYP2C9* | *2 | rs1799853 | Star-allele label represented by a single defining rsID | 0.22% (0.04–1.08); k=3, N=349 | Included in the principal 20-variant global comparison | Orientation-validated representation used as CYP2C9*2 in Tables S9A–S9B and main Table 3. | Pairwise testing used non-overlapping Vietnamese allele counts and 1000 Genomes Phase 3 superpopulation counts. |
| *CYP2C9* | *3 | rs1057910 | Star-allele label represented by a single defining rsID | 3.27% (2.46–4.32); k=6, N=738 | Included in the principal 20-variant global comparison | Orientation-validated representation used as CYP2C9*3 in Tables S9A–S9B and main Table 3. | Pairwise testing used non-overlapping Vietnamese allele counts and 1000 Genomes Phase 3 superpopulation counts. |
| *CYP2D6* | *10BX2 | — | Copy-number variant | 0.40% (0.07–2.21); k=1, N=126 | Not directly comparable with a single-rsID reference panel | No unique biallelic rsID/effect-allele representation was available for a valid direct 1000 Genomes comparison. | Retained for clinical interpretation in Tables S7–S8; no single-marker proxy was imposed. |
| *CYP2D6* | *13 | — | Star-allele/haplotype representation | 0.74% (0.20–2.64); k=1, N=136 | Not directly comparable with a single-rsID reference panel | No unique biallelic rsID/effect-allele representation was available for a valid direct 1000 Genomes comparison. | Retained for clinical interpretation in Tables S7–S8; no single-marker proxy was imposed. |
| *CYP2D6* | *14 | — | Star-allele/haplotype representation | 0.37% (0.06–2.05); k=1, N=136 | Not directly comparable with a single-rsID reference panel | No unique biallelic rsID/effect-allele representation was available for a valid direct 1000 Genomes comparison. | Retained for clinical interpretation in Tables S7–S8; no single-marker proxy was imposed. |
| *CYP2D6* | *14B | — | Star-allele/haplotype representation | 0.79% (0.22–2.85); k=1, N=126 | Not directly comparable with a single-rsID reference panel | No unique biallelic rsID/effect-allele representation was available for a valid direct 1000 Genomes comparison. | Retained for clinical interpretation in Tables S7–S8; no single-marker proxy was imposed. |
| *CYP2D6* | *15 | — | Star-allele/haplotype representation | 0.37% (0.06–2.05); k=1, N=136 | Not directly comparable with a single-rsID reference panel | No unique biallelic rsID/effect-allele representation was available for a valid direct 1000 Genomes comparison. | Retained for clinical interpretation in Tables S7–S8; no single-marker proxy was imposed. |
| *CYP2D6* | *1XN | — | Copy-number variant | 0.40% (0.07–2.21); k=1, N=126 | Not directly comparable with a single-rsID reference panel | No unique biallelic rsID/effect-allele representation was available for a valid direct 1000 Genomes comparison. | Retained for clinical interpretation in Tables S7–S8; no single-marker proxy was imposed. |
| *CYP2D6* | *36 | — | Star-allele/haplotype representation | 0.37% (0.06–2.05); k=1, N=136 | Not directly comparable with a single-rsID reference panel | No unique biallelic rsID/effect-allele representation was available for a valid direct 1000 Genomes comparison. | Retained for clinical interpretation in Tables S7–S8; no single-marker proxy was imposed. |
| *CYP2D6* | *36-*36-*10 | — | Tandem structural arrangement | 0.37% (0.06–2.05); k=1, N=136 | Not directly comparable with a single-rsID reference panel | No unique biallelic rsID/effect-allele representation was available for a valid direct 1000 Genomes comparison. | Retained for clinical interpretation in Tables S7–S8; no single-marker proxy was imposed. |
| *CYP2D6* | *4 | — | Star-allele/haplotype representation | 0.74% (0.20–2.64); k=1, N=136 | Not directly comparable with a single-rsID reference panel | No unique biallelic rsID/effect-allele representation was available for a valid direct 1000 Genomes comparison. | Retained for clinical interpretation in Tables S7–S8; no single-marker proxy was imposed. |
| *CYP2D6* | rs3892097 G>A marker (source labelled CYP2D6*4) | rs3892097 | Single-rsID variant | 1.45% (0.40–5.13); k=1, N=69 | Not used as a star-allele proxy | The single marker was retained for source traceability but was not used to estimate the corresponding CYP2D6 star allele or assign phenotype. The star-allele representation in Table S8/Table 4 is primary. | CYP2D6 phenotype assignment requires haplotype, copy-number, and structural-variant resolution. |
| *CYP2D6* | *41 | — | Star-allele/haplotype representation | 4.37% (2.45–7.65); k=1, N=126 | Not directly comparable with a single-rsID reference panel | No unique biallelic rsID/effect-allele representation was available for a valid direct 1000 Genomes comparison. | Retained for clinical interpretation in Tables S7–S8; no single-marker proxy was imposed. |
| *CYP2D6* | *49 | — | Star-allele/haplotype representation | 1.19% (0.41–3.44); k=1, N=126 | Not directly comparable with a single-rsID reference panel | No unique biallelic rsID/effect-allele representation was available for a valid direct 1000 Genomes comparison. | Retained for clinical interpretation in Tables S7–S8; no single-marker proxy was imposed. |
| *CYP2D6* | *60 | — | Star-allele/haplotype representation | 0.74% (0.20–2.64); k=1, N=136 | Not directly comparable with a single-rsID reference panel | No unique biallelic rsID/effect-allele representation was available for a valid direct 1000 Genomes comparison. | Retained for clinical interpretation in Tables S7–S8; no single-marker proxy was imposed. |
| *CYP2D6* | *65 | — | Star-allele/haplotype representation | 2.94% (1.50–5.70); k=1, N=136 | Not directly comparable with a single-rsID reference panel | No unique biallelic rsID/effect-allele representation was available for a valid direct 1000 Genomes comparison. | Retained for clinical interpretation in Tables S7–S8; no single-marker proxy was imposed. |
| *CYP2D6* | *67 | — | Star-allele/haplotype representation | 0.37% (0.06–2.05); k=1, N=136 | Not directly comparable with a single-rsID reference panel | No unique biallelic rsID/effect-allele representation was available for a valid direct 1000 Genomes comparison. | Retained for clinical interpretation in Tables S7–S8; no single-marker proxy was imposed. |
| *CYP2D6* | *68-*4 | — | Hybrid structural allele | 0.37% (0.06–2.05); k=1, N=136 | Not directly comparable with a single-rsID reference panel | No unique biallelic rsID/effect-allele representation was available for a valid direct 1000 Genomes comparison. | Retained for clinical interpretation in Tables S7–S8; no single-marker proxy was imposed. |
| *CYP2D6* | *86 | — | Star-allele/haplotype representation | 0.37% (0.06–2.05); k=1, N=136 | Not directly comparable with a single-rsID reference panel | No unique biallelic rsID/effect-allele representation was available for a valid direct 1000 Genomes comparison. | Retained for clinical interpretation in Tables S7–S8; no single-marker proxy was imposed. |
| *CYP3A5* | *6 | rs10264272 | Star-allele label represented by a single defining rsID | 0.00% (0.00–2.60); k=1, N=72 | Not included in the principal 20-variant matrix | A source-level rsID was available, but the representation was not retained in the complete principal matrix after orientation, comparator-availability, redundancy, and analysis-readiness checks. | The Vietnamese source-level estimate remains available in Table S3 and the clinical annotation in Table S7. |
| *CYP3A5* | *7 | rs41303343 | Star-allele label represented by a single defining rsID | 0.00% (0.00–2.60); k=1, N=72 | Not included in the principal 20-variant matrix | A source-level rsID was available, but the representation was not retained in the complete principal matrix after orientation, comparator-availability, redundancy, and analysis-readiness checks. | The Vietnamese source-level estimate remains available in Table S3 and the clinical annotation in Table S7. |
| *CYP3A5* | *8 | rs55817950 | Star-allele label represented by a single defining rsID | 0.00% (0.00–2.60); k=1, N=72 | Not included in the principal 20-variant matrix | A source-level rsID was available, but the representation was not retained in the complete principal matrix after orientation, comparator-availability, redundancy, and analysis-readiness checks. | The Vietnamese source-level estimate remains available in Table S3 and the clinical annotation in Table S7. |
| *NUDT15* | *2;*3 | rs116855232 | Single-rsID variant | 4.50% (2.39–8.33); k=1, N=100 | Not included in the principal 20-variant matrix | A source-level rsID was available, but the representation was not retained in the complete principal matrix after orientation, comparator-availability, redundancy, and analysis-readiness checks. | The Vietnamese source-level estimate remains available in Table S3 and the clinical annotation in Table S7. |
| *SLCO1B1* | *5 | rs4149056 | Star-allele/haplotype label with an rsID component | 0.00% (0.00–1.81); k=1, N=104 | Not used as an independent comparison row | The source-reported SLCO1B1 *5/*15 labels involve rs4149056 but were not treated as independent c.521T>C observations. The explicit c.521T>C rows define the principal comparison. | Retained to document source nomenclature and prevent double-counting of the same rsID component. |
| *TPMT* | rs1142345 | rs1142345 | Single-rsID variant | 2.50% (1.07–5.72); k=1, N=100 | Not included in the principal 20-variant matrix | A source-level rsID was available, but the representation was not retained in the complete principal matrix after orientation, comparator-availability, redundancy, and analysis-readiness checks. | The Vietnamese source-level estimate remains available in Table S3 and the clinical annotation in Table S7. |
| *CYP2D6* | *2 | — | Star-allele/haplotype representation | 7.94% (5.20–11.94); k=1, N=126 | Not directly comparable with a single-rsID reference panel | No unique biallelic rsID/effect-allele representation was available for a valid direct 1000 Genomes comparison. | Retained for clinical interpretation in Tables S7–S8; no single-marker proxy was imposed. |
| *CYP2D6* | *2 | — | Star-allele/haplotype representation | 7.72% (5.10–11.51); k=1, N=136 | Not directly comparable with a single-rsID reference panel | No unique biallelic rsID/effect-allele representation was available for a valid direct 1000 Genomes comparison. | Retained for clinical interpretation in Tables S7–S8; no single-marker proxy was imposed. |
| *ABCB1* | 3435C>T | rs1045642 | Single-rsID variant | 40.28% (32.62–48.44); k=1, N=72 | Included in the principal 20-variant global comparison | Orientation-validated representation used as ABCB1 3435C>T in Tables S9A–S9B and main Table 3. | Pairwise testing used non-overlapping Vietnamese allele counts and 1000 Genomes Phase 3 superpopulation counts. |
| *ABCG2* | rs2231142 | rs2231142 | Single-rsID variant | 36.00% (29.67–42.86); k=1, N=100 | Not included in the principal 20-variant matrix | A source-level rsID was available, but the representation was not retained in the complete principal matrix after orientation, comparator-availability, redundancy, and analysis-readiness checks. | The Vietnamese source-level estimate remains available in Table S3 and the clinical annotation in Table S7. |
| *CYP1B1* | Dup | — | Copy-number variant | 0.97% (0.33–2.82); k=1, N=154 | Not directly comparable with a single-rsID reference panel | No unique biallelic rsID/effect-allele representation was available for a valid direct 1000 Genomes comparison. | Retained for clinical interpretation in Tables S7–S8; no single-marker proxy was imposed. |
| *CYP2A6* | *11 | rs28399447 | Single-rsID variant | 1.50% (0.51–4.32); k=1, N=100 | Not included in the principal 20-variant matrix | A source-level rsID was available, but the representation was not retained in the complete principal matrix after orientation, comparator-availability, redundancy, and analysis-readiness checks. | The Vietnamese source-level estimate remains available in Table S3 and the clinical annotation in Table S7. |
| *CYP2A6* | *15 | rs199916117 | Single-rsID variant | 0.50% (0.09–2.78); k=1, N=100 | Not included in the principal 20-variant matrix | A source-level rsID was available, but the representation was not retained in the complete principal matrix after orientation, comparator-availability, redundancy, and analysis-readiness checks. | The Vietnamese source-level estimate remains available in Table S3 and the clinical annotation in Table S7. |
| *CYP2A6* | *4 | — | Whole-gene deletion / copy-number variant | 11.81% (7.50–18.09); k=1, N=72 | Not directly comparable with a single-rsID reference panel | No unique biallelic rsID/effect-allele representation was available for a valid direct 1000 Genomes comparison. | Retained for clinical interpretation in Tables S7–S8; no single-marker proxy was imposed. |
| *CYP2A6* | *5 | rs5031017 | Single-rsID variant | 14.58% (9.74–21.27); k=1, N=72 | Not included in the principal 20-variant matrix | A source-level rsID was available, but the representation was not retained in the complete principal matrix after orientation, comparator-availability, redundancy, and analysis-readiness checks. | The Vietnamese source-level estimate remains available in Table S3 and the clinical annotation in Table S7. |
| *CYP2A6* | Del | — | Copy-number variant | 11.04% (8.01–15.03); k=1, N=154 | Not directly comparable with a single-rsID reference panel | No unique biallelic rsID/effect-allele representation was available for a valid direct 1000 Genomes comparison. | Retained for clinical interpretation in Tables S7–S8; no single-marker proxy was imposed. |
| *CYP2A6* | rs1809810 | rs1809810 | Single-rsID variant | 2.00% (0.78–5.03); k=1, N=100 | Not included in the principal 20-variant matrix | A source-level rsID was available, but the representation was not retained in the complete principal matrix after orientation, comparator-availability, redundancy, and analysis-readiness checks. | The Vietnamese source-level estimate remains available in Table S3 and the clinical annotation in Table S7. |
| *CYP2B6* | *2 | rs8192709 | Single-rsID variant | 5.50% (3.10–9.58); k=1, N=100 | Not included in the principal 20-variant matrix | A source-level rsID was available, but the representation was not retained in the complete principal matrix after orientation, comparator-availability, redundancy, and analysis-readiness checks. | The Vietnamese source-level estimate remains available in Table S3 and the clinical annotation in Table S7. |
| *CYP2B6* | *4 | rs2279343 | Single-rsID variant | 35.42% (28.08–43.51); k=1, N=72 | Not included in the principal 20-variant matrix | A source-level rsID was available, but the representation was not retained in the complete principal matrix after orientation, comparator-availability, redundancy, and analysis-readiness checks. | The Vietnamese source-level estimate remains available in Table S3 and the clinical annotation in Table S7. |
| *CYP2B6* | *5 | rs3211371 | Single-rsID variant | 0.00% (0.00–2.60); k=1, N=72 | Not included in the principal 20-variant matrix | A source-level rsID was available, but the representation was not retained in the complete principal matrix after orientation, comparator-availability, redundancy, and analysis-readiness checks. | The Vietnamese source-level estimate remains available in Table S3 and the clinical annotation in Table S7. |
| *CYP2C19* | *26 | rs375781227 | Star-allele label represented by a single defining rsID | 0.50% (0.09–2.78); k=1, N=100 | Not included in the principal 20-variant matrix | A source-level rsID was available, but the representation was not retained in the complete principal matrix after orientation, comparator-availability, redundancy, and analysis-readiness checks. | The Vietnamese source-level estimate remains available in Table S3 and the clinical annotation in Table S7. |
| *CYP2C19* | rs3758581 | rs3758581 | Single-rsID variant | 97.50% (94.28–98.93); k=1, N=100 | Not included in the principal 20-variant matrix | A source-level rsID was available, but the representation was not retained in the complete principal matrix after orientation, comparator-availability, redundancy, and analysis-readiness checks. | The Vietnamese source-level estimate remains available in Table S3 and the clinical annotation in Table S7. |
| *CYP2C8* | *11 | rs78637571 | Single-rsID variant | 1.00% (0.27–3.57); k=1, N=100 | Not included in the principal 20-variant matrix | A source-level rsID was available, but the representation was not retained in the complete principal matrix after orientation, comparator-availability, redundancy, and analysis-readiness checks. | The Vietnamese source-level estimate remains available in Table S3 and the clinical annotation in Table S7. |
| *CYP2C9* | *13 | rs72558187 | Star-allele label represented by a single defining rsID | 0.79% (0.22–2.83); k=1, N=127 | Not included in the principal 20-variant matrix | A source-level rsID was available, but the representation was not retained in the complete principal matrix after orientation, comparator-availability, redundancy, and analysis-readiness checks. | The Vietnamese source-level estimate remains available in Table S3 and the clinical annotation in Table S7. |
| *CYP2C9* | *13 | rs72558187 | Star-allele label represented by a single defining rsID | 0.50% (0.09–2.78); k=1, N=100 | Not included in the principal 20-variant matrix | A source-level rsID was available, but the representation was not retained in the complete principal matrix after orientation, comparator-availability, redundancy, and analysis-readiness checks. | The Vietnamese source-level estimate remains available in Table S3 and the clinical annotation in Table S7. |
| *CYP2C9* | Del | — | Copy-number variant | 0.97% (0.33–2.82); k=1, N=154 | Not directly comparable with a single-rsID reference panel | No unique biallelic rsID/effect-allele representation was available for a valid direct 1000 Genomes comparison. | Retained for clinical interpretation in Tables S7–S8; no single-marker proxy was imposed. |
| *CYP2D6* | *21B | — | Star-allele/haplotype representation | 0.40% (0.07–2.21); k=1, N=126 | Not directly comparable with a single-rsID reference panel | No unique biallelic rsID/effect-allele representation was available for a valid direct 1000 Genomes comparison. | Retained for clinical interpretation in Tables S7–S8; no single-marker proxy was imposed. |
| *CYP2D6* | G4268C | rs1135840 | Single-rsID variant | 73.25% (68.71–77.35); k=1, N=200 | Not included in the principal 20-variant matrix | A source-level rsID was available, but the representation was not retained in the complete principal matrix after orientation, comparator-availability, redundancy, and analysis-readiness checks. | The Vietnamese source-level estimate remains available in Table S3 and the clinical annotation in Table S7. |
| *CYP2E1* | *5B | rs2031920 | Single-rsID variant | 17.19% (12.51–23.15); k=1, N=96 | Not included in the principal 20-variant matrix | A source-level rsID was available, but the representation was not retained in the complete principal matrix after orientation, comparator-availability, redundancy, and analysis-readiness checks. | The Vietnamese source-level estimate remains available in Table S3 and the clinical annotation in Table S7. |
| *CYP2E1* | *7B | rs6413420 | Single-rsID variant | 0.00% (0.00–1.96); k=1, N=96 | Not included in the principal 20-variant matrix | A source-level rsID was available, but the representation was not retained in the complete principal matrix after orientation, comparator-availability, redundancy, and analysis-readiness checks. | The Vietnamese source-level estimate remains available in Table S3 and the clinical annotation in Table S7. |
| *CYP2E1* | Del | — | Copy-number variant | 0.32% (0.06–1.82); k=1, N=154 | Not directly comparable with a single-rsID reference panel | No unique biallelic rsID/effect-allele representation was available for a valid direct 1000 Genomes comparison. | Retained for clinical interpretation in Tables S7–S8; no single-marker proxy was imposed. |
| *CYP2E1* | Dup | — | Copy-number variant | 0.97% (0.33–2.82); k=1, N=154 | Not directly comparable with a single-rsID reference panel | No unique biallelic rsID/effect-allele representation was available for a valid direct 1000 Genomes comparison. | Retained for clinical interpretation in Tables S7–S8; no single-marker proxy was imposed. |
| *CYP2J2* | *8 | — | Non-rsID variant representation | 0.00% (0.00–1.19); k=1, N=159 | Not directly comparable with a single-rsID reference panel | No unique biallelic rsID/effect-allele representation was available for a valid direct 1000 Genomes comparison. | Retained for clinical interpretation in Tables S7–S8; no single-marker proxy was imposed. |
| *CYP2J2* | *9 | — | Non-rsID variant representation | 0.00% (0.00–1.19); k=1, N=159 | Not directly comparable with a single-rsID reference panel | No unique biallelic rsID/effect-allele representation was available for a valid direct 1000 Genomes comparison. | Retained for clinical interpretation in Tables S7–S8; no single-marker proxy was imposed. |
| *CYP3A4* | *18 | — | Star-allele/haplotype representation | 0.79% (0.22–2.83); k=1, N=127 | Not directly comparable with a single-rsID reference panel | No unique biallelic rsID/effect-allele representation was available for a valid direct 1000 Genomes comparison. | Retained for clinical interpretation in Tables S7–S8; no single-marker proxy was imposed. |
| *CYP3A4* | *1B | rs2740574 | Single-rsID variant | 2.08% (0.71–5.95); k=1, N=72 | Not included in the principal 20-variant matrix | A source-level rsID was available, but the representation was not retained in the complete principal matrix after orientation, comparator-availability, redundancy, and analysis-readiness checks. | The Vietnamese source-level estimate remains available in Table S3 and the clinical annotation in Table S7. |
| *DPYD* | rs17376848 | rs17376848 | Single-rsID variant | 16.00% (11.57–21.71); k=1, N=100 | Not included in the principal 20-variant matrix | A source-level rsID was available, but the representation was not retained in the complete principal matrix after orientation, comparator-availability, redundancy, and analysis-readiness checks. | The Vietnamese source-level estimate remains available in Table S3 and the clinical annotation in Table S7. |
| *DPYD* | rs1801159 | rs1801159 | Single-rsID variant | 22.50% (17.26–28.77); k=1, N=100 | Not included in the principal 20-variant matrix | A source-level rsID was available, but the representation was not retained in the complete principal matrix after orientation, comparator-availability, redundancy, and analysis-readiness checks. | The Vietnamese source-level estimate remains available in Table S3 and the clinical annotation in Table S7. |
| *DPYD* | rs1801160 | rs1801160 | Single-rsID variant | 2.00% (0.78–5.03); k=1, N=100 | Not included in the principal 20-variant matrix | A source-level rsID was available, but the representation was not retained in the complete principal matrix after orientation, comparator-availability, redundancy, and analysis-readiness checks. | The Vietnamese source-level estimate remains available in Table S3 and the clinical annotation in Table S7. |
| *DPYD* | rs1801265 | rs1801265 | Single-rsID variant | 17.00% (12.43–22.82); k=1, N=100 | Not included in the principal 20-variant matrix | A source-level rsID was available, but the representation was not retained in the complete principal matrix after orientation, comparator-availability, redundancy, and analysis-readiness checks. | The Vietnamese source-level estimate remains available in Table S3 and the clinical annotation in Table S7. |
| *DPYD* | rs2297595 | rs2297595 | Single-rsID variant | 2.50% (1.07–5.72); k=1, N=100 | Not included in the principal 20-variant matrix | A source-level rsID was available, but the representation was not retained in the complete principal matrix after orientation, comparator-availability, redundancy, and analysis-readiness checks. | The Vietnamese source-level estimate remains available in Table S3 and the clinical annotation in Table S7. |
| *HNF4A* | G36S | — | Non-rsID variant representation | 3.60% (1.97–6.49); k=1, N=139 | Not directly comparable with a single-rsID reference panel | No unique biallelic rsID/effect-allele representation was available for a valid direct 1000 Genomes comparison. | Retained for clinical interpretation in Tables S7–S8; no single-marker proxy was imposed. |
| *HNF4A* | G60D | — | Non-rsID variant representation | 0.72% (0.20–2.58); k=1, N=139 | Not directly comparable with a single-rsID reference panel | No unique biallelic rsID/effect-allele representation was available for a valid direct 1000 Genomes comparison. | Retained for clinical interpretation in Tables S7–S8; no single-marker proxy was imposed. |
| *NAT2* | *12 | — | Star-allele/haplotype representation | 3.47% (1.49–7.87); k=1, N=72 | Not directly comparable with a single-rsID reference panel | No unique biallelic rsID/effect-allele representation was available for a valid direct 1000 Genomes comparison. | Retained for clinical interpretation in Tables S7–S8; no single-marker proxy was imposed. |
| *NAT2* | *13 | — | Star-allele/haplotype representation | 30.56% (23.62–38.50); k=1, N=72 | Not directly comparable with a single-rsID reference panel | No unique biallelic rsID/effect-allele representation was available for a valid direct 1000 Genomes comparison. | Retained for clinical interpretation in Tables S7–S8; no single-marker proxy was imposed. |
| *NAT2* | *19 | — | Star-allele/haplotype representation | 1.39% (0.38–4.92); k=1, N=72 | Not directly comparable with a single-rsID reference panel | No unique biallelic rsID/effect-allele representation was available for a valid direct 1000 Genomes comparison. | Retained for clinical interpretation in Tables S7–S8; no single-marker proxy was imposed. |
| *NAT2* | *5 | — | Star-allele/haplotype representation | 2.78% (1.09–6.92); k=1, N=72 | Not directly comparable with a single-rsID reference panel | No unique biallelic rsID/effect-allele representation was available for a valid direct 1000 Genomes comparison. | Retained for clinical interpretation in Tables S7–S8; no single-marker proxy was imposed. |
| *NAT2* | *5 | rs1801280 | Star-allele label represented by a single defining rsID | 5.97% (3.96–8.89); k=4, N=457 | Included in the principal 20-variant global comparison | Orientation-validated representation used as NAT2*5 in Tables S9A–S9B and main Table 3. | Pairwise testing used non-overlapping Vietnamese allele counts and 1000 Genomes Phase 3 superpopulation counts. |
| *NAT2* | *6 | rs1799930 | Star-allele label represented by a single defining rsID | 27.00% (18.69–37.31); k=4, N=457 | Not included in the principal 20-variant matrix | A source-level rsID was available, but the representation was not retained in the complete principal matrix after orientation, comparator-availability, redundancy, and analysis-readiness checks. | The Vietnamese source-level estimate remains available in Table S3 and the clinical annotation in Table S7. |
| *NAT2* | *6A | — | Star-allele/haplotype representation | 17.36% (12.04–24.37); k=1, N=72 | Not directly comparable with a single-rsID reference panel | No unique biallelic rsID/effect-allele representation was available for a valid direct 1000 Genomes comparison. | Retained for clinical interpretation in Tables S7–S8; no single-marker proxy was imposed. |
| *NAT2* | *7 | — | Star-allele/haplotype representation | 0.00% (0.00–2.60); k=1, N=72 | Not directly comparable with a single-rsID reference panel | No unique biallelic rsID/effect-allele representation was available for a valid direct 1000 Genomes comparison. | Retained for clinical interpretation in Tables S7–S8; no single-marker proxy was imposed. |
| *NAT2* | *7 | rs1799931 | Star-allele label represented by a single defining rsID | 17.75% (13.51–22.95); k=4, N=457 | Included in the principal 20-variant global comparison | Orientation-validated representation used as NAT2*7 in Tables S9A–S9B and main Table 3. | Pairwise testing used non-overlapping Vietnamese allele counts and 1000 Genomes Phase 3 superpopulation counts. |
| *NAT2* | rs1208 | rs1208 | Single-rsID variant | 91.00% (86.22–94.23); k=1, N=100 | Included in the principal 20-variant global comparison | Orientation-validated representation used as NAT2 rs1208 in Tables S9A–S9B and main Table 3. | Pairwise testing used non-overlapping Vietnamese allele counts and 1000 Genomes Phase 3 superpopulation counts. |
| *PON1* | Q192R | rs662 | Single-rsID variant | 33.08% (28.63–37.86); k=1, N=198 | Not included in the principal 20-variant matrix | A source-level rsID was available, but the representation was not retained in the complete principal matrix after orientation, comparator-availability, redundancy, and analysis-readiness checks. | The Vietnamese source-level estimate remains available in Table S3 and the clinical annotation in Table S7. |
| *PSORS1C1* | rs9263726 | rs9263726 | Single-rsID variant | 7.89% (5.22–11.77); k=1, N=133 | Not included in the principal 20-variant matrix | A source-level rsID was available, but the representation was not retained in the complete principal matrix after orientation, comparator-availability, redundancy, and analysis-readiness checks. | The Vietnamese source-level estimate remains available in Table S3 and the clinical annotation in Table S7. |
| *SLCO1B1* | c.388A>G | rs2306283 | Single-rsID variant | 78.85% (72.80–83.85); k=1, N=104 | Included through a harmonized rsID/effect-allele group | Contributed to the principal comparison as SLCO1B1 c.388A>G (two synonymous source labels harmonized) after label and effect-allele harmonization. | Source-level estimates remain separate in Table S3; allele counts were combined only after confirming matching orientation. |
| *SLCO1B1* | rs2306283 | rs2306283 | Single-rsID variant | 73.00% (66.46–78.68); k=1, N=100 | Included through a harmonized rsID/effect-allele group | Contributed to the principal comparison as SLCO1B1 c.388A>G (two synonymous source labels harmonized) after label and effect-allele harmonization. | Source-level estimates remain separate in Table S3; allele counts were combined only after confirming matching orientation. |
| *UGT1A1* | c.189C>T | rs191471887 | Single-rsID variant | 0.00% (0.00–3.70); k=1, N=50 | Not included in the principal 20-variant matrix | A source-level rsID was available, but the representation was not retained in the complete principal matrix after orientation, comparator-availability, redundancy, and analysis-readiness checks. | The Vietnamese source-level estimate remains available in Table S3 and the clinical annotation in Table S7. |
| *UGT1A1* | rs1042640C>G | rs1042640 | Single-rsID variant | 25.00% (19.51–31.43); k=1, N=100 | Not included in the principal 20-variant matrix | A source-level rsID was available, but the representation was not retained in the complete principal matrix after orientation, comparator-availability, redundancy, and analysis-readiness checks. | The Vietnamese source-level estimate remains available in Table S3 and the clinical annotation in Table S7. |
| *UGT1A1* | rs10929303C>T | rs10929303 | Single-rsID variant | 25.00% (19.51–31.43); k=1, N=100 | Not included in the principal 20-variant matrix | A source-level rsID was available, but the representation was not retained in the complete principal matrix after orientation, comparator-availability, redundancy, and analysis-readiness checks. | The Vietnamese source-level estimate remains available in Table S3 and the clinical annotation in Table S7. |
| *UGT1A1* | rs8330C>G | rs8330 | Single-rsID variant | 25.00% (19.51–31.43); k=1, N=100 | Not included in the principal 20-variant matrix | A source-level rsID was available, but the representation was not retained in the complete principal matrix after orientation, comparator-availability, redundancy, and analysis-readiness checks. | The Vietnamese source-level estimate remains available in Table S3 and the clinical annotation in Table S7. |
| *VKORC1* | 1542G>C | rs8050894 | Single-rsID variant | 85.71% (77.44–91.30); k=1, N=49 | Included in the principal 20-variant global comparison | Orientation-validated representation used as VKORC1 1542G>C in Tables S9A–S9B and main Table 3. | Pairwise testing used non-overlapping Vietnamese allele counts and 1000 Genomes Phase 3 superpopulation counts. |
| *VKORC1* | 2255C>T | rs2359612 | Single-rsID variant | 85.71% (77.44–91.30); k=1, N=49 | Included in the principal 20-variant global comparison | Orientation-validated representation used as VKORC1 2255C>T in Tables S9A–S9B and main Table 3. | Pairwise testing used non-overlapping Vietnamese allele counts and 1000 Genomes Phase 3 superpopulation counts. |
| *VKORC1* | 3730G>A | rs7294 | Single-rsID variant | 14.29% (8.70–22.56); k=1, N=49 | Included in the principal 20-variant global comparison | Orientation-validated representation used as VKORC1 3730G>A in Tables S9A–S9B and main Table 3. | Pairwise testing used non-overlapping Vietnamese allele counts and 1000 Genomes Phase 3 superpopulation counts. |
| *VKORC1* | 497T>G | rs2884737 | Single-rsID variant | 1.02% (0.18–5.56); k=1, N=49 | Included in the principal 20-variant global comparison | Orientation-validated representation used as VKORC1 497T>G in Tables S9A–S9B and main Table 3. | Pairwise testing used non-overlapping Vietnamese allele counts and 1000 Genomes Phase 3 superpopulation counts. |

The 108 source-level representations comprise 66 rows with an rsID and 42 complex or non-rsID representations. Synonymous source labels can yield more than one status row for a single harmonized rsID; this does not increase the number of principal comparison variants.
